# Unraveling shared susceptibility loci and Mendelian genetic associations linking educational attainment with multiple neuropsychiatric disorders

**DOI:** 10.3389/fpsyt.2023.1303430

**Published:** 2024-01-04

**Authors:** Dongze Chen, Yi Zhou, Yali Zhang, Huatang Zeng, Liqun Wu, Yuyang Liu

**Affiliations:** ^1^Key Laboratory of Carcinogenesis and Translational Research (Ministry of Education/Beijing), Department of Genetics, Peking University Cancer Hospital & Institute, Beijing, China; ^2^Shenzhen Health Development Research and Data Management Center, Shenzhen, China; ^3^Department of Occupational and Environmental Health Sciences, School of Public Health, Peking University, Beijing, China

**Keywords:** educational attainment, neuropsychiatric disorders, genetic correlation, shared genetic architecture, Mendelian randomization

## Abstract

**Background:**

Empirical studies have demonstrated that educational attainment (EA) is associated with neuropsychiatric disorders (NPDs), suggesting a shared etiological basis between them. However, little is known about the shared genetic mechanisms and causality behind such associations.

**Methods:**

This study explored the shared genetic basis and causal relationships between EA and NPDs using the high-definition likelihood (HDL) method, cross phenotype association study (CPASSOC), transcriptome-wide association study (TWAS), and bidirectional Mendelian randomization (MR) with summary-level data for EA (*N* = 293,723) and NPDs (*N* range = 9,725 to 455,258).

**Results:**

Significant genetic correlations between EA and 12 NPDs (*r*_g_ range − 0.49 to 0.35; all *p* < 3.85 × 10^−3^) were observed. CPASSOC identified 37 independent loci shared between EA and NPDs, one of which was novel (rs71351952, mapped gene: *ARFGEF2*). Functional analyses and TWAS found shared genes were enriched in brain tissue, especially in the cerebellum and highlighted the regulatory role of neuronal signaling, purine nucleotide metabolic process, and cAMP-mediated signaling pathways. CPASSOC and TWAS supported the role of three regions of 6q16.1, 3p21.31, and 17q21.31 might account for the shared causes between EA and NPDs. MR confirmed higher genetically predicted EA lower the risk of ADHD (OR_IVW_: 0.50; 95% CI: 0.39 to 0.63) and genetically predicted ADHD decreased the risk of EA (Causal effect: −2.8 months; 95% CI: −3.9 to −1.8).

**Conclusion:**

These findings provided evidence of shared genetics and causation between EA and NPDs, advanced our understanding of EA, and implicated potential biological pathways that might underlie both EA and NPDs.

## Highlights

- The strongest negative genetic correlation was observed between educational attainment and attention deficit/hyperactivity disorder; the strongest positive genetic correlation was observed between educational attainment and bipolar disorder.
- Cross-trait meta-analysis identifies 37 independent genomic loci shared between educational attainment and neuropsychiatric disorders, one of which is novel.
- Associated genetic variants may indicate potential therapeutic targets and implicated signaling pathways in low educational attainment phenotypes and neuropsychiatric disorders.
- Mendelian randomization analysis provided strong evidence in support of low educational attainment causally increasing the risk of attention deficit/hyperactivity disorder, Alzheimer’s dementia, and cannabis use disorder.
- Cross-trait meta-analysis and transcriptome-wide association studies support the role of three particular region 6q16.1, 3p21.31 and 17q21.31 may account for the shared causes between educational attainment and neuropsychiatric disorders.

## 1 Introduction

Educational attainment (EA) is moderately heritable (heritability = 40.0%) from behavior-genetic studies and an important correlate of socioeconomic status, and health outcomes (1, 2). Neuropsychiatric disorders (NPDs) are characterized by affective, cognitive, and behavioral abnormalities stemming from underlying cerebral dysfunction or secondary effects of systemic disease (3). Previous observational studies have found negative associations between EA and risk of attention-deficit/hyperactivity disorder (ADHD) (4), Alzheimer’s dementia (AD) (5), amyotrophic lateral sclerosis (ALS) (6), alcohol use disorders(AUD) (7), cannabis use disorder (CUD) (7), major depression disorder (MDD) (8), obsessive-compulsive disorder (OCD) (9), posttraumatic stress disorder (PTSD) (10), or Tourette’s syndrome (TS) (11). Other observational studies suggest a positive relation between EA and bipolar disorder(BIP) (8), autism spectrum disorder (ASD) (12), or anorexia nervosa (AN) (13). Therefore, not all the correlation results between EA and NPDs exhibit isotropic behavior in nature. ADHD is a prevalent psychiatric condition manifesting as persistent symptoms of inattention, hyperactivity, and impulsivity, potentially leading to worse education outcomes, suggesting a potential bidirectional association between educational attainment (EA) and ADHD. Given that genetic factors are less susceptible to reverse causation nor external confounding factors, it is informative to investigate the bidirectional causality behind these associations using Mendelian randomization. To date, several Mendelian randomization studies have reported causal associations between EA and different NPDs [e.g., AD (14), ADHD (15), ASD (15), ALS (16), and CUD (17)]. The present study will provide further evidence of potential causal associations between EA and NPDs.

More importantly, the genetic underpinnings of EA and NPDs lack a comprehensive understanding. Large-scale genome-wide association studies (GWASs) have identified 74 susceptibility loci for EA (18), including specific loci at 3p21.31 and 17q21.31. These loci are also associated with the risk of neuropsychiatric disorders (NPDs) (19–25), indicating a potential shared genetic architecture between them. Genetic studies can provide valuable insights into the specific biological processes contributing to the co-occurrence of EA and NPDs, potentially serving as targets for early prevention or treatment of NPDs. Yet, our understanding of the shared genetics underlying possible genetic correlations, specific shared genetic variants, and the corresponding biological pathways remains poorly characterized.

In this study, we conducted a large-scale cross-trait genetic analysis to explore the genetic correlations and potential causality between EA and 13 NPDs, and attempted to delineate the precise shared genetic variants and biological pathways linking them.

## 2 Methods

### 2.1 Study design, data source, and study population

**Figure 1:**
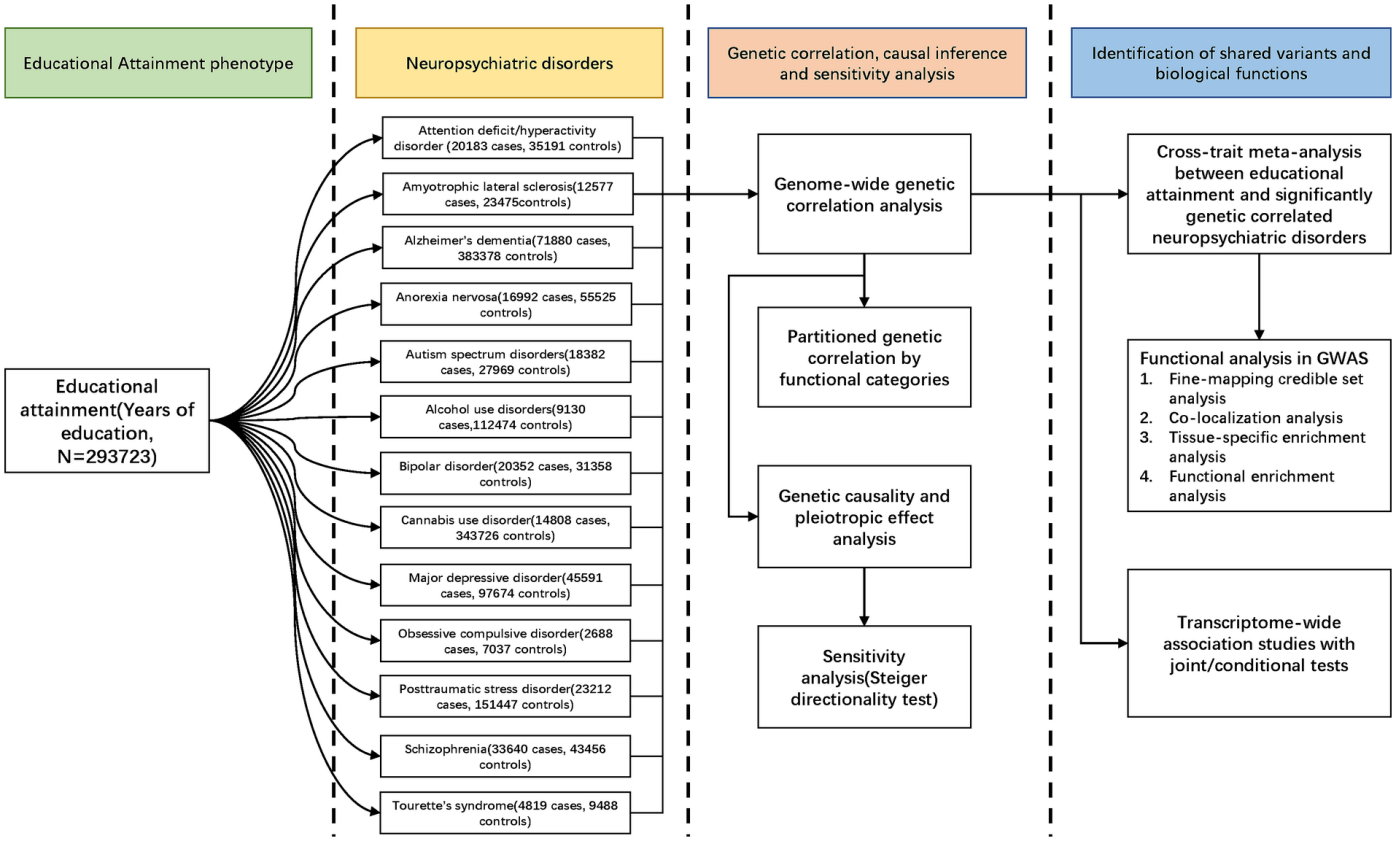
Workflow of the study.

Our overall study design was shown in Figure 1. GWAS summary statistics for EA were obtained from the meta-analysis of 64 independent cohorts (*N* = 293,723) (18). Summary statistics for ADHD, AN, ASD, AUD, BIP, CUD, MDD, OCD, PTSD, SCZ, and TS were available at PGC (psychiatric genomics consortium). Summary statistics for ALS and AD were available at the Project MinE and CTGlab (complex trait genetics lab), respectively. All participants were of European ancestry to avoid confounding bias. Details of study designs and each of the GWASs were present in Supplementary Table S1. The potential sample overlap between the GWASs of EA and NPDs is detailed in the Supplementary methods “sample overlap” section and Supplementary Table S2.

### 2.2 Main analyses

#### 2.2.1 Genetic correlation analysis

Genetic correlations (***r***_g_) were estimated using the High-Definition Likelihood (HDL)^1^ method, which yielded more precise genetic correlation estimates compared to linkage disequilibrium score regression (LDSC)^2^ (26). We estimated ***r***_g_ through comparisons within specific traits, utilizing LD reference data derived from 335,265 individuals of British ancestry from the United Kingdom Biobank (UKB). We also estimated annotation-specific genetic correlations using 13 extensive annotations through partitioned LD score regression (27) (Supplementary methods).

#### 2.2.2 Cross trait meta-analysis

Cross-trait association analysis integrates effect estimates and standard errors from GWAS summary statistics to assess the association between an SNP and two traits (28). We employed the heterogenous variant of the cross-phenotype association test statistic (SHet), calculated using a fixed-effects model, offering enhanced statistical power in the presence of heterogeneity among studies. A significance level of *p* < 3.8 × 10^−9^ (5 × 10^−8^/13 for 13 meta-analysis tests) was employed in the genome-wide cross-trait meta-analysis (Supplementary methods).

#### 2.2.3 Mendelian randomization

Bidirectional Mendelian randomization (MR) analysis was performed to estimate the causal relationship between EA and NPDs (29). Additionally, given the potential impact of horizontal pleiotropy as a confounding factor that may bias the estimates and inflate test statistics in MR analysis, we employed MR-Egger regression and the MR-Pleiotropy Residual Sum and Outlier (MR-PRESSO)^3^ methods (30) to identify and address potential horizontal pleiotropy. We chose the inverse variance-weighted (IVW) method as the primary approach. Bonferroni correction was used for the *p* value (*p* < 0.05/13). A detailed explanation of the causal effect estimates can be found in the Supplementary material.

#### 2.2.4 Transcriptome-wide association studies

To uncover common genes that elucidate the shared mechanisms behind the genetic correlations observed between EA and NPDs, we further delved into the gene level to examine tissue-specific expression-trait associations, as well as shared expression-trait associations between EA and each genetically correlated NPD, using transcriptome-wide association studies (TWAS)^4^ (31). We applied the Benjamini-Hochberg correction to identify significant expression-trait associations, accounting for multiple comparisons across all gene-tissue pairs tested for each trait (totaling ~230,000 gene-tissue pairs in total; significant expression-trait associations were defined as *P*_Benjamini-Hochberg_ < 0.05) (Supplementary methods).

### 2.3 Secondary analyses

#### 2.3.1 Fine-mapping credible set analysis

For each shared locus between EA and NPDs that met the significance criteria in the cross-trait meta-analysis, variants within 500 kb of the lead SNP were extracted, and then a 99% credible set of potentially causal SNPs was identified utilizing the Bayesian likelihood fine-mapping algorithm (FM-summary)^5^ (32). If no variants with a linkage disequilibrium *r*^2^ greater than or equal to 0.9 with the lead SNP were found, the FM-summary cannot be performed.

#### 2.3.2 Co-localization analysis

Summary statistics for variants within 500 kb of the lead SNP at shared loci between EA and NPDs were extracted. We employed the ‘coloc’^6^ package in R to conduct genetic colocalization analysis, calculating the probability of shared common genetic causal variants between the two traits. In this study, loci with a probability (H4) equal or greater than 0.5 were considered to be colocalized (33).

#### 2.3.3 Tissue-specific enrichment analysis

To assess whether the shared gene sets identified through cross-trait meta-analysis exhibited significant enrichment or tissue-specific expression, we performed tissue-specific enrichment analysis (TSEA) using the ‘deTS’^7^ package in R (34) (Supplementary methods).

#### 2.3.4 Functional enrichment analysis

For gaining biological insights into the shared genes identified (*P*_meta_ < 3.8 × 10^−9^) in the cross-trait meta-analysis, we employed the ClueGO^8^ plug-in within the Cytoscape tool to assess the enrichment of gene sets in the Gene Ontology (GO) biological processes and visualized the relationships between genes and GO terms (35, 36). We applied the Benjamini-Hochberg procedure to correct for multiple testing (False Discovery Rate < 0.05).

## 3 Results

### 3.1 Genetic correlations of EA with NPDs

EA was negatively genetically correlated with ADHD (*r*_g_ = −0.49; *p* = 1.36 × 10^−111^), ALS (*r*_g_ = −0.29; *p* = 2.77 × 10^−04^), AD (*r*_g_ = −0.25; *p* = 1.84 × 10^−07^), CUD (*r*_g_ = −0.41; *p* = 5.44 × 10^−5^), MDD (*r*_g_ = −0.19; *p* = 5.52 × 10^−15^), PTSD (*r*_g_ = −0.29; *p* = 3.37 × 10^−10^), and TS (*r*_g_ = −0.17; *p* = 6.87 × 10^−15^), and positively genetically associated with AN (*r*_g_ = 0.23; *p* = 8.15 × 10^−20^), ASD (*r*_g_ = 0.17; *p* = 3.08 × 10^−13^), AUD (*r*_g_ = 0.20; *p* = 3.97 × 10^−19^), BIP (*r*_g_ = 0.20; *p* = 2.27 × 10^−22^), and OCD (*r*_g_ = 0.35; *p* = 1.45 × 10^−7^) (Table 1). No significant genetic correlation was observed for EA and SCZ.

**Table 1:** Genetic correlation of educational attainment with related neuropsychiatric traits (NPDs) using high-definition likelihood method.

NPDs trait	*r*_g_	*r*_g_, SE	*r*_g_, 95% CI	*p* value^*^
ADHD	−0.49	0.022	−0.53 to −0.45	1.36E-111
ALS	−0.29	0.079	−0.44 to −0.14	2.77E-04
AD	−0.25	0.048	−0.34 to −0.16	1.84E-07
AN	0.23	0.025	0.18 to 0.28	8.15E-20
ASD	0.17	0.023	0.12 to 0.22	3.08E-13
AUD	0.20	0.022	0.16 to 0.24	3.97E-19
BIP	0.20	0.021	0.16 to 0.24	2.27E-22
CUD	−0.41	0.043	−0.49 to −0.33	5.44E-22
MDD	−0.19	0.024	−0.24 to −0.14	5.52E-15
OCD	0.35	0.066	0.22 to 0.48	1.45E-07
PTSD	−0.29	0.046	−0.38 to −0.2	3.37E-10
SCZ	0.07	0.021	0.03 to 0.11	1.01E-03
TS	−0.17	0.043	−0.25 to −0.09	6.87E-05

Summary statistics for each trait were combined with Hapmap3 SNPs, excluding the HLA region, to estimate rg. ^*^p value < 0.05/13. ADHD, Attention-deficit/hyperactivity disorder; ALS, Amyotrophic lateral sclerosis; AD, Alzheimer’s dementia; AN, Anorexia nervosa; ASD, Autism spectrum disorders; AUD: Alcohol use disorders; BIP, Bipolar disorder; CUD, Cannabis use disorder; MDD, Major depressive disorder; OCD, Obsessive-compulsive disorder; PTSD, Posttraumatic stress disorder; SCZ, Schizophrenia; TS, Tourette’s syndrome.

To further investigate whether specific functional regions played a disproportionate role in the association between EA and related NPDs, we performed a partitioned genetic correlation analysis across 13 major functional categories (27). In general, annotation-specific LDSC yielded results consistent with the HDL estimate, but indicated a notably stronger genetic correlation at conserved regions and DNase I hypersensitivity sites (DHS) (Supplementary Figure S1; Supplementary Table S3). These findings provided robust evidence of substantial genetic correlations between EA and related NPDs.

### 3.2 Cross-trait meta-analysis between EA and NPDs

The QQ plot and corresponding inflation lambda for the cross-trait GWAS could be found in Supplementary Figure S2. In total, we identified 37 independent loci shared between EA and NPDs (*P*_meta_ < 3.8 × 10^−9^ and single-trait *p* < 1 × 10^−5^) (Table 2). We also determined the credible set of SNPs for each of these shared loci (Supplementary Tables S4–S12). We discovered a previously unreported locus at 20q13.13 (index SNP: rs71351952) that is shared between EA and PTSD. This locus is associated with the protein-coding gene *ARFGEF2*. *ARFGEF2* encodes the large brefeldin A (BFA)-inhibited GEF2 protein (BIG2), which is involved in vesicle and membrane trafficking originating from the trans-Golgi network (37). Vesicle trafficking plays a crucial role in regulating cell proliferation and migration during the development of the human cerebral cortex (38).

**Table 2:** Cross-trait meta-analysis results between educational attainment and neuropsychiatric traits (*P*_CPASSOC_ < 3.8 × 10^−9^ and *P*_single-trait_ < 1 × 10^−5^).

Trait	Index SNP	POS	EA	NEA	Trait 1		Trait 2		*P*_CPASSOC_	Genes
					BETA	*P*	BETA	*P*		
ADHD	rs673253	chr1p34.2	T	C	0.015	8.28E-10	−0.092	1.53E-11	1.17E-14	[KDM4A, PTPRF]
	rs3791101	chr1p34.1	A	G	0.012	7.14E-06	−0.097	5.3E-11	7.98E-11	[MIR6079, ST3GAL3]
	rs549845	chr1p34.2	A	G	0.015	5.78E-08	−0.093	1.38E-10	1.832E-10	[KDM4A, PTPRF, ST3GAL3]
	rs62260755	chr3p21.31	C	G	0.021	3.37E-12	−0.080	6.74E-07	1.026E-12	[CAMKV, TRAIP, CDHR4, MST1R, UBA7]
	rs304137	chr5q14.3	A	G	−0.016	1.78E-10	0.064	1.79E-06	3.228E-16	[MEF2C]
	rs12653396	chr5q14.3	A	T	−0.013	2.14E-07	0.073	1.13E-07	3.725E-11	[LINC00461]
	rs4839923	chr6q16.1	A	G	−0.013	1.1E-07	0.065	1.9E-06	4.286E-11	[MIR2113]
AN	rs13093385	chr3p21.31	A	T	−0.028	3.43E-22	−0.071	1E-06	4.377E-25	[APEH, BSN, MST1, RNF123]
	rs13096760	chr3p21.31	T	C	0.015	5.18E-09	0.065	1.45E-06	2.463E-17	[APEH, BSN, MST1, RNF123]
	rs9821797	chr3p21.31	A	T	0.02	2.4E-07	−0.157	6.99E-15	7.55E-15	[IP6K2, NCKIPSD, MIR4793]
	rs73073015	chr3p21.31	A	G	0.023	9.32E-08	−0.173	3.24E-13	3.887E-13	[DAG1, NICN1, AMT, RHOA, GPX1]
	rs9832454	chr3p21.31	T	C	0.023	2.27E-07	−0.125	1.68E-07	1.428E-11	[GNAI2, GNAT1, MIR566, SEMA3F, RBM5, RBM6]
	rs6967776	chr7q33	A	G	−0.016	1.54E-07	−0.075	9.43E-06	7.207E-10	[EXOC4]
	rs705696	chr12q13.2	A	G	0.018	2.5E-11	−0.064	7.67E-06	9.125E-12	[ERBB3]
ASD	rs9320913	chr6q16.1	A	C	0.024	2.05E-21	0.067	1.51E-06	1.196E-36	[MIR2113]
	rs1106761	chr8q24.3	A	G	−0.017	4.08E-11	−0.065	7.16E-06	1.514E-09	[MROH5]
	rs62057121	chr17q21.31	A	G	−0.018	2.8E-08	0.078	5.94E-06	1.066E-11	[CRHR1, KANSL1-AS1, MAPT, MAPT-AS1, SPPL2C, STH]
AUD	rs2101975	chr4q24	A	G	0.013	2.75E-07	−0.006	5.5E-06	1.655E-14	[TET2, PPA2]
	rs1338549	chr6q16.1	T	G	0.021	8.26E-17	−0.005	6.73E-06	1.4E-30	[MIR2113]
	rs13266268	chr8q24.3	T	C	−0.017	4.1E-11	−0.006	3.95E-06	6.634E-12	[MROH5]
	rs113925422	chr17q21.31	A	T	−0.018	1.02E-07	−0.008	2.6E-08	3.904E-14	[MAPT, MAPT-AS1, SPPL2C, STH]
	rs538628	chr17q21.31	C	G	−0.018	2.16E-08	−0.008	3.27E-08	4.408E-14	[NSF, WNT3]
	rs1879581	chr17q21.31	T	C	0.016	2.1E-06	−0.007	5.16E-06	5.599E-11	[PLEKHM1]
BIP	rs12754946	chr1p31.1	T	C	0.012	1.77E-06	0.066	2.39E-06	1.19E-10	[AK5]
	rs9320913	chr6q16.1	A	C	0.024	2.05E-21	0.070	1.27E-07	5.566E-37	[MIR2113]
	rs10429537	chr9p21.3	C	G	−0.025	1.02E-21	−0.067	1.21E-06	6.978E-19	[ELAVL2]
CUD	rs12122664	chr1p22.2	A	C	0.013	6.22E-07	−0.083	1.92E-06	2.768E-09	[BARHL2]
	rs11711407	chr3p21.31	A	G	−0.020	3.73E-15	0.077	6.41E-06	2.34E-23	[GNAT1, SEMA3F, SLC38A3]
	rs35926495	chr3p21.31	T	C	−0.017	1.31E-11	0.088	2.73E-07	1.608E-12	[GNAT1, GNAI2, LSMEM2, MIR566, NAT6, SEMA3B, SLC38A3]
	rs9467773	chr6p22.2	A	G	0.012	3E-06	−0.074	9.13E-06	1.868E-11	[BTN1A1, HCG11]
	rs7783012	chr7q31.1	A	G	−0.013	4.69E-07	0.101	1.84E-09	4.17E-12	[FOXP2]
MDD	rs7531118	chr1p31.1	T	C	−0.012	1.84E-06	−0.045	2.15E-08	4.392E-10	[RPL31P12]
	rs76025409	chr5q21.2	C	G	−0.012	5.14E-06	0.057	2.32E-11	5.972E-11	[RP11-6 N13.1]
PTSD	rs71351952	chr20q13.13	T	C	0.013	9.6E-06	0.078	4.94E-06	1.772E-09	[ARFGEF2, CSE1L, LOC102723483, STAU1]
TS	rs9401593	chr6q16.1	A	C	−0.024	1.71E-21	0.128	7.87E-07	4.122E-35	[MIR2113]
	rs12154193	chr6q16.1	C	G	−0.017	1.38E-11	0.116	9.62E-06	3.961E-18	[MIR2113]
	rs1619561	chr12q24.31	C	G	−0.023	1.44E-14	0.144	5.06E-06	6.059E-15	[MPHOSPH9]

EA, Effect allele; NEA, non-effect allele; Trait 1 refers to the EA trait; Trait 2 refers to the corresponding neuropsychiatric traits. PCPASSOC means the cross-trait meta-analysis *p* value. EA, educational attainment.; ADHD, Attention deficit/hyperactivity disorder; AN, Anorexia nervosa; ASD, Autism spectrum disorders; AUD, Alcohol use disorders; BIP, Bipolar disorder; CUD, Cannabis use disorder; MDD, Major depressive disorder; PTSD, Posttraumatic stress disorder; TS, Tourette’s syndrome.

We identified seven, seven, three, six, three, five, two, and three independent loci shared between EA and ADHD, AN, ASD, AUD, BIP, CUD, MDD, or TS, respectively (Table 2). We did not find a shared locus between EA and AD, ALS, or OCD. Our findings revealed both commonalities and distinctions in the shared genetic factors between EA and various NPDs. One significant region, located at 6q16.1(near *MIR2113*), was shared between EA with ADHD (index-SNP: rs4839923, ***P***_meta_ = 4.29 × 10^−11^), ASD (index-SNP: rs9320913, ***P***_meta_ = 1.20 × 10^−36^), AUD (index-SNP: rs1338549, ***P***_meta_ = 1.40 × 10^−30^), BIP (index-SNP: rs9320913, ***P***_meta_ = 5.57 × 10^−37^), and TS (index-SNP: rs9401593, ***P***_meta_ = 4.12 × 10^−35^; index-SNP: rs12154193, ***P***_meta_ = 3.96 × 10^−18^). Interestingly, this region was also the strongest shared signal between EA with ASD, AUD, BIP, or TS. *MIR2113* was found to be associated with regulatory elements, including open chromatin, histone modifications, DNase hypersensitive sites, and position weight matrix sites, indicating that the associated SNPs are located within regions of active transcription and may have a regulatory role in transcription (39).

Another prominent multigenic locus, lied in 3p21.31 (mapped genes: *CAMKV*, and *BSN*) was common for EA and ADHD (index-SNP: rs62260755, ***P***_meta_ = 1.03 × 10^−12^), AN (index-SNP: rs13093385, ***P***_meta_ = 4.38 × 10^−25^; index-SNP: rs13096760, ***P***_meta_ = 2.46 × 10^−17^; index-SNP: rs9821797, ***P***_meta_ = 7.55 × 10^−15^; index-SNP: rs73073015, ***P***_meta_ = 3.89 × 10^−13^; index-SNP: rs9832454, ***P***_meta_ = 1.43 × 10^−11^), CUD (index-SNP: rs11711407, ***P***_meta_ = 2.34 × 10^−23^; index-SNP: rs35926495, ***P***_meta_ = 1.61 × 10^−12^). The genes mapped to these loci are all protein-coding and have a role in brain neurology and brain function. For example, *CAMKV* acts as a convergence point for transducing Ca^2+^ signals to the neuronal cytoskeleton through RhoA (Ras homolog gene family, member A), playing a crucial role in normal synaptic transmission that underlies brain function (40). *BSN* (Bassoon) is known to participate in presynaptic transmission in excitatory neurons; its loss results in reduced transmission of the excitatory neurotransmitter glutamate in both the brain and retina (41, 42). The loss of 3p21.31 may contribute to intellectual disability and developmental delays, potentially playing a functional role in the observed intellectual disability and developmental delays in individuals with ADHD and low academic attainment (43). Additional results are available in the Supplementary materials.

Additionally, two loci at chromosome 8q24.3 (*MROH5*, index-SNP: rs1106761, ***P***_meta_ = 1.51 × 10^−9^ for ASD; index-SNP: rs13266268, ***P***_meta_ = 6.63 × 10^−12^ for AUD) and 17q21.31 (*CRHR1* and *WNT3*, index-SNP: rs62057121, ***P***_meta_ = 1.07 × 10^−11^ for ASD; index-SNP: rs113925422, ***P***_meta_ = 3.90 × 10^−14^; index-SNP: rs538628, ***P***_meta_ = 4.41 × 10^−14^; index-SNP: rs1879581, ***P***_meta_ = 1.19 × 10^−10^ for AUD) were common for EA, ASD, and AUD. *MROH5* is a risk factor for ASD (24), but its function is not yet clear. Chromosome 17q21.31 region spanned several genes. For example, *CRHR1* (corticotropin receptor gene) has been linked to alcohol use in both animals and humans (44). *WNT3* has demonstrated its role not only as an axon guidance molecule but also notably as a gradient for retinotopic mapping along the medial-lateral axis (45). Furthermore, we identified additional shared loci between EA and ADHD, AN, AUD, BIP, CUD, or MDD, consistent with previous research (19, 21–23, 25, 46).

### 3.3 Colocalization analysis

Out of the 37 independent loci shared between EA and NPDs, seventeen of these loci exhibited colocalization at the same candidate causal variant (PPH_4_ > 0.5), sixteen loci exhibited colocalization with different candidate causal variants (PPH_3_ > 0.5), while four loci did not exhibit colocalization (Supplementary Table S13).

### 3.4 Tissue-specific enrichment analysis

**Figure 2:**
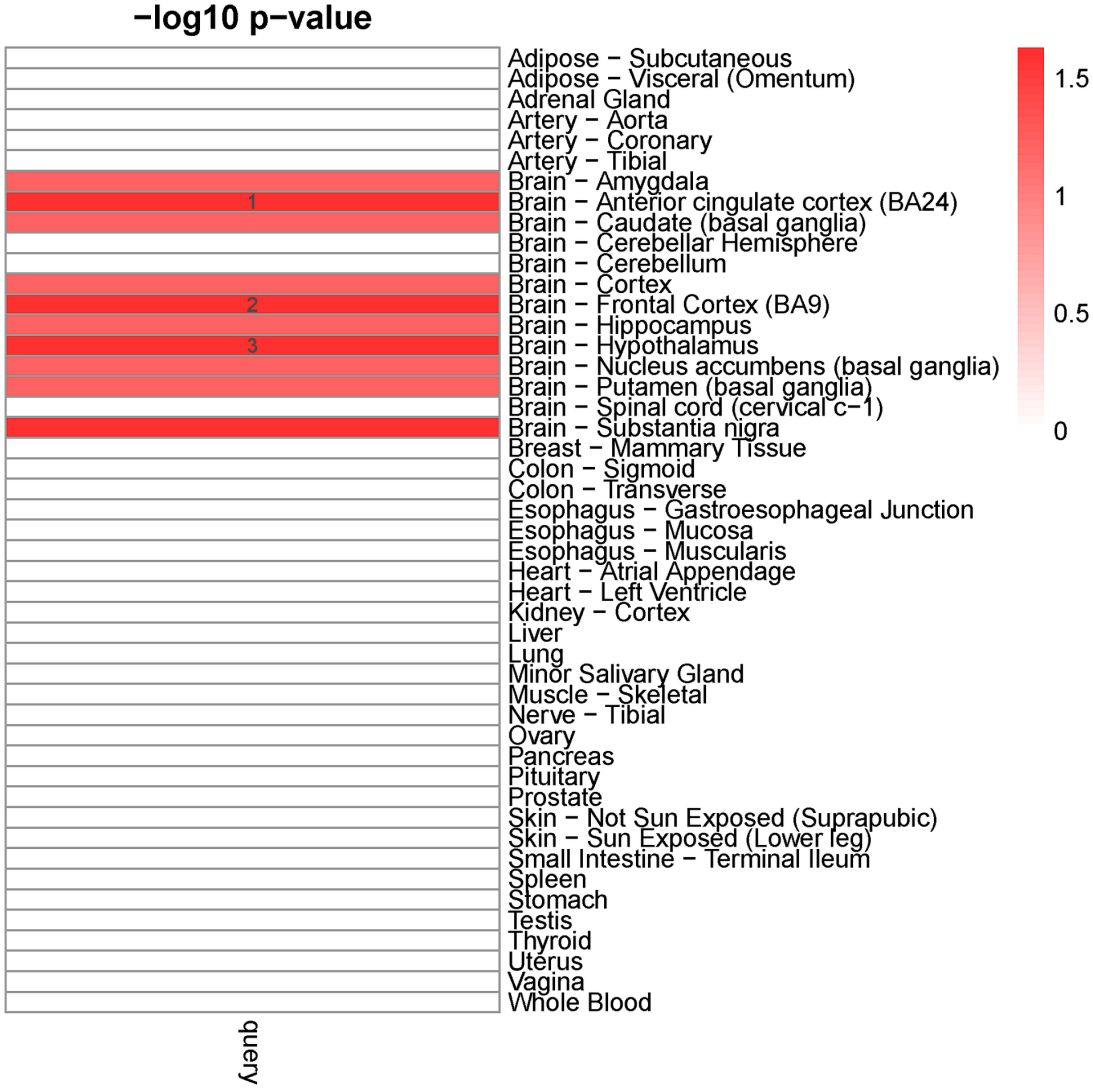
Tissue-specific enrichment result for query gene list. X-axis: groups of genes defined according to different thresholds based on Pascal value of p. Y-axis: 47 GTEx tissues were used as the reference panel. The top three significant tissues (adjusted *p* < 0.1 from Fisher’s Exact Test after Benjamini-Hochberg correction) were marked in numbers.

We detected ten distinct tissues that exhibited significantly enriched expression of genes associated with cross-traits (The query gene list was extracted from the shared genes between EA with ADHD, ASD, AUD, BIP, and TS) (Figure 2). The three most significantly enriched tissues were all part of the nervous system, including the brain-anterior cingulate cortex, brain-frontal cortex, and brain-hypothalamus.

### 3.5 Functional enrichment analysis

**Figure 3:**
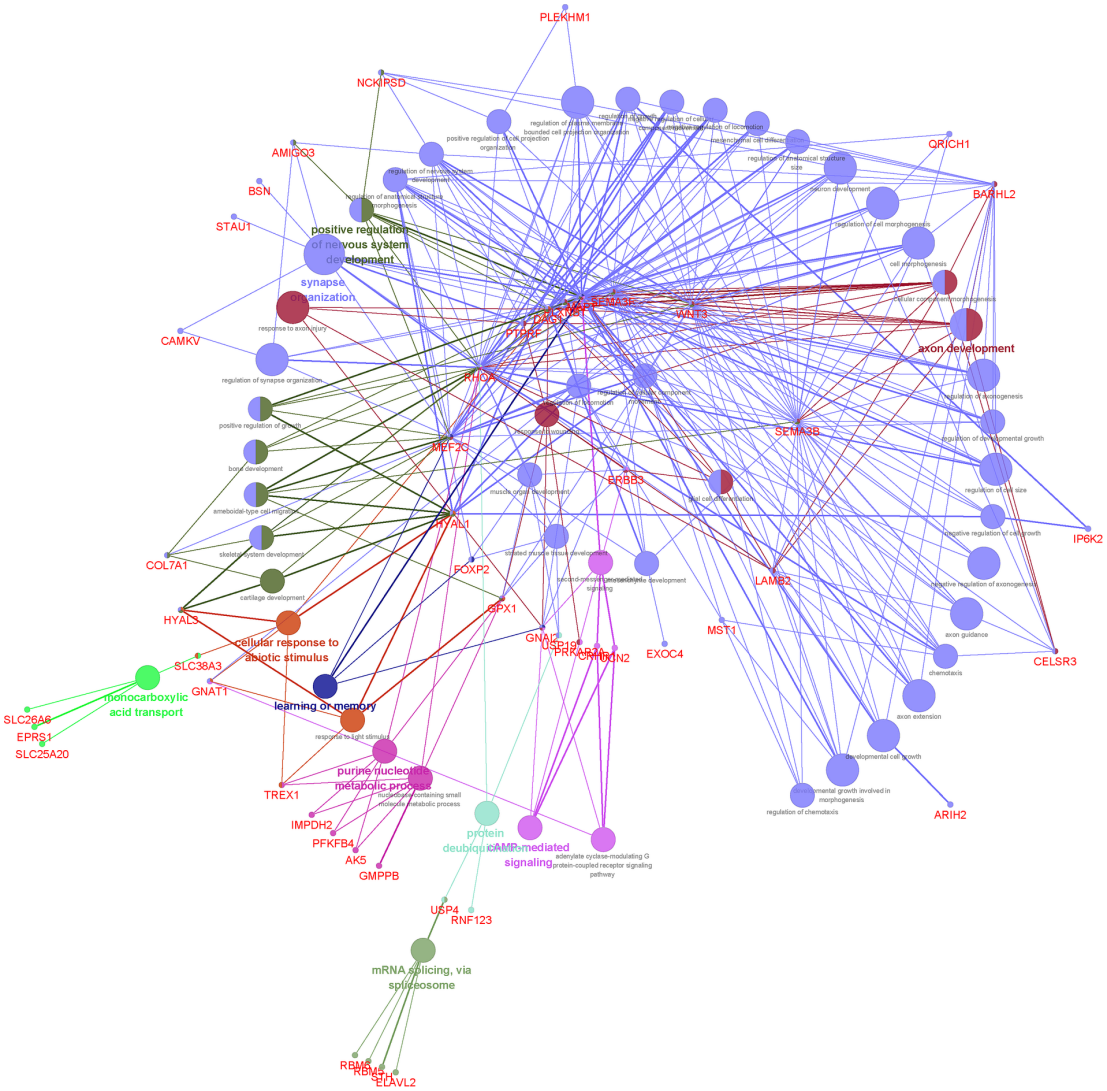
Functional enrichment for the consensus set. Functional enrichment analysis results using the ClueGO method in Cytoscape (34). Each dot represented a gene or a GO term. Dots in the same color were considered from the same functional group by ClueGO annotation. Gene names were highlighted in red. Each edge indicated the gene was a component gene of the linked GO term.

Our finding showed that the 117 genes shared between EA and NPDs were enriched in axon development, positive regulation of nervous system development, synapse organization, learning or memory, purine nucleotide metabolic process, cAMP (cyclic adenosine monophosphate)-mediated signaling, cellular response to abiotic stimulus, protein deubiquitinating, monocarboxylic acid transport, and mRNA splicing, via spliceosome (Figure 3; Supplementary Table S14).

### 3.6 Causal inference

We then tested the causality between EA and related NPDs using Two sample MR.^9^ MR-Egger regression and MR-PRESSO method indicated little significant horizontal pleiotropy (most ***P***_MR_Egger_ > 0.05) (Supplementary Tables S15, S16). Table 3 indicates a causal relationship between EA and ADHD. The findings suggest that a genetic predisposition to higher EA is associated with a reduced risk of ADHD [OR_IVW_ (odds ratio): 0.50 per SD increment; 95% CI: 0.39–0.63; ***P***_IVW_ = 1.62 × 10^−8^], and ADHD is estimated to have a causal effect of −2.8 months on EA, corresponding to a doubling in ADHD prevalence [Causal effect = −2.8; 95%CI: −3.9 to −1.8; ***P***_IVW_ = 8.46 × 10^−8^]. In addition, there was evidence suggesting a negative causal effect of genetic predisposition to higher EA on a reduced risk of AD [OR_IVW_: 0.94 per SD increment; 95%CI: 0.91 to 0.98; ***P***_IVW_ = 0.0028] and CUD [OR_IVW_: 0.50 per SD increment; 95%CI: 0.35 to 0.73; ***P***_IVW_ = 0.0003]. Moreover, we observed that higher EA had a positive causal effect on ASD [OR_IVW_: 1.45 per SD increment; 95%CI: 1.15 to 1.83; ***P***_IVW_ = 0.0019]. Other related NPDs (including ALS, AN, AUD, BIP, MDD, OCD, PTSD, SCZ, and TS) were not related to higher EA and vice versa after correction. Sensitivity analysis confirmed the reliability of our results, indicating that they were not influenced by horizontal pleiotropy or outliers (Supplementary Table S17).

**Table 3:** Causal inference between educational attainment and neuropsychiatric traits using two-sample MR.

Outcome	Direction	Method	N_snp	Causal_Effect_Size	95% CI	*p*_value
AD	Forward	Inverse variance weighted	63	−0.059	(−0.098, −0.020)	0.0028
	Reverse	Inverse variance weighted	24	0.030	(−0.029, 0.088)	0.3191
ADHD	Forward	Inverse variance weighted	54	−0.699	(−0.941, −0.456)	1.62E-08
	Reverse	Inverse variance weighted	8	−0.095	(−0.130, −0.060)	8.46E-08
ALS	Forward	Inverse variance weighted	58	−0.264	(−0.548, 0.019)	0.0679
	Reverse	Inverse variance weighted	4	−0.015	(−0.037, 0.007)	0.1799
AN	Forward	Inverse variance weighted	44	0.333	(0.078, 0.588)	0.01055
	Reverse	Inverse variance weighted	11	0.012	(−0.025, 0.049)	0.5185
ASD	Forward	Inverse variance weighted	53	0.369	(0.136, 0.602)	0.0019
	Reverse	Inverse variance weighted	9	0.004	(−0.051, 0.059)	0.8804
AUD	Forward	Inverse variance weighted	61	0.040	(0.009, 0.071)	0.01048
	Reverse	Inverse variance weighted	6	−0.178	(−0.539, 0.183)	0.3344
BIP	Forward	Inverse variance weighted	47	0.245	(−0.016, 0.506)	0.0662
	Reverse	Inverse variance weighted	11	0.0360	(0.000, 0.071)	0.0481
CUD	Forward	Inverse variance weighted	63	−0.688	(−1.058, −0.318)	0.0003
	Reverse	Inverse variance weighted	11	−0.039	(−0.065, −0.014)	0.0024
MDD	Forward	Inverse variance weighted	39	−0.075	(−0.290, 0.140)	0.4956
	Reverse	Inverse variance weighted	4	0.010	(−0.133, 0.153)	0.8921
OCD	Forward	Inverse variance weighted	46	−0.072	(−0.678, 0.534)	0.8150
	Reverse	Inverse variance weighted	11	−0.004	(−0.015, 0.006)	0.3963
PTSD	Forward	Inverse variance weighted	61	0.0160	(−0.222, 0.254)	0.8954
	Reverse	Inverse variance weighted	4	−0.034	(−0.083, 0.014)	0.1662
SCZ	Forward	Inverse variance weighted	43	0.152	(−0.063, 0.368)	0.1661
	Reverse	Inverse variance weighted	65	0.003	(−0.017, 0.023)	0.7721
TS	Forward	Inverse variance weighted	42	0.009	(−0.488, 0.505)	0.9731
	Reverse	Inverse variance weighted	16	−0.015	(−0.043,0.014)	0.3224

*, The causal effect size was the beta coefficient from linear or logistic regression models for corresponding outcome; MR: Mendelian randomization; Direction: Forward means the causal effect size of EA on neuropsychiatric traits, Reverse means the causal effect size of neuropsychiatric traits on EA; N_snp: number of instrumental variables; The threshold of significance was at the Bonferroni-adjusted level p-value < 0.0038(0.05/13). AD, Alzheimer’s dementia; ADHD, Attention deficit/hyperactivity disorder; ALS, Amyotrophic lateral sclerosis; AN, Anorexia nervosa; ASD:,Autism spectrum disorders; AUD, Alcohol use disorders; BIP, Bipolar disorder; CUD, Cannabis use disorder; MDD, Major depressive disorder; OCD, Obsessive compulsive disorder; PTSD, Posttraumatic stress disorder; TS, Tourette’s syndrome.

### 3.7 Single trait TWAS and shared genetics between EA and NPDs from TWAS

We then conducted a gene-level analysis to investigate the common genes expressed in both EA and related NPDs. In total, we identified 5,874 gene-tissue pairs that exhibited a significant association with EA across 48 GTEx tissues after applying the Benjamini-Hochberg correction. Additionally, we found 495, 2,108, 962, 487, 1,227, 1707, 128, and 207 gene-tissue pairs associated with ADHD, AD, AN, ASD, AUD, BIP, CUD, and MDD, respectively (Supplementary Figures S3–S5).

**Figure 4:**
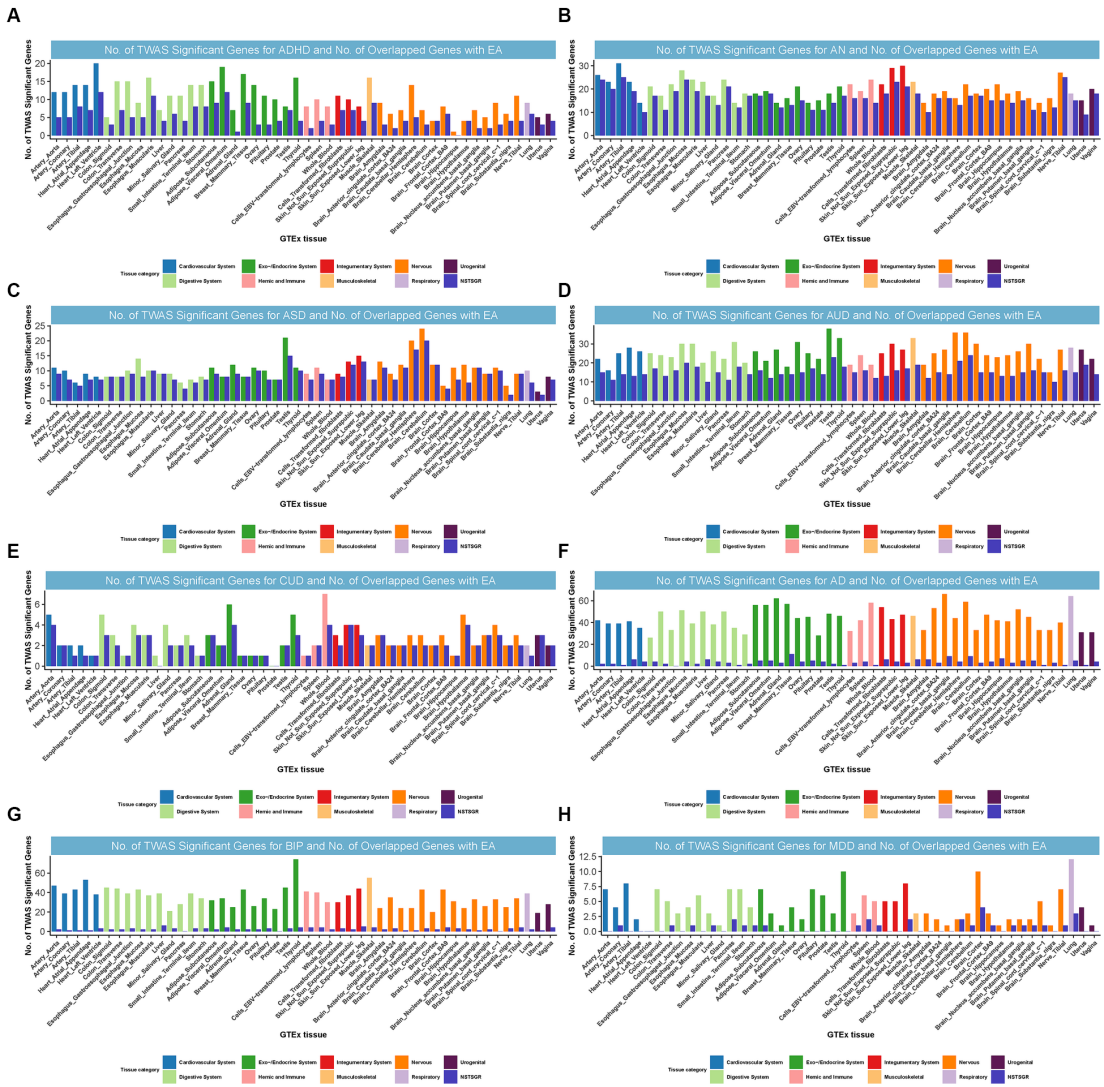
Numbers of significant genes related to ADHD, AN, ASD, AUD, CUD, AD, BIP, and MDD, and the number of shared genes with EA. Significant genes were identified by *P*_BH_ < 0.05. GTEx = genotype-tissue expression project; GWAS = genome-wide association studies; TWAS = transcriptome-wide association study; NSTSGR = Number of Shared TWAS Significant Genes with EA; EA: Educational attainment; ADHD: Attention-deficit/hyperactivity disorder; AD: Alzheimer’s dementia; AN: Anorexia nervosa; ASD: Autism spectrum disorders; AUD: Alcohol use disorders; BIP: Bipolar disorder; CUD: Cannabis use disorder; MDD: Major depressive disorder. **(A)**: No. of TWAS Significant Genes for ADHD and No. of Overlapped Genes with EA. **(B)**: No. of TWAS Significant Genes for AN and No. of Overlapped Genes with EA. **(C)**: No. of TWAS Significant Genes for ASD and No. of Overlapped Genes with EA. **(D)**: No. of TWAS Significant Genes for AUD and No. of Overlapped Genes with EA. **(E)**: No. of TWAS Significant Genes for CUD and No. of Overlapped Genes with EA. **(F)**: No. of TWAS Significant Genes for AD and No. of Overlapped Genes with EA. **(G)**: No. of TWAS Significant Genes for BIP and No. of Overlapped Genes with EA. **(H)**: No. of TWAS Significant Genes for MDD and No. of Overlapped Genes with EA.

We identified 55, 86, 34, 49, 26, 39, 51, and 16 TWAS-significant genes shared between EA and ADHD, AN, ASD, AUD, CUD, AD, BIP, or MDD, respectively (Supplementary Table S18). Most of these genes were observed in tissues from the nervous system, cardiovascular system, endocrine system, digestive system, and musculoskeletal system (Figure 4). We did not observe TWAS-significant genes shared between EA and ALS, OCD, PTSD, or TS.

Interestingly, in strong alignment with the findings from CPASSOC,^10^ certain loci were common to both EA and multiple NPDs. For instance, locus 3p21.31, harboring multiple genes, was commonly TWAS significant for EA, ADHD, AN, BIP, CUD, and MDD and was simultaneously the most prominent shared locus among EA and AN, BIP, or CUD. Notably, we found *NAT6* (3p21.31) was commonly significant for EA, ADHD, AN, BIP, and CUD from TWAS. *NAT6* encodes a member of the N-acetyltransferase family. N-acetyltransferases modify proteins by transferring acetyl groups from acetyl-CoA to the N-termini of protein substrates. The encoded protein is a cytoplasmic N-acetyltransferase with a substrate specificity for N-termini that is enriched for acidic residues (47). This gene was significantly related to CUD through genetically regulated gene expression (23). Locus 17q21.31 was co-significant for EA, ASD, and AUD and was also the most significant shared locus between them (Supplementary results).

## 4 Discussion

Our analysis yielded four main findings. First and foremost, we presented compelling evidence demonstrating a significant genetic correlation between EA and 12 NPDs using the HDL method and partitioned LDSC. Second, cross-trait meta-analysis identified independent shared loci between EA and NPDs. Functional analysis revealed that these shared loci were predominantly enriched in brain tissues, associated with neurogenesis, synapse formation, and the purine nucleotide metabolic and cAMP-mediated signaling pathways. Third, TWAS uncovered shared gene expressions between EA and NPDs in tissues from the nervous system, endocrine system, and digestive system. Fourth, MR analyses revealed bidirectional causality between EA and ADHD, i.e., higher genetically predicted EA was associated with lower risk of ADHD, and genetically predicted ADHD was associated with lower EA. Our results advanced our understanding of the shared genetic contributions to EA and NPDs, elucidated the causal connections between these traits, provided insight into the potential regulatory roles of shared genetics, and shed light on the etiologies and mechanisms underlying the coexistence of low EA and NPDs.

Low educational attainment is indicative of the neurobiological abnormalities that underlie NPDs and has been proposed as a potential premorbid marker for these disorders (48). Based on our results, we found significantly negative genetic correlations of EA with ADHD, ALS, AD, CUD, MDD, PTSD, and TS, and positive genetic associations of EA with AN, ASD, AUD, BIP, and OCD. The positive association of EA with AN, ASD, AUD, BIP, and OCD may be driven by perfectionism (49). Meta-analysis suggested that perfectionism contributes to higher academic achievement, and several studies have confirmed that higher levels of perfectionism are associated with a greater risk of AN (50), ASD (51), AUD (52), BIP (53), and OCD (54). We observed that EA exhibited a positive association with SCZ (*r*_g_ = 0.07, *p* = 1.01 × 10^−3^), although this significance was no longer present after Bonferroni correction. However, recent studies have demonstrated a negative association between higher EA and SCZ using the proxy phenotype method (55). The mechanisms underlying this counterintuitive finding of an inverse relationship between EA and SCZ remained unknown. It may be partially attributed to the genetic heterogeneity of EA, which can be deconstructed into sub-phenotypes (including higher IQ, greater openness, and increased conscientiousness) that exhibit imperfect genetic correlations among themselves. If the various symptoms of SCZ also possess non-identical genetic architectures, this could lead to conflicting phenotypic and genetic correlation outcomes (56).

According to a previous study, the hallmark biology process of EA was neural development (18). It was well known that excitatory synapse, developing brain, neuronal morphogenesis, and central nervous system morphogenesis were significant biological pathways for ADHD (19), ASD (24), MDD (22), and CUD (23), respectively. Several of these pathways were associated with EA regulation, consistent with the results of our functional enrichment analysis, suggesting a common biological mechanism for these disorders. Shared genes identified from CPASSOC and TWAS also supported the shared biological pathways between EA and NPDs. Noteworthy, our findings emphasized the potentially intriguing roles of novel associations involving *ARFGEF2* in the context of the relationship between EA and PTSD. Additionally, our attention was directed towards genes located within the 6q16.1, 3p21.31, and 17q21.31 loci.

Genes shared between EA and NPDs exhibited enrichment in various brain tissues, notably the cerebellum, suggesting that these disorders may be attributed to nervous system dysfunction. For instance, mutations in the *ARFGEF2* gene, responsible for encoding the BIG2 protein and identified in patients with autosomal recessive periventricular heterotopia with microcephaly, are closely associated with neuronal migration disorders (57). Hong et al. (58) reported that BIG2 is a RhoA interactor that regulates dendrite morphogenesis and Golgi polarization during neuronal cell development. BIG2 also contains A-kinase anchoring protein (AKAP) sequences that can act as scaffolds for multimolecular assemblies that facilitate and limit cAMP signaling temporally and spatially (59) indicating the axon development and cAMP-mediated signaling pathways shared between EA and PTSD. Notably, *MIR2113* (6q16.1) has been associated with accelerated decline in episodic memory (60) and neurodegenerative diseases such as AD and ALS (61). It is also biologically and genetically implicated in EA (62), ASD (24), AUD (63), BIP (64), and TS (65). The genealogical study demonstrated that the 6q16.1 deletion will cause developmental delay and intellectual disability (66).

Additionally, a microdeletion located at 3p21.31 was previously associated with mild cognitive disability (67), CNS abnormalities, and developmental delay (43). A comprehensive transcriptomic study identified *CAMKV* within the synaptic neuropil (68), and its protein synthesis is governed by neuronal activity. Moreover, the knockdown of CAMKV in mouse hippocampal CA1 pyramidal neurons adversely affects synaptic transmission and plasticity *in vivo*, leading to hyperactivity and spatial memory impairment (40). Okerlund et al. (69) discovered that *BSN* plays a pivotal role in the local regulation of presynaptic autophagy, a process dependent on poly-ubiquitination but not on the E3 ubiquitin ligase Siah1. A recent study found that neuroinflammation leads to the induction and toxic accumulation of the synaptic protein BSN in the neuronal somata of mice and patients with multiple sclerosis, implying that BSN may contribute to neurodegeneration (70). In particular, locus 3p21.31 was also confirmed to be shared for EA, ADHD, AN, BIP, CUD, and MDD using TWAS.

Moreover, *CHRH1* and *WNT3* have been shown to have strong associations with AUD (25) and ASD (24). Recurrent microdeletions of 17q21.31, which include the *CHRH1* gene, have been identified as a relatively common cause of intellectual disability. This underscores the potential involvement of *CHRH1* in neurodevelopmental disorders (71). *WNT3* encoded the proto-oncogene protein Wnt-3, which played a crucial role in brain development and patterning. It was also implicated in the pathogenesis of diseases such as Parkinson’s, Alzheimer’s, and schizophrenia (72). More importantly, 17q21.31 was also co-significant for EA, ASD, and AUD from TWAS. CPASSOC, functional analysis, and TWAS underlined the molecular pathways in the regulation of axon development, nervous system development, synapse organization, learning or memory, purine nucleotide metabolic process, cAMP-mediated signaling, and protein deubiquitinating, further underscores the potential significance of neurological dysregulation throughout the entire body in predisposition to both low EA and NPDs.

In addition to the nervous system, what particularly interested us is the enrichment of tissues in the cardiovascular system, endocrine system, and musculoskeletal system. This suggested that the shared pathway between EA and NPDs may have significant functions beyond brain tissue. For example, AN was associated with cardiac abnormalities including hypotension, and dysregulation in peripheral vascular contractility (73). Medication treatment in ADHD patients was associated with an increased risk of arrhythmias and myocardial infarctions (74). EA may influence the risk of AN and ADHD through alterations in blood pressure or high-density lipoprotein cholesterol in the cardiovascular system (75). Consistent with the result from Shadrin (76), musculoskeletal tissue was TWAS significant tissue between EA and ADHD. Taken together, TWAS provided additional evidence that the expressed enriched genes may not be specifically expressed in brain tissue; rather, it appeared to be prevalent throughout the body in brain, metabolic, musculoskeletal, and endocrine-related organs.

In our bidirectional MR analysis, we observed a causal relationship between higher EA and a reduced risk of ADHD (OR_IVW_ = 0.33). This finding aligns with results obtained from Mendelian randomization analysis, which indicated a 0.3-fold decrease in ADHD risk per SD increase in EA (15). Conversely, the onset of ADHD was associated with a subsequent reduction in EA (Causal effect = −2.8 months), suggesting that targeting EA could be a promising therapeutic strategy for ADHD. The bidirectional causal association between EA and ADHD is highly similar to the results of a recent MR study conducted by Artigas et al. (77). Research suggested that attentional strengths may be a potential phenotype that simultaneously drives the association between lower EA and ADHD (51, 78).Our results also provided evidence of a causal relationship between higher EA and a decreased risk of AD and CUD, while increasing the risk of ASD. These findings were in line with discoveries from recent studies (14, 15, 79, 80). MR analysis demonstrated that lower EA in individuals may exacerbate the risk of NPDs (especially for ADHD, AD, and CUD). Therefore, to improve this relationship, increased efforts to reduce dropout rates and create more educational opportunities are critical to ensure optimal long-term EA for this vulnerable group (81). The mechanisms underlying the causality between EA and NPDs remain to be elucidated, but the shared CPASSOC and TWAS genes could offer new insights into revealing shared etiologies and mechanisms between them.

Our results have provided clear insights into the shared genetic architecture between EA and NPDs. We emphasized the importance of implementing NPD prevention programs for individuals with lower levels of EA. However, our study has several limitations. First, while TWAS has enhanced the power to detect significant expression trait associations, the relatively smaller sample size for metabolic traits and GTEx reference panels in certain tissues may not be sufficient to detect signals with small to moderate effects. Second, although we observed higher instrumental estimates from EA to NPDs than in the reverse path, we cannot rule out the possibility that EA mediates the effects of certain upstream latent factors on NPDs. Third, given that the traits we investigated involved EA, it cannot be ruled out that the detected causal effects were caused by dynastic effects or assortative mating (82). Fourth, our study was confined to individuals of European ancestry, and the shared genetics in other ethnic groups remain uncertain. Therefore, future research should encompass other ethnic groups, and exploration into how mediating variables, such as family background, personality characteristics, and intelligence, influence the relationship between EA and NPDs is encouraged.

In conclusion, our study provided evidence of significant genetic correlations and instrumental causality between EA and NPDs. The identification of genetic loci associated with both EA and the risk of NPDs presents therapeutic opportunities to enhance EA and reduce the risk of NPDs. Furthermore, our study advanced our understanding of EA and revealed a common etiology for the coexistence of low EA and NPDs.

## Data availability statement

The original contributions presented in the study are included in the article/Supplementary material, further inquiries can be directed to the corresponding authors.

## Author contributions

DC: Conceptualization, Data curation, Formal analysis, Investigation, Software, Supervision, Visualization, Writing – original draft, Writing – review & editing. YiZ: Conceptualization, Data curation, Investigation, Resources, Software, Supervision, Writing – original draft, Writing – review & editing. YaZ: Formal analysis, Investigation, Methodology, Project administration, Writing – review & editing. HZ: Conceptualization, Data curation, Investigation, Writing – review & editing. LW: Conceptualization, Data curation, Investigation, Supervision, Writing – review & editing. YL: Conceptualization, Data curation, Investigation, Writing – review & editing.

## Footnotes

^1^https://github.com/zhenin/HDL

^2^https://github.com/bulik/ldsc

^3^https://github.com/rondolab/MR-PRESSO

^4^http://gusevlab.org/projects/fusion/

^5^https://github.com/hailianghuang/FM-summary

^6^https://github.com/chr1swallace/coloc

^7^https://github.com/bsml320/deTS

^8^http://apps.cytoscape.org/apps/cluego

^9^https://github.com/mrcieu/TwoSampleMR

^10^http://hal.case.edu/~xxz10/zhu-web/

## Glossary

EA: Educational attainment
NPDs: Neuropsychiatric disorders
ADHD: Attention-deficit/hyperactivity disorder
ALS: Amyotrophic lateral sclerosis
AD: Alzheimer’s dementia
AN: Anorexia nervosa
ASD: Autism spectrum disorder
AUD: Alcohol use disorder
BIP: Bipolar disorder
CUD: Cannabis use disorder
MDD: Major depressive disorder
OCD: Obsessive-compulsive disorder
PTSD: Posttraumatic stress disorder
SCZ: Schizophrenia
TS: Tourette’s syndrome
HDL: High-definition likelihood
GWAS: Genome-wide association study
LDSC: Linkage disequilibrium score regression
CPASSOC: Cross phenotype association study
TSEA: Tissue-specific enrichment analysis
MR: Mendelian randomization
TWAS: Transcriptome-wide association study
SNP: Single nucleotide polymorphism
GTEx: Genotype-tissue expression portal
