# Supplementary material for: Unraveling shared susceptibility loci and Mendelian genetic associations linking educational attainment with multiple neuropsychiatric disorders
Source: Front Psychiatry. 2024 Jan 4;14:1303430. doi: 10.3389/fpsyt.2023.1303430 (PMC10797721; doi:10.3389/fpsyt.2023.1303430)

**Supplementary material**

**Contents**

[Online Methods 3](#_Toc152973560)

[Summary statistics for educational attainment and neuropsychiatric traits, quality control 3](#_Toc152973561)

[Study design 3](#_Toc152973562)

[Sample overlap 3](#_Toc152973563)

[Partitioned genetic correlation 4](#_Toc152973564)

[Cross trait meta-analysis 4](#_Toc152973565)

[Tissue-specific enrichment analysis 4](#_Toc152973566)

[Mendelian randomization 5](#_Toc152973567)

[Interpretation of the causal effect estimates 6](#_Toc152973568)

[Transcriptome-wide association studies 6](#_Toc152973569)

[Online Results 7](#_Toc152973570)

[The ClueGO log in our experiment 7](#_Toc152973571)

[Shared genetics between EA and neuropsychiatric disorders from TWAS 8](#_Toc152973572)

[References 10](#_Toc152973573)

[Supplementary Table 12](#_Toc152973574)

[Supplementary Table 1. Information of summary statistics used in this study 12](#_Toc152973575)

[Supplementary Table 2. Sample overlap between educational attainment (EA) and neuropsychiatric disorders (NPDs). 14](#_Toc152973576)

[Supplementary Table 3. Partitioned genetic correlation by 13 functional categories between educational attainment and related neuropsychiatric traits 20](#_Toc152973577)

[Supplementary Table 4. Fine-mapping 99% credible-set of sentinel SNP from cross-trait meta-analysis between educational attainment (EA) and attention deficit/hyperactivity disorder (ADHD). 28](#_Toc152973578)

[Supplementary Table 5. Fine-mapping 99% credible-set of sentinel SNP from cross-trait meta-analysis between educational attainment (EA) and anorexia nervosa (AN). 37](#_Toc152973579)

[Supplementary Table 6. Fine-mapping 99% credible-set of sentinel SNP from cross-trait meta-analysis between educational attainment (EA) and autism spectrum disorders (ASD) 47](#_Toc152973580)

[Supplementary Table 7. Fine-mapping 99% credible-set of sentinel SNP from cross-trait meta-analysis between educational attainment (EA) and Alcohol use disorders (AUD) 57](#_Toc152973581)

[Supplementary Table 8. Fine-mapping 99% credible-set of sentinel SNP from cross-trait meta-analysis between educational attainment (EA) and bipolar disorder (BIP) 70](#_Toc152973582)

[Supplementary Table 9. Fine-mapping 99% credible-set of sentinel SNP from cross-trait meta-analysis between educational attainment (EA) and cannabis use disorder (CUD) 72](#_Toc152973583)

[Supplementary Table 10. Fine-mapping 99% credible-set of sentinel SNP from cross-trait meta-analysis between educational attainment (EA) and major depressive disorder (MDD). 79](#_Toc152973584)

[Supplementary Table 11. Fine-mapping 99% credible-set of sentinel SNP from cross-trait meta-analysis between educational attainment (EA) and posttraumatic stress disorder (PTSD). 85](#_Toc152973585)

[Supplementary Table 12. Fine-mapping 99% credible-set of sentinel SNP from cross-trait meta-analysis between educational attainment (EA) and Tourette’s syndrome (TS). 91](#_Toc152973586)

[Supplementary Table 13. Results of co-localization analysis of educational attainment (EA) and each neuropsychiatric trait. 100](#_Toc152973587)

[Supplementary Table 14. Biological process of the shared gene set between EA and neuropsychiatric disorders in Gene Ontology (GO) terms 102](#_Toc152973588)

[Supplementary Table 15. Horizontal pleiotropy test and heterogeneity assessment in the bidirectional MR analysis between EA and neuropsychiatric disorders. 107](#_Toc152973589)

[Supplementary Table 16. Causal inference between EA and neuropsychiatric traits using Two-sample MR, MR-RAPS and MR-PRESSO methods 108](#_Toc152973590)

[Supplementary Table 17. Causal inference between educational attainment (EA) and neuropsychiatric traits after removing SNPs associated with potential confounding diseases or traits. 117](#_Toc152973591)

[Supplementary Table 18. No. of TWAS significant genes for educational attainment (EA) and neuropsychiatric traits, and No. of shared TWAS significant genes between EA and related neuropsychiatric traits across 48 GTEx tissues. 118](#_Toc152973592)

[Supplementary Table 19. List of instrumental variables used in our Mendelian randomization analyses. 120](#_Toc152973593)

[Supplementary Table 20. List of SNPs with genome-wide significance (*P* < 5×10^-8^) for potential confounding traits or disease searched from PhenoScanner and GWAS catalog. 133](#_Toc152973594)

[Supplementary Figure 140](#_Toc152973595)

[Supplementary Figure 1. Annotation-specific Genetic Correlations (±SE) of EA with Neuropsychiatric Traits. 140](#_Toc152973596)

[Supplementary Figure 2. The QQ plot and inflation lambda for the cross-trait GWAS. 141](#_Toc152973597)

[Supplementary Figure 3. Number of significant expression-trait associations from transcriptome-wide association study (TWAS) for EA, AD, AUD, and BIP 142](#_Toc152973598)

[Supplementary Figure 4. Number of significant expression-trait associations from transcriptome-wide association study (TWAS) for ADHD, AN, and ASD 143](#_Toc152973599)

[Supplementary Figure 5. Number of significant expression-trait associations from transcriptome-wide association study (TWAS) for CUD and MDD 144](#_Toc152973600)

# Online Methods

## Summary statistics for educational attainment and neuropsychiatric traits, quality control

In our analysis, educational attainment is measured in all main analyses as the number of years of schooling completed (*EduYears*, a continuous variable, standard deviation (SD) = 3.6 years)^1^. Information for all of the Consortia is presented in eTable1.The ethical approval and quality control procedures of each consortium have been described in previous studies, respectively. In addition, we restricted the chromosome region to autosomal chromosomes and excluded single nucleotide polymorphism (SNPs) in MHC region (chr6:25Mb-34Mb) as recommended.

## Study design

We assessed the genetic correlations of EA with 13 neuropsychiatric traits, followed by a cross phenotype association study to combine association evidence for EA with related neuropsychiatric disorders at individual loci and a bidirectional two sample Mendelian Randomization (MR) analysis to infer the causality between them. Then, we investigated the shared genes between them using TWAS, which relates genetic effect of tissue-specific gene expression with each of the aforementioned traits. We further applied tissue-specific gene enrichment analysis, functional enrichment analysis, colocalization analysis to explore possible biological pathways, molecular mechanisms and causal loci.

## Sample overlap

**Supplementary Table 2** showed the overlapped cohorts between EA and each NPDs. Sample overlap rate was calculated using the following method. We first examined whether there were overlapped cohorts between the EA and each NPDs (see Supplementary Table 2). If there are no overlapping cohorts, we considered that there is no sample overlap. If there existed overlapped cohorts, then we assumed that trait 1 and trait 2 have two overlapping cohorts (called cohort A and cohort B). Assume that the total sample size for trait 1 and trait 2 is *n*_1_ and *n*_2_, respectively. The sample size of cohort A and cohort B in trait 1 is *x*_1_ and *y*_1_, respectively. The sample size of cohort A and cohort B in trait 2 is *x*_2_ and *y*_2_, respectively. Then the overlapped samples (denoted as overlap_n) equal to “min(*x*_1_, *x*_2_) + min(*y*_1_, *y*_2_)”, which means the minimum of *x*_1_ and *x*_2_ plus minimum of *y*_1_ and *y*_2_. And the sample overlap rate equals to “overlap_n/(*n*_1_ + *n*_2_ - overlap_n)”. The results showed that there is no sample overlap between EA and AD, ADHD, ALS, AN, BIP, OCD, PTSD and TS. In contrast, a smaller sample overlap existed between EA and AUD, CUD, MDD and SCZ, with overlap rate of approximately 4.9%, 7.5%, 1.2% and 1.9%, respectively. This result showed that the sample overlap in this study was relatively low. To formally assess the risk of bias resulting from sample overlap, we performed a calculation of bias and type I error rate using the method proposed by Burgess et al. (<https://sb452.shinyapps.io/overlap>). Formal assessment revealed a minimal risk of bias from sample overlap (<0.001, regardless of overlap proportion).

## Partitioned genetic correlation

To investigate whether the whole-genome genetic correlation between EA and related neuropsychiatric traits are attributed to specific functional regions, we estimated annotation specific genetic correlations between EA and neuropsychiatric traits in 13 large annotations using partitioned LD score regression. We used 13 large annotations which include DNase I hypersensitivity sites (DHS), fetal DHS, DNaseI digital genomic foot printing (DGF) region, histone marks (H3K4me1, H3K4me3, H3K9ac, and H3K27ac), intron, Super Enhancer, transcription factor binding sites (TFBS), transcribed region, conserved region, and repressed region^2, 3^. Each annotation contains more than 600,000 overlapping SNPs between EA and each neuropsychiatric trait. For each annotation, LD scores were calculated for SNPs assigned to each annotation and then estimated genetic correlation between EA and each neuropsychiatric trait using the annotation-specific LD scores, one at a time.

## Cross trait meta-analysis

After assessing genetic correlations among all traits, we applied cross-trait GWAS meta-analysis using the R code Cross-Phenotype Association (CPASSOC) to combine the association evidence for EA with related neuropsychiatric traits, based on the criteria of both *r*_g_ > 10% and *P*_bonferroni_ < 0.05 from HDL^4^. SHet is a cross-phenotype meta-analysis method based on fixed effect model. It can be viewed as the maximum of weighted sum of trait-specific test statistics, which is closely related to a gamma distribution. It is more powerful when there is heterogeneous effect present between studies, which is common in meta-analysis of different phenotypes ^5, 6^. SHet also uses the sample size for a trait as a weight instead of variance.

We applied PLINK1.9 clumping function (parameters: --clump-p1 3.8e-9 --clump-p2 1e-5 --clump-r2 0.4 --clump-kb 500) to determine top loci that are independent of each other, i.e., variants with P value less than 1×10^-5^ have *r*^2^ more than 0.4 and less than 500 kb away from the peak will be assigned to that peak’s clump. We identified all genes falling within each clump region. A P value of 3.8×10^-9^ (5×10^-8^/13) was used as genome-wide significance level for cross-trait meta-analysis to account of 13 meta-analysis testing. SNPs with a meta-analysis P value less than 3.8×10^-9^ and trait specific P value less than 1×10^-5^ were selected for downstream analysis.

We use R package “P_lambda” to calculate the inflation lambda. The inflation lambda and the corresponding QQ plot for the cross-trait GWAS can be found in supplementary figure 5.

## Tissue-specific enrichment analysis

‘deTS’ provides a preprocessed reference panel with 47 tissues (each with ≥ 30 samples) from the GTEx (v7) expression data ^7^ and implements Fisher’s Exact Test for the enrichment analysis. The p-values were corrected for multiple hypothesis testing using the Benjamini & Hochberg correction. We selected suggestive significant loci for this analysis (***P***_TSEA_< 0.1) to ensure the robustness of TSEA results.

## Mendelian randomization

We accomplished bi-directional MR analysis using Two-sample MR package (0.5.6 version) with the aim of inferring putative causal links between EA and neuropsychiatric traits. Two sample MR leverages power from all of the top SNPs associated with exposure at a genome-wide significance level as instruments, with accounting for LD between the variants to test for causality. Three basic assumptions must be met in MR analysis: (1) the genetic instruments used must be associated with the exposure of interest, (2) the genetic instruments must not be associated with the confounders of the exposure outcome association, and (3) the genetic instruments must be conditionally independent of the outcome given the exposure ^8^. We ascertained the valid instrumental variables (IV) grounded in LD-independent SNPs derived from genome-wide significance variations for exposure factor. In practice, we extracted SNPs with *P* < 5×10^−8^ from the GWAS summary statistics for interested exposure as the genetic instruments to satisfy the first assumption, conducted a horizontal pleiotropy analysis to test the second assumption, and selected independent genetic instruments (*r*^2^ < 0.001) to avoid violating the third assumption.

For each phenotype, we initially extracted independent genome-wide significant (*P* < 5×10^−8^) SNPs from the corresponding summary statistics as instrumental variants (IVs). The complete list of instruments is summarized in ***Supplementary Tables 19***. While MR methods, such as median based, require more than two SNPs for analysis; thus, to improve statistical power, we chose a relaxed threshold of 5×10^−7^ for AN, ASD, CUD and PTSD and of 5×10^−6^ for OCD and TS, and then used PLINK version 1.9 (*r*^2^ = 0.001, window size = 10000 kb) for clumping. This relaxed threshold approach can be applied in MR studies when no or few genome-wide significant SNPs are found in GWAS data^9, 10^. However, we admit that lowering the inclusion threshold may result in weak instrumental bias in our estimations^11^. To address this, we use robust adjusted profile score MR scores (MR-RAPS) ^12^ and debiased inverse-variance weighted estimator (MR-DIVW)^13^, which both offer robust effect estimates with weak instrumental bias.

In our MR analysis, we used the TwoSampleMR R package (version 0.5.6) to undertake a harmonization procedure for integrating IVs information between exposure and outcome. We also removed SNPs that were palindromic with intermediate allele frequencies. We then applied Steiger filtering to ensure whether each IV explains more phenotypical variance in the exposure than the outcome, and removes those genetic variants that do not satisfy this criterion. The IVs with a ‘false’ Steiger direction will be excluded. To further exclude bias due to horizontal pleiotropy, for trait pairs with significant causal relationships in univariable MR analyses, we also searched the PhenoScanner and the NHGRI-EBI GWAS catalog to examine whether some of the selected IVs may have a genome-wide significant association with other diseases or traits. (***Supplementary Table 20***). We excluded these instruments to obtain more robust estimates to avoid estimation bias caused by these pleiotropic associations.

Several approaches containing simple median method, weighted median method, inverse variance-weighted (IVW) method, MR-Egger, MR-RAPS, MR-Pleiotropy Residual Sum and Outlier (MR-PRESSO), MR-DIVW method were exploited to explore the causal effect. Finally, we performed a serial of sensitivity analyses (including pleiotropy test, and heterogeneity assessment) for assessing the impact of horizontal pleiotropy and other violations of assumptions.

## Interpretation of the causal effect estimates

As neuropsychiatric disorders are binary variables, we interpreted the reverse causal estimates as the average change in EA per doubling (2-fold increase) in the prevalence of neuropsychiatric disorders, which could be obtained by multiplying the reverse causal estimate by 0.693 (log*_e_*2)^14^.

In the analyses investigating the causal effects of genetic liability of neuropsychiatric traits on EA, the estimated MR effects and 95% confidence intervals were expressed as one standard deviation (SD) increase in EA per log-odd increase in genetic liability of neuropsychiatric traits. In order to assist the interpretation of the findings, we converted the estimates to months/days (where appropriate) of education per doubling the genetic liability of neuropsychiatric traits. Specifically, we multiplied the estimates by the standard deviation (SD) of educational attainment (years of education SD = 3.6 years) ^1^ and then multiplied by log*_e_*2 to estimate the effect of per doubling of liability to neuropsychiatric traits. For example, if we observed the estimated effect size (beta) of ADHD on EA is -0.095, then the causal effect = -0.095×0.693×3.6×12 = -2.8 (12 means 12 months).

In the analyses investigating the causal effects of genetic liability to higher educational attainment on risk of neuropsychiatric traits, MR estimates and 95% confidence intervals are expressed per one SD increase in educational attainment on the odds of developing neuropsychiatric disorders.

## Transcriptome-wide association studies

In total, we performed 48 TWAS for EA and each of its genetically related neuropsychiatric traits, 1 tissue-trait pair at a time. We leveraged expression imputation from genetic data to perform a transcriptome-wide association study (TWAS) that integrate genetic and transcriptional variation with the FUSION software package based on 48 GTEx (version 7) tissue expression reference weights to identify genes with expression associated with complex traits^15^.

# Online Results

## The ClueGO log in our experiment

### All results were created with ClueGO v2.5.7 ###

Organism analyzed: Homo Sapiens [9606]

Identifier types used: [SymbolID]

Evidence codes used: [All]

#Genes in GO_BiologicalProcess-EBI-UniProt-GOA-ACAP-ARAP_08.05.2020_00h00 : 17972

#All unique genes in selected ontologies: 17972 (reference set for hypergeometric test)

#Genes from Cluster#1: unique uploaded ids 117 -> corresponding genes 115, with 2 (1.71%) missing -> 115 recognized by ClueGO.

-> To improve the % of found genes, verify gene identifiers, download new available ClueGO conversion files or add/request additional files.

#Genes with functional annotations in all selected Ontologies from Cluster#1: 80 (69.57%) -> 35 (30.43%) are not functionally annotated in any selected Ontology!

-> To improve the % of annotated genes, chose additional ontologies.

#Genes from all Clusters associated to 158 representative Terms and Pathways (after applying general selection criteria): 64 (55.65%)

#Genes from all Clusters associated to 57 representative Terms and Pathways (after fusion selection criteria): 51 (44.35%)

#Genes from all Clusters associated to 52 representative Terms and Pathways (after p-value significance selection criteria): 50 (43.48%)

KappaScore Grouping:

Iteration: 0 with 24 groups

Iteration: 1 with 27 groups

Iteration: 2 with 10 groups

Final KappaScore groups = 10

# Terms not grouped = 0

# Merge redundant groups with >50.0% overlap

Final group size after merging: 10

#GO All Terms Specific for Cluster #1: 52

Ontology used:

GO_BiologicalProcess-EBI-UniProt-GOA-ACAP-ARAP_08.05.2020_00h00

Evidence codes used:

All

Identifiers used:

SymbolID

List of missing Genes:

Cluster #1

Gene IDs not found (no annotation or wrong ids) in Cluster#1:

LOC102723483

RP11-6N13.1

Statistical Test Used = Enrichment/Depletion (Two-sided hypergeometric test)

Use pvalue cutoff = true

pvalue cutoff = 0.05000000074505806

Correction Method Used = Benjamini-Hochberg

Min GO Level = 3

Max GO Level = 8

Cluster #1

Sample File Name = File selection: ManuallyAddedOrModi

## Shared genetics between EA and neuropsychiatric disorders from TWAS

The TWAS-significant genes that were shared between EA and ADHD, AN, ASD, AUD, CUD, AD, BIP, or MDD, respectively, most of which were observed at tissues from the nervous system (9 genes at brain cerebellum for AD, 25 genes at nerve tibial for AN; 20 genes at brain cerebellum for ASD; 24 genes at brain cerebellum for AUD; 4 genes at brain hypothalamus for CUD; 4 genes at nerve tibial for BIP, 4 genes at brain cortex for MDD), cardiovascular system (12 genes at heart left ventricle for ADHD, 25 genes at artery tibial for AN, 4 genes at artery aorta for CUD), endocrine system (12 genes at adipose visceral omentum for ADHD, 15 genes at testis for ASD, 23 genes at testis for AUD, 4 genes at adrenal gland for CUD, 11 genes at breast mammary tissue for AD), digestive system (24 genes at esophagus mucosa for AN, 6 genes at liver for BIP), and musculoskeletal system (9 genes at muscle skeletal for ADHD).

The strongest association between EA and ADHD was observed at 1p34.1(mapped gene: *ST3GAL3*) covers a gene-rich 250-kb region of strong LD. Most SNPs in the credible set are strongly associated with expression of *ST3GAL3* in whole blood^16^. *ST3GAL3* encodes a sialyltransferase responsible for the terminal sialylation of brain gangliosides and glycoproteins, which constitute a major part of the surface glycan coat of neurons and glia and acts as an interface for cellular interactions^17^. Interestingly, missense mutations of *ST3GAL3* may impair the development of higher cognitive functions^18^ and are associated with severe infantile epilepsy^19^. The most significant shared signals between EA and MDD was verified at 1p31.1 (mapped gene: *NEGR1*). *NEGR1* (neuronal growth regulator 1) influences axon extension and synaptic plasticity in cortex, hypothalamus, and hippocampus, and modulates synapse formation in hippocampus via regulation of neurite outgrowth^20-22^. Of note, a 1p31.1 deletion including only part of *NEGR1* has been reported in two siblings who are only modestly underweight but have neuropsychiatric and behavioral phenotypes^23^. Shared TWAS associations provided potential biological shared pathways, mechanisms, or potential therapeutic targets to follow-up in the future.

Of the shared gene-tissue pairs between EA and neuropsychiatric, 144 gene-tissue pairs were overlapped between EA and ADHD. Notably, *PTPRF* (located at 1p34.2) expressed in multiple tissues including brain, nerve, artery, esophagus, heart, liver, pancreas tissue and so on. Consistent with the report by Shadrin et al.^24^, *PTPRF* was validated as a shared genetic component in EA and ADHD from our TWAS results. The protein encoded by *PTPRF* is a member of the protein tyrosine phosphatase (PTP) family, which regulates a variety of cellular processes, including cell growth, differentiation, mitotic cycle, and oncogenic transformation. Murine studies showed that *PTPRF* promotes neurogenesis in the hippocampus, a brain region linked to memory^25^. Furthermore, we identified 433 gene-tissue pairs were shared by EA and AN. Some genes expressed among multiple tissues like *RBM6* (located at 3p21.31) and *DAG1* (located at 3p21.31). *RBM6* encodes RNA-binding protein 6 and as a tumor suppressor repressed the growth and progression in laryngocarcinoma^26^. *DAG1* encodes dystroglycan, a receptor that binds extracellular matrix proteins. *DAG1* falls within locus 1 (47.5–51.3 Mb). The pathway of positive regulation of embryonic development points to a potential role of developmental processes in the etiology of this complex phenotype (although this is currently speculative)^27^.

In addition, 396 and 649 gene-tissue pairs were common for EA with ASD and AUD, respectively. Interestingly, *DND1P1* (located at 17q21.31) and *KANSL1-AS1*(located at 17q21.31) were the highest co-expressed among 48 tissues of both trait pairs. To our knowledge, the pseudogene *DND1P1* has been reported to be associated with AUD^28^, but its function is not yet clear and warrants further study. *KANSL1-AS1* has been described in the context of Alzheimer’s and Parkinson’s diseases^29^ and showed a significant increased expression in osteoarthritis^30^. Additionally, we verified 48 gene-tissue pairs shared between EA and CUD. Among these shared genes, *NAT6* (located at 3p21.31) expressed in nine tissues. *NAT6* encodes a member of the N-acetyltransferase family. N-acetyltransferases modify proteins by transferring acetyl groups from acetyl-CoA to the N-termini of protein substrates. The encoded protein is a cytoplasmic N-acetyltransferase with a substrate specificity for N-termini that are enriched for acidic residues^31^. This gene was significantly related to cannabis use disorder through genetically regulated gene expression^32^.

Moreover, we identified 14 gene-tissue pairs shared between EA and MDD. The most expressed gene in the 48 tissues was *NEGR1* (1p31.1). The functions of *NEGR1* have been described in detail above. Ultimately, we found only three and five gene-tissue pairs between EA and AD or BIP, respectively, and no multi-tissue expressed genes were found either.

# References

1. Okbay A, Beauchamp JP, Fontana MA, et al. Genome-wide association study identifies 74 loci associated with educational attainment. *Nature* 2016; **533**: 539-42.

2. Finucane HK, Bulik-Sullivan B, Gusev A, et al. Partitioning heritability by functional annotation using genome-wide association summary statistics. *Nature genetics* 2015; **47**: 1228-35.

3. Bulik-Sullivan B, Finucane HK, Anttila V, et al. An atlas of genetic correlations across human diseases and traits. *Nature genetics* 2015; **47**: 1236-41.

4. Ning Z, Pawitan Y, Shen X. High-definition likelihood inference of genetic correlations across human complex traits. *Nat Genet* 2020; **52**: 859-64.

5. Watson HJ, Yilmaz Z, Thornton LM, et al. Genome-wide association study identifies eight risk loci and implicates metabo-psychiatric origins for anorexia nervosa. *Nature genetics* 2019; **51**: 1207-14.

6. Zhu Z, Anttila V, Smoller JW, Lee PH. Statistical power and utility of meta-analysis methods for cross-phenotype genome-wide association studies. *PLoS One* 2018; **13**: e0193256.

7. Battle A, Brown CD, Engelhardt BE, Montgomery SB. Genetic effects on gene expression across human tissues. *Nature* 2017; **550**: 204-13.

8. Benn M, Nordestgaard BG. From genome-wide association studies to Mendelian randomization: novel opportunities for understanding cardiovascular disease causality, pathogenesis, prevention, and treatment. *Cardiovasc Res* 2018; **114**: 1192-208.

9. Orri M, Séguin JR, Castellanos-Ryan N, et al. A genetically informed study on the association of cannabis, alcohol, and tobacco smoking with suicide attempt. *Molecular psychiatry* 2021; **26**: 5061-70.

10. Choi KW, Chen CY, Stein MB, et al. Assessment of Bidirectional Relationships Between Physical Activity and Depression Among Adults: A 2-Sample Mendelian Randomization Study. *JAMA psychiatry* 2019; **76**: 399-408.

11. Pierce BL, Burgess S. Efficient design for Mendelian randomization studies: subsample and 2-sample instrumental variable estimators. *American journal of epidemiology* 2013; **178**: 1177-84.

12. Zhao Q, Wang J, Hemani G, Bowden J, Small DS. Statistical inference in two-sample summary-data Mendelian randomization using robust adjusted profile score. 2020; **48 %J The Annals of Statistics**: 1742-69, 28.

13. Ye T, Shao J, Kang H. Debiased inverse-variance weighted estimator in two-sample summary-data Mendelian randomization. 2021; **49 %J The Annals of Statistics**: 2079-100, 22.

14. Burgess S, Labrecque JA. Mendelian randomization with a binary exposure variable: interpretation and presentation of causal estimates. *Eur J Epidemiol* 2018; **33**: 947-52.

15. Gusev A, Ko A, Shi H, et al. Integrative approaches for large-scale transcriptome-wide association studies. *Nature genetics* 2016; **48**: 245-52.

16. Zhernakova DV, Deelen P, Vermaat M, et al. Identification of context-dependent expression quantitative trait loci in whole blood. *Nat Genet* 2017; **49**: 139-45.

17. Yoo SW, Motari MG, Susuki K, et al. Sialylation regulates brain structure and function. *Faseb j* 2015; **29**: 3040-53.

18. Hu H, Eggers K, Chen W, et al. ST3GAL3 mutations impair the development of higher cognitive functions. *American journal of human genetics* 2011; **89**: 407-14.

19. Edvardson S, Baumann AM, Mühlenhoff M, et al. West syndrome caused by ST3Gal-III deficiency. *Epilepsia* 2013; **54**: e24-7.

20. Sanz R, Ferraro GB, Fournier AE. IgLON cell adhesion molecules are shed from the cell surface of cortical neurons to promote neuronal growth. *J Biol Chem* 2015; **290**: 4330-42.

21. Schäfer M, Bräuer AU, Savaskan NE, Rathjen FG, Brümmendorf T. Neurotractin/kilon promotes neurite outgrowth and is expressed on reactive astrocytes after entorhinal cortex lesion. *Mol Cell Neurosci* 2005; **29**: 580-90.

22. Hashimoto T, Yamada M, Maekawa S, Nakashima T, Miyata S. IgLON cell adhesion molecule Kilon is a crucial modulator for synapse number in hippocampal neurons. *Brain Res* 2008; **1224**: 1-11.

23. Genovese A, Cox DM, Butler MG. Partial Deletion of Chromosome 1p31.1 Including only the Neuronal Growth Regulator 1 Gene in Two Siblings. *J Pediatr Genet* 2015; **4**: 23-8.

24. Shadrin AA, Smeland OB, Zayats T, et al. Novel Loci Associated With Attention-Deficit/Hyperactivity Disorder Are Revealed by Leveraging Polygenic Overlap With Educational Attainment. *Journal of the American Academy of Child and Adolescent Psychiatry* 2018; **57**: 86-95.

25. Bernabeu R, Yang T, Xie Y, Mehta B, Ma SY, Longo FM. Downregulation of the LAR protein tyrosine phosphatase receptor is associated with increased dentate gyrus neurogenesis and an increased number of granule cell layer neurons. *Molecular and cellular neurosciences* 2006; **31**: 723-38.

26. Wang Q, Wang F, Zhong W, et al. RNA-binding protein RBM6 as a tumor suppressor gene represses the growth and progression in laryngocarcinoma. *Gene* 2019; **697**: 26-34.

27. Bello V, Moreau N, Sirour C, Hidalgo M, Buisson N, Darribère T. The dystroglycan: nestled in an adhesome during embryonic development. *Dev Biol* 2015; **401**: 132-42.

28. Sanchez-Roige S, Palmer AA, Fontanillas P, et al. Genome-Wide Association Study Meta-Analysis of the Alcohol Use Disorders Identification Test (AUDIT) in Two Population-Based Cohorts. *Am J Psychiatry* 2019; **176**: 107-18.

29. Ferrari R, Wang Y, Vandrovcova J, et al. Genetic architecture of sporadic frontotemporal dementia and overlap with Alzheimer's and Parkinson's diseases. *Journal of neurology, neurosurgery, and psychiatry* 2017; **88**: 152-64.

30. Rice SJ, Cheung K, Reynard LN, Loughlin J. Discovery and analysis of methylation quantitative trait loci (mQTLs) mapping to novel osteoarthritis genetic risk signals. *Osteoarthritis and cartilage* 2019; **27**: 1545-56.

31. Wiame E, Tahay G, Tyteca D, et al. NAT6 acetylates the N-terminus of different forms of actin. *The FEBS journal* 2018; **285**: 3299-316.

32. Johnson EC, Demontis D, Thorgeirsson TE, et al. A large-scale genome-wide association study meta-analysis of cannabis use disorder. *Lancet Psychiatry* 2020; **7**: 1032-45.

# Supplementary Table

## Supplementary Table 1. Information of summary statistics used in this study

| Trait | Consortium | Sample size | Reference |
| --- | --- | --- | --- |
| ADHD | PGC | 55374(20183 cases,  35191 controls) | Demontis D, Walters RK, Martin J, Mattheisen M, Als TD, Agerbo E et al. Discovery of the first genome-wide significant risk loci for attention deficit/hyperactivity disorder. Nature genetics 2019; 51(1): 63-75. |
| ALS | Project MinE Website | 36052(12577 cases,  23475controls) | van Rheenen W, Shatunov A, Dekker AM, McLaughlin RL, Diekstra FP, Pulit SL et al. Genome-wide association analyses identify new risk variants and the genetic architecture of amyotrophic lateral sclerosis. Nature genetics 2016; 48(9): 1043-1048. |
| AD | CTGlab | 455258(71880 cases,  383378 controls) | Jansen IE, Savage JE, Watanabe K, Bryois J, Williams DM, Steinberg S et al. Genome-wide meta-analysis identifies new loci and functional pathways influencing Alzheimer's disease risk. Nature genetics 2019; 51(3): 404-413. |
| ASD | PGC | 46351(18382 cases,  27969 controls) | Grove J, Ripke S, Als TD, Mattheisen M, Walters RK, Won H et al. Identification of common genetic risk variants for autism spectrum disorder. Nature genetics 2019; 51(3): 431-444. |
| AUD | PGC | 121604(9130 cases,  112474 controls) | Sanchez-Roige S, Palmer AA, Fontanillas P, Elson SL, Adams MJ, Howard DM et al. Genome-Wide Association Study Meta-Analysis of the Alcohol Use Disorders Identification Test (AUDIT) in Two Population-Based Cohorts. The American journal of psychiatry 2019; 176(2): 107-118. |
| BIP | PGC | 51710(20352 cases,  31358 controls) | Stahl EA, Breen G, Forstner AJ, McQuillin A, Ripke S, Trubetskoy V et al. Genome-wide association study identifies 30 loci associated with bipolar disorder. Nature genetics 2019; 51(5): 793-803. |
| CUD | PGC | 358534(14808 cases,  343726 controls) | Demontis D, Rajagopal VM, Thorgeirsson TE, Als TD, Grove J, Leppälä K et al. Genome-wide association study implicates CHRNA2 in cannabis use disorder. Nature neuroscience 2019; 22(7): 1066-1074. |
| AN | PGC | 72517(16992 cases,  55525 controls) | Watson HJ, Yilmaz Z, Thornton LM, Hübel C, Coleman JRI, Gaspar HA et al. Genome-wide association study identifies eight risk loci and implicates metabo-neuropsychiatric origins for anorexia nervosa. Nature genetics 2019; 51(8): 1207-1214. |
| MDD | PGC | 143265(45591 cases,  97674 controls) | Wray NR, Ripke S, Mattheisen M, Trzaskowski M, Byrne EM, Abdellaoui A et al. Genome-wide association analyses identify 44 risk variants and refine the genetic architecture of major depression. Nature genetics 2018; 50(5): 668-681 |
| OCD | PGC | 9725(2688 cases,  7037 controls) | Revealing the complex genetic architecture of obsessive-compulsive disorder using meta-analysis. Molecular psychiatry 2018; 23(5): 1181-1188. |
| PTSD | PGC | 174659(23212 cases,  151447 controls) | Nievergelt CM, Maihofer AX, Klengel T, Atkinson EG, Chen CY, Choi KW et al. International meta-analysis of PTSD genome-wide association studies identifies sex- and ancestry-specific genetic risk loci. Nature communications 2019; 10(1): 4558. |
| SCZ | PGC | 77096(33640 cases,  43456 controls) | Biological insights from 108 schizophrenia-associated genetic loci. Nature 2014; 511(7510): 421-427. |
| TS | PGC | 14307(4819 cases,  9488 controls) | Yu D, Sul JH, Tsetsos F, Nawaz MS, Huang AY, Zelaya I et al. Interrogating the Genetic Determinants of Tourette's Syndrome and Other Tic Disorders Through Genome-Wide Association Studies. The American journal of psychiatry 2019; 176(3): 217-227 |

*Note*: ADHD: Attention deficit/hyperactivity disorder; ALS: Amyotrophic lateral sclerosis; AD: Alzheimer’s dementia; AN: Anorexia nervosa; ASD: Autism spectrum disorders; AUD: Alcohol use disorders; BIP: Bipolar disorder; CUD: Cannabis use disorder; MDD: Major depressive disorder; OCD: Obsessive compulsive disorder;

PTSD: Posttraumatic stress disorder; SCZ: Schizophrenia; TS: Tourette’s syndrome

## Supplementary Table 2. Sample overlap between educational attainment (EA) and neuropsychiatric disorders (NPDs).

**Supplementary Table 2a. No sample overlaps**

| Phenotype | Educational Attainment  (N=293723) | AD (N=455258) | ADHD  (N=55374) | ALS  (N=36052) | AN  (N=72517) | ASD  (N=46351) | BIP  (N=51710) | OCD  (N=9725) | PTSD  (N=174659) | TS  (N=14307) |
| --- | --- | --- | --- | --- | --- | --- | --- | --- | --- | --- |
| Cohorts | ACPRC | ADSP | Barcelona-Spain | NL1 | CHOP/PFCG | iPSYCH | BOMA-Australia | IOCDF-GC | ADNH | TAAICG-GGRI |
|  | AGES | IGAP | Bergen-Norway | BE1 | GCAN/WTCCC-3 | DPCRR | BOMA-Germany I | OCGAS | BAKE | TAAICG |
|  | ALSPAC | DemGene | Cardiff-UK | NL2 | ANGI | DNSB | BOMA-Germany II | | BETR | TIC Genetics |
|  | ASPS | STSA | CHOP | SW1 | ANGI-ANZ | ACE | BOMA-Germany III | | BOBA | CNP |
|  | BASE-II | TwinGene | Germany | NL3 | ANGI-DK | AGP | BOMA-Poland \| | | BRY2 | GSP |
|  | CoLaus |  | IMAGE-I | FR1 | ANGI-SE | AGRE | BOMA-Romania | | BRYA | NINDS |
|  | COPSAC2000 | | IMAGE-II | UK1 | ANGI-US | NIMH Repository | BOMA-Spain | | COGA | AJ_ctl |
|  | CROATIA-Korčula | | iPSYCH | US1 | UK Biobank | MONBOS | Bulgarian trios | | COGB | GPC |
|  | deCODE |  | PUWMa | IR1 |  | SSC | Cardiff and Worcester, UK  (ICCBD-BDRN) | | COM1 | iControl |
|  | DHS |  | PUWMa (strict) | IR2 |  |  | Edinburgh, UK | | DAIP | SAGE |
|  | DIL |  | Toronto-Canadian | UK2 |  |  | France |  | DAMI | Dutch_ctl |
|  | EGCUT1 |  | YalePenn | US2 |  |  | Ireland |  | DCSR |  |
|  | EGCUT2 |  |  | US3 |  |  | Los Angeles, USA (ICCBD-GPC) | | DEFE |  |
|  | EGCUT3 |  |  | IT1 |  |  | Mayo Clinic, USA(MCBB) | | DNHS |  |
|  | ERF |  |  | IT2 |  |  | Michigan, USA  (Pritzker and NIMH) | | EACR |  |
|  | FamHS |  |  | UK3 |  |  | Netherlands | | EGHS |  |
|  | FINRISK |  |  | UK4 |  |  | Norway (TOP7) | | FEEN |  |
|  | FTC |  |  | US4 |  |  | Norway (TOP8) | | FSCD |  |
|  | GOYA |  |  | US5 |  |  | Nova Scotia, Canada;  I2B2 controls (SADS-L) | | FTCB |  |
|  | GRAPHIC | |  | FIN1 |  |  | Sweden (ICCBD) | | GMRF |  |
|  | GS |  |  | FIN2 |  |  | Sweden (ICCBD) | | GRAC |  |
|  | H2000 Cases | |  | FIN3 |  |  | UCL (University College London),  London, UK | | GSDC |  |
|  | H2000 Controls | |  | IT3 |  |  | UK trios |  | GTPC |  |
|  | HBCS |  |  | FR2 |  |  | Umeå, Sweden(MINI, DIGS,  FIGS, SCAN) | | GUTS |  |
|  | HCS |  |  | GER1 |  |  | USA (FAT2; FaST, BiGS, TGEN) | | INTR |  |
|  | HNRS (CorexB) | |  | NL4 |  |  | USA (GAIN) | | KMCT |  |
|  | HNRS (Oexpr) | |  | GER2 |  |  | USA (Janssen), SAGE controls | | KSUD |  |
|  | HNRS3 |  |  | IT4 |  |  | USA (Pfizer) | | MIRE |  |
|  | HRS |  |  | PU1 |  |  | USA (STEP1) | | MRSC |  |
|  | Hypergenes | |  | SP1 |  |  | USA (STEP2) | | NCMH |  |
|  | INGI-CARL | |  | SWISS1 |  |  | WTCCC |  | NCPT |  |
|  | INGI-FVG | |  | BE2 |  |  |  |  | NHRV |  |
|  | KORA-S3 | |  | FIN4 |  |  |  |  | NHS2 |  |
|  | KORA-S4 | |  | IR3 |  |  |  |  | NHSY |  |
|  | LBC1921 |  |  | SW2 |  |  |  |  | NIUT |  |
|  | LBC1936 |  |  | US6 |  |  |  |  | NSS1 |  |
|  | LifeLines |  |  | GER3 |  |  |  |  | NSS2 |  |
|  | MCTFR |  |  | FR3 |  |  |  |  | ONGA |  |
|  | MGS |  |  | US7 |  |  |  |  | PORT |  |
|  | MoBa |  |  | UK5 |  |  |  |  | PPDS |  |
|  | NBS |  |  | NL5 |  |  |  |  | PRIS |  |
|  | NESDA |  |  |  |  |  |  |  | PROM |  |
|  | NFBC66 |  |  |  |  |  |  |  | QIMR |  |
|  | NTR |  |  |  |  |  |  |  | RING |  |
|  | OGP |  |  |  |  |  |  |  | SAFR |  |
|  | OGP-Talana | |  |  |  |  |  |  | SATU |  |
|  | ORCADES | |  |  |  |  |  |  | SEEP |  |
|  | PREVEND | |  |  |  |  |  |  | SHRS |  |
|  | QIMR |  |  |  |  |  |  |  | STRO |  |
|  | RS-I |  |  |  |  |  |  |  | TEIC |  |
|  | RS-II |  |  |  |  |  |  |  | TRAC |  |
|  | RS-III |  |  |  |  |  |  |  | UKBB |  |
|  | Rush-MAP | |  |  |  |  |  |  | VETS |  |
|  | Rush-ROS | |  |  |  |  |  |  | VRIS |  |
|  | SardiNIA |  |  |  |  |  |  |  | WACH |  |
|  | SHIP |  |  |  |  |  |  |  | WANG |  |
|  | SHIP-TREND | |  |  |  |  |  |  | YEHU |  |
|  | STR – Salty | |  |  |  |  |  |  |  |  |
|  | STR – Twingene | |  |  |  |  |  |  |  |  |
|  | THISEAS | |  |  |  |  |  |  |  |  |
|  | TwinsUK |  |  |  |  |  |  |  |  |  |
|  | WTCCC58C | |  |  |  |  |  |  |  |  |
|  | YFS |  |  |  |  |  |  |  |  |  |
|  | 23andMe |  |  |  |  |  |  |  |  |  |

**Supplementary Table 2b. Partial sample overlaps**

| Phenotypes | Educational Attainment (N=293723) | AUD (N=121604) | CUD (N=358534) | MDD (N=143265) | SCZ (N=77096) |
| --- | --- | --- | --- | --- | --- |
| Cohorts | ACPRC | UK Biobank | bigCOGA | BOMA | Umea.Sweden |
|  | AGES | 23andMe [N=20,328] | BLTS | CoFaMS | Umea,Sweden |
|  | ALSPAC |  | CADD | DGN | TOP |
|  | ASPS |  | CATS | Edinburgh | Edinburgh.UK |
|  | BASE-II |  | CEDAR | GenRED | Danish Newborn Screening Biobank |
|  | CoLaus |  | CHDS | GenRED | Seven countries(PEIC,WTCCC2) |
|  | COPSAC2000 |  | COGEND-Nico | GSK/MPIP | Spain(PEIC,WTCCC2) |
|  | CROATIA-Korčula |  | COGEND-SAGE | i2b2-TRD | NewYork.US&lsrael, Mount Sinai |
|  | deCODE [N=46758] |  | deCODE [N=286429] | Janssen | Ireland, International Schizophrenia Consortium |
|  | DHS |  | FSCD | MARS | Ireland(WTCCC2) |
|  | DIL |  | GEDI-GSMS | NESDA/NTR [N=3096] | Germany(GRAS) |
|  | EGCUT1 [N=5597] |  | IASPSAD | NEWMEDS-GENPOD | Estonia(EGCUT) [N=1386] |
|  | EGCUT2 [N=1328] |  | iPSYCH | Pfizer | J&J,Roche cases, EGCUTcontrols [N=5779] |
|  | EGCUT3 [N=2047] |  | MCTFR [N=2074] | PsyCoLaus | US, Australia(MGS) |
|  | ERF |  | OZ-ALC | QIMR | London,UK |
|  | FamHS |  | VTSABD | RADIANT-DEN | Sweden(Hubin) |
|  | FINRISK |  | VTSABD | RADIANT-GER | Bulgaria |
|  | FTC |  | YalePenn1 | RADIANT-IRISH | Canada(Toronto)-US(Lilly)-US(MIGen) |
|  | GOYA |  | YalePenn2 | RADIANT-UK | Israel, Ashkenazi Jewish repository |
|  | GRAPHIC |  |  | RADIANT-US | Six countries,WTCCCcontrols |
|  | GS |  |  | Roche | NewYork,US |
|  | H2000 Cases |  |  | Rotterdam | Australia(ASRB) |
|  | H2000 Controls |  |  | SHIP [N=1453] | Cardiff.UK |
|  | HBCS |  |  | SHIP-TREND [N=647] | UK(CLOZUK) |
|  | HCS |  |  | STAR*D | Netherlands |
|  | HNRS (CorexB) |  |  | TwinGene | Finland |
|  | HNRS (Oexpr) |  |  |  | Finnish |
|  | HNRS3 |  |  |  | Portugal |
|  | HRS |  |  |  | Boston,US(CIDAR) |
|  | Hypergenes |  |  |  | Pfizer |
|  | INGI-CARL |  |  |  | Bonn/Mannheim,Gemany(MooDS, PopGen) |
|  | INGI-FVG |  |  |  | Munich,Germany |
|  | KORA-S3 |  |  |  | Aberdeen,Uk |
|  | KORA-S4 |  |  |  | US(CATIE) |
|  | LBC1921 |  |  |  | Swedish Schizophrenia Study(sw1) |
|  | LBC1936 |  |  |  | Swedish Schizophrenia Study(sw234) |
|  | LifeLines |  |  |  | Swedish Schizophrenia Study(sw5) |
|  | MCTFR [N=3819] |  |  |  | Swedish Schizophrenia Study(sw6) |
|  | MGS |  |  |  | Cardiff,UK(CoguK) |
|  | MoBa |  |  |  | NIMH CBDB |
|  | NBS |  |  |  | Denmark |
|  | NESDA |  |  |  |  |
|  | NFBC66 |  |  |  |  |
|  | NTR [N=5246] |  |  |  |  |
|  | OGP |  |  |  |  |
|  | OGP-Talana |  |  |  |  |
|  | ORCADES |  |  |  |  |
|  | PREVEND |  |  |  |  |
|  | QIMR |  |  |  |  |
|  | RS-I |  |  |  |  |
|  | RS-II |  |  |  |  |
|  | RS-III |  |  |  |  |
|  | Rush-MAP |  |  |  |  |
|  | Rush-ROS |  |  |  |  |
|  | SardiNIA |  |  |  |  |
|  | SHIP [N=3556] |  |  |  |  |
|  | SHIP-TREND [N=901] |  |  |  |  |
|  | STR – Salty |  |  |  |  |
|  | STR – Twingene |  |  |  |  |
|  | THISEAS |  |  |  |  |
|  | TwinsUK |  |  |  |  |
|  | WTCCC58C |  |  |  |  |
|  | YFS |  |  |  |  |
|  | 23andMe [N=76155] |  |  |  |  |

Note: The yellow background indicates the overlapping cohorts of EA and AUD and their corresponding cohort sample sizes. The blue background indicates the overlapping cohorts of EA and CUD and their corresponding cohort sample sizes. The orange background indicates the overlapping cohorts of EA and MDD and their corresponding cohort sample sizes. The green background indicates the overlapping cohorts of EA and SCZ and their corresponding cohort sample sizes. ADHD: Attention deficit/hyperactivity disorder; ALS: Amyotrophic lateral sclerosis; AD: Alzheimer’s dementia; AN: Anorexia nervosa; ASD: Autism spectrum disorders; AUD: Alcohol use disorders; BIP: Bipolar disorder; CUD: Cannabis use disorder; MDD: Major depressive disorder; OCD: Obsessive compulsive disorder;

PTSD: Posttraumatic stress disorder; SCZ: Schizophrenia; TS: Tourette’s syndrome.

## Supplementary Table 3. Partitioned genetic correlation by 13 functional categories between educational attainment and related neuropsychiatric traits

| **Functional category** | **Trait 1** | **Trait 2** | **r_g_** | **se** | **z statistics** | **P** |
| --- | --- | --- | --- | --- | --- | --- |
| Conserved | EA | ADHD | -0.48 | 0.035 | -13.899 | 6.39E-44 |
|  | EA | ALS | -0.25 | 0.146 | -1.738 | 8.22E-02 |
|  | EA | AD | -0.31 | 0.098 | -3.171 | 1.50E-03 |
|  | EA | AN | 0.23 | 0.040 | 5.780 | 7.45E-09 |
|  | EA | ASD | 0.22 | 0.041 | 5.270 | 1.37E-07 |
|  | EA | AUD | 0.20 | 0.039 | 4.983 | 6.26E-07 |
|  | EA | BIP | 0.20 | 0.038 | 5.290 | 1.23E-07 |
|  | EA | CUD | -0.37 | 0.039 | -9.427 | 4.20E-21 |
|  | EA | MDD | -0.20 | 0.046 | -4.323 | 1.54E-05 |
|  | EA | OCD | 0.24 | 0.068 | 3.619 | 3.00E-04 |
|  | EA | PTSD | -0.23 | 0.063 | -3.657 | 3.00E-04 |
|  | EA | TS | -0.11 | 0.057 | -1.939 | 5.25E-02 |
| DGF | EA | ADHD | -0.60 | 0.049 | -12.231 | 2.12E-34 |
|  | EA | ALS | -0.09 | 0.075 | -1.143 | 2.53E-01 |
|  | EA | AD | -0.14 | 0.045 | -3.039 | 2.40E-03 |
|  | EA | AN | 0.23 | 0.058 | 4.038 | 5.40E-05 |
|  | EA | ASD | 0.16 | 0.054 | 2.932 | 3.40E-03 |
|  | EA | AUD | 0.20 | 0.052 | 3.899 | 9.66E-05 |
|  | EA | BIP | 0.18 | 0.041 | 4.424 | 9.71E-06 |
|  | EA | CUD | -0.41 | 0.054 | -7.589 | 3.23E-14 |
|  | EA | MDD | -0.25 | 0.063 | -3.920 | 8.87E-05 |
|  | EA | OCD | 0.31 | 0.091 | 3.372 | 7.00E-04 |
|  | EA | PTSD | -0.35 | 0.115 | -3.050 | 2.30E-03 |
|  | EA | TS | -0.11 | 0.071 | -1.591 | 1.12E-01 |
| DHS | EA | ADHD | -0.59 | 0.042 | -14.008 | 1.39E-44 |
|  | EA | ALS | -0.12 | 0.075 | -1.533 | 1.25E-01 |
|  | EA | AD | -0.20 | 0.061 | -3.252 | 1.10E-03 |
|  | EA | AN | 0.24 | 0.047 | 5.103 | 3.34E-07 |
|  | EA | ASD | 0.19 | 0.047 | 4.106 | 4.03E-05 |
|  | EA | AUD | 0.19 | 0.044 | 4.281 | 1.86E-05 |
|  | EA | BIP | 0.20 | 0.036 | 5.656 | 1.55E-08 |
|  | EA | CUD | -0.40 | 0.043 | -9.297 | 1.44E-20 |
|  | EA | MDD | -0.24 | 0.051 | -4.643 | 3.43E-06 |
|  | EA | OCD | 0.29 | 0.077 | 3.823 | 1.00E-04 |
|  | EA | PTSD | -0.29 | 0.083 | -3.510 | 4.00E-04 |
|  | EA | TS | -0.14 | 0.059 | -2.391 | 1.68E-02 |
| Fetal DHS | EA | ADHD | -0.60 | 0.047 | -12.713 | 5.00E-37 |
|  | EA | ALS | -0.09 | 0.065 | -1.428 | 1.53E-01 |
|  | EA | AD | -0.12 | 0.048 | -2.569 | 1.02E-02 |
|  | EA | AN | 0.26 | 0.049 | 5.321 | 1.03E-07 |
|  | EA | ASD | 0.18 | 0.051 | 3.653 | 3.00E-04 |
|  | EA | AUD | 0.19 | 0.046 | 4.196 | 2.72E-05 |
|  | EA | BIP | 0.20 | 0.037 | 5.288 | 1.24E-07 |
|  | EA | CUD | -0.42 | 0.046 | -9.222 | 2.91E-20 |
|  | EA | MDD | -0.23 | 0.056 | -4.072 | 4.66E-05 |
|  | EA | OCD | 0.30 | 0.079 | 3.833 | 1.00E-04 |
|  | EA | PTSD | -0.29 | 0.105 | -2.723 | 6.50E-03 |
|  | EA | TS | -0.11 | 0.061 | -1.807 | 7.08E-02 |
| H3K27ac | EA | ADHD | -0.67 | 0.084 | -7.959 | 1.74E-15 |
|  | EA | ALS | -0.08 | 0.078 | -1.018 | 3.09E-01 |
|  | EA | AD | -0.13 | 0.051 | -2.603 | 9.20E-03 |
|  | EA | AN | 0.24 | 0.081 | 2.996 | 2.70E-03 |
|  | EA | ASD | 0.14 | 0.076 | 1.795 | 7.26E-02 |
|  | EA | AUD | 0.16 | 0.072 | 2.214 | 2.68E-02 |
|  | EA | BIP | 0.19 | 0.053 | 3.609 | 3.00E-04 |
|  | EA | CUD | -0.41 | 0.075 | -5.535 | 3.11E-08 |
|  | EA | MDD | -0.22 | 0.094 | -2.378 | 1.74E-02 |
|  | EA | OCD | 0.35 | 0.126 | 2.791 | 5.30E-03 |
|  | EA | PTSD | -0.44 | 0.336 | -1.310 | 1.90E-01 |
|  | EA | TS | -0.09 | 0.097 | -0.945 | 3.45E-01 |
| H3K4me1 | EA | ADHD | -0.64 | 0.050 | -12.819 | 1.29E-37 |
|  | EA | ALS | -0.13 | 0.069 | -1.930 | 5.36E-02 |
|  | EA | AD | -0.15 | 0.046 | -3.310 | 9.00E-04 |
|  | EA | AN | 0.24 | 0.055 | 4.390 | 1.13E-05 |
|  | EA | ASD | 0.16 | 0.050 | 3.261 | 1.10E-03 |
|  | EA | AUD | 0.19 | 0.048 | 3.949 | 7.86E-05 |
|  | EA | BIP | 0.20 | 0.038 | 5.158 | 2.49E-07 |
|  | EA | CUD | -0.42 | 0.049 | -8.618 | 6.82E-18 |
|  | EA | MDD | -0.21 | 0.061 | -3.382 | 7.00E-04 |
|  | EA | OCD | 0.36 | 0.096 | 3.755 | 2.00E-04 |
|  | EA | PTSD | -0.33 | 0.111 | -2.935 | 3.30E-03 |
|  | EA | TS | -0.09 | 0.064 | -1.457 | 1.45E-01 |
| H3K4me3 | EA | ADHD | -0.70 | 0.069 | -10.235 | 1.38E-24 |
|  | EA | ALS | -0.07 | 0.069 | -1.004 | 3.16E-01 |
|  | EA | AD | -0.10 | 0.044 | -2.342 | 1.92E-02 |
|  | EA | AN | 0.22 | 0.071 | 3.030 | 2.40E-03 |
|  | EA | ASD | 0.15 | 0.069 | 2.232 | 2.56E-02 |
|  | EA | AUD | 0.22 | 0.060 | 3.665 | 2.00E-04 |
|  | EA | BIP | 0.18 | 0.047 | 3.829 | 1.00E-04 |
|  | EA | CUD | -0.43 | 0.066 | -6.465 | 1.01E-10 |
|  | EA | MDD | -0.24 | 0.086 | -2.842 | 4.50E-03 |
|  | EA | OCD | 0.37 | 0.114 | 3.254 | 1.10E-03 |
|  | EA | PTSD | -0.33 | 0.169 | -1.959 | 5.01E-02 |
|  | EA | TS | -0.15 | 0.087 | -1.693 | 9.05E-02 |
| H3K9ac | EA | ADHD | -0.69 | 0.075 | -9.234 | 2.60E-20 |
|  | EA | ALS | -0.10 | 0.075 | -1.373 | 1.70E-01 |
|  | EA | AD | -0.10 | 0.048 | -2.126 | 3.35E-02 |
|  | EA | AN | 0.26 | 0.076 | 3.478 | 5.00E-04 |
|  | EA | ASD | 0.15 | 0.070 | 2.101 | 3.56E-02 |
|  | EA | AUD | 0.23 | 0.066 | 3.455 | 6.00E-04 |
|  | EA | BIP | 0.16 | 0.050 | 3.188 | 1.40E-03 |
|  | EA | CUD | -0.38 | 0.070 | -5.429 | 5.68E-08 |
|  | EA | MDD | -0.25 | 0.087 | -2.886 | 3.90E-03 |
|  | EA | OCD | 0.37 | 0.105 | 3.529 | 4.00E-04 |
|  | EA | PTSD | -0.46 | 0.233 | -1.968 | 4.90E-02 |
|  | EA | TS | -0.11 | 0.089 | -1.183 | 2.37E-01 |
| Intron | EA | ADHD | -0.73 | 0.068 | -10.738 | 6.77E-27 |
|  | EA | ALS | -0.11 | 0.081 | -1.303 | 1.93E-01 |
|  | EA | AD | -0.28 | 0.063 | -4.404 | 1.06E-05 |
|  | EA | AN | 0.26 | 0.068 | 3.766 | 2.00E-04 |
|  | EA | ASD | 0.06 | 0.069 | 0.887 | 3.75E-01 |
|  | EA | AUD | 0.13 | 0.054 | 2.505 | 1.23E-02 |
|  | EA | BIP | 0.17 | 0.043 | 3.817 | 1.00E-04 |
|  | EA | CUD | -0.45 | 0.067 | -6.662 | 2.70E-11 |
|  | EA | MDD | -0.21 | 0.068 | -3.061 | 2.20E-03 |
|  | EA | OCD | 0.41 | 0.108 | 3.825 | 1.00E-04 |
|  | EA | PTSD | -0.66 | 0.376 | -1.750 | 8.02E-02 |
|  | EA | TS | -0.02 | 0.073 | -0.289 | 7.72E-01 |
| Repressed | EA | ADHD | -0.45 | 0.059 | -7.694 | 1.42E-14 |
|  | EA | ALS | 0.00 | 0.000 | 0.000 | 0.00E+00 |
|  | EA | AD | 0.00 | 0.000 | 0.000 | 0.00E+00 |
|  | EA | AN | 0.27 | 0.062 | 4.381 | 1.18E-05 |
|  | EA | ASD | 0.23 | 0.058 | 3.961 | 7.48E-05 |
|  | EA | AUD | 0.17 | 0.062 | 2.765 | 5.70E-03 |
|  | EA | BIP | 0.25 | 0.062 | 4.047 | 5.18E-05 |
|  | EA | CUD | -0.39 | 0.068 | -5.816 | 6.01E-09 |
|  | EA | MDD | -0.22 | 0.057 | -3.841 | 1.00E-04 |
|  | EA | OCD | 0.18 | 0.113 | 1.593 | 1.11E-01 |
|  | EA | PTSD | -0.20 | 0.082 | -2.492 | 1.27E-02 |
|  | EA | TS | -0.17 | 0.086 | -1.958 | 5.02E-02 |
| Super Enhancer | EA | ADHD | -0.72 | 0.240 | -2.980 | 2.90E-03 |
|  | EA | ALS | 0.01 | 0.114 | 0.058 | 9.53E-01 |
|  | EA | AD | -0.12 | 0.068 | -1.713 | 8.66E-02 |
|  | EA | AN | 0.14 | 0.169 | 0.845 | 3.98E-01 |
|  | EA | ASD | 0.08 | 0.137 | 0.590 | 5.55E-01 |
|  | EA | AUD | 0.28 | 0.119 | 2.352 | 1.87E-02 |
|  | EA | BIP | 0.19 | 0.079 | 2.374 | 1.76E-02 |
|  | EA | CUD | -0.37 | 0.165 | -2.230 | 2.57E-02 |
|  | EA | MDD | -0.22 | 0.191 | -1.151 | 2.50E-01 |
|  | EA | OCD | 0.44 | 0.267 | 1.664 | 9.61E-02 |
|  | EA | PTSD | 0.00 | 0.000 | 0.000 | 0.00E+00 |
|  | EA | TS | -0.20 | 0.173 | -1.151 | 2.50E-01 |
| TFBS | EA | ADHD | -0.60 | 0.056 | -10.714 | 8.72E-27 |
|  | EA | ALS | -0.06 | 0.075 | -0.855 | 3.93E-01 |
|  | EA | AD | -0.10 | 0.043 | -2.310 | 2.09E-02 |
|  | EA | AN | 0.24 | 0.063 | 3.849 | 1.00E-04 |
|  | EA | ASD | 0.19 | 0.059 | 3.183 | 1.50E-03 |
|  | EA | AUD | 0.20 | 0.055 | 3.548 | 4.00E-04 |
|  | EA | BIP | 0.16 | 0.044 | 3.575 | 4.00E-04 |
|  | EA | CUD | -0.37 | 0.058 | -6.379 | 1.78E-10 |
|  | EA | MDD | -0.24 | 0.069 | -3.437 | 6.00E-04 |
|  | EA | OCD | 0.29 | 0.084 | 3.426 | 6.00E-04 |
|  | EA | PTSD | -0.31 | 0.132 | -2.382 | 1.72E-02 |
|  | EA | TS | -0.08 | 0.084 | -0.906 | 3.65E-01 |
| Transcribed | EA | ADHD | -0.55 | 0.043 | -12.767 | 2.50E-37 |
|  | EA | ALS | -0.06 | 0.071 | -0.881 | 3.78E-01 |
|  | EA | AD | -0.17 | 0.046 | -3.679 | 2.00E-04 |
|  | EA | AN | 0.21 | 0.053 | 3.994 | 6.51E-05 |
|  | EA | ASD | 0.20 | 0.052 | 3.770 | 2.00E-04 |
|  | EA | AUD | 0.20 | 0.044 | 4.510 | 6.49E-06 |
|  | EA | BIP | 0.21 | 0.037 | 5.511 | 3.56E-08 |
|  | EA | CUD | -0.42 | 0.053 | -8.038 | 9.13E-16 |
|  | EA | MDD | -0.20 | 0.054 | -3.650 | 3.00E-04 |
|  | EA | OCD | 0.29 | 0.071 | 4.050 | 5.12E-05 |
|  | EA | PTSD | -0.29 | 0.102 | -2.878 | 4.00E-03 |
|  | EA | TS | -0.11 | 0.067 | -1.594 | 1.11E-01 |

Abbreviations: *rg=*genetic correlation estimate; *se*=standard error; EA: Educational attainment; ADHD: Attention deficit/hyperactivity disorder; ALS: Amyotrophic lateral sclerosis; AD: Alzheimer’s dementia; AN: Anorexia nervosa; ASD: Autism spectrum disorders; AUD: Alcohol use disorders; BIP: Bipolar disorder; CUD: Cannabis use disorder; MDD: Major depressive disorder; OCD: Obsessive compulsive disorder; PTSD: Posttraumatic stress disorder; TS: Tourette’s syndrome; DGF=DNase digital genomic footprints; DHS=DNaseI hypersensitivity sites; Fetal DHS=DNaseI hypersensitivity sites for fetal tissues; H3K4me1, H3K4me3, H3K9ac, and H3K27ac=Histone marks; TFBS= transcription factor binding sites. Summary statistics for each trait were merged with Hapmap3 SNPs excluding the HLA region to estimate *rg*.

## Supplementary Table 4. Fine-mapping 99% credible-set of sentinel SNP from cross-trait meta-analysis between educational attainment (EA) and attention deficit/hyperactivity disorder (ADHD).

| Sentinel SNP | Credible-set SNPs | CHR | BP | EA | | | ADHD | | |
| --- | --- | --- | --- | --- | --- | --- | --- | --- | --- |
|  |  |  |  | **GWAS-p-value** | **probNorm** | **cumSum** | **GWAS-p-value** | **probNorm** | **cumSum** |
| rs304137 | rs10462335 | 5 | 88088204 | 1.73E-10 | 0.015341 | 0.494328 | 0.000367 | 0.000592 | 0.9863 |
|  | rs1081158 | 5 | 88004616 | 3.25E-10 | 0.008279 | 0.963716 | 0.000707 | 0.000321 | 0.988652 |
|  | rs12054920 | 5 | 88161417 | 6.18E-11 | 0.041966 | 0.131093 | 2.16E-06 | 0.077726 | 0.670157 |
|  | rs1671539 | 5 | 88168395 | 1.97E-10 | 0.013506 | 0.683663 | 2.07E-06 | 0.080784 | 0.431683 |
|  | rs17558396 | 5 | 88102281 | 2.17E-10 | 0.012311 | 0.746497 | 0.000346 | 0.000625 | 0.985708 |
|  | rs185291 | 5 | 88006764 | 2.16E-10 | 0.012344 | 0.734186 | 0.000687 | 0.00033 | 0.987361 |
|  | rs188581 | 5 | 88175251 | 1.78E-09 | 0.001574 | 0.984333 | 2.72E-05 | 0.006904 | 0.95353 |
|  | rs188582 | 5 | 88167740 | 1.48E-10 | 0.017869 | 0.399477 | 2.08E-06 | 0.080412 | 0.512094 |
|  | rs194225 | 5 | 88001186 | 2.03E-10 | 0.013122 | 0.696785 | 0.000727 | 0.000313 | 0.988965 |
|  | rs216057 | 5 | 88170331 | 5.78E-10 | 0.004728 | 0.976135 | 1.74E-06 | 0.095586 | 0.095586 |
|  | rs254781 | 5 | 88000636 | 1.67E-10 | 0.01587 | 0.447935 | 0.000699 | 0.000325 | 0.987685 |
|  | rs304136 | 5 | 88170066 | 2.46E-10 | 0.010868 | 0.815074 | 2.33E-06 | 0.072089 | 0.742246 |
|  | rs304137 | 5 | 88169652 | 1.78E-10 | 0.014903 | 0.569831 | 1.79E-06 | 0.093219 | 0.188804 |
|  | rs304138 | 5 | 88169567 | 2.64E-10 | 0.010141 | 0.888261 | 2.07E-06 | 0.080934 | 0.350898 |
|  | rs34316 | 5 | 88015545 | 1.21E-10 | 0.021827 | 0.360164 | 0.000857 | 0.000269 | 0.99013 |
|  | rs34338 | 5 | 87993365 | 5.6E-11 | 0.046204 | 0.046204 | 0.000179 | 0.001158 | 0.982799 |
|  | rs410671 | 5 | 87993198 | 6.82E-11 | 0.03811 | 0.169203 | 0.000183 | 0.001138 | 0.985083 |
|  | rs42850 | 5 | 88019474 | 9.98E-11 | 0.026264 | 0.286548 | 0.000825 | 0.000278 | 0.989862 |
|  | rs446219 | 5 | 88005827 | 2.12E-09 | 0.001332 | 0.990098 | 0.000615 | 0.000366 | 0.987031 |
|  | rs447801 | 5 | 88002653 | 1.86E-10 | 0.014308 | 0.627918 | 0.00073 | 0.000312 | 0.989277 |
|  | rs4518438 | 5 | 88157552 | 9.51E-10 | 0.002907 | 0.979043 | 1.17E-05 | 0.015465 | 0.917202 |
|  | rs56947091 | 5 | 88160354 | 2.51E-10 | 0.010669 | 0.83645 | 1.27E-05 | 0.014232 | 0.946625 |
|  | rs61104616 | 5 | 88163771 | 6.04E-11 | 0.042924 | 0.089128 | 2.09E-06 | 0.080337 | 0.592432 |
|  | rs62380364 | 5 | 88107337 | 9.99E-11 | 0.026251 | 0.312799 | 0.000181 | 0.001146 | 0.983945 |
|  | rs639725 | 5 | 88001969 | 2.16E-10 | 0.01235 | 0.721842 | 0.000744 | 0.000306 | 0.989584 |
|  | rs640177 | 5 | 88002084 | 2.26E-10 | 0.011796 | 0.770165 | 0.000704 | 0.000322 | 0.988331 |
|  | rs797419 | 5 | 88168955 | 1.81E-10 | 0.014647 | 0.599243 | 2.06E-06 | 0.08116 | 0.269964 |
| rs3791101 | rs1013793 | 1 | 44207553 | 6.74E-10 | 0.006692 | 0.800455 | 3.88E-11 | 0.003029 | 0.940518 |
|  | rs1029632 | 1 | 44207899 | 6.79E-10 | 0.00665 | 0.807105 | 4.26E-11 | 0.002761 | 0.963604 |
|  | rs10890280 | 1 | 44238218 | 7.25E-10 | 0.00624 | 0.877947 | 3.22E-11 | 0.003636 | 0.903129 |
|  | rs10890281 | 1 | 44245939 | 8.34E-10 | 0.005439 | 0.964472 | 1.14E-11 | 0.010054 | 0.793738 |
|  | rs11210912 | 1 | 44220737 | 9.45E-10 | 0.004815 | 0.979647 | 4.98E-11 | 0.002372 | 0.973438 |
|  | rs11210914 | 1 | 44223456 | 1.2E-09 | 0.003809 | 0.98809 | 4.07E-11 | 0.002889 | 0.949343 |
|  | rs11210916 | 1 | 44232926 | 6.96E-10 | 0.006489 | 0.839638 | 2.97E-11 | 0.003931 | 0.895718 |
|  | rs11210917 | 1 | 44234520 | 6.91E-10 | 0.006532 | 0.813637 | 3.3E-11 | 0.003548 | 0.910275 |
|  | rs112361411 | 1 | 44183923 | 8.93E-11 | 0.048198 | 0.36649 | 1.13E-12 | 0.097206 | 0.400296 |
|  | rs112518613 | 1 | 44224985 | 9.03E-10 | 0.005035 | 0.974832 | 4.96E-11 | 0.002381 | 0.971067 |
|  | rs112646031 | 1 | 44213051 | 4.97E-10 | 0.009014 | 0.728377 | 2.95E-11 | 0.003953 | 0.891787 |
|  | rs11581514 | 1 | 44222204 | 7.08E-10 | 0.00638 | 0.865398 | 4.08E-11 | 0.002879 | 0.955102 |
|  | rs11584382 | 1 | 44216634 | 7.25E-10 | 0.006239 | 0.884186 | 3.47E-11 | 0.003374 | 0.927522 |
|  | rs11587515 | 1 | 44222570 | 8.17E-10 | 0.005547 | 0.942592 | 3.46E-11 | 0.003381 | 0.924148 |
|  | rs11590279 | 1 | 44244183 | 6.95E-10 | 0.006499 | 0.833149 | 1.09E-11 | 0.010516 | 0.783684 |
|  | rs11805774 | 1 | 44152715 | 1.4E-09 | 0.003276 | 0.991365 | 4.05E-12 | 0.027652 | 0.666177 |
|  | rs11810109 | 1 | 44186812 | 6.5E-11 | 0.065814 | 0.261249 | 2.33E-12 | 0.047506 | 0.542288 |
|  | rs12062824 | 1 | 44215859 | 8.24E-10 | 0.005505 | 0.948097 | 4.98E-11 | 0.00237 | 0.975809 |
|  | rs12083110 | 1 | 44245049 | 7.5E-10 | 0.006037 | 0.902386 | 1.25E-11 | 0.009142 | 0.81284 |
|  | rs12354267 | 1 | 44248272 | 9.83E-10 | 0.004634 | 0.984281 | 2.99E-12 | 0.037249 | 0.579537 |
|  | rs12410155 | 1 | 44188465 | 1.25E-10 | 0.034759 | 0.522425 | 1.09E-12 | 0.100268 | 0.303091 |
|  | rs12410444 | 1 | 44188719 | 2.14E-11 | 0.195435 | 0.195435 | 1.16E-12 | 0.094486 | 0.494783 |
|  | rs1472661 | 1 | 44209075 | 6.71E-10 | 0.006727 | 0.793763 | 3.56E-11 | 0.003295 | 0.937488 |
|  | rs17401357 | 1 | 44153619 | 3.31E-10 | 0.013424 | 0.65251 | 3.97E-12 | 0.028197 | 0.638525 |
|  | rs17531412 | 1 | 44182244 | 7.52E-11 | 0.057043 | 0.318292 | 1.07E-12 | 0.10192 | 0.10192 |
|  | rs1971342 | 1 | 44152189 | 3.39E-10 | 0.013095 | 0.678934 | 4.09E-12 | 0.027393 | 0.69357 |
|  | rs2158954 | 1 | 44244667 | 7.17E-10 | 0.006308 | 0.871707 | 1.26E-11 | 0.009085 | 0.821925 |
|  | rs2270972 | 1 | 44158129 | 2.86E-10 | 0.015439 | 0.625366 | 3.63E-12 | 0.030791 | 0.610328 |
|  | rs3791035 | 1 | 44154479 | 3.23E-10 | 0.01372 | 0.639086 | 4.38E-12 | 0.025626 | 0.773169 |
|  | rs3791040 | 1 | 44202733 | 4.7E-10 | 0.009521 | 0.719363 | 5.38E-11 | 0.002198 | 0.992042 |
|  | rs3791043 | 1 | 44219546 | 7.5E-10 | 0.006035 | 0.908421 | 4.09E-11 | 0.002871 | 0.957973 |
|  | rs3791044 | 1 | 44219933 | 6.49E-10 | 0.006948 | 0.773449 | 2.86E-11 | 0.004074 | 0.879884 |
|  | rs3791046 | 1 | 44231807 | 6.94E-10 | 0.006505 | 0.826649 | 2.95E-11 | 0.003957 | 0.887833 |
|  | rs3791047 | 1 | 44236114 | 6.08E-10 | 0.00741 | 0.766501 | 3.32E-11 | 0.00352 | 0.917319 |
|  | rs4660257 | 1 | 44148168 | 2.4E-10 | 0.018363 | 0.609928 | 4.15E-12 | 0.026992 | 0.720563 |
|  | rs4660745 | 1 | 44234076 | 6.94E-10 | 0.006508 | 0.820144 | 3.39E-11 | 0.003449 | 0.920767 |
|  | rs55695044 | 1 | 44243658 | 7E-10 | 0.006455 | 0.852582 | 1.3E-11 | 0.008805 | 0.83073 |
|  | rs56338811 | 1 | 44214888 | 8.14E-10 | 0.005572 | 0.937045 | 5E-11 | 0.00236 | 0.985264 |
|  | rs56367078 | 1 | 44215015 | 7.86E-10 | 0.005766 | 0.920116 | 4.1E-11 | 0.002869 | 0.960843 |
|  | rs56701980 | 1 | 44215412 | 8.27E-10 | 0.005484 | 0.953581 | 4.98E-11 | 0.00237 | 0.978179 |
|  | rs59305903 | 1 | 44210191 | 4.65E-10 | 0.009611 | 0.709842 | 3.93E-11 | 0.002989 | 0.943506 |
|  | rs61768374 | 1 | 44151633 | 3.33E-10 | 0.013329 | 0.665839 | 4.16E-12 | 0.02698 | 0.747542 |
|  | rs61768432 | 1 | 44243561 | 7.02E-10 | 0.006437 | 0.859019 | 1.32E-11 | 0.008726 | 0.839456 |
|  | rs6429637 | 1 | 44198531 | 4.17E-10 | 0.010693 | 0.689627 | 3.48E-11 | 0.003365 | 0.930887 |
|  | rs6429639 | 1 | 44217753 | 7.45E-10 | 0.006072 | 0.896349 | 4.08E-11 | 0.00288 | 0.952223 |
|  | rs6656457 | 1 | 44233662 | 6.64E-10 | 0.006797 | 0.780246 | 3.54E-11 | 0.003306 | 0.934193 |
|  | rs6664485 | 1 | 44225532 | 6.65E-10 | 0.006789 | 0.787036 | 4.93E-11 | 0.002392 | 0.968685 |
|  | rs6668616 | 1 | 44216376 | 8.32E-10 | 0.005453 | 0.959034 | 5.02E-11 | 0.002351 | 0.987614 |
|  | rs6673970 | 1 | 44199469 | 4.21E-10 | 0.010604 | 0.700231 | 3.98E-11 | 0.002948 | 0.946454 |
|  | rs6678767 | 1 | 44220670 | 8.52E-10 | 0.005326 | 0.969798 | 4.99E-11 | 0.002365 | 0.980544 |
|  | rs6700256 | 1 | 44216887 | 8.08E-10 | 0.00561 | 0.931473 | 5E-11 | 0.00236 | 0.982904 |
|  | rs7516647 | 1 | 44237465 | 5.64E-10 | 0.007974 | 0.736351 | 2.14E-11 | 0.005419 | 0.866345 |
|  | rs7520053 | 1 | 44226657 | 7.88E-10 | 0.005746 | 0.925863 | 3.09E-11 | 0.003776 | 0.899494 |
|  | rs7529895 | 1 | 44241870 | 5.74E-10 | 0.007829 | 0.74418 | 1.15E-11 | 0.009959 | 0.803698 |
|  | rs7531165 | 1 | 44237247 | 6.96E-10 | 0.006489 | 0.846127 | 3.32E-11 | 0.003524 | 0.913799 |
|  | rs7551551 | 1 | 44213482 | 6E-10 | 0.007498 | 0.751679 | 2.92E-11 | 0.003992 | 0.883876 |
|  | rs7551644 | 1 | 44228016 | 7.43E-10 | 0.006091 | 0.890277 | 3.25E-11 | 0.003597 | 0.906726 |
| rs549845 | rs10749850 | 1 | 44205094 | 8.9E-08 | 0.001295 | 0.961773 | 6.93E-09 | 0.003677 | 0.976303 |
|  | rs10789436 | 1 | 44040966 | 4.17E-09 | 0.025278 | 0.70123 | 5.45E-09 | 0.004642 | 0.954517 |
|  | rs10890255 | 1 | 44031493 | 6.04E-09 | 0.017623 | 0.844756 | 4.18E-08 | 0.000642 | 0.989956 |
|  | rs10890261 | 1 | 44052377 | 6.59E-09 | 0.016191 | 0.860947 | 1.36E-09 | 0.017986 | 0.795433 |
|  | rs10890273 | 1 | 44186444 | 8.63E-08 | 0.001334 | 0.960478 | 8.89E-10 | 0.027174 | 0.513029 |
|  | rs10890275 | 1 | 44194216 | 7.87E-08 | 0.001459 | 0.956446 | 1.02E-09 | 0.023871 | 0.658925 |
|  | rs11210871 | 1 | 44029353 | 6.84E-09 | 0.015626 | 0.892427 | 3.43E-09 | 0.007288 | 0.872413 |
|  | rs11210887 | 1 | 44076019 | 4.36E-08 | 0.002587 | 0.940783 | 1.48E-10 | 0.156378 | 0.323703 |
|  | rs11210907 | 1 | 44191759 | 9.15E-08 | 0.00126 | 0.963033 | 9.52E-10 | 0.025423 | 0.538452 |
|  | rs11210913 | 1 | 44222597 | 1.25E-07 | 0.000933 | 0.973325 | 4.46E-09 | 0.005646 | 0.902204 |
|  | rs11210915 | 1 | 44227579 | 9.38E-08 | 0.00123 | 0.964263 | 4.7E-09 | 0.005367 | 0.924138 |
|  | rs12119149 | 1 | 44243283 | 1.4E-07 | 0.000836 | 0.977809 | 2.81E-09 | 0.008844 | 0.856299 |
|  | rs2004899 | 1 | 44045465 | 3.75E-09 | 0.02806 | 0.62145 | 1.27E-09 | 0.019141 | 0.740778 |
|  | rs2158956 | 1 | 44211697 | 1.3E-07 | 0.0009 | 0.976973 | 4.73E-09 | 0.005332 | 0.92947 |
|  | rs2819340 | 1 | 44039710 | 5.87E-09 | 0.018126 | 0.809429 | 3.75E-09 | 0.006682 | 0.879095 |
|  | rs2842186 | 1 | 44035093 | 9.46E-09 | 0.011393 | 0.931653 | 2.31E-08 | 0.00114 | 0.988516 |
|  | rs28833034 | 1 | 44212942 | 1.23E-07 | 0.000948 | 0.971454 | 4.82E-09 | 0.005235 | 0.940002 |
|  | rs3001723 | 1 | 44037685 | 8.44E-08 | 0.001363 | 0.957809 | 4.94E-10 | 0.04819 | 0.371894 |
|  | rs304303 | 1 | 44178070 | 7.06E-08 | 0.001621 | 0.951899 | 1.23E-08 | 0.002111 | 0.987376 |
|  | rs3791041 | 1 | 44202991 | 1.04E-07 | 0.001109 | 0.965372 | 5.11E-09 | 0.004944 | 0.944945 |
|  | rs3791042 | 1 | 44219275 | 1.21E-07 | 0.000962 | 0.969553 | 4.59E-09 | 0.005492 | 0.918771 |
|  | rs3791137 | 1 | 44050004 | 4.5E-09 | 0.023477 | 0.748368 | 1.33E-09 | 0.018341 | 0.75912 |
|  | rs3791138 | 1 | 44050027 | 2.93E-09 | 0.035669 | 0.461475 | 1.26E-09 | 0.019379 | 0.721637 |
|  | rs3828150 | 1 | 44050856 | 5.14E-09 | 0.020634 | 0.791303 | 1.72E-09 | 0.0143 | 0.809733 |
|  | rs3862228 | 1 | 44196945 | 7.39E-08 | 0.001551 | 0.953449 | 1.01E-09 | 0.023963 | 0.635054 |
|  | rs4660260 | 1 | 44195353 | 7.45E-08 | 0.001538 | 0.954987 | 1.08E-09 | 0.022551 | 0.681476 |
|  | rs4660261 | 1 | 44229597 | 1.1E-07 | 0.001059 | 0.968592 | 4.53E-09 | 0.005563 | 0.907767 |
|  | rs4660743 | 1 | 44195404 | 6.39E-08 | 0.001783 | 0.948521 | 1E-09 | 0.024197 | 0.586964 |
|  | rs519669 | 1 | 44075505 | 4.74E-09 | 0.022301 | 0.770669 | 5.57E-09 | 0.004547 | 0.968222 |
|  | rs539096 | 1 | 44072420 | 1.67E-08 | 0.006543 | 0.938196 | 4.44E-09 | 0.005669 | 0.896558 |
|  | rs549845 | 1 | 44076469 | 5.78E-08 | 0.001968 | 0.944949 | 1.38E-10 | 0.167325 | 0.167325 |
|  | rs55656032 | 1 | 44098739 | 7.99E-09 | 0.013428 | 0.92026 | 4.37E-08 | 0.000615 | 0.990571 |
|  | rs573350 | 1 | 44057950 | 3.83E-09 | 0.027453 | 0.648904 | 1.33E-09 | 0.018328 | 0.777448 |
|  | rs631248 | 1 | 44071221 | 6.73E-09 | 0.015854 | 0.876801 | 8.81E-10 | 0.027406 | 0.485855 |
|  | rs6429636 | 1 | 44183540 | 8.62E-08 | 0.001335 | 0.959144 | 9.96E-10 | 0.024315 | 0.562767 |
|  | rs6429638 | 1 | 44211010 | 1.27E-07 | 0.00092 | 0.974245 | 5.13E-09 | 0.00493 | 0.949876 |
|  | rs650729 | 1 | 44042539 | 6.01E-09 | 0.017703 | 0.827132 | 2.77E-09 | 0.00898 | 0.847456 |
|  | rs663618 | 1 | 44043105 | 4.46E-09 | 0.023661 | 0.724891 | 2.55E-09 | 0.009731 | 0.819464 |
|  | rs6669157 | 1 | 44216876 | 1.09E-07 | 0.001063 | 0.967532 | 4.57E-09 | 0.005512 | 0.913279 |
|  | rs6686238 | 1 | 44210253 | 1.28E-07 | 0.000913 | 0.976073 | 4.76E-09 | 0.005297 | 0.934767 |
|  | rs674725 | 1 | 44061795 | 7.43E-09 | 0.014404 | 0.906831 | 2.58E-09 | 0.009624 | 0.829088 |
|  | rs7538463 | 1 | 44196416 | 6.49E-08 | 0.001757 | 0.950278 | 1E-09 | 0.024126 | 0.61109 |
|  | rs7543520 | 1 | 44243866 | 1.51E-07 | 0.000778 | 0.98101 | 2.82E-09 | 0.008825 | 0.865124 |
|  | rs7549094 | 1 | 44213178 | 1.06E-07 | 0.001097 | 0.966469 | 7.45E-09 | 0.003428 | 0.979731 |
| rs673253 | rs1013793 | 1 | 44207553 | 6.74E-10 | 0.00402 | 0.851751 | 3.88E-11 | 0.002894 | 0.910489 |
|  | rs1029632 | 1 | 44207899 | 6.79E-10 | 0.003994 | 0.855746 | 4.26E-11 | 0.002638 | 0.935218 |
|  | rs10890280 | 1 | 44238218 | 7.25E-10 | 0.003748 | 0.898298 | 3.22E-11 | 0.003473 | 0.874773 |
|  | rs10890281 | 1 | 44245939 | 8.34E-10 | 0.003267 | 0.96062 | 1.14E-11 | 0.009604 | 0.75823 |
|  | rs11210912 | 1 | 44220737 | 9.45E-10 | 0.002892 | 0.972737 | 4.98E-11 | 0.002266 | 0.947144 |
|  | rs11210914 | 1 | 44223456 | 1.2E-09 | 0.002288 | 0.977808 | 4.07E-11 | 0.002759 | 0.91892 |
|  | rs11210916 | 1 | 44232926 | 6.96E-10 | 0.003898 | 0.875287 | 2.97E-11 | 0.003755 | 0.867694 |
|  | rs11210917 | 1 | 44234520 | 6.91E-10 | 0.003923 | 0.859669 | 3.3E-11 | 0.003389 | 0.881599 |
|  | rs112361411 | 1 | 44183923 | 8.93E-11 | 0.02895 | 0.262062 | 1.13E-12 | 0.092857 | 0.382389 |
|  | rs112518613 | 1 | 44224985 | 9.03E-10 | 0.003024 | 0.966843 | 4.96E-11 | 0.002275 | 0.944878 |
|  | rs112646031 | 1 | 44213051 | 4.97E-10 | 0.005414 | 0.776626 | 2.95E-11 | 0.003777 | 0.863938 |
|  | rs112984125 | 1 | 44173423 | 2.04E-09 | 0.001364 | 0.986629 | 1.08E-12 | 0.09639 | 0.19375 |
|  | rs11581514 | 1 | 44222204 | 7.08E-10 | 0.003832 | 0.89076 | 4.08E-11 | 0.00275 | 0.924421 |
|  | rs11584382 | 1 | 44216634 | 7.25E-10 | 0.003747 | 0.902045 | 3.47E-11 | 0.003223 | 0.898075 |
|  | rs11587515 | 1 | 44222570 | 8.17E-10 | 0.003332 | 0.944185 | 3.46E-11 | 0.003229 | 0.894852 |
|  | rs11590279 | 1 | 44244183 | 6.95E-10 | 0.003904 | 0.871389 | 1.09E-11 | 0.010045 | 0.748626 |
|  | rs11805774 | 1 | 44152715 | 1.4E-09 | 0.001968 | 0.983886 | 4.05E-12 | 0.026415 | 0.636376 |
|  | rs11810109 | 1 | 44186812 | 6.5E-11 | 0.039532 | 0.198848 | 2.33E-12 | 0.045381 | 0.518029 |
|  | rs12062824 | 1 | 44215859 | 8.24E-10 | 0.003307 | 0.947491 | 4.98E-11 | 0.002264 | 0.949408 |
|  | rs12083110 | 1 | 44245049 | 7.5E-10 | 0.003626 | 0.916646 | 1.25E-11 | 0.008733 | 0.776477 |
|  | rs12354267 | 1 | 44248272 | 9.83E-10 | 0.002783 | 0.97552 | 2.99E-12 | 0.035582 | 0.553611 |
|  | rs12410155 | 1 | 44188465 | 1.25E-10 | 0.020878 | 0.409026 | 1.09E-12 | 0.095782 | 0.289532 |
|  | rs12410444 | 1 | 44188719 | 2.14E-11 | 0.11739 | 0.11739 | 1.16E-12 | 0.09026 | 0.472649 |
|  | rs1472661 | 1 | 44209075 | 6.71E-10 | 0.004041 | 0.839666 | 3.56E-11 | 0.003148 | 0.907595 |
|  | rs17371903 | 1 | 44070691 | 1.39E-09 | 0.001986 | 0.981918 | 2.28E-11 | 0.004863 | 0.839635 |
|  | rs17401357 | 1 | 44153619 | 3.31E-10 | 0.008063 | 0.676618 | 3.97E-12 | 0.026936 | 0.609961 |
|  | rs17531412 | 1 | 44182244 | 7.52E-11 | 0.034264 | 0.233112 | 1.07E-12 | 0.09736 | 0.09736 |
|  | rs1808730 | 1 | 44045528 | 1.84E-10 | 0.014294 | 0.517343 | 8.49E-11 | 0.001344 | 0.983477 |
|  | rs1971342 | 1 | 44152189 | 3.39E-10 | 0.007866 | 0.708466 | 4.09E-12 | 0.026168 | 0.662544 |
|  | rs2158954 | 1 | 44244667 | 7.17E-10 | 0.003789 | 0.894549 | 1.26E-11 | 0.008679 | 0.785156 |
|  | rs2270972 | 1 | 44158129 | 2.86E-10 | 0.009273 | 0.607748 | 3.63E-12 | 0.029413 | 0.583025 |
|  | rs2842171 | 1 | 44039850 | 8.04E-10 | 0.003388 | 0.934136 | 1.65E-10 | 0.000703 | 0.9887 |
|  | rs3791035 | 1 | 44154479 | 3.23E-10 | 0.008241 | 0.668555 | 4.38E-12 | 0.02448 | 0.738581 |
|  | rs3791040 | 1 | 44202733 | 4.7E-10 | 0.005719 | 0.771212 | 5.38E-11 | 0.0021 | 0.962785 |
|  | rs3791043 | 1 | 44219546 | 7.5E-10 | 0.003625 | 0.920271 | 4.09E-11 | 0.002743 | 0.927164 |
|  | rs3791044 | 1 | 44219933 | 6.49E-10 | 0.004173 | 0.827465 | 2.86E-11 | 0.003891 | 0.852568 |
|  | rs3791046 | 1 | 44231807 | 6.94E-10 | 0.003907 | 0.867485 | 2.95E-11 | 0.00378 | 0.860162 |
|  | rs3791047 | 1 | 44236114 | 6.08E-10 | 0.004451 | 0.819012 | 3.32E-11 | 0.003362 | 0.888328 |
|  | rs3791134 | 1 | 44049156 | 3.92E-10 | 0.006824 | 0.722355 | 7.66E-11 | 0.001486 | 0.979378 |
|  | rs3791136 | 1 | 44049947 | 3.11E-10 | 0.008562 | 0.651895 | 5.85E-11 | 0.001936 | 0.970877 |
|  | rs3791139 | 1 | 44051771 | 2.86E-10 | 0.009286 | 0.598475 | 6.6E-11 | 0.001718 | 0.976272 |
|  | rs3791140 | 1 | 44051778 | 2.57E-10 | 0.01032 | 0.549425 | 6.12E-11 | 0.001849 | 0.972727 |
|  | rs4660257 | 1 | 44148168 | 2.4E-10 | 0.01103 | 0.528372 | 4.15E-12 | 0.025785 | 0.688328 |
|  | rs4660745 | 1 | 44234076 | 6.94E-10 | 0.003909 | 0.863578 | 3.39E-11 | 0.003295 | 0.891622 |
|  | rs501299 | 1 | 44051834 | 2.67E-10 | 0.009931 | 0.579384 | 8.47E-11 | 0.001347 | 0.982134 |
|  | rs552638 | 1 | 44043392 | 3.32E-10 | 0.008035 | 0.684653 | 1.02E-10 | 0.001127 | 0.984605 |
|  | rs55695044 | 1 | 44243658 | 7E-10 | 0.003877 | 0.883062 | 1.3E-11 | 0.008411 | 0.793567 |
|  | rs56175694 | 1 | 44065148 | 4.58E-10 | 0.005868 | 0.75972 | 4.2E-11 | 0.002676 | 0.932581 |
|  | rs56338811 | 1 | 44214888 | 8.14E-10 | 0.003347 | 0.940852 | 5E-11 | 0.002254 | 0.956186 |
|  | rs56352978 | 1 | 44231480 | 2.68E-09 | 0.001048 | 0.98886 | 1.69E-11 | 0.00652 | 0.823631 |
|  | rs56367078 | 1 | 44215015 | 7.86E-10 | 0.003463 | 0.927296 | 4.1E-11 | 0.002741 | 0.929905 |
|  | rs56701980 | 1 | 44215412 | 8.27E-10 | 0.003294 | 0.950785 | 4.98E-11 | 0.002264 | 0.951672 |
|  | rs583040 | 1 | 44060483 | 2.93E-10 | 0.009085 | 0.616833 | 5.49E-11 | 0.002058 | 0.966909 |
|  | rs59305903 | 1 | 44210191 | 4.65E-10 | 0.005773 | 0.765493 | 3.93E-11 | 0.002855 | 0.913344 |
|  | rs596522 | 1 | 44066424 | 5.5E-10 | 0.004902 | 0.78671 | 4.44E-11 | 0.002531 | 0.940318 |
|  | rs605709 | 1 | 44058467 | 3.09E-10 | 0.008605 | 0.643333 | 5.56E-11 | 0.002033 | 0.968942 |
|  | rs61768374 | 1 | 44151633 | 3.33E-10 | 0.008006 | 0.692659 | 4.16E-12 | 0.025773 | 0.714101 |
|  | rs61768432 | 1 | 44243561 | 7.02E-10 | 0.003867 | 0.886928 | 1.32E-11 | 0.008336 | 0.801903 |
|  | rs61769642 | 1 | 44051630 | 3.16E-10 | 0.008419 | 0.660314 | 8.09E-11 | 0.001409 | 0.980787 |
|  | rs6429637 | 1 | 44198531 | 4.17E-10 | 0.006423 | 0.73538 | 3.48E-11 | 0.003215 | 0.90129 |
|  | rs6429639 | 1 | 44217753 | 7.45E-10 | 0.003647 | 0.91302 | 4.08E-11 | 0.002751 | 0.921671 |
|  | rs6656457 | 1 | 44233662 | 6.64E-10 | 0.004083 | 0.831548 | 3.54E-11 | 0.003158 | 0.904448 |
|  | rs6664485 | 1 | 44225532 | 6.65E-10 | 0.004078 | 0.835626 | 4.93E-11 | 0.002285 | 0.942603 |
|  | rs6668616 | 1 | 44216376 | 8.32E-10 | 0.003275 | 0.957353 | 5.02E-11 | 0.002245 | 0.960685 |
|  | rs6673970 | 1 | 44199469 | 4.21E-10 | 0.006369 | 0.74175 | 3.98E-11 | 0.002816 | 0.91616 |
|  | rs6678767 | 1 | 44220670 | 8.52E-10 | 0.003199 | 0.963819 | 4.99E-11 | 0.002259 | 0.953932 |
|  | rs6696529 | 1 | 44210387 | 6.07E-10 | 0.004452 | 0.814561 | 2.55E-10 | 0.000459 | 0.990273 |
|  | rs6698389 | 1 | 44051574 | 2.67E-10 | 0.009935 | 0.569453 | 7.01E-11 | 0.00162 | 0.977892 |
|  | rs6700256 | 1 | 44216887 | 8.08E-10 | 0.00337 | 0.937506 | 5E-11 | 0.002254 | 0.95844 |
|  | rs673242 | 1 | 44062159 | 4.06E-10 | 0.006602 | 0.728957 | 5.47E-11 | 0.002066 | 0.964851 |
|  | rs673253 | 1 | 44062154 | 8.28E-10 | 0.003292 | 0.954078 | 1.53E-11 | 0.007182 | 0.817111 |
|  | rs72673082 | 1 | 44059242 | 3.36E-10 | 0.007941 | 0.7006 | 6.2E-11 | 0.001828 | 0.974554 |
|  | rs7511800 | 1 | 44214269 | 2.37E-09 | 0.001183 | 0.987812 | 1.85E-11 | 0.005964 | 0.829595 |
|  | rs7516647 | 1 | 44237465 | 5.64E-10 | 0.00479 | 0.7915 | 2.14E-11 | 0.005177 | 0.834772 |
|  | rs7520053 | 1 | 44226657 | 7.88E-10 | 0.003452 | 0.930748 | 3.09E-11 | 0.003607 | 0.8713 |
|  | rs7529895 | 1 | 44241870 | 5.74E-10 | 0.004703 | 0.800919 | 1.15E-11 | 0.009514 | 0.767744 |
|  | rs7531165 | 1 | 44237247 | 6.96E-10 | 0.003897 | 0.879184 | 3.32E-11 | 0.003366 | 0.884965 |
|  | rs7551551 | 1 | 44213482 | 6E-10 | 0.004504 | 0.810109 | 2.92E-11 | 0.003813 | 0.856381 |
|  | rs7551644 | 1 | 44228016 | 7.43E-10 | 0.003659 | 0.909372 | 3.25E-11 | 0.003436 | 0.87821 |
|  | rs9787076 | 1 | 44141149 | 1.26E-10 | 0.020684 | 0.450556 | 2.13E-10 | 0.000547 | 0.989815 |

Abbreviations: probNorm refers to the normalized posterior p-value for each SNP. cumSum means the cumulative normalized posterior p-value for SNP sets. For each sentinel SNP, a 99% credible set of potentially causal SNPs was obtained using the FM-summary method. Each SNP has a corresponding posterior probability, i.e., probNorm, and with each superimposition of another SNP, there is a subsequent cumSum, which is obtained by the the corresponding probNorm of each SNP is summed up. Ultimately, this can be interpreted to mean that there is a 99% probability that the causal SNPs that actually have an effect on the two traits are contained in such a credible set.

## Supplementary Table 5. Fine-mapping 99% credible-set of sentinel SNP from cross-trait meta-analysis between educational attainment (EA) and anorexia nervosa (AN).

| Sentinel.SNP | Credible-set SNPs | CHR | BP | EA | | | AN | | |
| --- | --- | --- | --- | --- | --- | --- | --- | --- | --- |
|  |  |  |  | **GWAS-p-value** | **probNorm** | **cumSum** | **GWAS-p-value** | **probNorm** | **cumSum** |
| rs13093385 | rs10212296 | 3 | 49542273 | 1E-22 | 0.018298 | 0.288052 | 9.68E-06 | 0.001516 | 0.897261 |
|  | rs1050450 | 3 | 49394834 | 1.19E-22 | 0.015459 | 0.509268 | 1.06E-05 | 0.001389 | 0.924661 |
|  | rs10640 | 3 | 49454277 | 1.4E-22 | 0.01315 | 0.637399 | 1.24E-05 | 0.001195 | 0.969039 |
|  | rs11130203 | 3 | 49575367 | 4.18E-22 | 0.004454 | 0.926986 | 1.2E-05 | 0.001231 | 0.966638 |
|  | rs111903592 | 3 | 49427319 | 2.02E-22 | 0.009123 | 0.840385 | 1.16E-05 | 0.001278 | 0.961607 |
|  | rs11706370 | 3 | 49441091 | 1.95E-22 | 0.009485 | 0.812752 | 1.1E-05 | 0.001337 | 0.935536 |
|  | rs11710037 | 3 | 49675334 | 5E-22 | 0.003726 | 0.963056 | 2E-06 | 0.006883 | 0.788902 |
|  | rs11711536 | 3 | 49391240 | 1.47E-22 | 0.012546 | 0.649945 | 1.05E-05 | 0.001404 | 0.919093 |
|  | rs11712569 | 3 | 49540152 | 3.41E-22 | 0.005443 | 0.898132 | 9.67E-06 | 0.001518 | 0.895745 |
|  | rs11715915 | 3 | 49455330 | 2E-22 | 0.009236 | 0.831261 | 1.08E-05 | 0.00136 | 0.930142 |
|  | rs11716974 | 3 | 49431853 | 1.25E-22 | 0.014724 | 0.568808 | 1.07E-05 | 0.001376 | 0.927418 |
|  | rs11719996 | 3 | 49525958 | 2.65E-22 | 0.006995 | 0.887203 | 9.95E-06 | 0.001477 | 0.906251 |
|  | rs11720597 | 3 | 49635354 | 8.63E-22 | 0.002173 | 0.985992 | 6.29E-06 | 0.00229 | 0.856665 |
|  | rs11917431 | 3 | 49644012 | 4.68E-22 | 0.00398 | 0.95179 | 2.36E-06 | 0.005869 | 0.839823 |
|  | rs11921590 | 3 | 49644193 | 4.36E-22 | 0.004274 | 0.93126 | 2.57E-06 | 0.005397 | 0.84522 |
|  | rs11922013 | 3 | 49458355 | 1.01E-22 | 0.018226 | 0.306278 | 1.14E-05 | 0.0013 | 0.950003 |
|  | rs12330269 | 3 | 49513910 | 1.83E-22 | 0.010097 | 0.774078 | 1.06E-05 | 0.001392 | 0.921883 |
|  | rs13062429 | 3 | 49559485 | 7.29E-22 | 0.002567 | 0.974235 | 8.36E-06 | 0.001743 | 0.870045 |
|  | rs13064576 | 3 | 49642430 | 4.08E-22 | 0.004555 | 0.922532 | 2.65E-06 | 0.005233 | 0.850453 |
|  | rs13079082 | 3 | 49574808 | 6.13E-22 | 0.00305 | 0.966105 | 1.23E-05 | 0.001207 | 0.967844 |
|  | rs13079643 | 3 | 49477466 | 1.61E-22 | 0.011429 | 0.698269 | 1.12E-05 | 0.001323 | 0.940841 |
|  | rs13087851 | 3 | 49408974 | 1.14E-22 | 0.016062 | 0.462083 | 1.15E-05 | 0.001287 | 0.959041 |
|  | rs13090388 | 3 | 49391082 | 4.29E-23 | 0.042406 | 0.186096 | 1.05E-05 | 0.001406 | 0.917689 |
|  | rs13093385 | 3 | 49710479 | 3.43E-22 | 0.00541 | 0.903541 | 1E-06 | 0.013321 | 0.531325 |
|  | rs148734725 | 3 | 49406708 | 1.25E-23 | 0.143691 | 0.143691 | 1.11E-05 | 0.00133 | 0.936866 |
|  | rs17080528 | 3 | 49389842 | 1.05E-22 | 0.01742 | 0.412978 | 1.03E-05 | 0.001431 | 0.912027 |
|  | rs1800668 | 3 | 49395757 | 1.51E-22 | 0.012161 | 0.68684 | 1.01E-05 | 0.001462 | 0.907714 |
|  | rs1801143 | 3 | 49570200 | 3.78E-22 | 0.004911 | 0.913394 | 1.14E-05 | 0.001298 | 0.951301 |
|  | rs1873625 | 3 | 49666964 | 4.06E-22 | 0.004583 | 0.917977 | 1.91E-06 | 0.007163 | 0.782019 |
|  | rs1987628 | 3 | 49399259 | 1.48E-22 | 0.012462 | 0.662407 | 1.04E-05 | 0.001414 | 0.914869 |
|  | rs34196454 | 3 | 49563456 | 2.03E-22 | 0.009119 | 0.849504 | 1.02E-05 | 0.001449 | 0.909162 |
|  | rs34293138 | 3 | 49579017 | 7.14E-22 | 0.002619 | 0.971668 | 1.11E-05 | 0.001325 | 0.939518 |
|  | rs34363169 | 3 | 49537712 | 2.38E-22 | 0.007762 | 0.865267 | 9.42E-06 | 0.001556 | 0.888044 |
|  | rs34588335 | 3 | 49407660 | 1.34E-22 | 0.013712 | 0.610968 | 1.11E-05 | 0.001327 | 0.938193 |
|  | rs35115732 | 3 | 49475155 | 1.64E-22 | 0.011215 | 0.720899 | 1.04E-05 | 0.001414 | 0.916283 |
|  | rs35169793 | 3 | 49423274 | 1.02E-22 | 0.018066 | 0.342427 | 1.13E-05 | 0.001313 | 0.943471 |
|  | rs35261698 | 3 | 49537839 | 1.74E-22 | 0.010626 | 0.753385 | 9.13E-06 | 0.001603 | 0.883364 |
|  | rs35999162 | 3 | 49597230 | 6.35E-22 | 0.002943 | 0.969048 | 8.42E-06 | 0.001732 | 0.871776 |
|  | rs3811697 | 3 | 49590770 | 8E-22 | 0.002342 | 0.981534 | 9.44E-06 | 0.001552 | 0.89115 |
|  | rs3811699 | 3 | 49396360 | 1.26E-22 | 0.01455 | 0.583358 | 1.05E-05 | 0.001397 | 0.92049 |
|  | rs3926569 | 3 | 49558716 | 8.66E-22 | 0.002164 | 0.988156 | 1.09E-05 | 0.001354 | 0.931496 |
|  | rs4625 | 3 | 49572140 | 7.7E-22 | 0.002431 | 0.979191 | 1.15E-05 | 0.001291 | 0.95389 |
|  | rs6446264 | 3 | 49417243 | 1.74E-22 | 0.010596 | 0.763981 | 1.16E-05 | 0.001276 | 0.964161 |
|  | rs6446272 | 3 | 49463287 | 1.12E-22 | 0.016359 | 0.446021 | 1.15E-05 | 0.001288 | 0.956468 |
|  | rs6446277 | 3 | 49473077 | 1.04E-22 | 0.017602 | 0.377972 | 8.44E-06 | 0.001728 | 0.873505 |
|  | rs67216675 | 3 | 49493151 | 8.5E-23 | 0.021532 | 0.231548 | 9.75E-06 | 0.001505 | 0.900272 |
|  | rs67485053 | 3 | 49405164 | 1.32E-22 | 0.013897 | 0.597255 | 1.13E-05 | 0.001305 | 0.948703 |
|  | rs6766131 | 3 | 49538932 | 1.99E-22 | 0.009273 | 0.822025 | 9.09E-06 | 0.00161 | 0.880154 |
|  | rs6766581 | 3 | 49539401 | 2.52E-22 | 0.007353 | 0.880208 | 9.43E-06 | 0.001554 | 0.889598 |
|  | rs6779524 | 3 | 49450449 | 9.74E-23 | 0.018824 | 0.269754 | 1.15E-05 | 0.00129 | 0.95518 |
|  | rs6793308 | 3 | 49424283 | 1.21E-22 | 0.015132 | 0.5244 | 1.13E-05 | 0.001306 | 0.947398 |
|  | rs6797664 | 3 | 49539222 | 2.44E-22 | 0.007589 | 0.872855 | 9.38E-06 | 0.001563 | 0.884927 |
|  | rs6803222 | 3 | 49665976 | 4.67E-22 | 0.003987 | 0.94781 | 2.08E-06 | 0.006613 | 0.808963 |
|  | rs6809216 | 3 | 49412559 | 1.25E-22 | 0.014724 | 0.554084 | 1.13E-05 | 0.001312 | 0.944783 |
|  | rs6997 | 3 | 49453834 | 1.01E-22 | 0.018083 | 0.324361 | 1.15E-05 | 0.001287 | 0.960328 |
|  | rs71324962 | 3 | 49487487 | 2.31E-22 | 0.008001 | 0.857505 | 1.03E-05 | 0.001427 | 0.913455 |
|  | rs7614725 | 3 | 49650395 | 3.38E-22 | 0.005486 | 0.892689 | 2.26E-06 | 0.006098 | 0.82786 |
|  | rs7622302 | 3 | 49547561 | 4.39E-22 | 0.004238 | 0.935498 | 9.59E-06 | 0.001529 | 0.894227 |
|  | rs7623659 | 3 | 49414791 | 1.1E-22 | 0.016683 | 0.429661 | 1.12E-05 | 0.001316 | 0.942158 |
|  | rs7630869 | 3 | 49522543 | 7.64E-23 | 0.02392 | 0.210017 | 9.47E-06 | 0.001548 | 0.892698 |
|  | rs7633271 | 3 | 49555963 | 1.93E-22 | 0.009543 | 0.803267 | 1.08E-05 | 0.001363 | 0.928781 |
|  | rs7646366 | 3 | 49470668 | 1.39E-22 | 0.013281 | 0.624249 | 1.13E-05 | 0.001309 | 0.946091 |
|  | rs7648841 | 3 | 49416825 | 9.45E-23 | 0.019382 | 0.25093 | 1.16E-05 | 0.001278 | 0.962885 |
|  | rs9811982 | 3 | 49624377 | 7.41E-22 | 0.002526 | 0.976761 | 7.29E-06 | 0.001989 | 0.862897 |
|  | rs9814873 | 3 | 49454112 | 1.15E-22 | 0.015924 | 0.478007 | 1.09E-05 | 0.001353 | 0.932849 |
|  | rs9818590 | 3 | 49525096 | 1.5E-22 | 0.012273 | 0.67468 | 9.75E-06 | 0.001506 | 0.898767 |
|  | rs9821311 | 3 | 49543656 | 1.23E-22 | 0.014961 | 0.539361 | 9.76E-06 | 0.001504 | 0.90328 |
|  | rs9824092 | 3 | 49674147 | 4.93E-22 | 0.003777 | 0.955567 | 2.04E-06 | 0.006728 | 0.795629 |
|  | rs9827021 | 3 | 49514764 | 1.61E-22 | 0.011415 | 0.709683 | 1.03E-05 | 0.001434 | 0.910596 |
|  | rs9827708 | 3 | 49649989 | 3.76E-22 | 0.004941 | 0.908483 | 2.27E-06 | 0.006093 | 0.833953 |
|  | rs9833611 | 3 | 49651210 | 4.43E-22 | 0.004202 | 0.9397 | 2.23E-06 | 0.006196 | 0.821762 |
|  | rs9837027 | 3 | 49597013 | 8.2E-22 | 0.002285 | 0.983819 | 8.88E-06 | 0.001647 | 0.878544 |
|  | rs9837341 | 3 | 49664767 | 4.52E-22 | 0.004123 | 0.943823 | 2.08E-06 | 0.006603 | 0.815567 |
|  | rs9841110 | 3 | 49492481 | 1.72E-22 | 0.010706 | 0.742759 | 1.06E-05 | 0.00139 | 0.923272 |
|  | rs9850072 | 3 | 49529482 | 1.87E-22 | 0.009877 | 0.783955 | 9.83E-06 | 0.001494 | 0.904775 |
|  | rs9853683 | 3 | 49512588 | 1.65E-22 | 0.011155 | 0.732053 | 1.07E-05 | 0.001381 | 0.926042 |
|  | rs9858280 | 3 | 49597737 | 9.19E-22 | 0.002042 | 0.990198 | 8.77E-06 | 0.001665 | 0.876897 |
|  | rs9858418 | 3 | 49543288 | 1.89E-22 | 0.009768 | 0.793723 | 9.76E-06 | 0.001504 | 0.901776 |
|  | rs9859556 | 3 | 49455986 | 1.16E-22 | 0.015802 | 0.493808 | 1.09E-05 | 0.00135 | 0.934199 |
|  | rs9862080 | 3 | 49674458 | 4.95E-22 | 0.003763 | 0.95933 | 2.05E-06 | 0.006721 | 0.802351 |
|  | rs9871380 | 3 | 49438221 | 1.02E-22 | 0.017943 | 0.36037 | 1.19E-05 | 0.001246 | 0.965407 |
|  | rs9878943 | 3 | 49434654 | 1.04E-22 | 0.017586 | 0.395558 | 1.15E-05 | 0.001287 | 0.957755 |
|  | rs71615484 | 3 | 49518299 | 6.76E-17 | 0.999996 | 0.999996 | 3.59E-06 | 0.003026 | 0.960037 |
| rs6967776 | rs1017945 | 7 | 1.33E+08 | 2.7E-08 | 0.011511 | 0.732051 | 2.73E-05 | 0.006993 | 0.718463 |
|  | rs10260563 | 7 | 1.33E+08 | 2.12E-08 | 0.01455 | 0.474642 | 2.71E-05 | 0.007057 | 0.711469 |
|  | rs10262548 | 7 | 1.33E+08 | 3.08E-08 | 0.010131 | 0.773214 | 2.65E-05 | 0.007202 | 0.690164 |
|  | rs10267029 | 7 | 1.33E+08 | 1.35E-07 | 0.002419 | 0.968836 | 1.35E-05 | 0.013686 | 0.331399 |
|  | rs10276144 | 7 | 1.33E+08 | 1.41E-08 | 0.021705 | 0.125995 | 3.7E-05 | 0.005253 | 0.877078 |
|  | rs10276325 | 7 | 1.33E+08 | 7.8E-08 | 0.004114 | 0.932593 | 1.97E-05 | 0.009567 | 0.544771 |
|  | rs1038630 | 7 | 1.33E+08 | 1.68E-08 | 0.018256 | 0.221131 | 3.64E-05 | 0.005328 | 0.866518 |
|  | rs10488169 | 7 | 1.33E+08 | 1.17E-07 | 0.002784 | 0.953342 | 1.29E-05 | 0.014292 | 0.289905 |
|  | rs10954417 | 7 | 1.33E+08 | 7.27E-08 | 0.004406 | 0.920085 | 1.74E-05 | 0.010729 | 0.432659 |
|  | rs1149551 | 7 | 1.33E+08 | 1.58E-08 | 0.01939 | 0.145385 | 4.7E-05 | 0.004184 | 0.951999 |
|  | rs1149555 | 7 | 1.33E+08 | 5.4E-08 | 0.005876 | 0.86367 | 3.91E-05 | 0.00498 | 0.892158 |
|  | rs11765450 | 7 | 1.33E+08 | 2.16E-08 | 0.014295 | 0.503421 | 4.67E-05 | 0.004203 | 0.947815 |
|  | rs11766325 | 7 | 1.33E+08 | 2.16E-08 | 0.014269 | 0.51769 | 3.92E-05 | 0.004972 | 0.897131 |
|  | rs11767900 | 7 | 1.33E+08 | 1.19E-07 | 0.002744 | 0.956085 | 1.14E-05 | 0.016107 | 0.184542 |
|  | rs11772444 | 7 | 1.33E+08 | 1.87E-08 | 0.016417 | 0.306342 | 1.38E-05 | 0.01343 | 0.371867 |
|  | rs11772553 | 7 | 1.33E+08 | 2.05E-08 | 0.015075 | 0.415573 | 4.16E-05 | 0.00469 | 0.921206 |
|  | rs11975951 | 7 | 1.33E+08 | 1.01E-07 | 0.003211 | 0.950557 | 1.91E-05 | 0.009853 | 0.515921 |
|  | rs1247708 | 7 | 1.33E+08 | 8.41E-08 | 0.003826 | 0.940439 | 4.96E-05 | 0.003975 | 0.955974 |
|  | rs13238007 | 7 | 1.33E+08 | 3.03E-08 | 0.010289 | 0.752917 | 6.17E-05 | 0.003231 | 0.966163 |
|  | rs1345939 | 7 | 1.33E+08 | 1.76E-08 | 0.017468 | 0.238599 | 4.01E-05 | 0.004866 | 0.906964 |
|  | rs1364364 | 7 | 1.33E+08 | 1.72E-07 | 0.001919 | 0.983321 | 1.94E-05 | 0.009689 | 0.52561 |
|  | rs1364503 | 7 | 1.33E+08 | 1.18E-08 | 0.025737 | 0.057217 | 4.29E-05 | 0.004557 | 0.935003 |
|  | rs1424580 | 7 | 1.33E+08 | 9.58E-09 | 0.03148 | 0.03148 | 2.74E-05 | 0.006989 | 0.725451 |
|  | rs1628880 | 7 | 1.33E+08 | 7.6E-08 | 0.00422 | 0.924304 | 4.2E-05 | 0.004654 | 0.925859 |
|  | rs17167170 | 7 | 1.33E+08 | 1.62E-08 | 0.018947 | 0.202875 | 1.23E-05 | 0.015026 | 0.23145 |
|  | rs17576069 | 7 | 1.33E+08 | 7.99E-08 | 0.00402 | 0.936613 | 4.46E-05 | 0.004393 | 0.939396 |
|  | rs17597439 | 7 | 1.33E+08 | 3.07E-08 | 0.010166 | 0.763083 | 3.34E-05 | 0.005782 | 0.827523 |
|  | rs17654334 | 7 | 1.33E+08 | 1.33E-08 | 0.022927 | 0.104291 | 0.000041 | 0.004759 | 0.916516 |
|  | rs17656452 | 7 | 1.33E+08 | 4.7E-08 | 0.006723 | 0.813353 | 2.64E-05 | 0.00722 | 0.675751 |
|  | rs17658042 | 7 | 1.33E+08 | 2.66E-08 | 0.011679 | 0.70897 | 1.86E-05 | 0.010085 | 0.506068 |
|  | rs1862871 | 7 | 1.33E+08 | 7.69E-08 | 0.004174 | 0.928479 | 2.24E-05 | 0.008443 | 0.599596 |
|  | rs1946301 | 7 | 1.33E+08 | 2.61E-08 | 0.011879 | 0.68543 | 2.51E-05 | 0.007592 | 0.646514 |
|  | rs1946303 | 7 | 1.33E+08 | 4.01E-08 | 0.007849 | 0.798787 | 3.39E-05 | 0.005701 | 0.838965 |
|  | rs1978507 | 7 | 1.33E+08 | 5.08E-08 | 0.006236 | 0.845605 | 1.97E-05 | 0.009567 | 0.554338 |
|  | rs2012240 | 7 | 1.33E+08 | 6.08E-08 | 0.005243 | 0.885874 | 1.81E-05 | 0.010366 | 0.495983 |
|  | rs2059375 | 7 | 1.33E+08 | 2.01E-08 | 0.015367 | 0.369957 | 3.1E-05 | 0.006204 | 0.80403 |
|  | rs2059376 | 7 | 1.33E+08 | 2.13E-08 | 0.014484 | 0.489126 | 3.01E-05 | 0.006374 | 0.785257 |
|  | rs2113565 | 7 | 1.33E+08 | 4.86E-08 | 0.006504 | 0.82656 | 2.85E-05 | 0.006714 | 0.752615 |
|  | rs2345667 | 7 | 1.33E+08 | 1.71E-07 | 0.001929 | 0.979477 | 9.8E-06 | 0.018595 | 0.114505 |
|  | rs2346264 | 7 | 1.34E+08 | 1.77E-07 | 0.001861 | 0.985182 | 0.000101 | 0.002026 | 0.97043 |
|  | rs2432637 | 7 | 1.33E+08 | 1.29E-07 | 0.002531 | 0.96393 | 1.25E-05 | 0.014728 | 0.26103 |
|  | rs2432641 | 7 | 1.33E+08 | 1.26E-07 | 0.002585 | 0.961398 | 1.36E-05 | 0.01359 | 0.344988 |
|  | rs2911496 | 7 | 1.33E+08 | 1.31E-07 | 0.002488 | 0.966418 | 1.14E-05 | 0.016093 | 0.200635 |
|  | rs2971970 | 7 | 1.34E+08 | 2.51E-08 | 0.012342 | 0.661393 | 0.000122 | 0.001694 | 0.9777 |
|  | rs2991226 | 7 | 1.33E+08 | 1.01E-07 | 0.00322 | 0.947346 | 1.35E-05 | 0.013724 | 0.317713 |
|  | rs2991227 | 7 | 1.33E+08 | 1.19E-07 | 0.002728 | 0.958813 | 1.78E-05 | 0.010516 | 0.485617 |
|  | rs3919590 | 7 | 1.33E+08 | 2.11E-08 | 0.014624 | 0.460092 | 2.94E-05 | 0.006533 | 0.772449 |
|  | rs4363141 | 7 | 1.33E+08 | 6.14E-08 | 0.005191 | 0.896265 | 1.97E-05 | 0.009539 | 0.563877 |
|  | rs4377904 | 7 | 1.33E+08 | 6.75E-08 | 0.004732 | 0.911108 | 1.56E-05 | 0.011946 | 0.42193 |
|  | rs4596592 | 7 | 1.33E+08 | 2.18E-08 | 0.014168 | 0.546025 | 3.66E-05 | 0.005307 | 0.871825 |
|  | rs4728284 | 7 | 1.33E+08 | 2.08E-08 | 0.014835 | 0.445469 | 3.4E-05 | 0.005688 | 0.844653 |
|  | rs4728285 | 7 | 1.33E+08 | 6.46E-08 | 0.004939 | 0.906375 | 2.61E-05 | 0.007306 | 0.661243 |
|  | rs4728287 | 7 | 1.33E+08 | 1.59E-08 | 0.019259 | 0.183928 | 2.77E-05 | 0.006916 | 0.732368 |
|  | rs4728288 | 7 | 1.33E+08 | 1.76E-08 | 0.017411 | 0.25601 | 3.04E-05 | 0.006317 | 0.791573 |
|  | rs4728294 | 7 | 1.33E+08 | 5.21E-08 | 0.006091 | 0.857793 | 1.49E-05 | 0.012505 | 0.397746 |
|  | rs4728298 | 7 | 1.33E+08 | 4.88E-08 | 0.006485 | 0.833044 | 4.66E-05 | 0.004216 | 0.943612 |
|  | rs4731951 | 7 | 1.33E+08 | 2.34E-08 | 0.013243 | 0.559268 | 3.28E-05 | 0.005877 | 0.821741 |
|  | rs4731952 | 7 | 1.33E+08 | 2.4E-08 | 0.01289 | 0.611424 | 2.46E-05 | 0.007729 | 0.631276 |
|  | rs4731955 | 7 | 1.33E+08 | 2.48E-08 | 0.012511 | 0.636584 | 2.9E-05 | 0.006619 | 0.765916 |
|  | rs4731959 | 7 | 1.33E+08 | 4.72E-08 | 0.006702 | 0.820055 | 2.49E-05 | 0.007647 | 0.638923 |
|  | rs4731967 | 7 | 1.33E+08 | 1.43E-07 | 0.002283 | 0.973489 | 1.26E-05 | 0.014583 | 0.275613 |
|  | rs4731968 | 7 | 1.33E+08 | 6.16E-08 | 0.005171 | 0.901436 | 1.96E-05 | 0.009595 | 0.535205 |
|  | rs57352738 | 7 | 1.33E+08 | 4E-08 | 0.007864 | 0.790938 | 1E-05 | 0.018221 | 0.150964 |
|  | rs62469960 | 7 | 1.33E+08 | 1.59E-08 | 0.019283 | 0.164668 | 3.82E-05 | 0.005086 | 0.882164 |
|  | rs62469961 | 7 | 1.33E+08 | 1.81E-08 | 0.016972 | 0.272981 | 4.07E-05 | 0.004792 | 0.911757 |
|  | rs62469963 | 7 | 1.33E+08 | 2.01E-08 | 0.015367 | 0.385323 | 3.26E-05 | 0.005916 | 0.815864 |
|  | rs62469985 | 7 | 1.33E+08 | 8.74E-08 | 0.003687 | 0.944126 | 2.01E-05 | 0.009363 | 0.57324 |
|  | rs62469986 | 7 | 1.33E+08 | 1.71E-07 | 0.001925 | 0.981402 | 1.24E-05 | 0.014852 | 0.246303 |
|  | rs62470027 | 7 | 1.33E+08 | 7E-08 | 0.004571 | 0.915678 | 1.38E-05 | 0.013374 | 0.385241 |
|  | rs62472376 | 7 | 1.33E+08 | 6.13E-08 | 0.0052 | 0.891074 | 9.08E-05 | 0.002241 | 0.968404 |
|  | rs62472378 | 7 | 1.33E+08 | 1.26E-08 | 0.024146 | 0.081364 | 4.26E-05 | 0.004587 | 0.930447 |
|  | rs6467490 | 7 | 1.33E+08 | 2.69E-08 | 0.011569 | 0.720539 | 2.67E-05 | 0.00716 | 0.697325 |
|  | rs6467492 | 7 | 1.33E+08 | 2.18E-08 | 0.014168 | 0.531858 | 3.37E-05 | 0.005741 | 0.833264 |
|  | rs6467495 | 7 | 1.33E+08 | 5.2E-08 | 0.006098 | 0.851702 | 2.35E-05 | 0.008083 | 0.607678 |
|  | rs6949225 | 7 | 1.33E+08 | 5.01E-08 | 0.006324 | 0.839369 | 1.78E-05 | 0.01055 | 0.464573 |
|  | rs6949831 | 7 | 1.33E+08 | 2.36E-08 | 0.013123 | 0.572391 | 5.84E-05 | 0.003401 | 0.962932 |
|  | rs6956399 | 7 | 1.33E+08 | 4.01E-08 | 0.007843 | 0.80663 | 1.38E-05 | 0.013448 | 0.358437 |
|  | rs6961498 | 7 | 1.33E+08 | 1.81E-08 | 0.016944 | 0.289925 | 3.88E-05 | 0.005015 | 0.887179 |
|  | rs6966473 | 7 | 1.33E+08 | 5.7E-08 | 0.005574 | 0.880631 | 1.75E-05 | 0.010711 | 0.44337 |
|  | rs6967240 | 7 | 1.33E+08 | 1.38E-07 | 0.002369 | 0.971206 | 9.21E-06 | 0.019722 | 0.019722 |
|  | rs6967776 | 7 | 1.33E+08 | 1.54E-07 | 0.002126 | 0.975615 | 9.43E-06 | 0.019292 | 0.058585 |
|  | rs6968089 | 7 | 1.33E+08 | 1.92E-07 | 0.001722 | 0.986903 | 9.73E-06 | 0.018729 | 0.077313 |
|  | rs6971417 | 7 | 1.33E+08 | 2.62E-08 | 0.011861 | 0.697291 | 2.99E-05 | 0.006433 | 0.778882 |
|  | rs6974951 | 7 | 1.33E+08 | 2.37E-08 | 0.013064 | 0.598534 | 2.11E-05 | 0.008944 | 0.591153 |
|  | rs6975722 | 7 | 1.33E+08 | 2.45E-08 | 0.01265 | 0.624073 | 2.62E-05 | 0.007288 | 0.668531 |
|  | rs6976440 | 7 | 1.33E+08 | 2.49E-08 | 0.012467 | 0.649051 | 2.65E-05 | 0.007212 | 0.682963 |
|  | rs6979546 | 7 | 1.33E+08 | 1.89E-08 | 0.01629 | 0.322633 | 2.7E-05 | 0.007087 | 0.704412 |
|  | rs718656 | 7 | 1.33E+08 | 2.03E-08 | 0.015175 | 0.400499 | 2.85E-05 | 0.006734 | 0.745901 |
|  | rs73148993 | 7 | 1.33E+08 | 2.05E-08 | 0.01506 | 0.430634 | 3.08E-05 | 0.006252 | 0.797826 |
|  | rs73148998 | 7 | 1.33E+08 | 2.55E-08 | 0.012159 | 0.673551 | 2.82E-05 | 0.0068 | 0.739167 |
|  | rs73150821 | 7 | 1.33E+08 | 1.7E-07 | 0.001933 | 0.977548 | 2.44E-05 | 0.007799 | 0.623547 |
|  | rs7777241 | 7 | 1.33E+08 | 5.66E-08 | 0.005615 | 0.875057 | 1.78E-05 | 0.010527 | 0.475101 |
|  | rs7778778 | 7 | 1.33E+08 | 2.37E-08 | 0.01308 | 0.585471 | 3.44E-05 | 0.00562 | 0.850273 |
|  | rs7779631 | 7 | 1.33E+08 | 3.17E-08 | 0.009861 | 0.783074 | 1.16E-05 | 0.015789 | 0.216425 |
|  | rs7781266 | 7 | 1.34E+08 | 2.08E-07 | 0.001594 | 0.988498 | 0.000108 | 0.001897 | 0.974274 |
|  | rs7786489 | 7 | 1.33E+08 | 1.91E-08 | 0.016084 | 0.338716 | 2.87E-05 | 0.006682 | 0.759297 |
|  | rs7803514 | 7 | 1.33E+08 | 5.5E-08 | 0.005773 | 0.869442 | 2.35E-05 | 0.00807 | 0.615748 |
|  | rs889824 | 7 | 1.34E+08 | 2.2E-07 | 0.001511 | 0.990009 | 0.000208 | 0.001025 | 0.982528 |
|  | rs958408 | 7 | 1.33E+08 | 2.95E-08 | 0.010577 | 0.742628 | 2.57E-05 | 0.007423 | 0.653937 |
|  | rs9649045 | 7 | 1.33E+08 | 1.94E-08 | 0.015874 | 0.35459 | 3.92E-05 | 0.004967 | 0.902098 |
| rs705696 | rs10876866 | 12 | 56445366 | 6.11E-11 | 0.006018 | 0.988293 | 1.86E-05 | 0.026096 | 0.402829 |
|  | rs1689510 | 12 | 56396768 | 6.54E-12 | 0.053616 | 0.611788 | 3.34E-05 | 0.01498 | 0.799553 |
|  | rs1701704 | 12 | 56412487 | 2.13E-12 | 0.161447 | 0.377898 | 1.66E-05 | 0.029143 | 0.292962 |
|  | rs1702877 | 12 | 56427808 | 1.64E-11 | 0.021749 | 0.922994 | 1.67E-05 | 0.028944 | 0.321906 |
|  | rs2292239 | 12 | 56482180 | 1.89E-11 | 0.019011 | 0.942004 | 1.41E-05 | 0.034004 | 0.174734 |
|  | rs2456973 | 12 | 56416928 | 1.58E-12 | 0.216452 | 0.216452 | 1.69E-05 | 0.028731 | 0.350637 |
|  | rs2640562 | 12 | 56466473 | 4.94E-11 | 0.007398 | 0.976215 | 1.04E-05 | 0.045653 | 0.106586 |
|  | rs2640564 | 12 | 56455294 | 7.1E-12 | 0.049448 | 0.763286 | 2.86E-05 | 0.017364 | 0.73792 |
|  | rs34415530 | 12 | 56444632 | 7.98E-12 | 0.044128 | 0.901245 | 2.01E-05 | 0.024345 | 0.527243 |
|  | rs3741499 | 12 | 56474379 | 6.06E-11 | 0.006059 | 0.982274 | 2.01E-05 | 0.024299 | 0.551542 |
|  | rs4759229 | 12 | 56474480 | 6.14E-11 | 0.005988 | 0.994281 | 1.86E-05 | 0.026096 | 0.376733 |
|  | rs61938962 | 12 | 56446766 | 6.74E-12 | 0.052071 | 0.663859 | 1.96E-05 | 0.024926 | 0.502898 |
|  | rs61938963 | 12 | 56446804 | 7.28E-12 | 0.048263 | 0.811549 | 1.95E-05 | 0.024999 | 0.477972 |
|  | rs705696 | 12 | 56480648 | 2.5E-11 | 0.014431 | 0.956435 | 7.67E-06 | 0.060933 | 0.060933 |
|  | rs705704 | 12 | 56435412 | 7.72E-12 | 0.045567 | 0.857116 | 1.94E-05 | 0.025084 | 0.427914 |
|  | rs705705 | 12 | 56435504 | 4.46E-12 | 0.078037 | 0.558172 | 1.62E-05 | 0.029846 | 0.20458 |
|  | rs7302200 | 12 | 56449435 | 7.03E-12 | 0.049979 | 0.713838 | 2.28E-05 | 0.021577 | 0.640984 |
|  | rs772921 | 12 | 56403577 | 3.39E-12 | 0.102237 | 0.480135 | 2.34E-05 | 0.021058 | 0.662042 |
|  | rs877636 | 12 | 56480583 | 2.92E-11 | 0.012382 | 0.968817 | 1.64E-05 | 0.029551 | 0.263819 |
| rs73073015 | rs73073015 | 3 | 49503166 | 9.32E-08 | 0.00088 | 0.987677 | 3.24E-13 | 0.020989 | 0.491697 |
| rs9821797 | rs111881466 | 3 | 48729460 | 9.07E-08 | 0.003619 | 0.893692 | 1.59E-12 | 0.000398 | 0.989166 |
|  | rs112928349 | 3 | 48714335 | 5.05E-08 | 0.006382 | 0.825361 | 4.15E-14 | 0.014244 | 0.826992 |
|  | rs11552724 | 3 | 49156473 | 8.59E-07 | 0.000413 | 0.990302 | 7.55E-13 | 0.000823 | 0.972009 |
|  | rs11557629 | 3 | 48732223 | 1.01E-07 | 0.003263 | 0.906915 | 1.59E-12 | 0.000397 | 0.989563 |
|  | rs11706189 | 3 | 49210833 | 7.48E-07 | 0.000472 | 0.988573 | 9.95E-13 | 0.000628 | 0.983261 |
|  | rs11719291 | 3 | 48735706 | 7.97E-08 | 0.004105 | 0.859427 | 1.31E-12 | 0.00048 | 0.987055 |
|  | rs12107252 | 3 | 48691316 | 3.31E-07 | 0.001036 | 0.979868 | 1.71E-14 | 0.034024 | 0.722372 |
|  | rs12107418 | 3 | 48689787 | 5.26E-07 | 0.000662 | 0.984712 | 1.9E-14 | 0.030644 | 0.753016 |
|  | rs12715429 | 3 | 48709246 | 5.59E-08 | 0.005782 | 0.831143 | 5.1E-14 | 0.011624 | 0.902792 |
|  | rs13070798 | 3 | 48705934 | 6.04E-08 | 0.005369 | 0.836512 | 5.06E-14 | 0.011717 | 0.891167 |
|  | rs13096406 | 3 | 49209058 | 5.41E-07 | 0.000645 | 0.985356 | 9.73E-13 | 0.000642 | 0.982004 |
|  | rs13324119 | 3 | 48710606 | 8.24E-08 | 0.003975 | 0.871432 | 4.57E-14 | 0.012938 | 0.866728 |
|  | rs13353481 | 3 | 48709463 | 7.86E-08 | 0.004161 | 0.855321 | 4E-14 | 0.014755 | 0.812748 |
|  | rs13434258 | 3 | 48738491 | 1.25E-07 | 0.002663 | 0.94124 | 1.61E-12 | 0.000391 | 0.990347 |
|  | rs2286651 | 3 | 48694147 | 1.62E-07 | 0.002064 | 0.952707 | 8.05E-14 | 0.007424 | 0.919976 |
|  | rs2286652 | 3 | 48689192 | 1.75E-07 | 0.001915 | 0.956672 | 9.46E-14 | 0.006335 | 0.939663 |
|  | rs2302295 | 3 | 48690110 | 1.28E-07 | 0.00259 | 0.946448 | 8.69E-14 | 0.006883 | 0.926858 |
|  | rs28452701 | 3 | 48708575 | 8.83E-08 | 0.003715 | 0.886436 | 4.65E-14 | 0.012722 | 0.87945 |
|  | rs28567949 | 3 | 48744560 | 1.22E-07 | 0.002726 | 0.925134 | 1.57E-12 | 0.000401 | 0.988768 |
|  | rs34096717 | 3 | 48719453 | 6.23E-08 | 0.005208 | 0.84172 | 3.52E-14 | 0.01674 | 0.797993 |
|  | rs34571182 | 3 | 49209057 | 5.42E-07 | 0.000643 | 0.985999 | 9.71E-13 | 0.000643 | 0.981362 |
|  | rs3733086 | 3 | 48699519 | 8.56E-08 | 0.003829 | 0.875261 | 9.25E-14 | 0.00647 | 0.933328 |
|  | rs3821875 | 3 | 48697654 | 1.85E-07 | 0.001816 | 0.958488 | 6.09E-14 | 0.00976 | 0.912552 |
|  | rs6768448 | 3 | 48714165 | 2.26E-07 | 0.001497 | 0.968416 | 8.99E-15 | 0.06407 | 0.507585 |
|  | rs9809222 | 3 | 48714411 | 2.3E-07 | 0.00147 | 0.969885 | 8.86E-15 | 0.065002 | 0.443515 |
|  | rs9811027 | 3 | 48725014 | 1.22E-07 | 0.002711 | 0.930556 | 1.35E-12 | 0.000464 | 0.987519 |
|  | rs9811318 | 3 | 48702606 | 3.35E-07 | 0.001024 | 0.980893 | 9.7E-15 | 0.05946 | 0.688348 |
|  | rs9812200 | 3 | 48695667 | 4.26E-07 | 0.000812 | 0.982624 | 2.07E-14 | 0.028237 | 0.781254 |
|  | rs9812977 | 3 | 48720303 | 3.74E-07 | 0.00092 | 0.981813 | 8.57E-15 | 0.067158 | 0.378513 |
|  | rs9821797 | 3 | 48718253 | 2.4E-07 | 0.001415 | 0.9713 | 6.99E-15 | 0.082136 | 0.082136 |
|  | rs9836462 | 3 | 48712791 | 2.55E-07 | 0.001333 | 0.973988 | 9.56E-15 | 0.060347 | 0.628888 |
|  | rs9837237 | 3 | 49215583 | 6.96E-07 | 0.000506 | 0.988101 | 1.24E-12 | 0.000505 | 0.986077 |
|  | rs9837625 | 3 | 49215966 | 6.11E-07 | 0.000573 | 0.986573 | 1.01E-12 | 0.000617 | 0.983878 |
|  | rs9841602 | 3 | 48713570 | 1.89E-07 | 0.001783 | 0.962063 | 9.46E-15 | 0.060956 | 0.568541 |
|  | rs9858236 | 3 | 48709529 | 8.79E-08 | 0.003734 | 0.878995 | 4.56E-14 | 0.012969 | 0.853791 |
|  | rs9862483 | 3 | 49195441 | 4.81E-07 | 0.000722 | 0.983347 | 8E-13 | 0.000778 | 0.975176 |
|  | rs9873726 | 3 | 48712367 | 2.56E-07 | 0.001328 | 0.975316 | 7.88E-15 | 0.072958 | 0.311355 |
|  | rs9877501 | 3 | 48718390 | 2.12E-07 | 0.001596 | 0.965405 | 7.66E-15 | 0.075029 | 0.238398 |
|  | rs9877794 | 3 | 48718388 | 2.68E-07 | 0.001269 | 0.976585 | 7.07E-15 | 0.081233 | 0.163369 |
|  | rs9883469 | 3 | 48719637 | 1.64E-08 | 0.019069 | 0.73288 | 1.08E-12 | 0.00058 | 0.985049 |
|  | rs111742464 | 3 | 50001393 | 2.01E-05 | 0.002447 | 0.988921 | 5.67E-13 | 0.110352 | 0.780345 |
|  | rs112101327 | 3 | 49813826 | 6.7E-07 | 0.063644 | 0.723875 | 1.33E-11 | 0.005 | 0.984731 |
|  | rs34823813 | 3 | 49749976 | 6.44E-07 | 0.066109 | 0.660231 | 4.04E-12 | 0.01607 | 0.968425 |
|  | rs73077200 | 3 | 49826987 | 1.12E-05 | 0.004274 | 0.969422 | 2.77E-13 | 0.223109 | 0.223109 |
|  | rs73079012 | 3 | 49861068 | 7.19E-06 | 0.006514 | 0.96078 | 4.05E-13 | 0.153378 | 0.540691 |
|  | rs73079018 | 3 | 49875327 | 7.71E-07 | 0.055611 | 0.779486 | 1.34E-11 | 0.004963 | 0.989694 |
|  | rs73080977 | 3 | 50189531 | 1.57E-05 | 0.003099 | 0.983784 | 1.31E-12 | 0.048428 | 0.906788 |
|  | rs9853352 | 3 | 49813258 | 6.13E-07 | 0.069329 | 0.52576 | 1.16E-11 | 0.005716 | 0.974142 |
|  | rs9858428 | 3 | 49877585 | 4.11E-07 | 0.101892 | 0.282649 | 1.19E-11 | 0.005589 | 0.979731 |

Abbreviations: probNorm refers to the normalized posterior p-value for each SNP. cumSum means the cumulative normalized posterior p-value for SNP sets. For each sentinel SNP, a 99% credible set of potentially causal SNPs was obtained using the FM-summary method. Each SNP has a corresponding posterior probability, i.e., probNorm, and with each superimposition of another SNP, there is a subsequent cumSum, which is obtained by the the corresponding probNorm of each SNP is summed up. Ultimately, this can be interpreted to mean that there is a 99% probability that the causal SNPs that actually have an effect on the two traits are contained in such a credible set.

## Supplementary Table 6. Fine-mapping 99% credible-set of sentinel SNP from cross-trait meta-analysis between educational attainment (EA) and autism spectrum disorders (ASD)

| Sentinel.SNP | Credible-set SNPs | CHR | BP | EA | | | ASD | | |
| --- | --- | --- | --- | --- | --- | --- | --- | --- | --- |
|  |  |  |  | **GWAS-p-value** | **probNorm** | **cumSum** | **GWAS-p-value** | **probNorm** | **cumSum** |
| rs1106761 | rs10088132 | 8 | 1.43E+08 | 1.83E-09 | 0.004143 | 0.991086 | 0.0003 | 0.002065 | 0.982232 |
|  | rs1078141 | 8 | 1.43E+08 | 2.06E-10 | 0.034849 | 0.860863 | 8.84E-06 | 0.058072 | 0.617297 |
|  | rs1106761 | 8 | 1.43E+08 | 4.08E-11 | 0.170201 | 0.170201 | 7.16E-06 | 0.071087 | 0.365716 |
|  | rs11775333 | 8 | 1.43E+08 | 5.2E-11 | 0.134065 | 0.473694 | 1.87E-05 | 0.028436 | 0.898351 |
|  | rs11782074 | 8 | 1.43E+08 | 6.77E-11 | 0.103565 | 0.577259 | 9.27E-06 | 0.055513 | 0.729267 |
|  | rs11782215 | 8 | 1.43E+08 | 1.77E-10 | 0.040405 | 0.826014 | 1.7E-05 | 0.031176 | 0.839016 |
|  | rs11782665 | 8 | 1.43E+08 | 4.31E-10 | 0.016976 | 0.95098 | 8.14E-06 | 0.062893 | 0.559225 |
|  | rs11787216 | 8 | 1.43E+08 | 6.12E-10 | 0.012054 | 0.978395 | 2.59E-06 | 0.188465 | 0.188465 |
|  | rs12549680 | 8 | 1.43E+08 | 2.62E-10 | 0.027606 | 0.888469 | 1.62E-05 | 0.032664 | 0.80784 |
|  | rs13266268 | 8 | 1.43E+08 | 4.1E-11 | 0.169428 | 0.339628 | 7.72E-06 | 0.066146 | 0.431862 |
|  | rs1566085 | 8 | 1.43E+08 | 1.18E-10 | 0.060234 | 0.637492 | 0.000104 | 0.005609 | 0.980167 |
|  | rs2379268 | 8 | 1.43E+08 | 1.37E-10 | 0.051864 | 0.743095 | 1.71E-05 | 0.030898 | 0.869914 |
|  | rs28744600 | 8 | 1.43E+08 | 3.05E-10 | 0.023807 | 0.912276 | 1.13E-05 | 0.045909 | 0.775176 |
|  | rs35981568 | 8 | 1.43E+08 | 4.78E-10 | 0.015361 | 0.966341 | 4.83E-05 | 0.011546 | 0.965435 |
|  | rs7357604 | 8 | 1.43E+08 | 8.71E-10 | 0.008548 | 0.986944 | 7.93E-06 | 0.06447 | 0.496332 |
|  | rs746839 | 8 | 1.43E+08 | 1.33E-10 | 0.053738 | 0.691231 | 4.71E-06 | 0.106164 | 0.294628 |
|  | rs7846428 | 8 | 1.43E+08 | 1.68E-10 | 0.042514 | 0.785609 | 2.32E-05 | 0.023147 | 0.921498 |
|  | rs903959 | 8 | 1.43E+08 | 3.35E-10 | 0.021728 | 0.934004 | 9.11E-06 | 0.056456 | 0.673754 |
| rs62057121 | rs10445362 | 17 | 43914554 | 1.32E-07 | 0.003463 | 0.820335 | 6.64E-06 | 0.000504 | 0.219706 |
|  | rs10445363 | 17 | 43914558 | 1.17E-07 | 0.003907 | 0.758133 | 6.39E-06 | 0.000523 | 0.19005 |
|  | rs111321973 | 17 | 43953170 | 4.26E-07 | 0.001121 | 0.972498 | 7.61E-06 | 0.000442 | 0.484802 |
|  | rs111327992 | 17 | 44126691 | 3.45E-07 | 0.001374 | 0.963715 | 8.81E-06 | 0.000384 | 0.822876 |
|  | rs111370985 | 17 | 43852742 | 1.76E-07 | 0.002634 | 0.91685 | 8.46E-06 | 0.000399 | 0.735182 |
|  | rs111372048 | 17 | 44136577 | 3.63E-07 | 0.001308 | 0.96765 | 8.37E-06 | 0.000404 | 0.710304 |
|  | rs111415173 | 17 | 43795573 | 5.92E-07 | 0.000816 | 0.982391 | 7.69E-06 | 0.000438 | 0.513831 |
|  | rs111433752 | 17 | 43857989 | 3.63E-07 | 0.001309 | 0.966342 | 7.15E-06 | 0.000469 | 0.337748 |
|  | rs111519055 | 17 | 44159672 | 5.48E-07 | 0.000878 | 0.979047 | 8.25E-06 | 0.00041 | 0.666188 |
|  | rs111541901 | 17 | 43994358 | 1.5E-07 | 0.003068 | 0.908517 | 7.12E-06 | 0.000471 | 0.324585 |
|  | rs111664122 | 17 | 43857990 | 3.6E-07 | 0.001319 | 0.965033 | 7.15E-06 | 0.000469 | 0.337278 |
|  | rs111735741 | 17 | 43856372 | 6.78E-08 | 0.006623 | 0.43011 | 8.45E-06 | 0.0004 | 0.731184 |
|  | rs111739681 | 17 | 43898459 | 8.71E-08 | 0.005193 | 0.630091 | 5.81E-06 | 0.000573 | 0.138352 |
|  | rs111751251 | 17 | 44042951 | 2.35E-07 | 0.001992 | 0.94724 | 7.1E-06 | 0.000473 | 0.318926 |
|  | rs111878933 | 17 | 43856341 | 1.9E-07 | 0.002447 | 0.932104 | 8.45E-06 | 0.0004 | 0.730784 |
|  | rs111913701 | 17 | 44159631 | 5.32E-07 | 0.000903 | 0.978169 | 8.25E-06 | 0.00041 | 0.670283 |
|  | rs111962225 | 17 | 43934016 | 1.48E-07 | 0.00312 | 0.899255 | 6.94E-06 | 0.000483 | 0.274079 |
|  | rs111970616 | 17 | 44169581 | 6.04E-07 | 0.0008 | 0.984804 | 7.46E-06 | 0.000451 | 0.448267 |
|  | rs111985258 | 17 | 43795859 | 6E-07 | 0.000805 | 0.984003 | 7.7E-06 | 0.000437 | 0.52958 |
|  | rs112003140 | 17 | 43980998 | 1.48E-07 | 0.003106 | 0.90236 | 7.87E-06 | 0.000428 | 0.576295 |
|  | rs112003311 | 17 | 44042939 | 5.19E-07 | 0.000926 | 0.977265 | 7.54E-06 | 0.000446 | 0.462161 |
|  | rs112166495 | 17 | 44005361 | 1.19E-07 | 0.003849 | 0.773629 | 8.59E-06 | 0.000394 | 0.776539 |
|  | rs112197756 | 17 | 44154105 | 5.76E-07 | 0.000837 | 0.980752 | 8.25E-06 | 0.00041 | 0.662092 |
|  | rs112275277 | 17 | 43981958 | 7.13E-08 | 0.006303 | 0.449577 | 7.87E-06 | 0.000428 | 0.57244 |
|  | rs112333322 | 17 | 44126673 | 3.3E-07 | 0.001435 | 0.96234 | 8.81E-06 | 0.000384 | 0.833639 |
|  | rs112364920 | 17 | 44161360 | 5.09E-07 | 0.000942 | 0.975407 | 8.21E-06 | 0.000411 | 0.647328 |
|  | rs112385572 | 17 | 44066172 | 9.51E-08 | 0.004771 | 0.684642 | 8.61E-06 | 0.000393 | 0.782437 |
|  | rs112439933 | 17 | 44005226 | 1.25E-07 | 0.003676 | 0.784869 | 8.73E-06 | 0.000388 | 0.806288 |
|  | rs112454267 | 17 | 44005329 | 1.19E-07 | 0.00384 | 0.777469 | 8.72E-06 | 0.000388 | 0.805123 |
|  | rs112570965 | 17 | 43801092 | 5.98E-07 | 0.000807 | 0.983198 | 7.82E-06 | 0.000431 | 0.559539 |
|  | rs112572874 | 17 | 44072984 | 3.18E-07 | 0.001484 | 0.959422 | 8.42E-06 | 0.000401 | 0.722373 |
|  | rs112578465 | 17 | 44125066 | 2.65E-07 | 0.001771 | 0.954772 | 9.32E-06 | 0.000364 | 0.893814 |
|  | rs112583797 | 17 | 43918418 | 8.18E-08 | 0.005523 | 0.560639 | 6.66E-06 | 0.000503 | 0.22373 |
|  | rs112596352 | 17 | 44170238 | 3.67E-07 | 0.001293 | 0.968943 | 7.91E-06 | 0.000426 | 0.590821 |
|  | rs112647192 | 17 | 43994623 | 8.11E-08 | 0.005569 | 0.555116 | 7.12E-06 | 0.000471 | 0.324114 |
|  | rs112746008 | 17 | 44126650 | 6.93E-07 | 0.0007 | 0.986975 | 8.81E-06 | 0.000384 | 0.822492 |
|  | rs112995313 | 17 | 43795768 | 7.11E-07 | 0.000684 | 0.987659 | 7.7E-06 | 0.000437 | 0.519959 |
|  | rs113029914 | 17 | 43981831 | 3.03E-07 | 0.001554 | 0.957938 | 7.87E-06 | 0.000428 | 0.57501 |
|  | rs113093579 | 17 | 43934116 | 1.82E-07 | 0.002553 | 0.927195 | 7.6E-06 | 0.000443 | 0.47772 |
|  | rs113161176 | 17 | 43974354 | 9.06E-08 | 0.005002 | 0.655505 | 8.19E-06 | 0.000412 | 0.646092 |
|  | rs113313477 | 17 | 44005186 | 1.14E-07 | 0.003993 | 0.750297 | 8.72E-06 | 0.000388 | 0.8059 |
|  | rs113414067 | 17 | 43955093 | 1.37E-07 | 0.003358 | 0.837351 | 7.73E-06 | 0.000436 | 0.536124 |
|  | rs113520245 | 17 | 44033132 | 3.18E-07 | 0.001484 | 0.960906 | 7.74E-06 | 0.000435 | 0.538301 |
|  | rs113589236 | 17 | 43981795 | 8.36E-08 | 0.005404 | 0.576872 | 7.87E-06 | 0.000428 | 0.57758 |
|  | rs113661667 | 17 | 43791610 | 7.4E-07 | 0.000658 | 0.988986 | 7.7E-06 | 0.000437 | 0.520834 |
|  | rs113788190 | 17 | 44161302 | 5.15E-07 | 0.000933 | 0.976339 | 8.09E-06 | 0.000417 | 0.621663 |
|  | rs113796169 | 17 | 44005254 | 1.23E-07 | 0.003725 | 0.781194 | 8.72E-06 | 0.000388 | 0.805512 |
|  | rs113856644 | 17 | 43932277 | 1.04E-07 | 0.004395 | 0.716567 | 7.56E-06 | 0.000445 | 0.467951 |
|  | rs113871181 | 17 | 43852733 | 1.77E-07 | 0.002618 | 0.919468 | 8.47E-06 | 0.000399 | 0.738776 |
|  | rs113925422 | 17 | 44046934 | 1.02E-07 | 0.004454 | 0.707735 | 7.27E-06 | 0.000462 | 0.37494 |
|  | rs113991678 | 17 | 43795634 | 6.33E-07 | 0.000764 | 0.985568 | 8.53E-06 | 0.000396 | 0.770618 |
|  | rs117124984 | 17 | 44051588 | 1.39E-07 | 0.003311 | 0.860654 | 8.63E-06 | 0.000392 | 0.785972 |
|  | rs117365970 | 17 | 43893259 | 3.98E-08 | 0.011097 | 0.064765 | 6.39E-06 | 0.000523 | 0.190573 |
|  | rs117646503 | 17 | 43893260 | 4.43E-08 | 0.009995 | 0.0853 | 6.41E-06 | 0.000521 | 0.193704 |
|  | rs118087478 | 17 | 44051589 | 1.28E-07 | 0.003576 | 0.799326 | 8.63E-06 | 0.000392 | 0.787933 |
|  | rs12150390 | 17 | 43896228 | 5.5E-08 | 0.008111 | 0.254873 | 5.85E-06 | 0.000569 | 0.142343 |
|  | rs1396862 | 17 | 43902997 | 6.65E-08 | 0.006745 | 0.416845 | 6.32E-06 | 0.000528 | 0.183211 |
|  | rs140713557 | 17 | 43953179 | 3.88E-07 | 0.001225 | 0.970167 | 6.96E-06 | 0.000482 | 0.276972 |
|  | rs16940665 | 17 | 43907896 | 1.38E-07 | 0.00332 | 0.850714 | 6.48E-06 | 0.000516 | 0.198883 |
|  | rs16940668 | 17 | 43907966 | 1.44E-07 | 0.003189 | 0.889802 | 6.63E-06 | 0.000504 | 0.215672 |
|  | rs16940671 | 17 | 43908151 | 5.82E-08 | 0.007676 | 0.30171 | 6.63E-06 | 0.000504 | 0.217185 |
|  | rs16940672 | 17 | 43908152 | 3.78E-08 | 0.011651 | 0.053668 | 6.63E-06 | 0.000504 | 0.216176 |
|  | rs16940674 | 17 | 43910507 | 1.36E-07 | 0.00337 | 0.833993 | 6.34E-06 | 0.000527 | 0.186903 |
|  | rs16940676 | 17 | 43911036 | 1.35E-07 | 0.003394 | 0.830623 | 5.73E-06 | 0.00058 | 0.130884 |
|  | rs16940677 | 17 | 43911898 | 1.37E-07 | 0.003346 | 0.84405 | 5.59E-06 | 0.000594 | 0.126802 |
|  | rs16940681 | 17 | 43912159 | 1.39E-07 | 0.003313 | 0.857343 | 6.34E-06 | 0.000527 | 0.186376 |
|  | rs17425752 | 17 | 43906726 | 1.1E-07 | 0.004162 | 0.742253 | 8.29E-06 | 0.000408 | 0.699742 |
|  | rs17426174 | 17 | 43830938 | 1.72E-07 | 0.002691 | 0.914215 | 7.51E-06 | 0.000448 | 0.456351 |
|  | rs17689378 | 17 | 43881790 | 7.57E-08 | 0.005953 | 0.480145 | 6.71E-06 | 0.000499 | 0.238268 |
|  | rs17689471 | 17 | 43892973 | 7.68E-08 | 0.005867 | 0.503696 | 6.2E-06 | 0.000538 | 0.173113 |
|  | rs17689608 | 17 | 43896528 | 4.5E-08 | 0.009847 | 0.124853 | 5.85E-06 | 0.000569 | 0.142912 |
|  | rs17689653 | 17 | 43898963 | 5.97E-08 | 0.007495 | 0.309204 | 5.8E-06 | 0.000573 | 0.13778 |
|  | rs17689824 | 17 | 43904397 | 2.25E-07 | 0.002071 | 0.945248 | 5.94E-06 | 0.00056 | 0.161581 |
|  | rs17689882 | 17 | 43906828 | 1.33E-07 | 0.003453 | 0.823788 | 7.83E-06 | 0.00043 | 0.566001 |
|  | rs17689918 | 17 | 43910088 | 1.16E-07 | 0.003929 | 0.754226 | 5.83E-06 | 0.000571 | 0.140066 |
|  | rs17762769 | 17 | 43893403 | 6.84E-08 | 0.006564 | 0.443273 | 5.74E-06 | 0.00058 | 0.131464 |
|  | rs17762882 | 17 | 43898887 | 8.5E-08 | 0.005321 | 0.614463 | 5.8E-06 | 0.000573 | 0.133766 |
|  | rs17762912 | 17 | 43899161 | 6.48E-08 | 0.006918 | 0.396339 | 5.85E-06 | 0.000569 | 0.14462 |
|  | rs17762954 | 17 | 43899786 | 7.77E-08 | 0.005799 | 0.521122 | 5.9E-06 | 0.000564 | 0.154844 |
|  | rs17763050 | 17 | 43903336 | 6.09E-08 | 0.007351 | 0.338778 | 6.32E-06 | 0.000528 | 0.183739 |
|  | rs17763086 | 17 | 43905481 | 7.6E-08 | 0.005929 | 0.486074 | 5.94E-06 | 0.00056 | 0.1599 |
|  | rs17763199 | 17 | 43910183 | 1.44E-07 | 0.003197 | 0.886613 | 6.39E-06 | 0.000522 | 0.191095 |
|  | rs1876827 | 17 | 43911832 | 1.31E-07 | 0.003497 | 0.813393 | 6.34E-06 | 0.000527 | 0.185849 |
|  | rs1876828 | 17 | 43911525 | 1.41E-07 | 0.003261 | 0.870526 | 6.57E-06 | 0.000509 | 0.204525 |
|  | rs1876829 | 17 | 43911443 | 1.32E-07 | 0.003479 | 0.816872 | 6.48E-06 | 0.000516 | 0.199914 |
|  | rs1876830 | 17 | 43911352 | 1.41E-07 | 0.003259 | 0.873784 | 6.34E-06 | 0.000527 | 0.18743 |
|  | rs1876831 | 17 | 43907745 | 1.89E-07 | 0.002461 | 0.929656 | 6.58E-06 | 0.000508 | 0.208084 |
|  | rs1912151 | 17 | 43902944 | 6.33E-08 | 0.007071 | 0.389422 | 6.32E-06 | 0.000528 | 0.184267 |
|  | rs2316763 | 17 | 43895530 | 5.35E-08 | 0.008324 | 0.205707 | 5.85E-06 | 0.000569 | 0.149171 |
|  | rs2316764 | 17 | 43895602 | 4.61E-08 | 0.009615 | 0.14431 | 5.85E-06 | 0.000569 | 0.143481 |
|  | rs2316765 | 17 | 43912454 | 1.3E-07 | 0.00352 | 0.806377 | 6.34E-06 | 0.000527 | 0.185322 |
|  | rs28364021 | 17 | 43912282 | 8.7E-08 | 0.005203 | 0.624898 | 6.1E-06 | 0.000547 | 0.168787 |
|  | rs28364023 | 17 | 43894159 | 2.09E-07 | 0.002229 | 0.936631 | 5.96E-06 | 0.000558 | 0.162698 |
|  | rs28364025 | 17 | 43894102 | 7.25E-08 | 0.006207 | 0.462015 | 6.16E-06 | 0.000541 | 0.170958 |
|  | rs3885074 | 17 | 43902738 | 7.35E-08 | 0.006125 | 0.468139 | 6.27E-06 | 0.000532 | 0.176857 |
|  | rs3885075 | 17 | 43902799 | 2.92E-07 | 0.001611 | 0.956384 | 6.26E-06 | 0.000533 | 0.176324 |
|  | rs41280116 | 17 | 43902842 | 2.19E-07 | 0.002133 | 0.943177 | 6.27E-06 | 0.000532 | 0.177389 |
|  | rs41280118 | 17 | 43903089 | 5.64E-08 | 0.007914 | 0.262787 | 6.32E-06 | 0.000528 | 0.181626 |
|  | rs41457044 | 17 | 43911424 | 1.3E-07 | 0.00352 | 0.809897 | 6.42E-06 | 0.00052 | 0.195786 |
|  | rs4277389 | 17 | 43895653 | 4.89E-08 | 0.009083 | 0.162674 | 5.71E-06 | 0.000582 | 0.129723 |
|  | rs4309444 | 17 | 43895797 | 5.43E-08 | 0.008211 | 0.230447 | 5.85E-06 | 0.000569 | 0.141774 |
|  | rs4335809 | 17 | 43902216 | 8.42E-08 | 0.005365 | 0.603803 | 6.17E-06 | 0.00054 | 0.171498 |
|  | rs4341787 | 17 | 43902505 | 9.82E-08 | 0.004623 | 0.703282 | 7.18E-06 | 0.000467 | 0.344773 |
|  | rs4482334 | 17 | 43912830 | 1.25E-07 | 0.00365 | 0.78852 | 6.6E-06 | 0.000507 | 0.209606 |
|  | rs4523962 | 17 | 43902522 | 1.06E-07 | 0.004303 | 0.733926 | 6.22E-06 | 0.000536 | 0.174723 |
|  | rs4525537 | 17 | 43912723 | 1.17E-07 | 0.003894 | 0.762027 | 6.6E-06 | 0.000507 | 0.210113 |
|  | rs4564621 | 17 | 43895501 | 6.27E-08 | 0.007143 | 0.38235 | 5.85E-06 | 0.000569 | 0.152014 |
|  | rs4566211 | 17 | 43895696 | 5.44E-08 | 0.0082 | 0.238647 | 5.67E-06 | 0.000586 | 0.127975 |
|  | rs4566212 | 17 | 43895751 | 5.35E-08 | 0.008328 | 0.197384 | 5.85E-06 | 0.000569 | 0.151445 |
|  | rs4640231 | 17 | 43912786 | 1.03E-07 | 0.004437 | 0.712172 | 7.14E-06 | 0.00047 | 0.331641 |
|  | rs55668363 | 17 | 43892788 | 6.8E-08 | 0.006599 | 0.436709 | 4.1E-06 | 0.0008 | 0.078322 |
|  | rs55763795 | 17 | 43908773 | 1.42E-07 | 0.003232 | 0.877017 | 6.63E-06 | 0.000504 | 0.216681 |
|  | rs55779147 | 17 | 43894510 | 5.79E-08 | 0.007714 | 0.294034 | 5.92E-06 | 0.000563 | 0.15597 |
|  | rs55825513 | 17 | 44176215 | 7.71E-07 | 0.000633 | 0.989619 | 6.93E-06 | 0.000483 | 0.272631 |
|  | rs55865707 | 17 | 43908826 | 1.37E-07 | 0.003344 | 0.847394 | 6.63E-06 | 0.000505 | 0.214663 |
|  | rs55915917 | 17 | 43892784 | 6.17E-08 | 0.007252 | 0.368003 | 3.55E-06 | 0.000918 | 0.06038 |
|  | rs55943044 | 17 | 43872228 | 4.78E-08 | 0.009281 | 0.153591 | 7.74E-06 | 0.000435 | 0.53743 |
|  | rs55979424 | 17 | 43900081 | 8.47E-08 | 0.005338 | 0.609142 | 6.01E-06 | 0.000554 | 0.164925 |
|  | rs56099546 | 17 | 43894609 | 4.5E-08 | 0.009842 | 0.134695 | 5.8E-06 | 0.000573 | 0.137206 |
|  | rs56127111 | 17 | 43913315 | 2.44E-07 | 0.00192 | 0.951102 | 6.59E-06 | 0.000508 | 0.209099 |
|  | rs56319902 | 17 | 43871982 | 5.14E-08 | 0.008666 | 0.189056 | 7.45E-06 | 0.000452 | 0.446914 |
|  | rs56357543 | 17 | 43894547 | 4.99E-08 | 0.008915 | 0.171589 | 5.92E-06 | 0.000563 | 0.156532 |
|  | rs62054760 | 17 | 43908989 | 1.45E-07 | 0.003168 | 0.89297 | 1.14E-05 | 0.0003 | 0.979093 |
|  | rs62054761 | 17 | 43909008 | 1.44E-07 | 0.0032 | 0.880216 | 1.14E-05 | 0.0003 | 0.979393 |
|  | rs62054762 | 17 | 43909022 | 1.39E-07 | 0.003316 | 0.85403 | 1.14E-05 | 0.0003 | 0.979693 |
|  | rs62054763 | 17 | 43910262 | 1.33E-07 | 0.003441 | 0.827229 | 6.48E-06 | 0.000516 | 0.198368 |
|  | rs62054802 | 17 | 43914598 | 1.39E-07 | 0.003311 | 0.863965 | 6.64E-06 | 0.000504 | 0.219201 |
|  | rs62054803 | 17 | 43914728 | 7.75E-08 | 0.005818 | 0.509514 | 6.64E-06 | 0.000504 | 0.218697 |
|  | rs62054804 | 17 | 43914809 | 8.33E-08 | 0.005424 | 0.566063 | 6.64E-06 | 0.000504 | 0.218193 |
|  | rs62054805 | 17 | 43915054 | 9.77E-08 | 0.004648 | 0.694016 | 6.65E-06 | 0.000503 | 0.221216 |
|  | rs62054835 | 17 | 43934672 | 1.53E-07 | 0.003007 | 0.911525 | 7E-06 | 0.000479 | 0.291841 |
|  | rs62055869 | 17 | 43830685 | 1.79E-07 | 0.002586 | 0.924642 | 7.31E-06 | 0.00046 | 0.390158 |
|  | rs62056785 | 17 | 43975263 | 8.39E-08 | 0.005388 | 0.593057 | 7.95E-06 | 0.000424 | 0.603573 |
|  | rs62056786 | 17 | 43975285 | 1.04E-07 | 0.004363 | 0.725308 | 7.95E-06 | 0.000424 | 0.603149 |
|  | rs62056790 | 17 | 43975417 | 1.18E-07 | 0.003868 | 0.769779 | 7.95E-06 | 0.000424 | 0.602302 |
|  | rs62056851 | 17 | 43992806 | 1.06E-07 | 0.004315 | 0.729623 | 7.49E-06 | 0.000449 | 0.452317 |
|  | rs62057062 | 17 | 43856710 | 9.27E-08 | 0.004889 | 0.670305 | 8.34E-06 | 0.000405 | 0.707066 |
|  | rs62057063 | 17 | 43856730 | 9.01E-08 | 0.005026 | 0.650503 | 8.34E-06 | 0.000405 | 0.707877 |
|  | rs62057064 | 17 | 43857033 | 7.76E-08 | 0.005809 | 0.515324 | 8.28E-06 | 0.000408 | 0.698518 |
|  | rs62057067 | 17 | 43858326 | 9.78E-08 | 0.004643 | 0.698659 | 7.97E-06 | 0.000423 | 0.606536 |
|  | rs62057068 | 17 | 43858629 | 8.36E-08 | 0.005404 | 0.582276 | 7.91E-06 | 0.000426 | 0.592525 |
|  | rs62057069 | 17 | 43859065 | 7.97E-08 | 0.00566 | 0.543919 | 7.79E-06 | 0.000432 | 0.554359 |
|  | rs62057070 | 17 | 43859640 | 9.13E-08 | 0.004965 | 0.66047 | 7.79E-06 | 0.000432 | 0.553494 |
|  | rs62057071 | 17 | 43859691 | 8.02E-08 | 0.005628 | 0.549547 | 7.79E-06 | 0.000432 | 0.553927 |
|  | rs62057073 | 17 | 43861117 | 1.45E-07 | 0.003165 | 0.896135 | 7.74E-06 | 0.000435 | 0.537865 |
|  | rs62057101 | 17 | 43885291 | 8.84E-08 | 0.005122 | 0.640362 | 7.3E-06 | 0.00046 | 0.386479 |
|  | rs62057107 | 17 | 43896032 | 3.04E-08 | 0.014421 | 0.030025 | 5.85E-06 | 0.000569 | 0.150308 |
|  | rs62057108 | 17 | 43896616 | 6.04E-08 | 0.007407 | 0.324029 | 5.85E-06 | 0.000569 | 0.140635 |
|  | rs62057109 | 17 | 43896637 | 6.03E-08 | 0.007418 | 0.316622 | 5.85E-06 | 0.000569 | 0.141205 |
|  | rs62057110 | 17 | 43896734 | 6.1E-08 | 0.00733 | 0.346108 | 5.85E-06 | 0.000569 | 0.147466 |
|  | rs62057111 | 17 | 43897130 | 5.71E-08 | 0.007821 | 0.278504 | 5.8E-06 | 0.000573 | 0.135486 |
|  | rs62057112 | 17 | 43897202 | 6.11E-08 | 0.007318 | 0.360751 | 5.81E-06 | 0.000573 | 0.138925 |
|  | rs62057113 | 17 | 43897449 | 5.39E-08 | 0.008271 | 0.213978 | 5.8E-06 | 0.000573 | 0.13606 |
|  | rs62057114 | 17 | 43899401 | 7.95E-08 | 0.005677 | 0.532585 | 5.85E-06 | 0.000569 | 0.150877 |
|  | rs62057115 | 17 | 43899417 | 8.38E-08 | 0.005392 | 0.587668 | 5.85E-06 | 0.000569 | 0.148034 |
|  | rs62057116 | 17 | 43899655 | 6.21E-08 | 0.007204 | 0.375207 | 5.85E-06 | 0.000569 | 0.146327 |
|  | rs62057117 | 17 | 43899657 | 6.76E-08 | 0.006642 | 0.423487 | 5.85E-06 | 0.000569 | 0.144051 |
|  | rs62057118 | 17 | 43899727 | 9.6E-08 | 0.004726 | 0.689368 | 5.85E-06 | 0.000569 | 0.146896 |
|  | rs62057119 | 17 | 43899736 | 7.79E-08 | 0.005786 | 0.526908 | 5.85E-06 | 0.000569 | 0.14974 |
|  | rs62057121 | 17 | 43900760 | 2.8E-08 | 0.015604 | 0.015604 | 5.94E-06 | 0.00056 | 0.160461 |
|  | rs62057122 | 17 | 43901001 | 8.4E-08 | 0.005382 | 0.598439 | 5.72E-06 | 0.000581 | 0.130304 |
|  | rs62057123 | 17 | 43901238 | 9.51E-08 | 0.004772 | 0.679871 | 5.98E-06 | 0.000557 | 0.164371 |
|  | rs62057143 | 17 | 43901528 | 9.46E-08 | 0.004793 | 0.675099 | 6.02E-06 | 0.000553 | 0.166585 |
|  | rs62057144 | 17 | 43901558 | 1.39E-07 | 0.0033 | 0.867265 | 6.02E-06 | 0.000553 | 0.166032 |
|  | rs62057146 | 17 | 43903106 | 5.05E-08 | 0.0088 | 0.18039 | 6.31E-06 | 0.000529 | 0.18004 |
|  | rs62057147 | 17 | 43903298 | 3.67E-08 | 0.011992 | 0.042017 | 6.52E-06 | 0.000513 | 0.201456 |
|  | rs62057148 | 17 | 43903485 | 6.48E-08 | 0.006918 | 0.403257 | 5.83E-06 | 0.000571 | 0.139496 |
|  | rs62057149 | 17 | 43903546 | 8.85E-08 | 0.005115 | 0.645477 | 6.31E-06 | 0.000529 | 0.180569 |
|  | rs62057150 | 17 | 43903548 | 8.79E-08 | 0.005149 | 0.63524 | 6.32E-06 | 0.000528 | 0.182154 |
|  | rs62057151 | 17 | 43903842 | 7.22E-08 | 0.006231 | 0.455807 | 5.18E-06 | 0.000639 | 0.116313 |
|  | rs62057153 | 17 | 43904528 | 1.26E-07 | 0.003645 | 0.792164 | 6.16E-06 | 0.000541 | 0.170416 |
|  | rs62057154 | 17 | 43904610 | 9.16E-08 | 0.004945 | 0.665416 | 6.42E-06 | 0.000521 | 0.194746 |
|  | rs62057155 | 17 | 43904673 | 8.36E-08 | 0.005405 | 0.571468 | 6.42E-06 | 0.000521 | 0.194225 |
|  | rs62057156 | 17 | 43904948 | 8.64E-08 | 0.005232 | 0.619695 | 6.47E-06 | 0.000517 | 0.196303 |
|  | rs62057157 | 17 | 43905313 | 7.44E-08 | 0.006053 | 0.474192 | 6.04E-06 | 0.000552 | 0.167137 |
|  | rs62057158 | 17 | 43907143 | 1.49E-07 | 0.003089 | 0.90545 | 6.58E-06 | 0.000508 | 0.207576 |
|  | rs62059005 | 17 | 44004472 | 3.93E-07 | 0.00121 | 0.971377 | 6.88E-06 | 0.000487 | 0.262453 |
|  | rs62062277 | 17 | 44093753 | 1.79E-07 | 0.002589 | 0.922057 | 8.48E-06 | 0.000399 | 0.748744 |
|  | rs62062278 | 17 | 44093860 | 2.13E-07 | 0.00219 | 0.941044 | 8.48E-06 | 0.000399 | 0.742766 |
|  | rs62062279 | 17 | 44093964 | 1.28E-07 | 0.003587 | 0.795751 | 8.48E-06 | 0.000399 | 0.742368 |
|  | rs62062280 | 17 | 44093993 | 1.44E-07 | 0.0032 | 0.883416 | 8.49E-06 | 0.000398 | 0.754721 |
|  | rs62063271 | 17 | 44036047 | 2.41E-07 | 0.001942 | 0.949182 | 9.73E-06 | 0.00035 | 0.953735 |
|  | rs62641967 | 17 | 44047216 | 2.47E-07 | 0.001899 | 0.953001 | 8.5E-06 | 0.000398 | 0.758705 |
|  | rs739644 | 17 | 43895008 | 4.48E-08 | 0.0099 | 0.105108 | 5.85E-06 | 0.000569 | 0.145758 |
|  | rs739645 | 17 | 43894990 | 4.48E-08 | 0.009898 | 0.115006 | 5.97E-06 | 0.000558 | 0.163256 |
|  | rs74998289 | 17 | 43913558 | 1.18E-07 | 0.003884 | 0.765911 | 7.23E-06 | 0.000464 | 0.355021 |
|  | rs75104593 | 17 | 43913557 | 1.37E-07 | 0.003353 | 0.840704 | 7.23E-06 | 0.000464 | 0.354556 |
|  | rs75183956 | 17 | 44182956 | 7.81E-07 | 0.000624 | 0.990243 | 5.47E-06 | 0.000606 | 0.125007 |
|  | rs75330746 | 17 | 44182955 | 6.86E-07 | 0.000707 | 0.986275 | 5.43E-06 | 0.000611 | 0.122575 |
|  | rs75530705 | 17 | 44154048 | 5.55E-07 | 0.000868 | 0.979915 | 8.25E-06 | 0.000409 | 0.687475 |
|  | rs76154201 | 17 | 44154033 | 5.87E-07 | 0.000822 | 0.981575 | 8.25E-06 | 0.00041 | 0.664959 |
|  | rs76563578 | 17 | 43933879 | 2.09E-07 | 0.002223 | 0.938854 | 7E-06 | 0.000479 | 0.291362 |
|  | rs76830096 | 17 | 43857129 | 7.66E-08 | 0.005885 | 0.491959 | 8.28E-06 | 0.000408 | 0.699334 |
|  | rs78026984 | 17 | 44025592 | 7.26E-07 | 0.00067 | 0.988328 | 6.66E-06 | 0.000503 | 0.222724 |
|  | rs78074121 | 17 | 43896690 | 6.05E-08 | 0.007397 | 0.331426 | 5.85E-06 | 0.000569 | 0.145189 |
|  | rs78506181 | 17 | 43897480 | 5.4E-08 | 0.008258 | 0.222236 | 5.8E-06 | 0.000573 | 0.136633 |
|  | rs78587102 | 17 | 43897246 | 4.47E-08 | 0.009909 | 0.095208 | 5.8E-06 | 0.000573 | 0.134913 |
|  | rs78872653 | 17 | 43905134 | 7.68E-08 | 0.005869 | 0.497829 | 6.52E-06 | 0.000513 | 0.201969 |
|  | rs78917479 | 17 | 43899611 | 6.11E-08 | 0.007326 | 0.353434 | 5.85E-06 | 0.000569 | 0.148603 |
|  | rs78917495 | 17 | 43858482 | 7.95E-08 | 0.005674 | 0.53826 | 8.2E-06 | 0.000412 | 0.646916 |
|  | rs79501144 | 17 | 43900697 | 1.04E-07 | 0.004379 | 0.720946 | 5.94E-06 | 0.00056 | 0.161021 |
|  | rs79600142 | 17 | 43897722 | 5.65E-08 | 0.007895 | 0.270682 | 5.8E-06 | 0.000573 | 0.13434 |
|  | rs80028338 | 17 | 44161470 | 4.83E-07 | 0.000991 | 0.973489 | 8.25E-06 | 0.00041 | 0.670693 |
|  | rs80184151 | 17 | 43879308 | 6.55E-08 | 0.006842 | 0.410099 | 7.45E-06 | 0.000451 | 0.447365 |
|  | rs80209523 | 17 | 43933830 | 2.02E-07 | 0.002298 | 0.934401 | 3.79E-06 | 0.000863 | 0.069231 |
|  | rs8072451 | 17 | 43893716 | 5.5E-08 | 0.008115 | 0.246762 | 4.43E-06 | 0.000742 | 0.094505 |
|  | rs8073146 | 17 | 43893751 | 5.71E-08 | 0.007816 | 0.28632 | 4.53E-06 | 0.000726 | 0.098912 |
|  | rs878886 | 17 | 43912490 | 1.3E-07 | 0.00353 | 0.802857 | 7.69E-06 | 0.000438 | 0.511204 |
|  | rs878887 | 17 | 43912582 | 1.09E-07 | 0.004166 | 0.738091 | 6.59E-06 | 0.000508 | 0.208592 |
|  | rs878888 | 17 | 43912635 | 1.13E-07 | 0.004051 | 0.746304 | 6.86E-06 | 0.000489 | 0.261966 |
| rs9320913 | rs12202969 | 6 | 98576223 | 3.57E-21 | 0.074162 | 0.851714 | 1.59E-06 | 0.062703 | 0.589952 |
|  | rs12206087 | 6 | 98582900 | 4.24E-21 | 0.062488 | 0.914202 | 1.51E-06 | 0.06606 | 0.39513 |
|  | rs1487441 | 6 | 98553894 | 2.48E-21 | 0.106317 | 0.512081 | 3.07E-06 | 0.033313 | 0.864136 |
|  | rs1487445 | 6 | 98565211 | 2.12E-21 | 0.123955 | 0.405763 | 1.95E-06 | 0.051567 | 0.755943 |
|  | rs1906252 | 6 | 98550289 | 5.89E-21 | 0.045126 | 0.959329 | 2.92E-06 | 0.035054 | 0.830823 |
|  | rs2388334 | 6 | 98591622 | 6.56E-21 | 0.040578 | 0.999907 | 1E-06 | 0.097637 | 0.260788 |
|  | rs9320913 | 6 | 98584733 | 2.05E-21 | 0.128325 | 0.281808 | 1.51E-06 | 0.06606 | 0.527249 |
|  | rs9372734 | 6 | 98577689 | 2.97E-21 | 0.088755 | 0.692205 | 1.59E-06 | 0.062627 | 0.65258 |
|  | rs9375188 | 6 | 98555272 | 3.09E-21 | 0.085348 | 0.777553 | 2.55E-06 | 0.039826 | 0.795769 |
|  | rs9401593 | 6 | 98549801 | 1.71E-21 | 0.153483 | 0.153483 | 3.44E-06 | 0.029881 | 0.894017 |
|  | rs968050 | 6 | 98574560 | 2.89E-21 | 0.091369 | 0.60345 | 1.94E-06 | 0.051797 | 0.704376 |

Abbreviations: probNorm refers to the normalized posterior p-value for each SNP. cumSum means the cumulative normalized posterior p-value for SNP sets. For each sentinel SNP, a 99% credible set of potentially causal SNPs was obtained using the FM-summary method. Each SNP has a corresponding posterior probability, i.e., probNorm, and with each superimposition of another SNP, there is a subsequent cumSum, which is obtained by the the corresponding probNorm of each SNP is summed up. Ultimately, this can be interpreted to mean that there is a 99% probability that the causal SNPs that actually have an effect on the two traits are contained in such a credible set.

## Supplementary Table 7. Fine-mapping 99% credible-set of sentinel SNP from cross-trait meta-analysis between educational attainment (EA) and Alcohol use disorders (AUD)

| Sentinel.SNP | Credible-set SNPs | CHR | BP | EA | | | AUD | | |
| --- | --- | --- | --- | --- | --- | --- | --- | --- | --- |
|  |  |  |  | **GWAS-p-value** | **probNorm** | **cumSum** | **GWAS-p-value** | **probNorm** | **cumSum** |
| rs113925422 | rs10445362 | 17 | 43914554 | 1.32E-07 | 0.003515 | 0.821865 | 3.75E-08 | 0.002796 | 0.693122 |
|  | rs10445363 | 17 | 43914558 | 1.17E-07 | 0.003965 | 0.758735 | 3.74E-08 | 0.002797 | 0.690326 |
|  | rs111321973 | 17 | 43953170 | 4.26E-07 | 0.001138 | 0.976295 | 3.06E-08 | 0.003398 | 0.574177 |
|  | rs111327992 | 17 | 44126691 | 3.45E-07 | 0.001395 | 0.967381 | 1.46E-08 | 0.006955 | 0.213495 |
|  | rs111370985 | 17 | 43852742 | 1.76E-07 | 0.002674 | 0.919817 | 4.49E-08 | 0.002347 | 0.949761 |
|  | rs111372048 | 17 | 44136577 | 3.63E-07 | 0.001327 | 0.971375 | 1.91E-08 | 0.005368 | 0.292179 |
|  | rs111415173 | 17 | 43795573 | 5.92E-07 | 0.000828 | 0.986335 | 2.83E-08 | 0.003671 | 0.520644 |
|  | rs111433752 | 17 | 43857989 | 3.63E-07 | 0.001328 | 0.970048 | 4.31E-08 | 0.002442 | 0.93537 |
|  | rs111519055 | 17 | 44159672 | 5.48E-07 | 0.000891 | 0.982942 | 2.16E-08 | 0.004779 | 0.341863 |
|  | rs111541901 | 17 | 43994358 | 1.5E-07 | 0.003113 | 0.911361 | 3.27E-08 | 0.00319 | 0.597139 |
|  | rs111664122 | 17 | 43857990 | 3.6E-07 | 0.001338 | 0.968719 | 4.08E-08 | 0.002574 | 0.882613 |
|  | rs111735741 | 17 | 43856372 | 6.78E-08 | 0.006722 | 0.425823 | 2.64E-08 | 0.003927 | 0.463561 |
|  | rs111739681 | 17 | 43898459 | 8.71E-08 | 0.00527 | 0.628785 | 3.33E-08 | 0.003133 | 0.60657 |
|  | rs111751251 | 17 | 44042951 | 2.35E-07 | 0.002022 | 0.950661 | 3.78E-08 | 0.002769 | 0.715367 |
|  | rs111878933 | 17 | 43856341 | 1.9E-07 | 0.002484 | 0.935299 | 2.64E-08 | 0.003932 | 0.459634 |
|  | rs111913701 | 17 | 44159631 | 5.32E-07 | 0.000917 | 0.982051 | 2.16E-08 | 0.004779 | 0.337084 |
|  | rs111962225 | 17 | 43934016 | 1.48E-07 | 0.003166 | 0.901961 | 3.32E-08 | 0.003147 | 0.603437 |
|  | rs111970616 | 17 | 44169581 | 6.04E-07 | 0.000812 | 0.988784 | 2.04E-08 | 0.005045 | 0.312593 |
|  | rs111985258 | 17 | 43795859 | 6E-07 | 0.000817 | 0.987972 | 2.81E-08 | 0.003698 | 0.50961 |
|  | rs112003140 | 17 | 43980998 | 1.48E-07 | 0.003152 | 0.905112 | 2.88E-08 | 0.003612 | 0.546184 |
|  | rs112003311 | 17 | 44042939 | 5.19E-07 | 0.00094 | 0.981134 | 3.54E-08 | 0.00295 | 0.621655 |
|  | rs112166495 | 17 | 44005361 | 1.19E-07 | 0.003907 | 0.774462 | 1.37E-08 | 0.007416 | 0.19224 |
|  | rs112197756 | 17 | 44154105 | 5.76E-07 | 0.00085 | 0.984673 | 2.2E-08 | 0.004688 | 0.365519 |
|  | rs112275277 | 17 | 43981958 | 7.13E-08 | 0.006397 | 0.44558 | 2.66E-08 | 0.003893 | 0.471367 |
|  | rs112333322 | 17 | 44126673 | 3.3E-07 | 0.001456 | 0.965986 | 1.85E-08 | 0.005551 | 0.286811 |
|  | rs112364920 | 17 | 44161360 | 5.09E-07 | 0.000957 | 0.979247 | 2.04E-08 | 0.005047 | 0.307549 |
|  | rs112385572 | 17 | 44066172 | 9.51E-08 | 0.004842 | 0.684149 | 2.17E-08 | 0.004755 | 0.351389 |
|  | rs112439933 | 17 | 44005226 | 1.25E-07 | 0.003731 | 0.78587 | 1.65E-08 | 0.006212 | 0.251516 |
|  | rs112454267 | 17 | 44005329 | 1.19E-07 | 0.003897 | 0.778359 | 2.48E-08 | 0.004175 | 0.419148 |
|  | rs112570965 | 17 | 43801092 | 5.98E-07 | 0.00082 | 0.987155 | 2.82E-08 | 0.003683 | 0.513292 |
|  | rs112572874 | 17 | 44072984 | 3.18E-07 | 0.001506 | 0.963025 | 2.51E-08 | 0.004115 | 0.431564 |
|  | rs112578465 | 17 | 44125066 | 2.65E-07 | 0.001797 | 0.958305 | 1.81E-08 | 0.005663 | 0.28126 |
|  | rs112583797 | 17 | 43918418 | 8.18E-08 | 0.005605 | 0.558298 | 3.81E-08 | 0.002747 | 0.759453 |
|  | rs112596352 | 17 | 44170238 | 3.67E-07 | 0.001312 | 0.972687 | 1.71E-08 | 0.00599 | 0.269774 |
|  | rs112647192 | 17 | 43994623 | 8.11E-08 | 0.005652 | 0.552692 | 2.53E-08 | 0.004093 | 0.435657 |
|  | rs113029914 | 17 | 43981831 | 3.03E-07 | 0.001578 | 0.961518 | 2.67E-08 | 0.003887 | 0.475254 |
|  | rs113093579 | 17 | 43934116 | 1.82E-07 | 0.002591 | 0.930317 | 2.98E-08 | 0.003494 | 0.560326 |
|  | rs113161176 | 17 | 43974354 | 9.06E-08 | 0.005077 | 0.654578 | 2.72E-08 | 0.003809 | 0.502133 |
|  | rs113313477 | 17 | 44005186 | 1.14E-07 | 0.004052 | 0.750782 | 1.63E-08 | 0.006268 | 0.245304 |
|  | rs113414067 | 17 | 43955093 | 1.37E-07 | 0.003408 | 0.839134 | 2.82E-08 | 0.003681 | 0.516973 |
|  | rs113589236 | 17 | 43981795 | 8.36E-08 | 0.005485 | 0.574773 | 2.67E-08 | 0.003887 | 0.479142 |
|  | rs113788190 | 17 | 44161302 | 5.15E-07 | 0.000947 | 0.980194 | 2.04E-08 | 0.005047 | 0.302502 |
|  | rs113796169 | 17 | 44005254 | 1.23E-07 | 0.00378 | 0.78214 | 2.48E-08 | 0.004172 | 0.42332 |
|  | rs113856644 | 17 | 43932277 | 1.04E-07 | 0.004461 | 0.71655 | 4.55E-08 | 0.002317 | 0.952079 |
|  | rs113871181 | 17 | 43852733 | 1.77E-07 | 0.002657 | 0.922475 | 3.92E-08 | 0.002677 | 0.835446 |
|  | rs113925422 | 17 | 44046934 | 1.02E-07 | 0.00452 | 0.707586 | 2.6E-08 | 0.003988 | 0.447771 |
|  | rs113991678 | 17 | 43795634 | 6.33E-07 | 0.000775 | 0.98956 | 2.83E-08 | 0.00367 | 0.527984 |
|  | rs117124984 | 17 | 44051588 | 1.39E-07 | 0.00336 | 0.866145 | 4.12E-08 | 0.002549 | 0.892828 |
|  | rs117365970 | 17 | 43893259 | 3.98E-08 | 0.011262 | 0.06573 | 3.31E-08 | 0.00315 | 0.60029 |
|  | rs117646503 | 17 | 43893260 | 4.43E-08 | 0.010144 | 0.075874 | 4.03E-08 | 0.002607 | 0.864502 |
|  | rs118087478 | 17 | 44051589 | 1.28E-07 | 0.003629 | 0.800543 | 3.81E-08 | 0.002752 | 0.740206 |
|  | rs12150390 | 17 | 43896228 | 5.5E-08 | 0.008232 | 0.247975 | 3.94E-08 | 0.00266 | 0.846114 |
|  | rs1396862 | 17 | 43902997 | 6.65E-08 | 0.006846 | 0.412361 | 3.77E-08 | 0.002781 | 0.70149 |
|  | rs140713557 | 17 | 43953179 | 3.88E-07 | 0.001243 | 0.97393 | 3.08E-08 | 0.003378 | 0.580947 |
|  | rs16940665 | 17 | 43907896 | 1.38E-07 | 0.00337 | 0.852696 | 3.81E-08 | 0.002748 | 0.756706 |
|  | rs16940668 | 17 | 43907966 | 1.44E-07 | 0.003236 | 0.892367 | 3.94E-08 | 0.002664 | 0.843454 |
|  | rs16940671 | 17 | 43908151 | 5.82E-08 | 0.00779 | 0.295509 | 5.69E-08 | 0.001863 | 0.99072 |
|  | rs16940674 | 17 | 43910507 | 1.36E-07 | 0.00342 | 0.835726 | 3.87E-08 | 0.002709 | 0.803116 |
|  | rs16940676 | 17 | 43911036 | 1.35E-07 | 0.003445 | 0.832306 | 3.85E-08 | 0.002724 | 0.786818 |
|  | rs16940677 | 17 | 43911898 | 1.37E-07 | 0.003396 | 0.845933 | 3.81E-08 | 0.00275 | 0.751209 |
|  | rs16940681 | 17 | 43912159 | 1.39E-07 | 0.003363 | 0.859424 | 4.02E-08 | 0.00261 | 0.861895 |
|  | rs17425752 | 17 | 43906726 | 1.1E-07 | 0.004224 | 0.742619 | 4.34E-08 | 0.002424 | 0.940219 |
|  | rs17426174 | 17 | 43830938 | 1.72E-07 | 0.002731 | 0.917144 | 2.98E-08 | 0.003494 | 0.56382 |
|  | rs17689378 | 17 | 43881790 | 7.57E-08 | 0.006042 | 0.476604 | 3.55E-08 | 0.002942 | 0.627545 |
|  | rs17689471 | 17 | 43892973 | 7.68E-08 | 0.005955 | 0.500506 | 4.05E-08 | 0.002591 | 0.872297 |
|  | rs17689608 | 17 | 43896528 | 4.5E-08 | 0.009993 | 0.116017 | 3.68E-08 | 0.002842 | 0.665029 |
|  | rs17689653 | 17 | 43898963 | 5.97E-08 | 0.007606 | 0.303116 | 3.62E-08 | 0.002891 | 0.645047 |
|  | rs17689824 | 17 | 43904397 | 2.25E-07 | 0.002102 | 0.948639 | 3.81E-08 | 0.002749 | 0.753958 |
|  | rs17689918 | 17 | 43910088 | 1.16E-07 | 0.003988 | 0.754771 | 3.74E-08 | 0.002799 | 0.68753 |
|  | rs17762769 | 17 | 43893403 | 6.84E-08 | 0.006662 | 0.439183 | 3.98E-08 | 0.002634 | 0.854043 |
|  | rs17762882 | 17 | 43898887 | 8.5E-08 | 0.005401 | 0.612924 | 3.74E-08 | 0.002803 | 0.679128 |
|  | rs17762912 | 17 | 43899161 | 6.48E-08 | 0.007021 | 0.398571 | 4.42E-08 | 0.002379 | 0.947414 |
|  | rs17762954 | 17 | 43899786 | 7.77E-08 | 0.005885 | 0.518192 | 2.93E-08 | 0.003545 | 0.553321 |
|  | rs17763050 | 17 | 43903336 | 6.09E-08 | 0.007461 | 0.33313 | 3.85E-08 | 0.002722 | 0.794984 |
|  | rs17763086 | 17 | 43905481 | 7.6E-08 | 0.006018 | 0.482622 | 3.84E-08 | 0.002726 | 0.784094 |
|  | rs17763199 | 17 | 43910183 | 1.44E-07 | 0.003245 | 0.88913 | 3.73E-08 | 0.002804 | 0.676325 |
|  | rs1876827 | 17 | 43911832 | 1.31E-07 | 0.003549 | 0.814819 | 3.89E-08 | 0.002695 | 0.819327 |
|  | rs1876828 | 17 | 43911525 | 1.41E-07 | 0.00331 | 0.872803 | 3.88E-08 | 0.0027 | 0.816632 |
|  | rs1876829 | 17 | 43911443 | 1.32E-07 | 0.00353 | 0.81835 | 3.88E-08 | 0.002701 | 0.813932 |
|  | rs1876830 | 17 | 43911352 | 1.41E-07 | 0.003307 | 0.876111 | 3.88E-08 | 0.002703 | 0.811231 |
|  | rs1876831 | 17 | 43907745 | 1.89E-07 | 0.002498 | 0.932815 | 3.81E-08 | 0.00275 | 0.748459 |
|  | rs1912151 | 17 | 43902944 | 6.33E-08 | 0.007177 | 0.384529 | 3.69E-08 | 0.002834 | 0.667863 |
|  | rs2316763 | 17 | 43895530 | 5.35E-08 | 0.008448 | 0.198076 | 4.19E-08 | 0.00251 | 0.918125 |
|  | rs2316764 | 17 | 43895602 | 4.61E-08 | 0.009758 | 0.135764 | 3.91E-08 | 0.00268 | 0.832769 |
|  | rs2316765 | 17 | 43912454 | 1.3E-07 | 0.003572 | 0.807698 | 3.89E-08 | 0.002692 | 0.822019 |
|  | rs28364021 | 17 | 43912282 | 8.7E-08 | 0.00528 | 0.623515 | 3.89E-08 | 0.002692 | 0.827403 |
|  | rs28364023 | 17 | 43894159 | 2.09E-07 | 0.002263 | 0.939894 | 3.85E-08 | 0.002722 | 0.78954 |
|  | rs28364025 | 17 | 43894102 | 7.25E-08 | 0.0063 | 0.458204 | 3.92E-08 | 0.002676 | 0.838123 |
|  | rs3885074 | 17 | 43902738 | 7.35E-08 | 0.006216 | 0.464419 | 3.8E-08 | 0.002756 | 0.737454 |
|  | rs3885075 | 17 | 43902799 | 2.92E-07 | 0.001635 | 0.959941 | 3.79E-08 | 0.002763 | 0.72366 |
|  | rs41280116 | 17 | 43902842 | 2.19E-07 | 0.002165 | 0.946537 | 3.79E-08 | 0.002763 | 0.720897 |
|  | rs41280118 | 17 | 43903089 | 5.64E-08 | 0.008032 | 0.256007 | 3.77E-08 | 0.002781 | 0.704271 |
|  | rs41457044 | 17 | 43911424 | 1.3E-07 | 0.003572 | 0.811271 | 3.87E-08 | 0.00271 | 0.800407 |
|  | rs4277389 | 17 | 43895653 | 4.89E-08 | 0.009218 | 0.154402 | 3.5E-08 | 0.00299 | 0.615731 |
|  | rs4309444 | 17 | 43895797 | 5.43E-08 | 0.008333 | 0.223185 | 4.02E-08 | 0.002613 | 0.859285 |
|  | rs4335809 | 17 | 43902216 | 8.42E-08 | 0.005445 | 0.602106 | 3.8E-08 | 0.002759 | 0.734698 |
|  | rs4341787 | 17 | 43902505 | 9.82E-08 | 0.004692 | 0.703066 | 3.8E-08 | 0.00276 | 0.72918 |
|  | rs4482334 | 17 | 43912830 | 1.25E-07 | 0.003705 | 0.789575 | 3.66E-08 | 0.002861 | 0.65077 |
|  | rs4523962 | 17 | 43902522 | 1.06E-07 | 0.004367 | 0.734167 | 3.8E-08 | 0.00276 | 0.73194 |
|  | rs4525537 | 17 | 43912723 | 1.17E-07 | 0.003952 | 0.762687 | 3.66E-08 | 0.00286 | 0.65649 |
|  | rs4564621 | 17 | 43895501 | 6.27E-08 | 0.00725 | 0.377352 | 3.75E-08 | 0.002791 | 0.698709 |
|  | rs4566211 | 17 | 43895696 | 5.44E-08 | 0.008323 | 0.231507 | 4.17E-08 | 0.002521 | 0.910579 |
|  | rs4566212 | 17 | 43895751 | 5.35E-08 | 0.008452 | 0.189629 | 4.22E-08 | 0.00249 | 0.920615 |
|  | rs4640231 | 17 | 43912786 | 1.03E-07 | 0.004503 | 0.712089 | 3.66E-08 | 0.002861 | 0.647908 |
|  | rs55668363 | 17 | 43892788 | 6.8E-08 | 0.006698 | 0.432521 | 2.54E-08 | 0.004075 | 0.439732 |
|  | rs55763795 | 17 | 43908773 | 1.42E-07 | 0.00328 | 0.879391 | 3.81E-08 | 0.002752 | 0.742959 |
|  | rs55779147 | 17 | 43894510 | 5.79E-08 | 0.007829 | 0.287719 | 3.88E-08 | 0.002705 | 0.808528 |
|  | rs55865707 | 17 | 43908826 | 1.37E-07 | 0.003394 | 0.849326 | 3.81E-08 | 0.002751 | 0.745709 |
|  | rs55915917 | 17 | 43892784 | 6.17E-08 | 0.00736 | 0.362791 | 2.32E-08 | 0.004453 | 0.397928 |
|  | rs55943044 | 17 | 43872228 | 4.78E-08 | 0.00942 | 0.145184 | 5.51E-08 | 0.001923 | 0.988857 |
|  | rs55979424 | 17 | 43900081 | 8.47E-08 | 0.005418 | 0.607524 | 4.31E-08 | 0.002442 | 0.932928 |
|  | rs56099546 | 17 | 43894609 | 4.5E-08 | 0.009989 | 0.126006 | 3.7E-08 | 0.002829 | 0.670693 |
|  | rs56127111 | 17 | 43913315 | 2.44E-07 | 0.001949 | 0.954581 | 3.9E-08 | 0.002687 | 0.830089 |
|  | rs56319902 | 17 | 43871982 | 5.14E-08 | 0.008795 | 0.181176 | 5.35E-08 | 0.001978 | 0.985001 |
|  | rs56357543 | 17 | 43894547 | 4.99E-08 | 0.009048 | 0.16345 | 3.7E-08 | 0.002828 | 0.67352 |
|  | rs62054760 | 17 | 43908989 | 1.45E-07 | 0.003215 | 0.895582 | 4.03E-08 | 0.002604 | 0.867106 |
|  | rs62054761 | 17 | 43909008 | 1.44E-07 | 0.003247 | 0.882638 | 3.93E-08 | 0.002667 | 0.84079 |
|  | rs62054762 | 17 | 43909022 | 1.39E-07 | 0.003365 | 0.856061 | 3.83E-08 | 0.002734 | 0.778641 |
|  | rs62054763 | 17 | 43910262 | 1.33E-07 | 0.003492 | 0.828861 | 3.87E-08 | 0.002708 | 0.805823 |
|  | rs62054802 | 17 | 43914598 | 1.39E-07 | 0.00336 | 0.862784 | 3.77E-08 | 0.002778 | 0.707049 |
|  | rs62054803 | 17 | 43914728 | 7.75E-08 | 0.005905 | 0.506411 | 3.79E-08 | 0.002761 | 0.726421 |
|  | rs62054804 | 17 | 43914809 | 8.33E-08 | 0.005505 | 0.563803 | 3.23E-08 | 0.003224 | 0.593949 |
|  | rs62054805 | 17 | 43915054 | 9.77E-08 | 0.004717 | 0.693663 | 3.78E-08 | 0.002774 | 0.712598 |
|  | rs62054835 | 17 | 43934672 | 1.53E-07 | 0.003052 | 0.914413 | 2.89E-08 | 0.003592 | 0.549776 |
|  | rs62055869 | 17 | 43830685 | 1.79E-07 | 0.002624 | 0.927727 | 2.99E-08 | 0.003474 | 0.570779 |
|  | rs62056785 | 17 | 43975263 | 8.39E-08 | 0.005469 | 0.591199 | 2.71E-08 | 0.003833 | 0.486853 |
|  | rs62056786 | 17 | 43975285 | 1.04E-07 | 0.004428 | 0.725422 | 2.71E-08 | 0.003832 | 0.490685 |
|  | rs62056790 | 17 | 43975417 | 1.18E-07 | 0.003926 | 0.770555 | 2.72E-08 | 0.003819 | 0.494504 |
|  | rs62056851 | 17 | 43992806 | 1.06E-07 | 0.004379 | 0.7298 | 2.46E-08 | 0.0042 | 0.414973 |
|  | rs62057062 | 17 | 43856710 | 9.27E-08 | 0.004962 | 0.669599 | 4.58E-08 | 0.002299 | 0.963593 |
|  | rs62057063 | 17 | 43856730 | 9.01E-08 | 0.005101 | 0.649501 | 4.89E-08 | 0.002159 | 0.974658 |
|  | rs62057064 | 17 | 43857033 | 7.76E-08 | 0.005896 | 0.512307 | 4.57E-08 | 0.002306 | 0.954385 |
|  | rs62057067 | 17 | 43858326 | 9.78E-08 | 0.004712 | 0.698375 | 4.25E-08 | 0.002472 | 0.925565 |
|  | rs62057068 | 17 | 43858629 | 8.36E-08 | 0.005484 | 0.580258 | 4.24E-08 | 0.002479 | 0.923094 |
|  | rs62057069 | 17 | 43859065 | 7.97E-08 | 0.005744 | 0.541329 | 4.18E-08 | 0.002516 | 0.915616 |
|  | rs62057070 | 17 | 43859640 | 9.13E-08 | 0.005039 | 0.659617 | 4.14E-08 | 0.002535 | 0.902994 |
|  | rs62057071 | 17 | 43859691 | 8.02E-08 | 0.005711 | 0.54704 | 4.14E-08 | 0.002538 | 0.900459 |
|  | rs62057101 | 17 | 43885291 | 8.84E-08 | 0.005199 | 0.63921 | 4.26E-08 | 0.002468 | 0.928033 |
|  | rs62057107 | 17 | 43896032 | 3.04E-08 | 0.014636 | 0.030472 | 3.23E-08 | 0.003229 | 0.590726 |
|  | rs62057108 | 17 | 43896616 | 6.04E-08 | 0.007517 | 0.318161 | 3.57E-08 | 0.002926 | 0.639261 |
|  | rs62057109 | 17 | 43896637 | 6.03E-08 | 0.007528 | 0.310644 | 3.57E-08 | 0.002926 | 0.636335 |
|  | rs62057110 | 17 | 43896734 | 6.1E-08 | 0.00744 | 0.340569 | 3.68E-08 | 0.002843 | 0.662187 |
|  | rs62057111 | 17 | 43897130 | 5.71E-08 | 0.007938 | 0.271958 | 4.17E-08 | 0.00252 | 0.9131 |
|  | rs62057112 | 17 | 43897202 | 6.11E-08 | 0.007427 | 0.355431 | 3.94E-08 | 0.002659 | 0.848773 |
|  | rs62057113 | 17 | 43897449 | 5.39E-08 | 0.008394 | 0.206471 | 3.56E-08 | 0.002936 | 0.630481 |
|  | rs62057114 | 17 | 43899401 | 7.95E-08 | 0.005762 | 0.529826 | 4.12E-08 | 0.002547 | 0.895375 |
|  | rs62057115 | 17 | 43899417 | 8.38E-08 | 0.005472 | 0.58573 | 4.11E-08 | 0.002557 | 0.887731 |
|  | rs62057116 | 17 | 43899655 | 6.21E-08 | 0.007311 | 0.370102 | 4.34E-08 | 0.002425 | 0.937795 |
|  | rs62057117 | 17 | 43899657 | 6.76E-08 | 0.006741 | 0.419102 | 4.29E-08 | 0.002453 | 0.930487 |
|  | rs62057118 | 17 | 43899727 | 9.6E-08 | 0.004797 | 0.688945 | 3.07E-08 | 0.003392 | 0.577569 |
|  | rs62057119 | 17 | 43899736 | 7.79E-08 | 0.005873 | 0.524065 | 2.96E-08 | 0.003511 | 0.556832 |
|  | rs62057121 | 17 | 43900760 | 2.8E-08 | 0.015836 | 0.015836 | 2.61E-08 | 0.003969 | 0.45174 |
|  | rs62057122 | 17 | 43901001 | 8.4E-08 | 0.005462 | 0.596661 | 4.07E-08 | 0.00258 | 0.877459 |
|  | rs62057123 | 17 | 43901238 | 9.51E-08 | 0.004843 | 0.679307 | 3.51E-08 | 0.002975 | 0.618705 |
|  | rs62057143 | 17 | 43901528 | 9.46E-08 | 0.004865 | 0.674464 | 3.79E-08 | 0.002767 | 0.718134 |
|  | rs62057144 | 17 | 43901558 | 1.39E-07 | 0.003349 | 0.869494 | 3.36E-08 | 0.003106 | 0.609677 |
|  | rs62057146 | 17 | 43903106 | 5.05E-08 | 0.008932 | 0.172381 | 3.77E-08 | 0.002775 | 0.709824 |
|  | rs62057147 | 17 | 43903298 | 3.67E-08 | 0.012171 | 0.042643 | 5.1E-08 | 0.002074 | 0.980961 |
|  | rs62057148 | 17 | 43903485 | 6.48E-08 | 0.007021 | 0.39155 | 3.75E-08 | 0.002795 | 0.695918 |
|  | rs62057149 | 17 | 43903546 | 8.85E-08 | 0.005191 | 0.6444 | 3.74E-08 | 0.002802 | 0.681929 |
|  | rs62057150 | 17 | 43903548 | 8.79E-08 | 0.005226 | 0.634011 | 3.74E-08 | 0.002801 | 0.68473 |
|  | rs62057153 | 17 | 43904528 | 1.26E-07 | 0.003699 | 0.793274 | 3.83E-08 | 0.002739 | 0.775907 |
|  | rs62057154 | 17 | 43904610 | 9.16E-08 | 0.005019 | 0.664636 | 3.82E-08 | 0.002745 | 0.762198 |
|  | rs62057155 | 17 | 43904673 | 8.36E-08 | 0.005486 | 0.569288 | 3.82E-08 | 0.002744 | 0.764942 |
|  | rs62057156 | 17 | 43904948 | 8.64E-08 | 0.00531 | 0.618235 | 3.82E-08 | 0.002742 | 0.770428 |
|  | rs62057157 | 17 | 43905313 | 7.44E-08 | 0.006143 | 0.470563 | 3.82E-08 | 0.00274 | 0.773168 |
|  | rs62057158 | 17 | 43907143 | 1.49E-07 | 0.003135 | 0.908248 | 3.84E-08 | 0.002727 | 0.781368 |
|  | rs62059005 | 17 | 44004472 | 3.93E-07 | 0.001228 | 0.975158 | 2.46E-08 | 0.004208 | 0.410773 |
|  | rs62062277 | 17 | 44093753 | 1.79E-07 | 0.002627 | 0.925102 | 2.51E-08 | 0.004129 | 0.427449 |
|  | rs62062278 | 17 | 44093860 | 2.13E-07 | 0.002223 | 0.944373 | 2.17E-08 | 0.004742 | 0.356131 |
|  | rs62062279 | 17 | 44093964 | 1.28E-07 | 0.00364 | 0.796914 | 2.23E-08 | 0.004624 | 0.388883 |
|  | rs62062280 | 17 | 44093993 | 1.44E-07 | 0.003247 | 0.885885 | 2.25E-08 | 0.004592 | 0.393475 |
|  | rs62063271 | 17 | 44036047 | 2.41E-07 | 0.001971 | 0.952632 | 2.2E-08 | 0.004687 | 0.374893 |
|  | rs62641967 | 17 | 44047216 | 2.47E-07 | 0.001927 | 0.956508 | 2.16E-08 | 0.004771 | 0.346634 |
|  | rs739644 | 17 | 43895008 | 4.48E-08 | 0.010048 | 0.095978 | 3.86E-08 | 0.002713 | 0.797697 |
|  | rs739645 | 17 | 43894990 | 4.48E-08 | 0.010045 | 0.106023 | 3.98E-08 | 0.002635 | 0.851408 |
|  | rs74998289 | 17 | 43913558 | 1.18E-07 | 0.003942 | 0.766629 | 4.72E-08 | 0.002235 | 0.968081 |
|  | rs75104593 | 17 | 43913557 | 1.37E-07 | 0.003403 | 0.842537 | 4.68E-08 | 0.002253 | 0.965846 |
|  | rs75330746 | 17 | 44182955 | 6.86E-07 | 0.000718 | 0.990277 | 4.57E-08 | 0.002305 | 0.95669 |
|  | rs75530705 | 17 | 44154048 | 5.55E-07 | 0.000881 | 0.983823 | 2.2E-08 | 0.004683 | 0.379576 |
|  | rs76154201 | 17 | 44154033 | 5.87E-07 | 0.000835 | 0.985507 | 2.2E-08 | 0.004683 | 0.384258 |
|  | rs76563578 | 17 | 43933879 | 2.09E-07 | 0.002256 | 0.94215 | 3.85E-08 | 0.002722 | 0.792262 |
|  | rs76830096 | 17 | 43857129 | 7.66E-08 | 0.005973 | 0.488595 | 4.35E-08 | 0.002421 | 0.94264 |
|  | rs78074121 | 17 | 43896690 | 6.05E-08 | 0.007508 | 0.325669 | 3.19E-08 | 0.00327 | 0.587496 |
|  | rs78506181 | 17 | 43897480 | 5.4E-08 | 0.008381 | 0.214852 | 4.15E-08 | 0.002531 | 0.908058 |
|  | rs78587102 | 17 | 43897246 | 4.47E-08 | 0.010056 | 0.08593 | 4.07E-08 | 0.002582 | 0.874879 |
|  | rs78872653 | 17 | 43905134 | 7.68E-08 | 0.005957 | 0.494552 | 3.82E-08 | 0.002744 | 0.767686 |
|  | rs78917479 | 17 | 43899611 | 6.11E-08 | 0.007435 | 0.348004 | 4.12E-08 | 0.002549 | 0.89028 |
|  | rs78917495 | 17 | 43858482 | 7.95E-08 | 0.005759 | 0.535585 | 4.13E-08 | 0.002545 | 0.897921 |
|  | rs79501144 | 17 | 43900697 | 1.04E-07 | 0.004444 | 0.720994 | 3.55E-08 | 0.002948 | 0.624603 |
|  | rs79600142 | 17 | 43897722 | 5.65E-08 | 0.008013 | 0.26402 | 3.57E-08 | 0.002928 | 0.633408 |
|  | rs80184151 | 17 | 43879308 | 6.55E-08 | 0.006944 | 0.405515 | 4.72E-08 | 0.002234 | 0.970315 |
|  | rs80209523 | 17 | 43933830 | 2.02E-07 | 0.002332 | 0.937631 | 2.09E-08 | 0.004915 | 0.327469 |
|  | rs8072451 | 17 | 43893716 | 5.5E-08 | 0.008236 | 0.239743 | 2.56E-08 | 0.00405 | 0.443783 |
|  | rs8073146 | 17 | 43893751 | 5.71E-08 | 0.007933 | 0.27989 | 2.75E-08 | 0.003779 | 0.505911 |
|  | rs878886 | 17 | 43912490 | 1.3E-07 | 0.003583 | 0.804126 | 3.89E-08 | 0.002692 | 0.824711 |
|  | rs878887 | 17 | 43912582 | 1.09E-07 | 0.004228 | 0.738395 | 3.67E-08 | 0.002854 | 0.659344 |
|  | rs878888 | 17 | 43912635 | 1.13E-07 | 0.004111 | 0.74673 | 3.66E-08 | 0.002861 | 0.65363 |
| rs13266268 | rs1078141 | 8 | 1.43E+08 | 2.06E-10 | 0.056189 | 0.857414 | 3.25E-05 | 0.047065 | 0.866672 |
|  | rs1106761 | 8 | 1.43E+08 | 4.08E-11 | 0.274422 | 0.274422 | 3.3E-05 | 0.04635 | 0.913022 |
|  | rs11782074 | 8 | 1.43E+08 | 6.77E-11 | 0.166982 | 0.71458 | 3.31E-05 | 0.046228 | 0.95925 |
|  | rs11782665 | 8 | 1.43E+08 | 4.31E-10 | 0.027372 | 0.964329 | 0.000175 | 0.009588 | 0.990458 |
|  | rs11787216 | 8 | 1.43E+08 | 6.12E-10 | 0.019435 | 0.983765 | 6.66E-06 | 0.213296 | 0.564985 |
|  | rs13266268 | 8 | 1.43E+08 | 4.1E-11 | 0.273176 | 0.547598 | 3.95E-06 | 0.351689 | 0.351689 |
|  | rs7357604 | 8 | 1.43E+08 | 8.71E-10 | 0.013783 | 0.997547 | 0.000149 | 0.01116 | 0.97041 |
|  | rs746839 | 8 | 1.43E+08 | 1.33E-10 | 0.086645 | 0.801225 | 1.96E-05 | 0.075934 | 0.819607 |
|  | rs903959 | 8 | 1.43E+08 | 3.35E-10 | 0.035033 | 0.936958 | 0.000159 | 0.01046 | 0.98087 |
| rs1338549 | rs10484867 | 6 | 98332567 | 1.37E-16 | 0.029597 | 0.585622 | 1.27E-05 | 0.027686 | 0.727572 |
|  | rs10484868 | 6 | 98332622 | 1.51E-16 | 0.026962 | 0.811343 | 1.27E-05 | 0.02771 | 0.699886 |
|  | rs12189679 | 6 | 98333409 | 1.08E-16 | 0.03755 | 0.257203 | 1.41E-05 | 0.025135 | 0.970656 |
|  | rs12205774 | 6 | 98324132 | 1.17E-16 | 0.034664 | 0.364036 | 1.06E-05 | 0.033102 | 0.292658 |
|  | rs12662596 | 6 | 98329165 | 1.88E-16 | 0.021692 | 0.935003 | 1.13E-05 | 0.031072 | 0.582536 |
|  | rs12662607 | 6 | 98329264 | 1.57E-16 | 0.02583 | 0.863532 | 1.12E-05 | 0.031195 | 0.551465 |
|  | rs1338549 | 6 | 98312143 | 8.26E-17 | 0.048804 | 0.1787 | 6.73E-06 | 0.050933 | 0.050933 |
|  | rs1933721 | 6 | 98313223 | 5.22E-17 | 0.076761 | 0.076761 | 1.04E-05 | 0.033468 | 0.193161 |
|  | rs2039719 | 6 | 98326863 | 1.46E-16 | 0.027874 | 0.756545 | 1.12E-05 | 0.031353 | 0.52027 |
|  | rs2039722 | 6 | 98314912 | 1.08E-16 | 0.037447 | 0.29465 | 1.2E-05 | 0.029294 | 0.642882 |
|  | rs4142530 | 6 | 98322282 | 9.86E-17 | 0.040953 | 0.219653 | 1.06E-05 | 0.032943 | 0.325601 |
|  | rs4580876 | 6 | 98322872 | 2E-16 | 0.020407 | 0.95541 | 3.1E-05 | 0.011882 | 1 |
|  | rs4839927 | 6 | 98325884 | 2.33E-16 | 0.017569 | 0.972979 | 1.06E-05 | 0.032936 | 0.424422 |
|  | rs4839928 | 6 | 98326012 | 7.57E-17 | 0.053135 | 0.129896 | 1.06E-05 | 0.032943 | 0.391486 |
|  | rs4839929 | 6 | 98326080 | 1.45E-16 | 0.028102 | 0.72867 | 1.07E-05 | 0.032726 | 0.457148 |
|  | rs7744474 | 6 | 98330626 | 1.32E-16 | 0.030817 | 0.525414 | 1.29E-05 | 0.027263 | 0.892833 |
|  | rs7756023 | 6 | 98330225 | 1.33E-16 | 0.03061 | 0.556024 | 1.28E-05 | 0.027613 | 0.782846 |
|  | rs7775492 | 6 | 98332930 | 1.4E-16 | 0.028952 | 0.614574 | 1.3E-05 | 0.027186 | 0.920019 |
|  | rs9320747 | 6 | 98310291 | 1.41E-16 | 0.02883 | 0.643404 | 9.87E-06 | 0.035298 | 0.159693 |
|  | rs9320759 | 6 | 98331357 | 1.46E-16 | 0.027837 | 0.784381 | 1.28E-05 | 0.02754 | 0.86557 |
|  | rs9372620 | 6 | 98330025 | 1.41E-16 | 0.02879 | 0.672194 | 1.2E-05 | 0.029294 | 0.672176 |
|  | rs9374896 | 6 | 98310484 | 1.54E-16 | 0.026359 | 0.837702 | 9.68E-06 | 0.03598 | 0.124395 |
|  | rs9374897 | 6 | 98310571 | 3.27E-16 | 0.012571 | 0.98555 | 9.27E-06 | 0.037482 | 0.088415 |
|  | rs9387823 | 6 | 98325000 | 1.28E-16 | 0.031622 | 0.494597 | 1.05E-05 | 0.03319 | 0.259556 |
|  | rs9387845 | 6 | 98328963 | 1.6E-16 | 0.02537 | 0.888902 | 1.13E-05 | 0.031051 | 0.613588 |
|  | rs9401250 | 6 | 98316447 | 1.28E-16 | 0.031744 | 0.462975 | 1.1E-05 | 0.031769 | 0.488917 |
|  | rs9401278 | 6 | 98324943 | 1.23E-16 | 0.032993 | 0.43123 | 1.05E-05 | 0.033205 | 0.226366 |
|  | rs9401305 | 6 | 98331937 | 1.17E-16 | 0.034722 | 0.329372 | 1.39E-05 | 0.025502 | 0.945521 |
|  | rs9489980 | 6 | 98325541 | 1.18E-16 | 0.034202 | 0.398237 | 1.06E-05 | 0.032943 | 0.358544 |
|  | rs9490000 | 6 | 98331085 | 1.67E-16 | 0.024409 | 0.913311 | 1.27E-05 | 0.027661 | 0.755233 |
|  | rs9490001 | 6 | 98331133 | 5.37E-16 | 0.007705 | 0.993255 | 1.28E-05 | 0.027595 | 0.810441 |
|  | rs9490003 | 6 | 98331316 | 1.43E-16 | 0.028373 | 0.700568 | 1.28E-05 | 0.027589 | 0.83803 |
| rs1879581 | rs11012 | 17 | 43513441 | 2.66E-06 | 0.054742 | 0.676353 | 1.1E-05 | 0.026046 | 1 |
|  | rs112538459 | 17 | 43527323 | 9.42E-07 | 0.148359 | 0.148359 | 9.27E-06 | 0.03067 | 0.699239 |
|  | rs12946900 | 17 | 43512206 | 1.95E-06 | 0.073713 | 0.492104 | 1.09E-05 | 0.026317 | 0.921355 |
|  | rs148269941 | 17 | 43540472 | 2.5E-05 | 0.006422 | 0.975148 | 9.04E-06 | 0.031414 | 0.637826 |
|  | rs17631303 | 17 | 43516402 | 3.4E-06 | 0.043234 | 0.905008 | 1.01E-05 | 0.028179 | 0.814749 |
|  | rs17631676 | 17 | 43549526 | 0.000052 | 0.003205 | 0.984123 | 5.4E-06 | 0.051414 | 0.410141 |
|  | rs1879581 | 17 | 43545893 | 2.1E-06 | 0.068614 | 0.560718 | 5.16E-06 | 0.053661 | 0.306582 |
|  | rs2139890 | 17 | 43541627 | 3.25E-06 | 0.045134 | 0.817578 | 9.24E-06 | 0.030744 | 0.66857 |
|  | rs34465449 | 17 | 43511435 | 1.66E-06 | 0.086005 | 0.338611 | 1.09E-05 | 0.026294 | 0.973954 |
|  | rs35489312 | 17 | 43517252 | 2.38E-06 | 0.060893 | 0.621611 | 1.07E-05 | 0.026707 | 0.868489 |
|  | rs55663797 | 17 | 43544379 | 4.19E-06 | 0.035351 | 0.940359 | 5.54E-06 | 0.050182 | 0.56226 |
|  | rs55671319 | 17 | 43548424 | 2.8E-05 | 0.00577 | 0.980918 | 5.47E-06 | 0.050806 | 0.512078 |
|  | rs55790407 | 17 | 43512439 | 3.13E-06 | 0.046722 | 0.772444 | 1.08E-05 | 0.026549 | 0.895038 |
|  | rs56005713 | 17 | 43536743 | 5.28E-06 | 0.028367 | 0.968726 | 9.77E-06 | 0.029144 | 0.758266 |
|  | rs56168933 | 17 | 43512318 | 1.8E-06 | 0.07978 | 0.418391 | 1.09E-05 | 0.026305 | 0.94766 |
|  | rs62064654 | 17 | 43513896 | 3.32E-06 | 0.044195 | 0.861773 | 1.01E-05 | 0.028304 | 0.78657 |
|  | rs62065378 | 17 | 43522361 | 1.36E-06 | 0.104247 | 0.252606 | 9.52E-06 | 0.029884 | 0.729123 |
|  | rs62065438 | 17 | 43558092 | 5.54E-05 | 0.003021 | 0.987144 | 4.73E-06 | 0.058313 | 0.142446 |
|  | rs9730 | 17 | 43513551 | 2.96E-06 | 0.049369 | 0.725722 | 1.06E-05 | 0.027032 | 0.841782 |
| rs2101975 | rs12639764 | 4 | 1.06E+08 | 1.37E-06 | 0.015999 | 0.985498 | 2.41E-06 | 0.15344 | 0.495163 |
|  | rs13103161 | 4 | 1.06E+08 | 1.51E-06 | 0.014502 | 1 | 2.31E-06 | 0.159881 | 0.341722 |
|  | rs13148166 | 4 | 1.06E+08 | 2.12E-07 | 0.096561 | 0.726342 | 2.97E-06 | 0.125461 | 0.620624 |
|  | rs2088483 | 4 | 1.06E+08 | 6.39E-07 | 0.033228 | 0.915005 | 2.02E-06 | 0.181841 | 0.181841 |
|  | rs2101975 | 4 | 1.06E+08 | 2.75E-07 | 0.075129 | 0.881777 | 5.5E-06 | 0.069587 | 0.888373 |
|  | rs2454202 | 4 | 1.06E+08 | 1.08E-07 | 0.184967 | 0.519086 | 1.29E-05 | 0.030852 | 0.967746 |
|  | rs2647228 | 4 | 1.06E+08 | 1.84E-07 | 0.110694 | 0.62978 | 3.41E-06 | 0.110133 | 0.730757 |
|  | rs2647239 | 4 | 1.06E+08 | 7.34E-07 | 0.02911 | 0.944115 | 8.02E-06 | 0.048521 | 0.936894 |
|  | rs2726513 | 4 | 1.06E+08 | 5.87E-08 | 0.334119 | 0.334119 | 4.3E-06 | 0.088029 | 0.818786 |
|  | rs57692580 | 4 | 1.06E+08 | 8.46E-07 | 0.025384 | 0.969499 | 1.72E-05 | 0.023442 | 0.991188 |
| rs538628 | rs1378358 | 17 | 44787312 | 4.3E-08 | 0.107959 | 0.318743 | 3.55E-08 | 0.009677 | 0.834286 |
|  | rs169201 | 17 | 44790203 | 9.69E-08 | 0.049067 | 0.513452 | 3.01E-08 | 0.011373 | 0.760902 |
|  | rs199439 | 17 | 44793503 | 1.54E-07 | 0.031332 | 0.5818 | 3.14E-08 | 0.010891 | 0.794215 |
|  | rs199443 | 17 | 44819565 | 4.16E-07 | 0.012011 | 0.90915 | 5.65E-08 | 0.006169 | 0.916811 |
|  | rs199445 | 17 | 44817408 | 3.76E-07 | 0.013239 | 0.884005 | 3.93E-08 | 0.00877 | 0.852626 |
|  | rs199447 | 17 | 44812188 | 4.83E-07 | 0.010395 | 0.940999 | 3.49E-08 | 0.009832 | 0.824609 |
|  | rs199448 | 17 | 44809001 | 3.79E-07 | 0.013134 | 0.897139 | 3.59E-08 | 0.00957 | 0.843856 |
|  | rs199451 | 17 | 44801784 | 2.64E-07 | 0.018629 | 0.710942 | 2.56E-08 | 0.013278 | 0.73752 |
|  | rs199456 | 17 | 44797919 | 2.95E-07 | 0.016706 | 0.779554 | 3.04E-08 | 0.011242 | 0.772144 |
|  | rs199457 | 17 | 44795469 | 2.58E-07 | 0.019061 | 0.692313 | 3.06E-08 | 0.01118 | 0.783324 |
|  | rs199503 | 17 | 44862162 | 5.86E-08 | 0.079859 | 0.398602 | 7.42E-08 | 0.004738 | 0.98798 |
|  | rs199504 | 17 | 44861003 | 2.29E-07 | 0.021373 | 0.652031 | 6.97E-08 | 0.005031 | 0.978389 |
|  | rs199505 | 17 | 44859410 | 3.21E-07 | 0.015441 | 0.827824 | 5.58E-08 | 0.006248 | 0.910642 |
|  | rs199506 | 17 | 44859031 | 4.81E-07 | 0.010436 | 0.930605 | 4.34E-08 | 0.007967 | 0.869355 |
|  | rs199507 | 17 | 44858855 | 3.67E-07 | 0.013541 | 0.870766 | 5.79E-08 | 0.006025 | 0.928888 |
|  | rs199509 | 17 | 44858728 | 3.39E-07 | 0.01464 | 0.857225 | 5.79E-08 | 0.006023 | 0.934911 |
|  | rs199510 | 17 | 44857929 | 2.8E-07 | 0.017593 | 0.728535 | 6.53E-08 | 0.005359 | 0.962927 |
|  | rs199512 | 17 | 44857352 | 1.8E-07 | 0.026947 | 0.608748 | 6.34E-08 | 0.005513 | 0.957568 |
|  | rs199513 | 17 | 44856932 | 2.23E-07 | 0.02191 | 0.630658 | 7.54E-08 | 0.004667 | 0.992647 |
|  | rs199514 | 17 | 44856881 | 2.31E-07 | 0.021221 | 0.673252 | 7.24E-08 | 0.004853 | 0.983242 |
|  | rs199515 | 17 | 44856641 | 1.3E-07 | 0.037016 | 0.550469 | 6.9E-08 | 0.005084 | 0.973358 |
|  | rs199516 | 17 | 44856485 | 3.36E-07 | 0.014762 | 0.842585 | 6.55E-08 | 0.005347 | 0.968274 |
|  | rs199525 | 17 | 44847834 | 8.86E-07 | 0.005792 | 0.970847 | 5.42E-08 | 0.006426 | 0.904394 |
|  | rs199533 | 17 | 44828931 | 7.16E-08 | 0.065783 | 0.464385 | 3.93E-08 | 0.008762 | 0.861388 |
|  | rs199534 | 17 | 44824213 | 5.16E-07 | 0.009756 | 0.950756 | 5.8E-08 | 0.006018 | 0.940929 |
|  | rs199535 | 17 | 44822662 | 6.24E-07 | 0.008121 | 0.958877 | 5.76E-08 | 0.006052 | 0.922863 |
|  | rs415430 | 17 | 44859144 | 2.85E-07 | 0.017283 | 0.745818 | 6.28E-08 | 0.005565 | 0.946494 |
|  | rs430685 | 17 | 44859148 | 3E-07 | 0.016438 | 0.795992 | 6.29E-08 | 0.005561 | 0.952055 |
|  | rs538628 | 17 | 44787313 | 2.16E-08 | 0.210784 | 0.210784 | 3.27E-08 | 0.010492 | 0.804707 |
|  | rs70600 | 17 | 44860021 | 2.9E-07 | 0.017029 | 0.762847 | 5.28E-08 | 0.006583 | 0.897968 |
|  | rs70602 | 17 | 44859715 | 3.01E-07 | 0.01639 | 0.812382 | 4.87E-08 | 0.007121 | 0.884348 |
|  | rs9896243 | 17 | 44826056 | 4.55E-07 | 0.011018 | 0.920168 | 1.67E-08 | 0.020083 | 0.662882 |

Abbreviations: probNorm refers to the normalized posterior p-value for each SNP. cumSum means the cumulative normalized posterior p-value for SNP sets. For each sentinel SNP, a 99% credible set of potentially causal SNPs was obtained using the FM-summary method. Each SNP has a corresponding posterior probability, i.e., probNorm, and with each superimposition of another SNP, there is a subsequent cumSum, which is obtained by the the corresponding probNorm of each SNP is summed up. Ultimately, this can be interpreted to mean that there is a 99% probability that the causal SNPs that actually have an effect on the two traits are contained in such a credible set.

## Supplementary Table 8. Fine-mapping 99% credible-set of sentinel SNP from cross-trait meta-analysis between educational attainment (EA) and bipolar disorder (BIP)

| Sentinel.SNP | Credible-set SNPs | CHR | BP | EA | | | BIP | | |
| --- | --- | --- | --- | --- | --- | --- | --- | --- | --- |
|  |  |  |  | **GWAS-p-value** | **probNorm** | **cumSum** | **GWAS-p-value** | **probNorm** | **cumSum** |
| rs10429537 | rs10429537 | 9 | 23346842 | 1.02E-21 | 0.009243 | 0.988642 | 1.21E-06 | 0.095547 | 0.095547 |
|  | rs10429582 | 9 | 23346850 | 1.16E-21 | 0.008086 | 0.996727 | 1.38E-06 | 0.084299 | 0.359185 |
|  | rs11793831 | 9 | 23362311 | 5.95E-22 | 0.01569 | 0.964449 | 2.61E-06 | 0.045573 | 0.903682 |
|  | rs11794152 | 9 | 23345347 | 6.24E-22 | 0.014949 | 0.979398 | 2.13E-06 | 0.055492 | 0.662027 |
|  | rs12553324 | 9 | 23347865 | 4.46E-22 | 0.020851 | 0.948759 | 1.3E-06 | 0.088968 | 0.274886 |
|  | rs12554512 | 9 | 23352293 | 1.94E-22 | 0.047634 | 0.898224 | 1.28E-06 | 0.090371 | 0.185919 |
|  | rs13294439 | 9 | 23358875 | 6.63E-23 | 0.137616 | 0.429198 | 2.55E-06 | 0.046672 | 0.858109 |
|  | rs1590949 | 9 | 23360417 | 3.12E-22 | 0.029684 | 0.927908 | 2.04E-06 | 0.057819 | 0.549969 |
|  | rs4977836 | 9 | 23355664 | 9.95E-23 | 0.092041 | 0.85059 | 2.08E-06 | 0.056566 | 0.606535 |
|  | rs4977839 | 9 | 23355310 | 9.39E-23 | 0.097502 | 0.661974 | 1.82E-06 | 0.064598 | 0.49215 |
|  | rs7029201 | 9 | 23358081 | 6.14E-23 | 0.148583 | 0.148583 | 2.53E-06 | 0.046884 | 0.811437 |
|  | rs7029718 | 9 | 23358495 | 6.38E-23 | 0.142999 | 0.291582 | 2.51E-06 | 0.047333 | 0.764553 |
|  | rs7467480 | 9 | 23354940 | 9.48E-23 | 0.096576 | 0.75855 | 1.71E-06 | 0.068367 | 0.427552 |
|  | rs7868984 | 9 | 23357826 | 6.75E-23 | 0.135273 | 0.564471 | 2.14E-06 | 0.055193 | 0.71722 |
| rs12754946 | rs12401738 | 1 | 78446761 | 5.18E-07 | 0.313704 | 0.313704 | 7.71E-06 | 0.035946 | 0.919578 |
|  | rs12402928 | 1 | 78053228 | 5.68E-06 | 0.031401 | 0.927878 | 2.96E-05 | 0.00997 | 0.964509 |
|  | rs12409142 | 1 | 78156975 | 2.51E-05 | 0.007606 | 0.987478 | 2.68E-05 | 0.010959 | 0.944193 |
|  | rs12409958 | 1 | 78314478 | 5.1E-06 | 0.034805 | 0.831535 | 4.51E-06 | 0.060009 | 0.845178 |
|  | rs12728967 | 1 | 78134934 | 2.95E-05 | 0.006521 | 0.993998 | 2.13E-05 | 0.013656 | 0.933234 |
|  | rs12741347 | 1 | 78152792 | 5.53E-06 | 0.03221 | 0.896477 | 4.42E-05 | 0.006805 | 0.988956 |
|  | rs12742653 | 1 | 78339481 | 1.63E-05 | 0.011453 | 0.979872 | 2.43E-06 | 0.108441 | 0.72042 |
|  | rs12754946 | 1 | 77989923 | 1.77E-06 | 0.096149 | 0.719977 | 2.39E-06 | 0.110356 | 0.61198 |
|  | rs1539738 | 1 | 78305492 | 4.34E-06 | 0.040638 | 0.760616 | 4.17E-06 | 0.064748 | 0.785168 |
|  | rs4130548 | 1 | 78463868 | 5.24E-07 | 0.310125 | 0.623828 | 7.18E-06 | 0.038455 | 0.883632 |
|  | rs4949660 | 1 | 78025459 | 6.9E-06 | 0.026071 | 0.95395 | 2.85E-05 | 0.010346 | 0.954539 |
|  | rs4949662 | 1 | 78080275 | 5.44E-06 | 0.032732 | 0.864267 | 5.47E-05 | 0.005564 | 0.99452 |
|  | rs6678622 | 1 | 78092479 | 4.91E-06 | 0.036114 | 0.79673 | 3.54E-05 | 0.008404 | 0.982151 |
|  | rs9729667 | 1 | 78349214 | 1.28E-05 | 0.014469 | 0.968419 | 1.42E-06 | 0.181624 | 0.501623 |
| rs9320913 | rs12202969 | 6 | 98576223 | 3.57E-21 | 0.074162 | 0.851714 | 1.02E-07 | 0.147208 | 0.320117 |
|  | rs12206087 | 6 | 98582900 | 4.24E-21 | 0.062488 | 0.914202 | 1.35E-07 | 0.111864 | 0.669851 |
|  | rs1487441 | 6 | 98553894 | 2.48E-21 | 0.106317 | 0.512081 | 4.43E-07 | 0.035575 | 0.962763 |
|  | rs1487445 | 6 | 98565211 | 2.12E-21 | 0.123955 | 0.405763 | 2.09E-07 | 0.073314 | 0.851617 |
|  | rs1906252 | 6 | 98550289 | 5.89E-21 | 0.045126 | 0.959329 | 6.62E-07 | 0.024134 | 0.986897 |
|  | rs2388334 | 6 | 98591622 | 6.56E-21 | 0.040578 | 0.999907 | 8.62E-08 | 0.172909 | 0.172909 |
|  | rs9320913 | 6 | 98584733 | 2.05E-21 | 0.128325 | 0.281808 | 1.27E-07 | 0.118754 | 0.557987 |
|  | rs9372734 | 6 | 98577689 | 2.97E-21 | 0.088755 | 0.692205 | 1.27E-07 | 0.119117 | 0.439233 |
|  | rs9375188 | 6 | 98555272 | 3.09E-21 | 0.085348 | 0.777553 | 4.24E-07 | 0.037071 | 0.927188 |
|  | rs9401593 | 6 | 98549801 | 1.71E-21 | 0.153483 | 0.153483 | 4.08E-07 | 0.0385 | 0.890117 |
|  | rs968050 | 6 | 98574560 | 2.89E-21 | 0.091369 | 0.60345 | 1.4E-07 | 0.108452 | 0.778303 |

Abbreviations: probNorm refers to the normalized posterior p-value for each SNP. cumSum means the cumulative normalized posterior p-value for SNP sets. For each sentinel SNP, a 99% credible set of potentially causal SNPs was obtained using the FM-summary method. Each SNP has a corresponding posterior probability, i.e., probNorm, and with each superimposition of another SNP, there is a subsequent cumSum, which is obtained by the the corresponding probNorm of each SNP is summed up. Ultimately, this can be interpreted to mean that there is a 99% probability that the causal SNPs that actually have an effect on the two traits are contained in such a credible set.

## Supplementary Table 9. Fine-mapping 99% credible-set of sentinel SNP from cross-trait meta-analysis between educational attainment (EA) and cannabis use disorder (CUD)

| Sentinel.SNP | Credible-set SNPs | CHR | BP | EA | | | CUD | | |
| --- | --- | --- | --- | --- | --- | --- | --- | --- | --- |
|  |  |  |  | **GWAS-p-value** | **probNorm** | **cumSum** | **GWAS-p-value** | **probNorm** | **cumSum** |
| rs12122664 | rs10801825 | 1 | 91206170 | 6.13E-10 | 0.048377 | 0.992065 | 3.2E-05 | 0.011358 | 0.914403 |
|  | rs10801826 | 1 | 91206578 | 4.26E-10 | 0.069015 | 0.571227 | 3.56E-05 | 0.010281 | 0.935724 |
|  | rs10922911 | 1 | 91205831 | 4.12E-10 | 0.071355 | 0.502212 | 2.9E-05 | 0.012496 | 0.866741 |
|  | rs10922912 | 1 | 91207367 | 4.7E-10 | 0.062737 | 0.768282 | 3.58E-05 | 0.010213 | 0.956177 |
|  | rs1526480 | 1 | 91209986 | 4.32E-10 | 0.068125 | 0.639351 | 2.18E-05 | 0.016388 | 0.607002 |
|  | rs2166171 | 1 | 91208451 | 4.81E-10 | 0.06131 | 0.891141 | 2.76E-05 | 0.013063 | 0.841249 |
|  | rs2166172 | 1 | 91208514 | 4.07E-10 | 0.072211 | 0.430857 | 3.3E-05 | 0.01104 | 0.925443 |
|  | rs6699397 | 1 | 91212216 | 5.63E-10 | 0.052548 | 0.943688 | 7.93E-05 | 0.004805 | 0.991665 |
|  | rs7548936 | 1 | 91207757 | 4.79E-10 | 0.061548 | 0.82983 | 2.66E-05 | 0.013529 | 0.815051 |
| rs35926495 | rs11919418 | 3 | 50233215 | 2.36E-17 | 0.014445 | 0.955119 | 3.66E-07 | 0.045552 | 0.679781 |
|  | rs12632110 | 3 | 50224225 | 2.74E-18 | 0.121003 | 0.258833 | 4.36E-07 | 0.038488 | 0.80451 |
|  | rs12637671 | 3 | 50224562 | 3.73E-17 | 0.009187 | 0.989312 | 7.93E-07 | 0.021639 | 0.9636 |
|  | rs13059311 | 3 | 50216421 | 4.7E-18 | 0.070867 | 0.586454 | 7.51E-07 | 0.022805 | 0.919998 |
|  | rs13064381 | 3 | 50233317 | 1.65E-17 | 0.0205 | 0.889345 | 3.67E-07 | 0.045516 | 0.725298 |
|  | rs13067082 | 3 | 50221715 | 1.02E-17 | 0.03314 | 0.844659 | 1.07E-06 | 0.016285 | 0.997723 |
|  | rs2236940 | 3 | 50244080 | 5.62E-18 | 0.059494 | 0.645948 | 3.59E-07 | 0.046509 | 0.634229 |
|  | rs2236941 | 3 | 50247824 | 8.84E-18 | 0.038007 | 0.811519 | 7.81E-07 | 0.021963 | 0.941961 |
|  | rs2526390 | 3 | 50192760 | 1.81E-17 | 0.018714 | 0.908059 | 6.19E-07 | 0.027446 | 0.897194 |
|  | rs2624835 | 3 | 50209049 | 7.82E-18 | 0.042921 | 0.732506 | 1.94E-07 | 0.08408 | 0.08408 |
|  | rs2624838 | 3 | 50205642 | 3.09E-18 | 0.107444 | 0.366276 | 5.95E-07 | 0.028535 | 0.869748 |
|  | rs2624841 | 3 | 50198415 | 2.4E-18 | 0.137829 | 0.137829 | 3.42E-07 | 0.048686 | 0.58772 |
|  | rs35137368 | 3 | 50243948 | 2.64E-17 | 0.012912 | 0.968031 | 2.92E-07 | 0.056659 | 0.385914 |
|  | rs35219849 | 3 | 50243801 | 4.06E-17 | 0.008454 | 0.997766 | 2.92E-07 | 0.056678 | 0.329254 |
|  | rs58648044 | 3 | 50248954 | 7.69E-18 | 0.043637 | 0.689585 | 4.11E-07 | 0.040724 | 0.766022 |
|  | rs6807194 | 3 | 50236373 | 1.89E-17 | 0.017924 | 0.925983 | 3.33E-07 | 0.049884 | 0.48938 |
|  | rs9858297 | 3 | 50218879 | 8.19E-18 | 0.041006 | 0.773512 | 2.45E-07 | 0.067276 | 0.151355 |
| rs7783012 | rs1015511 | 7 | 1.14E+08 | 6.15E-07 | 0.043895 | 0.619447 | 1.3E-08 | 0.027408 | 0.84883 |
|  | rs10249234 | 7 | 1.14E+08 | 6.23E-07 | 0.043325 | 0.749677 | 4.37E-09 | 0.079048 | 0.821422 |
|  | rs10262103 | 7 | 1.14E+08 | 2.65E-05 | 0.001194 | 0.988752 | 2.94E-08 | 0.012423 | 0.902408 |
|  | rs10262462 | 7 | 1.14E+08 | 5.29E-07 | 0.050765 | 0.431341 | 1.69E-07 | 0.002276 | 0.986411 |
|  | rs10266297 | 7 | 1.14E+08 | 6.11E-07 | 0.044193 | 0.575552 | 1.66E-08 | 0.021678 | 0.870508 |
|  | rs10280045 | 7 | 1.14E+08 | 1.19E-05 | 0.002566 | 0.974381 | 5.33E-08 | 0.006968 | 0.943572 |
|  | rs12705966 | 7 | 1.14E+08 | 4.15E-06 | 0.007003 | 0.966255 | 1.26E-07 | 0.00303 | 0.981776 |
|  | rs1989903 | 7 | 1.14E+08 | 6.31E-07 | 0.042855 | 0.792532 | 3.52E-09 | 0.097632 | 0.562342 |
|  | rs2040658 | 7 | 1.14E+08 | 6.36E-07 | 0.042504 | 0.835036 | 3.88E-09 | 0.088872 | 0.742374 |
|  | rs2045292 | 7 | 1.14E+08 | 2.73E-06 | 0.010467 | 0.959252 | 9.29E-08 | 0.004068 | 0.972007 |
|  | rs2189010 | 7 | 1.14E+08 | 5.3E-07 | 0.0507 | 0.482041 | 3.78E-09 | 0.091161 | 0.653502 |
|  | rs2189012 | 7 | 1.14E+08 | 1.02E-05 | 0.00296 | 0.969216 | 1.99E-07 | 0.001944 | 0.990366 |
|  | rs2189015 | 7 | 1.14E+08 | 4.65E-07 | 0.057489 | 0.214396 | 8.95E-08 | 0.004217 | 0.96794 |
|  | rs2396753 | 7 | 1.14E+08 | 5.45E-07 | 0.049318 | 0.531359 | 7.93E-08 | 0.004741 | 0.959089 |
|  | rs4727799 | 7 | 1.14E+08 | 2.78E-07 | 0.094334 | 0.094334 | 1.85E-08 | 0.019476 | 0.889985 |
|  | rs6466488 | 7 | 1.14E+08 | 8.48E-07 | 0.032199 | 0.948784 | 6.44E-08 | 0.005805 | 0.949378 |
|  | rs6969376 | 7 | 1.14E+08 | 5.19E-07 | 0.051698 | 0.380576 | 1.07E-07 | 0.003556 | 0.975563 |
|  | rs6974757 | 7 | 1.14E+08 | 6.22E-07 | 0.043439 | 0.706352 | 1.89E-09 | 0.17914 | 0.36332 |
|  | rs6980093 | 7 | 1.14E+08 | 4.26E-07 | 0.062573 | 0.156907 | 1.2E-07 | 0.003184 | 0.978747 |
|  | rs727644 | 7 | 1.14E+08 | 4.65E-07 | 0.057477 | 0.271873 | 3.39E-09 | 0.10139 | 0.46471 |
|  | rs7458242 | 7 | 1.14E+08 | 6.69E-07 | 0.040452 | 0.916585 | 1.93E-07 | 0.002011 | 0.988422 |
|  | rs7783012 | 7 | 1.14E+08 | 4.69E-07 | 0.057004 | 0.328877 | 1.84E-09 | 0.18418 | 0.18418 |
|  | rs8180817 | 7 | 1.14E+08 | 3.19E-05 | 0.001001 | 0.990865 | 3.72E-08 | 0.009878 | 0.912285 |
|  | rs9969232 | 7 | 1.14E+08 | 6.21E-07 | 0.043466 | 0.662913 | 1.63E-07 | 0.002359 | 0.984136 |
| rs9467773 | rs1001687 | 6 | 26573218 | 2.67E-05 | 0.003771 | 0.915127 | 1.41E-05 | 0.005073 | 0.875773 |
|  | rs10214634 | 6 | 26564582 | 1.44E-05 | 0.006808 | 0.779212 | 6.41E-06 | 0.010758 | 0.174227 |
|  | rs10223789 | 6 | 26579609 | 3.79E-05 | 0.002703 | 0.963293 | 1.46E-05 | 0.004914 | 0.915522 |
|  | rs10223792 | 6 | 26579616 | 3.86E-05 | 0.00266 | 0.965952 | 1.45E-05 | 0.00492 | 0.910609 |
|  | rs10484442 | 6 | 26555879 | 2.17E-05 | 0.004599 | 0.854625 | 7.54E-06 | 0.009206 | 0.599593 |
|  | rs1056347 | 6 | 26527524 | 7.94E-06 | 0.012 | 0.408148 | 6.74E-06 | 0.010249 | 0.246704 |
|  | rs1056667 | 6 | 26510564 | 6.56E-06 | 0.014392 | 0.291213 | 1.02E-05 | 0.006916 | 0.754251 |
|  | rs1056668 | 6 | 26510605 | 2.29E-05 | 0.004366 | 0.867968 | 1.64E-05 | 0.004381 | 0.962426 |
|  | rs1078679 | 6 | 26568741 | 1.09E-05 | 0.008855 | 0.603381 | 6.28E-06 | 0.010963 | 0.152609 |
|  | rs10946834 | 6 | 26533664 | 8.75E-06 | 0.010937 | 0.546888 | 6.76E-06 | 0.010214 | 0.277363 |
|  | rs10946835 | 6 | 26533757 | 9.1E-06 | 0.010529 | 0.557417 | 6.93E-06 | 0.009984 | 0.388493 |
|  | rs10946837 | 6 | 26540844 | 4.07E-05 | 0.002527 | 0.9711 | 1.53E-05 | 0.00469 | 0.953384 |
|  | rs11752946 | 6 | 26560364 | 5.01E-05 | 0.002077 | 0.984807 | 1.37E-05 | 0.005219 | 0.839739 |
|  | rs11754138 | 6 | 26526941 | 8.52E-06 | 0.011215 | 0.524741 | 6.74E-06 | 0.01025 | 0.236455 |
|  | rs11756120 | 6 | 26529808 | 7.98E-06 | 0.011932 | 0.444028 | 6.56E-06 | 0.010524 | 0.195292 |
|  | rs12525684 | 6 | 26483910 | 2.36E-05 | 0.004244 | 0.872212 | 8.82E-06 | 0.007922 | 0.703287 |
|  | rs12526680 | 6 | 26550954 | 1E-05 | 0.009586 | 0.567003 | 5.64E-06 | 0.012146 | 0.038256 |
|  | rs12663883 | 6 | 26577326 | 2.69E-05 | 0.003751 | 0.918878 | 1.37E-05 | 0.005211 | 0.84495 |
|  | rs12663894 | 6 | 26577370 | 3.18E-05 | 0.0032 | 0.93965 | 1.67E-05 | 0.004319 | 0.971113 |
|  | rs12665431 | 6 | 26577308 | 2.85E-05 | 0.003543 | 0.929687 | 1.54E-05 | 0.004661 | 0.958045 |
|  | rs1321479 | 6 | 26501897 | 6.95E-06 | 0.013625 | 0.319099 | 1.08E-05 | 0.006524 | 0.794232 |
|  | rs1321480 | 6 | 26532742 | 8.52E-06 | 0.01121 | 0.535951 | 7.47E-06 | 0.009292 | 0.581104 |
|  | rs1321481 | 6 | 26538210 | 1.71E-05 | 0.005755 | 0.828777 | 6.97E-06 | 0.009929 | 0.428266 |
|  | rs1321482 | 6 | 26575154 | 2.6E-05 | 0.003865 | 0.903659 | 1.42E-05 | 0.005036 | 0.880809 |
|  | rs1407045 | 6 | 26476155 | 2.18E-05 | 0.004575 | 0.8592 | 9.39E-06 | 0.007461 | 0.726158 |
|  | rs1535277 | 6 | 26567802 | 1.43E-05 | 0.006849 | 0.772404 | 5.73E-06 | 0.01198 | 0.062254 |
|  | rs1570059 | 6 | 26573325 | 2.82E-05 | 0.00358 | 0.926143 | 1.38E-05 | 0.005164 | 0.855297 |
|  | rs1570060 | 6 | 26573562 | 2.6E-05 | 0.003866 | 0.899794 | 1.38E-05 | 0.005157 | 0.865612 |
|  | rs1570061 | 6 | 26575986 | 2.6E-05 | 0.003865 | 0.907523 | 1.4E-05 | 0.005087 | 0.870699 |
|  | rs1796520 | 6 | 26410800 | 7.92E-05 | 0.001346 | 0.990857 | 1.22E-05 | 0.005836 | 0.812963 |
|  | rs1884947 | 6 | 26553273 | 4.13E-05 | 0.002491 | 0.973592 | 1.33E-05 | 0.005349 | 0.829247 |
|  | rs1884948 | 6 | 26567988 | 1.02E-05 | 0.009442 | 0.576445 | 5.85E-06 | 0.011727 | 0.085725 |
|  | rs1884949 | 6 | 26568067 | 1.15E-05 | 0.00842 | 0.637324 | 5.85E-06 | 0.011744 | 0.073998 |
|  | rs1977201 | 6 | 26573674 | 2.52E-05 | 0.003984 | 0.880255 | 1.38E-05 | 0.005157 | 0.860455 |
|  | rs2024970 | 6 | 26497520 | 3.2E-05 | 0.003179 | 0.942829 | 3.11E-05 | 0.002382 | 0.980982 |
|  | rs2145318 | 6 | 26496603 | 1.32E-05 | 0.007388 | 0.723474 | 9.79E-06 | 0.007175 | 0.733332 |
|  | rs2208331 | 6 | 26507319 | 5.6E-06 | 0.016764 | 0.170938 | 1.02E-05 | 0.00689 | 0.761141 |
|  | rs2224380 | 6 | 26553943 | 1.67E-05 | 0.005903 | 0.823022 | 7.16E-06 | 0.009674 | 0.496067 |
|  | rs2255070 | 6 | 26501777 | 6.31E-06 | 0.014935 | 0.232906 | 7.46E-06 | 0.009304 | 0.571811 |
|  | rs2393669 | 6 | 26503873 | 4.12E-06 | 0.022467 | 0.077476 | 1.08E-05 | 0.006547 | 0.781166 |
|  | rs2393670 | 6 | 26535541 | 1.26E-05 | 0.007735 | 0.685326 | 6.79E-06 | 0.010174 | 0.307962 |
|  | rs28558133 | 6 | 26531433 | 2.53E-05 | 0.003968 | 0.884223 | 6.76E-06 | 0.010213 | 0.287575 |
|  | rs35355150 | 6 | 26526039 | 2.63E-05 | 0.003833 | 0.911356 | 1.51E-05 | 0.004758 | 0.925108 |
|  | rs3734540 | 6 | 26463321 | 2.47E-05 | 0.004059 | 0.876271 | 5.05E-06 | 0.013521 | 0.013521 |
|  | rs3736781 | 6 | 26505362 | 7.45E-06 | 0.012747 | 0.371836 | 8.81E-06 | 0.007933 | 0.695365 |
|  | rs3736782 | 6 | 26505403 | 5.86E-06 | 0.016048 | 0.186986 | 1.1E-05 | 0.006411 | 0.807128 |
|  | rs3757151 | 6 | 26498165 | 5.99E-06 | 0.015714 | 0.202701 | 1.04E-05 | 0.006751 | 0.767892 |
|  | rs4573 | 6 | 26546808 | 4.17E-05 | 0.002469 | 0.97606 | 1.52E-05 | 0.004716 | 0.943991 |
|  | rs4713006 | 6 | 26519872 | 7.81E-06 | 0.012194 | 0.38403 | 7.73E-06 | 0.00899 | 0.617694 |
|  | rs4713008 | 6 | 26538268 | 4.53E-06 | 0.02052 | 0.097995 | 6.96E-06 | 0.009935 | 0.418337 |
|  | rs4871 | 6 | 26545632 | 1.38E-05 | 0.007095 | 0.73057 | 7.79E-06 | 0.008924 | 0.644502 |
|  | rs55930917 | 6 | 26577186 | 7.84E-05 | 0.001359 | 0.98951 | 3.35E-05 | 0.002223 | 0.983205 |
|  | rs6456733 | 6 | 26566804 | 1.39E-05 | 0.007027 | 0.744668 | 5.9E-06 | 0.011641 | 0.097366 |
|  | rs6456734 | 6 | 26566965 | 1.84E-05 | 0.005393 | 0.839779 | 5.44E-06 | 0.012589 | 0.02611 |
|  | rs6456735 | 6 | 26574149 | 2.27E-05 | 0.004402 | 0.863602 | 9.03E-06 | 0.007746 | 0.711033 |
|  | rs6903973 | 6 | 26499942 | 5E-06 | 0.018669 | 0.136813 | 1.3E-05 | 0.005487 | 0.81845 |
|  | rs6909277 | 6 | 26571537 | 3.92E-05 | 0.002621 | 0.968573 | 5.71E-06 | 0.012018 | 0.050274 |
|  | rs6910899 | 6 | 26524339 | 8.51E-06 | 0.011233 | 0.513526 | 7.25E-06 | 0.009552 | 0.524796 |
|  | rs6910930 | 6 | 26524061 | 8.45E-06 | 0.011304 | 0.502294 | 7.22E-06 | 0.009596 | 0.505663 |
|  | rs6913398 | 6 | 26540593 | 1.39E-05 | 0.007027 | 0.751695 | 7.33E-06 | 0.009456 | 0.534252 |
|  | rs6913462 | 6 | 26577530 | 2.55E-05 | 0.003943 | 0.888165 | 1.48E-05 | 0.004828 | 0.920351 |
|  | rs6913877 | 6 | 26577488 | 3.11E-05 | 0.00326 | 0.93645 | 1.44E-05 | 0.004966 | 0.895816 |
|  | rs6918360 | 6 | 26577867 | 3.36E-05 | 0.003032 | 0.948937 | 1.52E-05 | 0.004722 | 0.934552 |
|  | rs6918506 | 6 | 26577857 | 3.31E-05 | 0.003076 | 0.945904 | 1.52E-05 | 0.004722 | 0.92983 |
|  | rs6918854 | 6 | 26577924 | 2.6E-05 | 0.003866 | 0.895928 | 1.65E-05 | 0.004369 | 0.966795 |
|  | rs6922824 | 6 | 26553815 | 1.85E-05 | 0.005343 | 0.845121 | 7.14E-06 | 0.009695 | 0.447664 |
|  | rs6924838 | 6 | 26571756 | 1.14E-05 | 0.008484 | 0.628904 | 6.54E-06 | 0.010541 | 0.184768 |
|  | rs6924865 | 6 | 26521353 | 1.17E-05 | 0.008297 | 0.645621 | 8.21E-06 | 0.008486 | 0.679036 |
|  | rs6925703 | 6 | 26521589 | 8.23E-06 | 0.011587 | 0.49099 | 7.93E-06 | 0.008772 | 0.653274 |
|  | rs6925783 | 6 | 26570410 | 1.26E-05 | 0.007729 | 0.693055 | 6.34E-06 | 0.01086 | 0.163469 |
|  | rs6926629 | 6 | 26499903 | 3.77E-06 | 0.024492 | 0.055009 | 1.01E-05 | 0.006975 | 0.747335 |
|  | rs6930120 | 6 | 26555484 | 2.03E-05 | 0.004905 | 0.850026 | 7.41E-06 | 0.009359 | 0.562507 |
|  | rs6932156 | 6 | 26571506 | 1.24E-05 | 0.007842 | 0.669856 | 6.26E-06 | 0.011001 | 0.130648 |
|  | rs6932350 | 6 | 26571629 | 5.57E-05 | 0.001877 | 0.986683 | 1.09E-05 | 0.006484 | 0.800716 |
|  | rs6932865 | 6 | 26540166 | 1.26E-05 | 0.007723 | 0.700778 | 7.16E-06 | 0.009677 | 0.486393 |
|  | rs6933176 | 6 | 26540178 | 1.26E-05 | 0.007735 | 0.677591 | 7.16E-06 | 0.009677 | 0.476716 |
|  | rs6940053 | 6 | 26562122 | 1.38E-05 | 0.007071 | 0.737641 | 6.8E-06 | 0.010155 | 0.328288 |
|  | rs6940188 | 6 | 26562029 | 1.56E-05 | 0.006284 | 0.805123 | 6.94E-06 | 0.009968 | 0.398461 |
|  | rs6941022 | 6 | 26553531 | 1.76E-05 | 0.005608 | 0.834386 | 7.15E-06 | 0.009689 | 0.457353 |
|  | rs767471 | 6 | 26557854 | 1.26E-05 | 0.007712 | 0.70849 | 7.47E-06 | 0.009283 | 0.590387 |
|  | rs7753565 | 6 | 26560012 | 4.66E-05 | 0.002222 | 0.980535 | 6.74E-06 | 0.01025 | 0.226205 |
|  | rs9295694 | 6 | 26512994 | 7.11E-06 | 0.013335 | 0.345924 | 8.06E-06 | 0.008634 | 0.67055 |
|  | rs9295695 | 6 | 26528250 | 7.86E-06 | 0.012118 | 0.396148 | 6.87E-06 | 0.010062 | 0.348487 |
|  | rs9295698 | 6 | 26566099 | 1.47E-05 | 0.006671 | 0.785882 | 6.26E-06 | 0.010998 | 0.141646 |
|  | rs9295699 | 6 | 26566409 | 3.46E-05 | 0.002947 | 0.957837 | 6.2E-06 | 0.011095 | 0.119647 |
|  | rs9357010 | 6 | 26527945 | 8.16E-06 | 0.011688 | 0.479403 | 6.92E-06 | 0.009991 | 0.378509 |
|  | rs9358952 | 6 | 26516769 | 6.17E-06 | 0.01527 | 0.217971 | 7.76E-06 | 0.008954 | 0.626648 |
|  | rs9358954 | 6 | 26531178 | 7.96E-06 | 0.011961 | 0.432096 | 6.66E-06 | 0.010368 | 0.20566 |
|  | rs9379887 | 6 | 26518662 | 6.63E-06 | 0.014261 | 0.305474 | 7.78E-06 | 0.00893 | 0.635579 |
|  | rs9393728 | 6 | 26509330 | 4.62E-06 | 0.020149 | 0.118145 | 0.00001 | 0.007028 | 0.74036 |
|  | rs9393729 | 6 | 26514166 | 7.02E-06 | 0.013491 | 0.332589 | 8.3E-06 | 0.008396 | 0.687431 |
|  | rs9393731 | 6 | 26529374 | 8.04E-06 | 0.011853 | 0.455881 | 6.89E-06 | 0.010028 | 0.358515 |
|  | rs9393732 | 6 | 26530898 | 8.05E-06 | 0.011835 | 0.467715 | 6.76E-06 | 0.010213 | 0.297788 |
|  | rs9461259 | 6 | 26504835 | 6.51E-06 | 0.014508 | 0.276821 | 1.08E-05 | 0.006542 | 0.787708 |
|  | rs9461267 | 6 | 26525455 | 6.41E-06 | 0.014732 | 0.247638 | 7.33E-06 | 0.009456 | 0.543708 |
|  | rs9461270 | 6 | 26544110 | 1.64E-05 | 0.005999 | 0.81712 | 1.76E-05 | 0.004101 | 0.975214 |
|  | rs9461271 | 6 | 26554968 | 1.53E-05 | 0.006405 | 0.798839 | 7.15E-06 | 0.009686 | 0.467039 |
|  | rs9461272 | 6 | 26579648 | 3.72E-05 | 0.002752 | 0.96059 | 1.45E-05 | 0.00492 | 0.905688 |
|  | rs9467773 | 6 | 26498426 | 3E-06 | 0.030517 | 0.030517 | 9.13E-06 | 0.007664 | 0.718697 |
|  | rs9467774 | 6 | 26505036 | 6.43E-06 | 0.014675 | 0.262312 | 1.05E-05 | 0.006726 | 0.774619 |
|  | rs9467775 | 6 | 26513435 | 7.2E-06 | 0.013165 | 0.359089 | 8.06E-06 | 0.008642 | 0.661916 |
|  | rs9467776 | 6 | 26531954 | 5.4E-06 | 0.017361 | 0.154175 | 7.34E-06 | 0.00944 | 0.553148 |
|  | rs9467777 | 6 | 26534616 | 7.23E-05 | 0.001468 | 0.988151 | 6.76E-06 | 0.010226 | 0.25693 |
|  | rs9467778 | 6 | 26534743 | 1.14E-05 | 0.008527 | 0.611908 | 6.82E-06 | 0.010137 | 0.338425 |
|  | rs9467779 | 6 | 26536687 | 1.07E-05 | 0.009012 | 0.594526 | 6.91E-06 | 0.010003 | 0.368518 |
|  | rs9467782 | 6 | 26542773 | 1.5E-05 | 0.006552 | 0.792434 | 7.23E-06 | 0.009581 | 0.515244 |
|  | rs9467783 | 6 | 26542894 | 1.41E-05 | 0.006928 | 0.765555 | 6.96E-06 | 0.009942 | 0.408402 |
|  | rs9467787 | 6 | 26556769 | 2.58E-05 | 0.003896 | 0.892062 | 6.79E-06 | 0.010171 | 0.318133 |
|  | rs9467791 | 6 | 26562486 | 1.28E-05 | 0.007597 | 0.716086 | 7.14E-06 | 0.009703 | 0.437969 |
|  | rs9467796 | 6 | 26568411 | 1.19E-05 | 0.008137 | 0.662014 | 6.15E-06 | 0.011186 | 0.108552 |
|  | rs9467797 | 6 | 26568473 | 1.06E-05 | 0.009069 | 0.585514 | 6.71E-06 | 0.010294 | 0.215955 |
|  | rs9467798 | 6 | 26575697 | 2.74E-05 | 0.003685 | 0.922563 | 1.42E-05 | 0.005016 | 0.89085 |
|  | rs9467799 | 6 | 26578364 | 2.89E-05 | 0.003502 | 0.933189 | 1.52E-05 | 0.004704 | 0.948695 |
|  | rs9467800 | 6 | 26578525 | 3.44E-05 | 0.002963 | 0.954891 | 1.52E-05 | 0.004722 | 0.939274 |
|  | rs9467802 | 6 | 26581168 | 3.41E-05 | 0.002991 | 0.951928 | 1.42E-05 | 0.005026 | 0.885834 |
|  | rs9986382 | 6 | 26550619 | 1.17E-05 | 0.008256 | 0.653877 | 7.62E-06 | 0.009112 | 0.608704 |

Abbreviations: probNorm refers to the normalized posterior p-value for each SNP. cumSum means the cumulative normalized posterior p-value for SNP sets. For each sentinel SNP, a 99% credible set of potentially causal SNPs was obtained using the FM-summary method. Each SNP has a corresponding posterior probability, i.e., probNorm, and with each superimposition of another SNP, there is a subsequent cumSum, which is obtained by the the corresponding probNorm of each SNP is summed up. Ultimately, this can be interpreted to mean that there is a 99% probability that the causal SNPs that actually have an effect on the two traits are contained in such a credible set.

## Supplementary Table 10. Fine-mapping 99% credible-set of sentinel SNP from cross-trait meta-analysis between educational attainment (EA) and major depressive disorder (MDD).

| Sentinel.SNP | Credible-set SNPs | CHR | BP | EA | | | MDD | | |
| --- | --- | --- | --- | --- | --- | --- | --- | --- | --- |
|  |  |  |  | **GWAS-p-value** | **probNorm** | **cumSum** | **GWAS-p-value** | **probNorm** | **cumSum** |
| rs7531118 | rs10789336 | 1 | 72838406 | 2.77E-06 | 0.015139 | 0.747971 | 9.09E-08 | 0.030306 | 0.831839 |
|  | rs10889947 | 1 | 72828221 | 2.3E-06 | 0.018117 | 0.666275 | 1.67E-07 | 0.016867 | 0.922489 |
|  | rs11209943 | 1 | 72750500 | 7.07E-07 | 0.056346 | 0.056346 | 3.28E-07 | 0.008774 | 0.990392 |
|  | rs11209948 | 1 | 72811904 | 2.03E-06 | 0.020388 | 0.61012 | 2.75E-07 | 0.010411 | 0.971306 |
|  | rs11209950 | 1 | 72829680 | 1.61E-06 | 0.025572 | 0.379559 | 8.32E-08 | 0.033036 | 0.801534 |
|  | rs11209951 | 1 | 72837490 | 2.29E-06 | 0.018185 | 0.648158 | 1.22E-07 | 0.02289 | 0.882894 |
|  | rs11209952 | 1 | 72837500 | 1.73E-06 | 0.02386 | 0.476932 | 9.81E-08 | 0.028164 | 0.860003 |
|  | rs1432639 | 1 | 72813218 | 1.31E-06 | 0.031174 | 0.353988 | 4.84E-08 | 0.055774 | 0.357495 |
|  | rs1460942 | 1 | 72813256 | 1.62E-06 | 0.025389 | 0.404948 | 4.54E-08 | 0.059391 | 0.245004 |
|  | rs1460943 | 1 | 72813129 | 1.71E-06 | 0.024075 | 0.429023 | 4.27E-08 | 0.063028 | 0.185614 |
|  | rs1841499 | 1 | 72836456 | 2.72E-06 | 0.015433 | 0.732832 | 8.02E-08 | 0.03422 | 0.768498 |
|  | rs2012697 | 1 | 72819612 | 2.46E-06 | 0.017003 | 0.70076 | 2.44E-07 | 0.011656 | 0.950309 |
|  | rs2568952 | 1 | 72755105 | 9.29E-07 | 0.043297 | 0.195927 | 7.77E-08 | 0.035303 | 0.665288 |
|  | rs2568956 | 1 | 72764376 | 8.73E-07 | 0.045979 | 0.152629 | 6.24E-08 | 0.043636 | 0.547851 |
|  | rs2568958 | 1 | 72765116 | 9.55E-07 | 0.042175 | 0.281251 | 5.95E-08 | 0.045703 | 0.504215 |
|  | rs2568960 | 1 | 72812249 | 1.74E-06 | 0.023689 | 0.500621 | 6.25E-08 | 0.043549 | 0.5914 |
|  | rs2568961 | 1 | 72812747 | 2E-06 | 0.020741 | 0.589732 | 4.76E-08 | 0.056716 | 0.301721 |
|  | rs2613494 | 1 | 72812657 | 2.09E-06 | 0.019853 | 0.629973 | 5.46E-08 | 0.049647 | 0.458512 |
|  | rs2815752 | 1 | 72812440 | 1.77E-06 | 0.023353 | 0.523974 | 5.27E-08 | 0.05137 | 0.408865 |
|  | rs2815753 | 1 | 72812324 | 1.71E-06 | 0.024048 | 0.453071 | 7.94E-08 | 0.03455 | 0.699837 |
|  | rs2815765 | 1 | 72752230 | 7.95E-07 | 0.050304 | 0.106651 | 2.77E-07 | 0.010313 | 0.981618 |
|  | rs3101336 | 1 | 72751185 | 9.69E-07 | 0.041563 | 0.322814 | 7.97E-08 | 0.03444 | 0.734278 |
|  | rs3101337 | 1 | 72751134 | 9.32E-07 | 0.04315 | 0.239076 | 7.09E-08 | 0.038585 | 0.629985 |
|  | rs3101339 | 1 | 72748669 | 1.83E-06 | 0.022556 | 0.54653 | 1.22E-07 | 0.022728 | 0.905622 |
|  | rs7531118 | 1 | 72837239 | 1.84E-06 | 0.022462 | 0.568991 | 2.15E-08 | 0.122586 | 0.122586 |
|  | rs990871 | 1 | 72823713 | 2.39E-06 | 0.017482 | 0.683757 | 2.7E-07 | 0.010586 | 0.960895 |
| rs76025409 | rs10052804 | 5 | 1.04E+08 | 0.001042 | 0.000594 | 0.957362 | 2.31E-09 | 0.003148 | 0.880311 |
|  | rs10053368 | 5 | 1.04E+08 | 0.001154 | 0.000541 | 0.965364 | 7.77E-10 | 0.009111 | 0.544842 |
|  | rs10053371 | 5 | 1.04E+08 | 0.000871 | 0.000702 | 0.95166 | 6.5E-10 | 0.010842 | 0.525367 |
|  | rs10054977 | 5 | 1.04E+08 | 0.001223 | 0.000512 | 0.968469 | 2.15E-09 | 0.00338 | 0.854371 |
|  | rs10057459 | 5 | 1.04E+08 | 0.001111 | 0.00056 | 0.96371 | 1.02E-09 | 0.006969 | 0.643928 |
|  | rs10057469 | 5 | 1.04E+08 | 0.001241 | 0.000505 | 0.96999 | 1.38E-09 | 0.005212 | 0.715739 |
|  | rs10059133 | 5 | 1.04E+08 | 0.002426 | 0.000273 | 0.989383 | 1.26E-09 | 0.005692 | 0.667615 |
|  | rs10059643 | 5 | 1.04E+08 | 0.001218 | 0.000514 | 0.967956 | 1.87E-09 | 0.003881 | 0.811083 |
|  | rs10064425 | 5 | 1.04E+08 | 0.002204 | 0.000298 | 0.986282 | 1.76E-09 | 0.004102 | 0.791132 |
|  | rs10071115 | 5 | 1.04E+08 | 0.001548 | 0.000412 | 0.978132 | 1.44E-09 | 0.004997 | 0.720736 |
|  | rs10072579 | 5 | 1.04E+08 | 0.001472 | 0.000432 | 0.975632 | 1.34E-09 | 0.005356 | 0.689419 |
|  | rs10072849 | 5 | 1.04E+08 | 0.001113 | 0.000559 | 0.964269 | 1.19E-09 | 0.006008 | 0.656138 |
|  | rs10078807 | 5 | 1.04E+08 | 0.001103 | 0.000564 | 0.96315 | 9.43E-10 | 0.007544 | 0.586823 |
|  | rs10477834 | 5 | 1.04E+08 | 0.001368 | 0.000462 | 0.973862 | 2.21E-09 | 0.003294 | 0.861021 |
|  | rs10479296 | 5 | 1.04E+08 | 0.001034 | 0.000598 | 0.956171 | 6.81E-10 | 0.010364 | 0.535731 |
|  | rs10479297 | 5 | 1.04E+08 | 0.001055 | 0.000587 | 0.958541 | 9.95E-10 | 0.007161 | 0.615666 |
|  | rs11242522 | 5 | 1.04E+08 | 0.002404 | 0.000275 | 0.988837 | 2.25E-09 | 0.00323 | 0.870819 |
|  | rs11242523 | 5 | 1.04E+08 | 0.002577 | 0.000258 | 0.990168 | 2.06E-09 | 0.003516 | 0.840554 |
|  | rs11738191 | 5 | 1.04E+08 | 0.001413 | 0.000448 | 0.97476 | 1.51E-09 | 0.004761 | 0.730268 |
|  | rs11738197 | 5 | 1.04E+08 | 0.001005 | 0.000614 | 0.95496 | 2.21E-09 | 0.003287 | 0.864307 |
|  | rs12187898 | 5 | 1.04E+08 | 0.001981 | 0.000329 | 0.982551 | 1.37E-09 | 0.005249 | 0.7053 |
|  | rs12187903 | 5 | 1.04E+08 | 0.001938 | 0.000335 | 0.98156 | 1.01E-09 | 0.007049 | 0.636959 |
|  | rs12515429 | 5 | 1.04E+08 | 0.002496 | 0.000266 | 0.989649 | 2.12E-09 | 0.003432 | 0.850991 |
|  | rs12658019 | 5 | 1.04E+08 | 0.002105 | 0.000311 | 0.984766 | 9.88E-10 | 0.007211 | 0.601301 |
|  | rs12658032 | 5 | 1.04E+08 | 6.16E-06 | 0.075438 | 0.686832 | 1.18E-10 | 0.057354 | 0.33975 |
|  | rs12658276 | 5 | 1.04E+08 | 0.001329 | 0.000475 | 0.9734 | 1.33E-09 | 0.00538 | 0.684063 |
|  | rs13162928 | 5 | 1.04E+08 | 0.00058 | 0.001023 | 0.945172 | 1.35E-09 | 0.005329 | 0.694748 |
|  | rs13166408 | 5 | 1.04E+08 | 0.000861 | 0.000709 | 0.950959 | 5.23E-10 | 0.013413 | 0.49002 |
|  | rs13177365 | 5 | 1.04E+08 | 0.0011 | 0.000565 | 0.962586 | 1.66E-09 | 0.004357 | 0.761674 |
|  | rs13177473 | 5 | 1.04E+08 | 0.000675 | 0.000888 | 0.947062 | 2.22E-09 | 0.003282 | 0.86759 |
|  | rs13181679 | 5 | 1.04E+08 | 0.001313 | 0.00048 | 0.97245 | 7.65E-09 | 0.000982 | 0.976111 |
|  | rs1363100 | 5 | 1.04E+08 | 0.001617 | 0.000396 | 0.980151 | 5.1E-09 | 0.001458 | 0.955114 |
|  | rs1363102 | 5 | 1.04E+08 | 0.00106 | 0.000585 | 0.959711 | 1.35E-09 | 0.005302 | 0.700051 |
|  | rs1363104 | 5 | 1.04E+08 | 0.001215 | 0.000515 | 0.967442 | 2.6E-10 | 0.026508 | 0.402295 |
|  | rs1363105 | 5 | 1.04E+08 | 0.001244 | 0.000504 | 0.970999 | 2.68E-10 | 0.025735 | 0.428031 |
|  | rs1363106 | 5 | 1.04E+08 | 0.001588 | 0.000403 | 0.979755 | 5.64E-09 | 0.001321 | 0.963428 |
|  | rs1372500 | 5 | 1.04E+08 | 0.0015 | 0.000424 | 0.976057 | 4.44E-10 | 0.015718 | 0.460933 |
|  | rs1421665 | 5 | 1.04E+08 | 0.001212 | 0.000517 | 0.96641 | 1.31E-09 | 0.005468 | 0.678684 |
|  | rs1421666 | 5 | 1.04E+08 | 0.002075 | 0.000315 | 0.984144 | 2.8E-09 | 0.002611 | 0.9031 |
|  | rs1421667 | 5 | 1.04E+08 | 0.002242 | 0.000293 | 0.98687 | 3.64E-09 | 0.002025 | 0.932683 |
|  | rs1421668 | 5 | 1.04E+08 | 0.002233 | 0.000294 | 0.986577 | 1.67E-09 | 0.004327 | 0.770338 |
|  | rs1442111 | 5 | 1.04E+08 | 0.00197 | 0.00033 | 0.982223 | 2.01E-09 | 0.003611 | 0.833492 |
|  | rs1530303 | 5 | 1.04E+08 | 0.00167 | 0.000384 | 0.980536 | 2.53E-09 | 0.00288 | 0.892212 |
|  | rs1583953 | 5 | 1.04E+08 | 0.001068 | 0.000581 | 0.960873 | 9.8E-10 | 0.007267 | 0.59409 |
|  | rs1592754 | 5 | 1.04E+08 | 0.001081 | 0.000574 | 0.961447 | 4.42E-09 | 0.001676 | 0.9472 |
|  | rs1592755 | 5 | 1.04E+08 | 0.002067 | 0.000316 | 0.983829 | 1.91E-09 | 0.003786 | 0.818665 |
|  | rs1592757 | 5 | 1.04E+08 | 5.71E-06 | 0.081015 | 0.533856 | 1.9E-10 | 0.036038 | 0.375788 |
|  | rs17156671 | 5 | 1.04E+08 | 0.002423 | 0.000273 | 0.98911 | 2.42E-09 | 0.003012 | 0.88644 |
|  | rs171697 | 5 | 1.04E+08 | 4.89E-06 | 0.093952 | 0.194465 | 1.58E-09 | 0.004576 | 0.739456 |
|  | rs191800971 | 5 | 1.04E+08 | 5.56E-06 | 0.08322 | 0.452841 | 3.01E-09 | 0.002435 | 0.910611 |
|  | rs2028526 | 5 | 1.04E+08 | 0.00091 | 0.000674 | 0.95303 | 6.48E-09 | 0.001155 | 0.971953 |
|  | rs2032790 | 5 | 1.04E+08 | 0.002101 | 0.000311 | 0.984455 | 2.81E-09 | 0.0026 | 0.905699 |
|  | rs2112163 | 5 | 1.04E+08 | 0.001326 | 0.000476 | 0.972925 | 5.72E-09 | 0.001304 | 0.964732 |
|  | rs21126 | 5 | 1.04E+08 | 0.001067 | 0.000581 | 0.960292 | 4.06E-10 | 0.017184 | 0.445215 |
|  | rs2161097 | 5 | 1.04E+08 | 0.001509 | 0.000422 | 0.976479 | 4.56E-09 | 0.001627 | 0.950491 |
|  | rs2403284 | 5 | 1.04E+08 | 0.002391 | 0.000277 | 0.988562 | 3.11E-09 | 0.002356 | 0.915335 |
|  | rs2431108 | 5 | 1.04E+08 | 4.56E-06 | 0.100513 | 0.100513 | 1.69E-09 | 0.004274 | 0.774612 |
|  | rs2431109 | 5 | 1.04E+08 | 0.001047 | 0.000591 | 0.957954 | 5.29E-09 | 0.001406 | 0.959399 |
|  | rs2447828 | 5 | 1.04E+08 | 0.00156 | 0.000409 | 0.978542 | 4.4E-09 | 0.001683 | 0.945524 |
|  | rs2447832 | 5 | 1.04E+08 | 0.001953 | 0.000333 | 0.981892 | 4.7E-09 | 0.001578 | 0.953656 |
|  | rs2447838 | 5 | 1.04E+08 | 0.002059 | 0.000317 | 0.983513 | 1.91E-09 | 0.003796 | 0.814879 |
|  | rs254011 | 5 | 1.04E+08 | 0.001082 | 0.000574 | 0.962021 | 7.99E-09 | 0.000942 | 0.97989 |
|  | rs254024 | 5 | 1.04E+08 | 0.001309 | 0.000481 | 0.97197 | 5.53E-09 | 0.001347 | 0.962107 |
|  | rs254025 | 5 | 1.04E+08 | 0.001441 | 0.00044 | 0.975201 | 4.29E-09 | 0.001724 | 0.943841 |
|  | rs254035 | 5 | 1.04E+08 | 0.002021 | 0.000323 | 0.982874 | 4.45E-09 | 0.001664 | 0.948864 |
|  | rs2896539 | 5 | 1.04E+08 | 0.002326 | 0.000284 | 0.987726 | 1.05E-08 | 0.000719 | 0.99043 |
|  | rs30266 | 5 | 1.04E+08 | 5.98E-06 | 0.077537 | 0.611393 | 2.72E-09 | 0.002688 | 0.900488 |
|  | rs325481 | 5 | 1.04E+08 | 0.000325 | 0.001759 | 0.940412 | 2.07E-09 | 0.003514 | 0.844068 |
|  | rs325485 | 5 | 1.04E+08 | 0.001008 | 0.000613 | 0.955573 | 3.46E-09 | 0.002128 | 0.928596 |
|  | rs325500 | 5 | 1.04E+08 | 0.000522 | 0.001129 | 0.944149 | 1.77E-09 | 0.004093 | 0.795225 |
|  | rs325501 | 5 | 1.04E+08 | 0.00042 | 0.001382 | 0.941794 | 3.29E-09 | 0.002234 | 0.922107 |
|  | rs325502 | 5 | 1.04E+08 | 0.001568 | 0.000407 | 0.978949 | 6.15E-10 | 0.011447 | 0.514525 |
|  | rs325506 | 5 | 1.04E+08 | 0.000308 | 0.001846 | 0.938653 | 3.97E-09 | 0.00186 | 0.938516 |
|  | rs325521 | 5 | 1.04E+08 | 0.001284 | 0.00049 | 0.971489 | 6.43E-09 | 0.001164 | 0.970799 |
|  | rs325523 | 5 | 1.04E+08 | 0.000955 | 0.000644 | 0.954346 | 4.18E-09 | 0.001771 | 0.942117 |
|  | rs35207728 | 5 | 1.04E+08 | 0.000593 | 0.001002 | 0.946174 | 3.1E-09 | 0.002367 | 0.912978 |
|  | rs35792668 | 5 | 1.04E+08 | 0.000689 | 0.000873 | 0.947935 | 2.63E-09 | 0.002779 | 0.8978 |
|  | rs35949602 | 5 | 1.04E+08 | 0.000782 | 0.000775 | 0.94871 | 7.8E-10 | 0.009084 | 0.553926 |
|  | rs396755 | 5 | 1.04E+08 | 0.000783 | 0.000775 | 0.949485 | 8.01E-10 | 0.008843 | 0.562769 |
|  | rs410915 | 5 | 1.04E+08 | 0.001229 | 0.00051 | 0.968979 | 1.15E-09 | 0.006201 | 0.650129 |
|  | rs416223 | 5 | 1.04E+08 | 0.000478 | 0.001226 | 0.94302 | 1.24E-09 | 0.005786 | 0.661924 |
|  | rs4235642 | 5 | 1.04E+08 | 2.48E-05 | 0.019987 | 0.936807 | 5.47E-09 | 0.001361 | 0.96076 |
|  | rs4295362 | 5 | 1.04E+08 | 0.00186 | 0.000348 | 0.980884 | 3.28E-09 | 0.00224 | 0.919873 |
|  | rs4320234 | 5 | 1.04E+08 | 0.002558 | 0.00026 | 0.989909 | 2.17E-09 | 0.003356 | 0.857727 |
|  | rs445578 | 5 | 1.04E+08 | 0.002132 | 0.000307 | 0.985073 | 2.29E-09 | 0.003179 | 0.873998 |
|  | rs4510551 | 5 | 1.04E+08 | 0.000794 | 0.000764 | 0.95025 | 1.75E-09 | 0.004131 | 0.782908 |
|  | rs4521446 | 5 | 1.04E+08 | 0.0023 | 0.000287 | 0.987157 | 1.92E-09 | 0.003774 | 0.826217 |
|  | rs62362442 | 5 | 1.04E+08 | 0.001408 | 0.00045 | 0.974312 | 8.86E-10 | 0.008021 | 0.579279 |
|  | rs62362443 | 5 | 1.04E+08 | 0.001537 | 0.000415 | 0.976894 | 2.52E-09 | 0.002892 | 0.889332 |
|  | rs62362459 | 5 | 1.04E+08 | 0.00124 | 0.000506 | 0.969485 | 1.74E-09 | 0.004164 | 0.778776 |
|  | rs6596578 | 5 | 1.04E+08 | 0.002332 | 0.000283 | 0.988009 | 1.59E-09 | 0.004545 | 0.744001 |
|  | rs6865511 | 5 | 1.04E+08 | 0.002312 | 0.000285 | 0.987442 | 2.08E-09 | 0.003491 | 0.847559 |
|  | rs6867409 | 5 | 1.04E+08 | 0.002136 | 0.000307 | 0.98538 | 1.66E-09 | 0.004337 | 0.766011 |
|  | rs6869862 | 5 | 1.04E+08 | 0.002154 | 0.000304 | 0.985684 | 5.18E-09 | 0.001435 | 0.957993 |
|  | rs6874138 | 5 | 1.04E+08 | 0.002188 | 0.0003 | 0.985984 | 1.64E-09 | 0.004404 | 0.757317 |
|  | rs72776989 | 5 | 1.04E+08 | 0.001059 | 0.000585 | 0.959126 | 1E-09 | 0.007126 | 0.622792 |
|  | rs76025409 | 5 | 1.04E+08 | 5.14E-06 | 0.089575 | 0.28404 | 2.32E-11 | 0.282396 | 0.282396 |
|  | rs768792 | 5 | 1.04E+08 | 0.000879 | 0.000696 | 0.952356 | 2.05E-09 | 0.003546 | 0.837038 |
|  | rs768905 | 5 | 1.04E+08 | 0.001907 | 0.00034 | 0.981224 | 2.34E-09 | 0.003116 | 0.883428 |
|  | rs7703746 | 5 | 1.04E+08 | 0.001123 | 0.000554 | 0.964823 | 1.37E-09 | 0.005227 | 0.710527 |
|  | rs7706353 | 5 | 1.04E+08 | 0.002388 | 0.000277 | 0.988285 | 1.61E-09 | 0.004471 | 0.748471 |
|  | rs7710489 | 5 | 1.04E+08 | 0.001546 | 0.000413 | 0.97772 | 1.82E-09 | 0.003976 | 0.803278 |
|  | rs7723509 | 5 | 1.04E+08 | 0.001585 | 0.000403 | 0.979353 | 1.98E-09 | 0.003663 | 0.82988 |
|  | rs77960 | 5 | 1.04E+08 | 5.4E-06 | 0.085581 | 0.369621 | 2.6E-09 | 0.002809 | 0.895021 |
|  | rs876392 | 5 | 1.04E+08 | 0.001543 | 0.000413 | 0.977307 | 4.04E-09 | 0.00183 | 0.940347 |
|  | rs920623 | 5 | 1.04E+08 | 0.001244 | 0.000504 | 0.970495 | 1.75E-09 | 0.004122 | 0.78703 |
|  | rs959857 | 5 | 1.04E+08 | 0.002027 | 0.000322 | 0.983196 | 3.33E-09 | 0.002209 | 0.924317 |

Abbreviations: probNorm refers to the normalized posterior p-value for each SNP. cumSum means the cumulative normalized posterior p-value for SNP sets. For each sentinel SNP, a 99% credible set of potentially causal SNPs was obtained using the FM-summary method. Each SNP has a corresponding posterior probability, i.e., probNorm, and with each superimposition of another SNP, there is a subsequent cumSum, which is obtained by the the corresponding probNorm of each SNP is summed up. Ultimately, this can be interpreted to mean that there is a 99% probability that the causal SNPs that actually have an effect on the two traits are contained in such a credible set.

## Supplementary Table 11. Fine-mapping 99% credible-set of sentinel SNP from cross-trait meta-analysis between educational attainment (EA) and posttraumatic stress disorder (PTSD).

| Sentinel.SNP | Credible-set SNPs | CHR | BP | EA | | | PTSD | | |
| --- | --- | --- | --- | --- | --- | --- | --- | --- | --- |
|  |  |  |  | **GWAS-p-value** | **probNorm** | **cumSum** | **GWAS-p-value** | **probNorm** | **cumSum** |
| rs71351952 | rs10485609 | 20 | 47665612 | 1.55E-05 | 0.003813 | 0.765475 | 8.92E-06 | 0.016083 | 0.312736 |
|  | rs1048590 | 20 | 47610297 | 3.58E-05 | 0.001714 | 0.885207 | 0.000775 | 0.000238 | 0.988683 |
|  | rs11906283 | 20 | 47684868 | 1.22E-05 | 0.004788 | 0.70464 | 1.21E-05 | 0.01206 | 0.842488 |
|  | rs11906450 | 20 | 47555082 | 1.44E-05 | 0.004083 | 0.753745 | 8.1E-06 | 0.017642 | 0.180424 |
|  | rs11906713 | 20 | 47678100 | 9.21E-06 | 0.006246 | 0.610318 | 1.12E-05 | 0.012986 | 0.818025 |
|  | rs11908024 | 20 | 47698186 | 1.1E-05 | 0.005296 | 0.643909 | 1.06E-05 | 0.013665 | 0.604853 |
|  | rs13036715 | 20 | 47817815 | 1.08E-06 | 0.048982 | 0.211196 | 2.74E-05 | 0.005522 | 0.966458 |
|  | rs13037063 | 20 | 47525665 | 2.16E-05 | 0.002776 | 0.801759 | 4.16E-06 | 0.033399 | 0.112085 |
|  | rs13037557 | 20 | 47534590 | 5.87E-05 | 0.001073 | 0.981175 | 0.00048 | 0.000371 | 0.97626 |
|  | rs13037942 | 20 | 47525671 | 1.58E-05 | 0.003735 | 0.76921 | 3.94E-06 | 0.035151 | 0.078686 |
|  | rs13039749 | 20 | 47800615 | 3.18E-06 | 0.017272 | 0.340371 | 1.44E-05 | 0.010163 | 0.928692 |
|  | rs13040292 | 20 | 47721582 | 1.08E-05 | 0.005347 | 0.633294 | 1.06E-05 | 0.013665 | 0.618518 |
|  | rs13040426 | 20 | 47714453 | 1.17E-05 | 0.004988 | 0.680332 | 1.1E-05 | 0.01319 | 0.752541 |
|  | rs13041022 | 20 | 47642476 | 3.33E-05 | 0.001835 | 0.853496 | 0.000711 | 0.000257 | 0.980954 |
|  | rs13041213 | 20 | 47800829 | 3.54E-06 | 0.015586 | 0.355957 | 2.56E-05 | 0.005896 | 0.960936 |
|  | rs13042443 | 20 | 47663754 | 1.53E-05 | 0.003861 | 0.761662 | 9.16E-06 | 0.01568 | 0.328417 |
|  | rs13042947 | 20 | 47647876 | 3.57E-05 | 0.001721 | 0.883493 | 0.00071 | 0.000258 | 0.980182 |
|  | rs13044004 | 20 | 47718371 | 1.13E-05 | 0.005139 | 0.675345 | 1.05E-05 | 0.01374 | 0.591188 |
|  | rs13044144 | 20 | 47528808 | 9.37E-06 | 0.006149 | 0.616467 | 9.29E-06 | 0.015474 | 0.359369 |
|  | rs1556876 | 20 | 47716451 | 1.11E-05 | 0.005232 | 0.670206 | 1.07E-05 | 0.013471 | 0.672728 |
|  | rs1569749 | 20 | 47601509 | 4.13E-05 | 0.001498 | 0.919082 | 0.000772 | 0.000239 | 0.988445 |
|  | rs1569750 | 20 | 47603241 | 3.81E-05 | 0.001617 | 0.905153 | 0.00076 | 0.000242 | 0.987727 |
|  | rs17378391 | 20 | 47772601 | 6.14E-06 | 0.009212 | 0.4749 | 1.1E-05 | 0.01319 | 0.765731 |
|  | rs17448715 | 20 | 47691743 | 1.22E-05 | 0.004769 | 0.70941 | 1.05E-05 | 0.013802 | 0.563671 |
|  | rs17450430 | 20 | 47772264 | 5.08E-06 | 0.011036 | 0.426919 | 1.25E-05 | 0.011645 | 0.854134 |
|  | rs1983528 | 20 | 47686290 | 2.71E-05 | 0.002237 | 0.824286 | 1.1E-05 | 0.013213 | 0.739352 |
|  | rs1983639 | 20 | 47572282 | 3.61E-05 | 0.001701 | 0.888616 | 0.00077 | 0.000239 | 0.988207 |
|  | rs1997854 | 20 | 47602339 | 4.18E-05 | 0.001479 | 0.926522 | 0.000757 | 0.000243 | 0.987 |
|  | rs2075677 | 20 | 47701024 | 1.11E-05 | 0.005237 | 0.664973 | 1.03E-05 | 0.014046 | 0.521932 |
|  | rs2227946 | 20 | 47685320 | 1.25E-05 | 0.004678 | 0.718795 | 1.39E-05 | 0.010517 | 0.897924 |
|  | rs2273101 | 20 | 47538333 | 4.93E-06 | 0.011368 | 0.404646 | 6.31E-06 | 0.02241 | 0.162782 |
|  | rs2295026 | 20 | 47582785 | 8.41E-06 | 0.006817 | 0.584377 | 9.93E-06 | 0.014513 | 0.479314 |
|  | rs2295027 | 20 | 47582884 | 5.69E-05 | 0.001106 | 0.974665 | 0.000652 | 0.000279 | 0.977805 |
|  | rs2295033 | 20 | 47610896 | 3.46E-05 | 0.001771 | 0.86784 | 0.000757 | 0.000243 | 0.987243 |
|  | rs2869692 | 20 | 47638256 | 0.00005 | 0.001249 | 0.95246 | 0.00061 | 0.000297 | 0.977246 |
|  | rs2869694 | 20 | 47668383 | 5.84E-05 | 0.001078 | 0.980102 | 0.000732 | 0.000251 | 0.985027 |
|  | rs34417222 | 20 | 47450063 | 1.97E-05 | 0.003019 | 0.793223 | 0.000125 | 0.001308 | 0.967766 |
|  | rs34651656 | 20 | 47534729 | 5.32E-05 | 0.001178 | 0.963307 | 0.00045 | 0.000394 | 0.975888 |
|  | rs34806496 | 20 | 47812203 | 2.98E-06 | 0.018416 | 0.305754 | 1.45E-05 | 0.010102 | 0.938794 |
|  | rs34832487 | 20 | 47547112 | 7.86E-06 | 0.007273 | 0.556235 | 9.76E-06 | 0.01476 | 0.464801 |
|  | rs34841991 | 20 | 47746974 | 4.9E-06 | 0.011428 | 0.393278 | 1.25E-05 | 0.011628 | 0.865761 |
|  | rs34984028 | 20 | 47549047 | 5.55E-05 | 0.001131 | 0.970229 | 0.000776 | 0.000237 | 0.98892 |
|  | rs35334143 | 20 | 47530708 | 1.71E-05 | 0.003466 | 0.786953 | 3.15E-06 | 0.043535 | 0.043535 |
|  | rs35393280 | 20 | 47543634 | 8.42E-06 | 0.006805 | 0.591182 | 8.86E-06 | 0.016191 | 0.280528 |
|  | rs35640549 | 20 | 47656546 | 1.43E-05 | 0.004116 | 0.749661 | 9.65E-06 | 0.014928 | 0.435125 |
|  | rs35649484 | 20 | 47755162 | 6.16E-06 | 0.009185 | 0.484086 | 8.77E-06 | 0.016348 | 0.248067 |
|  | rs35667796 | 20 | 47803301 | 2.59E-06 | 0.021087 | 0.287338 | 1.46E-05 | 0.010043 | 0.948837 |
|  | rs35752040 | 20 | 47624600 | 7.83E-06 | 0.007301 | 0.548961 | 9.58E-06 | 0.015025 | 0.405231 |
|  | rs35843274 | 20 | 47665903 | 1.83E-05 | 0.00325 | 0.790203 | 8.9E-06 | 0.016125 | 0.296653 |
|  | rs35888516 | 20 | 47764275 | 5.99E-06 | 0.009421 | 0.456309 | 8.12E-06 | 0.017588 | 0.198013 |
|  | rs35971376 | 20 | 47721708 | 1.22E-05 | 0.004796 | 0.699852 | 1.02E-05 | 0.014164 | 0.507886 |
|  | rs35989889 | 20 | 47717353 | 1.17E-05 | 0.004988 | 0.68532 | 1.08E-05 | 0.013352 | 0.712904 |
|  | rs36098998 | 20 | 47688109 | 1.29E-05 | 0.004526 | 0.732492 | 1.08E-05 | 0.013364 | 0.699551 |
|  | rs3795075 | 20 | 47806092 | 2.49E-06 | 0.02185 | 0.266251 | 1.32E-05 | 0.011048 | 0.876809 |
|  | rs3795082 | 20 | 47673002 | 5.45E-05 | 0.001151 | 0.966804 | 0.000699 | 0.000261 | 0.978889 |
|  | rs3817652 | 20 | 47663368 | 5.66E-05 | 0.00111 | 0.97245 | 0.000734 | 0.00025 | 0.985528 |
|  | rs45622034 | 20 | 47775094 | 6.02E-06 | 0.009379 | 0.465688 | 1.17E-05 | 0.012404 | 0.830428 |
|  | rs4599176 | 20 | 47520094 | 1.1E-05 | 0.005268 | 0.654468 | 1.09E-05 | 0.013236 | 0.726139 |
|  | rs6012586 | 20 | 47647417 | 5.48E-05 | 0.001145 | 0.969097 | 0.00071 | 0.000258 | 0.98044 |
|  | rs6012590 | 20 | 47667237 | 5.67E-05 | 0.001109 | 0.97356 | 0.000731 | 0.000251 | 0.984777 |
|  | rs6012591 | 20 | 47667342 | 5.73E-05 | 0.001097 | 0.975762 | 0.000721 | 0.000254 | 0.983514 |
|  | rs6012592 | 20 | 47677162 | 4.33E-05 | 0.00143 | 0.935244 | 0.000782 | 0.000236 | 0.989156 |
|  | rs6019547 | 20 | 47561091 | 4.78E-05 | 0.001304 | 0.947399 | 0.000653 | 0.000279 | 0.978084 |
|  | rs6019557 | 20 | 47583372 | 6.71E-05 | 0.000945 | 0.98825 | 0.000751 | 0.000245 | 0.986514 |
|  | rs6019558 | 20 | 47583480 | 3.59E-05 | 0.001708 | 0.886915 | 0.000746 | 0.000246 | 0.986024 |
|  | rs6019567 | 20 | 47595961 | 3.3E-05 | 0.001853 | 0.847972 | 0.000515 | 0.000348 | 0.976608 |
|  | rs6019573 | 20 | 47602832 | 3.33E-05 | 0.001839 | 0.851661 | 0.000756 | 0.000243 | 0.986757 |
|  | rs6019591 | 20 | 47640029 | 3.79E-05 | 0.001625 | 0.903536 | 0.000711 | 0.000257 | 0.980697 |
|  | rs6019594 | 20 | 47643554 | 3.79E-05 | 0.001625 | 0.901911 | 0.000704 | 0.00026 | 0.979409 |
|  | rs6019595 | 20 | 47644032 | 3.65E-05 | 0.001683 | 0.89537 | 0.000712 | 0.000257 | 0.981469 |
|  | rs6019600 | 20 | 47646932 | 3.52E-05 | 0.00174 | 0.874865 | 0.000786 | 0.000234 | 0.989861 |
|  | rs6019601 | 20 | 47647662 | 3.75E-05 | 0.001639 | 0.898658 | 0.000674 | 0.00027 | 0.978627 |
|  | rs6019602 | 20 | 47651492 | 3.49E-05 | 0.001758 | 0.871369 | 0.000703 | 0.00026 | 0.979149 |
|  | rs6019603 | 20 | 47653406 | 5.32E-05 | 0.001177 | 0.964484 | 0.000715 | 0.000256 | 0.982239 |
|  | rs6019608 | 20 | 47655617 | 5.31E-05 | 0.001179 | 0.962129 | 0.000723 | 0.000253 | 0.983767 |
|  | rs6019609 | 20 | 47655768 | 4.88E-05 | 0.001278 | 0.948678 | 0.000717 | 0.000255 | 0.983005 |
|  | rs6019618 | 20 | 47671538 | 5.46E-05 | 0.001148 | 0.967952 | 0.00075 | 0.000245 | 0.986269 |
|  | rs6019620 | 20 | 47679300 | 3.4E-05 | 0.001799 | 0.857115 | 0.000765 | 0.00024 | 0.987968 |
|  | rs6019621 | 20 | 47679963 | 3.55E-05 | 0.001729 | 0.878325 | 0.000797 | 0.000232 | 0.990093 |
|  | rs6090932 | 20 | 47598298 | 3.42E-05 | 0.001792 | 0.862494 | 0.000759 | 0.000242 | 0.987485 |
|  | rs6090939 | 20 | 47665825 | 6.72E-05 | 0.000943 | 0.989194 | 0.000724 | 0.000253 | 0.984274 |
|  | rs6095360 | 20 | 47532536 | 5.31E-05 | 0.00118 | 0.96095 | 0.000415 | 0.000425 | 0.974695 |
|  | rs6095367 | 20 | 47550488 | 4.76E-05 | 0.001308 | 0.944789 | 0.000667 | 0.000273 | 0.978357 |
|  | rs6095391 | 20 | 47618835 | 8.03E-06 | 0.007124 | 0.570557 | 8.7E-06 | 0.016475 | 0.231719 |
|  | rs6095400 | 20 | 47645989 | 3.56E-05 | 0.001721 | 0.881772 | 0.00071 | 0.000258 | 0.979667 |
|  | rs6095401 | 20 | 47646621 | 1.33E-05 | 0.004409 | 0.7369 | 0.00071 | 0.000258 | 0.979924 |
|  | rs6095407 | 20 | 47661470 | 5.65E-05 | 0.001112 | 0.97134 | 0.000717 | 0.000255 | 0.982494 |
|  | rs6095417 | 20 | 47678201 | 1.1E-05 | 0.005291 | 0.6492 | 0.000784 | 0.000235 | 0.989627 |
|  | rs6512554 | 20 | 47532750 | 6.02E-05 | 0.001047 | 0.98327 | 0.000444 | 0.000399 | 0.975095 |
|  | rs6512562 | 20 | 47622729 | 7.79E-06 | 0.00733 | 0.541661 | 9.65E-06 | 0.014916 | 0.450041 |
|  | rs66501340 | 20 | 47677185 | 4.88E-05 | 0.001278 | 0.949956 | 0.000782 | 0.000236 | 0.989392 |
|  | rs66970580 | 20 | 47641339 | 3.9E-05 | 0.001582 | 0.909958 | 0.000717 | 0.000255 | 0.982749 |
|  | rs67623477 | 20 | 47669177 | 1.69E-05 | 0.003505 | 0.783488 | 9.29E-06 | 0.015479 | 0.343895 |
|  | rs67777906 | 20 | 47557129 | 7.04E-06 | 0.008081 | 0.526822 | 9.31E-06 | 0.015436 | 0.374805 |
|  | rs707533 | 20 | 47652254 | 3.56E-05 | 0.001726 | 0.880051 | 0.000712 | 0.000257 | 0.981726 |
|  | rs707534 | 20 | 47652338 | 4.17E-05 | 0.001483 | 0.925043 | 0.000727 | 0.000252 | 0.984526 |
|  | rs71351952 | 20 | 47523789 | 9.6E-06 | 0.006006 | 0.622474 | 4.94E-06 | 0.028288 | 0.140373 |
|  | rs71351953 | 20 | 47574747 | 8.91E-06 | 0.006452 | 0.597634 | 1.06E-05 | 0.013616 | 0.632134 |
|  | rs7262349 | 20 | 47795305 | 6.88E-06 | 0.008258 | 0.518742 | 1.43E-05 | 0.010237 | 0.918529 |
|  | rs7262379 | 20 | 47642579 | 3.63E-05 | 0.00169 | 0.892002 | 0.000712 | 0.000257 | 0.981212 |
|  | rs7264419 | 20 | 47701309 | 1.34E-05 | 0.00438 | 0.741281 | 1.06E-05 | 0.013616 | 0.645751 |
|  | rs7266065 | 20 | 47531817 | 5.81E-05 | 0.001083 | 0.977941 | 0.000444 | 0.000399 | 0.975494 |
|  | rs7269912 | 20 | 47656198 | 1.45E-05 | 0.004056 | 0.757801 | 9.34E-06 | 0.015401 | 0.390206 |
|  | rs7270848 | 20 | 47639737 | 3.54E-05 | 0.001732 | 0.876596 | 0.000719 | 0.000255 | 0.98326 |
|  | rs7271812 | 20 | 47693033 | 1.29E-05 | 0.004532 | 0.727966 | 1.04E-05 | 0.013942 | 0.549868 |
|  | rs7274221 | 20 | 47623498 | 8.17E-06 | 0.007003 | 0.57756 | 9.62E-06 | 0.014966 | 0.420197 |
|  | rs7274612 | 20 | 47702821 | 1.19E-05 | 0.004899 | 0.69022 | 1.1E-05 | 0.013178 | 0.778909 |
|  | rs7274851 | 20 | 47794355 | 4.99E-06 | 0.011237 | 0.415883 | 1.38E-05 | 0.010597 | 0.887407 |
|  | rs73611323 | 20 | 47647437 | 1.09E-05 | 0.005319 | 0.638613 | 1.11E-05 | 0.013053 | 0.805039 |
|  | rs73611328 | 20 | 47669980 | 5.36E-05 | 0.00117 | 0.965653 | 0.000734 | 0.00025 | 0.985778 |
|  | rs73611337 | 20 | 47717193 | 6.3E-06 | 0.008981 | 0.493067 | 1.07E-05 | 0.013507 | 0.659258 |
|  | rs73611338 | 20 | 47717235 | 1.1E-05 | 0.005268 | 0.659737 | 1.11E-05 | 0.013076 | 0.791985 |
|  | rs755588 | 20 | 47721028 | 1.2E-05 | 0.004837 | 0.695057 | 1E-05 | 0.014408 | 0.493722 |
|  | rs79941596 | 20 | 47793796 | 4.44E-06 | 0.012563 | 0.381849 | 1.41E-05 | 0.010368 | 0.908292 |
|  | rs8116693 | 20 | 47762210 | 5.34E-06 | 0.010525 | 0.437444 | 8.81E-06 | 0.01627 | 0.264337 |
|  | rs8118101 | 20 | 47689838 | 1.26E-05 | 0.004639 | 0.723434 | 1.05E-05 | 0.013777 | 0.577448 |
|  | rs8121567 | 20 | 47816873 | 3.17E-06 | 0.017345 | 0.323099 | 2.42E-05 | 0.006203 | 0.95504 |
|  | rs8122757 | 20 | 47762249 | 6.47E-06 | 0.008759 | 0.501826 | 8.3E-06 | 0.017231 | 0.215244 |
|  | rs8123784 | 20 | 47710434 | 1.37E-05 | 0.004265 | 0.745545 | 1.03E-05 | 0.013994 | 0.535926 |
|  | rs8125219 | 20 | 47598661 | 5.98E-06 | 0.009444 | 0.446888 | 1.08E-05 | 0.013459 | 0.686187 |
|  | rs927160 | 20 | 47654564 | 3.08E-05 | 0.001977 | 0.836628 | 0.000714 | 0.000257 | 0.981982 |

Abbreviations: probNorm refers to the normalized posterior p-value for each SNP. cumSum means the cumulative normalized posterior p-value for SNP sets. For each sentinel SNP, a 99% credible set of potentially causal SNPs was obtained using the FM-summary method. Each SNP has a corresponding posterior probability, i.e., probNorm, and with each superimposition of another SNP, there is a subsequent cumSum, which is obtained by the the corresponding probNorm of each SNP is summed up. Ultimately, this can be interpreted to mean that there is a 99% probability that the causal SNPs that actually have an effect on the two traits are contained in such a credible set.

## Supplementary Table 12. Fine-mapping 99% credible-set of sentinel SNP from cross-trait meta-analysis between educational attainment (EA) and Tourette’s syndrome (TS).

| Sentinel.SNP | Credible-set SNPs | CHR | BP | EA | | | TS | | |
| --- | --- | --- | --- | --- | --- | --- | --- | --- | --- |
|  |  |  |  | **GWAS-p-value** | **probNorm** | **cumSum** | **GWAS-p-value** | **probNorm** | **cumSum** |
| rs12154193 | rs10872224 | 6 | 98435125 | 4.92E-21 | 0.047219 | 0.533786 | 1.43E-06 | 0.063042 | 0.130405 |
|  | rs11153969 | 6 | 98358220 | 6.1E-20 | 0.003917 | 0.993424 | 2.48E-05 | 0.004097 | 0.856993 |
|  | rs12176311 | 6 | 98529166 | 2.7E-20 | 0.00878 | 0.942374 | 1.34E-05 | 0.007381 | 0.530145 |
|  | rs13215227 | 6 | 98506764 | 1.47E-20 | 0.016 | 0.749416 | 1.49E-05 | 0.00667 | 0.621336 |
|  | rs13216075 | 6 | 98522436 | 3.29E-20 | 0.007221 | 0.974571 | 1.37E-05 | 0.007222 | 0.551962 |
|  | rs28792186 | 6 | 98508087 | 6.41E-22 | 0.354963 | 0.354963 | 1.65E-05 | 0.006058 | 0.685861 |
|  | rs3004179 | 6 | 98513032 | 2.42E-20 | 0.009778 | 0.933593 | 3.24E-05 | 0.003185 | 0.874917 |
|  | rs35062457 | 6 | 98522698 | 2.3E-20 | 0.010264 | 0.903718 | 1.57E-05 | 0.00633 | 0.679803 |
|  | rs35121390 | 6 | 98399208 | 5.21E-21 | 0.044636 | 0.578423 | 2.06E-06 | 0.044331 | 0.327507 |
|  | rs4339469 | 6 | 98369230 | 1.75E-20 | 0.013466 | 0.836785 | 2.17E-05 | 0.004656 | 0.848729 |
|  | rs4401671 | 6 | 98519911 | 2.08E-20 | 0.011368 | 0.871459 | 1.54E-05 | 0.006475 | 0.667105 |
|  | rs4458695 | 6 | 98528404 | 2.05E-20 | 0.011499 | 0.860091 | 1.36E-05 | 0.007262 | 0.544741 |
|  | rs4548017 | 6 | 98399521 | 5.83E-21 | 0.039951 | 0.662244 | 2.05E-06 | 0.044414 | 0.283176 |
|  | rs4576255 | 6 | 98509656 | 1.65E-20 | 0.014316 | 0.823319 | 1.53E-05 | 0.006483 | 0.654151 |
|  | rs4839715 | 6 | 98365289 | 5.87E-20 | 0.004068 | 0.985499 | 1.96E-05 | 0.005135 | 0.789172 |
|  | rs4839938 | 6 | 98507430 | 1.64E-20 | 0.01435 | 0.809003 | 1.49E-05 | 0.006661 | 0.627998 |
|  | rs55731746 | 6 | 98514302 | 2.98E-20 | 0.007966 | 0.96735 | 1.48E-05 | 0.006726 | 0.614667 |
|  | rs62422661 | 6 | 98545611 | 3.46E-20 | 0.00686 | 0.981431 | 7.83E-06 | 0.012307 | 0.40271 |
|  | rs6905544 | 6 | 98411631 | 5.3E-21 | 0.04387 | 0.622293 | 1.94E-06 | 0.04685 | 0.238762 |
|  | rs6937215 | 6 | 98373765 | 1.34E-20 | 0.017482 | 0.716531 | 1.35E-05 | 0.007334 | 0.537479 |
|  | rs6938973 | 6 | 98421721 | 3.89E-21 | 0.059551 | 0.486567 | 1.46E-06 | 0.061508 | 0.191912 |
|  | rs76112426 | 6 | 98532427 | 5.96E-20 | 0.004008 | 0.989507 | 1.28E-05 | 0.007694 | 0.522764 |
|  | rs80147556 | 6 | 98532484 | 2.83E-20 | 0.008372 | 0.959384 | 1.28E-05 | 0.007694 | 0.515069 |
|  | rs901628 | 6 | 98539377 | 2.36E-20 | 0.010023 | 0.923816 | 1.18E-05 | 0.008301 | 0.483075 |
|  | rs901629 | 6 | 98539379 | 2E-20 | 0.011806 | 0.848591 | 1.18E-05 | 0.008301 | 0.474774 |
|  | rs901630 | 6 | 98539519 | 1.58E-20 | 0.01487 | 0.78019 | 1.18E-05 | 0.008295 | 0.49137 |
|  | rs9320823 | 6 | 98429337 | 6.33E-21 | 0.036804 | 0.699049 | 1.33E-06 | 0.067363 | 0.067363 |
|  | rs9372625 | 6 | 98344031 | 1.39E-20 | 0.016886 | 0.733417 | 8.23E-05 | 0.001315 | 0.927678 |
|  | rs9388083 | 6 | 98525726 | 2.74E-20 | 0.008638 | 0.951012 | 1.56E-05 | 0.006368 | 0.673473 |
|  | rs9398676 | 6 | 98526943 | 2.19E-20 | 0.010793 | 0.893454 | 1.53E-05 | 0.006479 | 0.66063 |
|  | rs9401452 | 6 | 98452680 | 1.63E-20 | 0.014463 | 0.794653 | 5.57E-06 | 0.017069 | 0.376132 |
|  | rs9401458 | 6 | 98458340 | 2.35E-20 | 0.010074 | 0.913792 | 6.71E-06 | 0.014271 | 0.390403 |
|  | rs9401501 | 6 | 98500712 | 1.48E-20 | 0.015903 | 0.76532 | 1.45E-05 | 0.006859 | 0.594344 |
|  | rs9482094 | 6 | 98364895 | 2.11E-20 | 0.011203 | 0.882661 | 1.76E-05 | 0.005696 | 0.709493 |
|  | rs9482120 | 6 | 98392667 | 3.21E-21 | 0.072053 | 0.427016 | 2.93E-06 | 0.031556 | 0.359062 |
| rs1619561 | rs1047158 | 12 | 1.24E+08 | 5.6E-14 | 0.000518 | 0.976864 | 2.4E-05 | 0.012697 | 0.357296 |
|  | rs1051431 | 12 | 1.24E+08 | 7.74E-14 | 0.000377 | 0.983657 | 2.24E-05 | 0.013554 | 0.318379 |
|  | rs1060105 | 12 | 1.24E+08 | 2.74E-15 | 0.010082 | 0.723778 | 0.001244 | 0.000311 | 0.983357 |
|  | rs10732573 | 12 | 1.24E+08 | 1.42E-15 | 0.019234 | 0.526481 | 0.000269 | 0.001292 | 0.934186 |
|  | rs10734900 | 12 | 1.24E+08 | 1.63E-15 | 0.016806 | 0.594902 | 0.000265 | 0.00131 | 0.931584 |
|  | rs10734901 | 12 | 1.24E+08 | 1.59E-15 | 0.017221 | 0.561096 | 0.000265 | 0.00131 | 0.932894 |
|  | rs10744147 | 12 | 1.24E+08 | 4.23E-14 | 0.000682 | 0.969 | 0.000209 | 0.001639 | 0.913169 |
|  | rs10744149 | 12 | 1.24E+08 | 9.64E-15 | 0.002923 | 0.881289 | 3.19E-05 | 0.009694 | 0.506769 |
|  | rs10744150 | 12 | 1.24E+08 | 2.1E-15 | 0.013123 | 0.667842 | 0.000261 | 0.00133 | 0.930274 |
|  | rs10744151 | 12 | 1.24E+08 | 3.57E-15 | 0.007785 | 0.809214 | 0.000312 | 0.001125 | 0.943832 |
|  | rs10772993 | 12 | 1.24E+08 | 1.41E-14 | 0.002006 | 0.913787 | 0.000186 | 0.001824 | 0.904661 |
|  | rs10772997 | 12 | 1.24E+08 | 2.73E-15 | 0.010122 | 0.713696 | 0.000259 | 0.001341 | 0.928944 |
|  | rs10772998 | 12 | 1.24E+08 | 4.12E-14 | 0.000701 | 0.96763 | 8.11E-05 | 0.003998 | 0.786864 |
|  | rs10772999 | 12 | 1.24E+08 | 3.46E-15 | 0.008011 | 0.785519 | 0.00035 | 0.00101 | 0.948031 |
|  | rs10773001 | 12 | 1.24E+08 | 2.33E-14 | 0.001227 | 0.943881 | 0.000135 | 0.002472 | 0.875102 |
|  | rs10773002 | 12 | 1.24E+08 | 1.04E-15 | 0.026096 | 0.467809 | 0.000316 | 0.001113 | 0.944945 |
|  | rs10773008 | 12 | 1.24E+08 | 4.94E-16 | 0.054546 | 0.186018 | 0.001129 | 0.00034 | 0.981402 |
|  | rs10846478 | 12 | 1.24E+08 | 9.74E-14 | 0.000301 | 0.989359 | 5.1E-05 | 0.006204 | 0.594738 |
|  | rs10846491 | 12 | 1.24E+08 | 9.61E-15 | 0.002934 | 0.878366 | 0.000257 | 0.001351 | 0.927604 |
|  | rs10846515 | 12 | 1.24E+08 | 5.24E-16 | 0.051477 | 0.237495 | 0.001152 | 0.000334 | 0.982074 |
|  | rs11057189 | 12 | 1.24E+08 | 7.4E-15 | 0.003794 | 0.855903 | 0.000426 | 0.00084 | 0.959057 |
|  | rs11057192 | 12 | 1.24E+08 | 6.01E-14 | 0.000483 | 0.97931 | 6.72E-05 | 0.004777 | 0.68697 |
|  | rs11057202 | 12 | 1.24E+08 | 2.46E-15 | 0.011237 | 0.703575 | 0.000252 | 0.001373 | 0.923535 |
|  | rs11057204 | 12 | 1.24E+08 | 6.28E-14 | 0.000463 | 0.980718 | 0.000106 | 0.003099 | 0.84841 |
|  | rs11057205 | 12 | 1.24E+08 | 3.49E-15 | 0.007952 | 0.801429 | 0.000312 | 0.001126 | 0.942707 |
|  | rs11057206 | 12 | 1.24E+08 | 3.59E-14 | 0.000803 | 0.963256 | 9.51E-05 | 0.00344 | 0.832109 |
|  | rs11057207 | 12 | 1.24E+08 | 1.82E-15 | 0.015094 | 0.640943 | 0.000273 | 0.001276 | 0.936747 |
|  | rs11057209 | 12 | 1.24E+08 | 2.12E-15 | 0.013007 | 0.680848 | 0.000252 | 0.001377 | 0.922162 |
|  | rs11057210 | 12 | 1.24E+08 | 3.53E-14 | 0.000816 | 0.962453 | 9.46E-05 | 0.003457 | 0.82867 |
|  | rs11057270 | 12 | 1.24E+08 | 6.84E-16 | 0.039594 | 0.323608 | 0.001338 | 0.000291 | 0.985457 |
|  | rs1106240 | 12 | 1.24E+08 | 1.25E-14 | 0.002263 | 0.907605 | 0.000177 | 0.001919 | 0.899105 |
|  | rs1106241 | 12 | 1.24E+08 | 4.62E-14 | 0.000626 | 0.970258 | 1.91E-05 | 0.015808 | 0.160905 |
|  | rs11532322 | 12 | 1.24E+08 | 1.54E-14 | 0.001844 | 0.919465 | 0.000216 | 0.001586 | 0.914756 |
|  | rs11830103 | 12 | 1.24E+08 | 2.4E-15 | 0.01149 | 0.692338 | 0.00118 | 0.000326 | 0.982729 |
|  | rs12298826 | 12 | 1.24E+08 | 7.58E-15 | 0.003704 | 0.859607 | 0.001 | 0.00038 | 0.977074 |
|  | rs12304248 | 12 | 1.24E+08 | 8.64E-16 | 0.031451 | 0.355059 | 0.001347 | 0.000289 | 0.985746 |
|  | rs12316131 | 12 | 1.24E+08 | 2E-15 | 0.013776 | 0.654719 | 0.001217 | 0.000317 | 0.983046 |
|  | rs12322888 | 12 | 1.24E+08 | 5.86E-15 | 0.004777 | 0.84799 | 0.001323 | 0.000294 | 0.985167 |
|  | rs1260294 | 12 | 1.24E+08 | 1.96E-14 | 0.001456 | 0.939912 | 0.001286 | 0.000301 | 0.984576 |
|  | rs12817892 | 12 | 1.24E+08 | 9.33E-15 | 0.003019 | 0.872489 | 0.000424 | 0.000845 | 0.958216 |
|  | rs12818067 | 12 | 1.24E+08 | 1.02E-14 | 0.002766 | 0.892556 | 0.000451 | 0.000798 | 0.962346 |
|  | rs12821431 | 12 | 1.24E+08 | 1.75E-15 | 0.015654 | 0.610556 | 0.001073 | 0.000356 | 0.979664 |
|  | rs12824685 | 12 | 1.24E+08 | 3.59E-15 | 0.007741 | 0.816955 | 0.001517 | 0.000259 | 0.987912 |
|  | rs12824957 | 12 | 1.24E+08 | 3.49E-15 | 0.007957 | 0.793476 | 0.000867 | 0.000434 | 0.974257 |
|  | rs12829456 | 12 | 1.24E+08 | 1.63E-14 | 0.001741 | 0.930177 | 0.000485 | 0.000745 | 0.966189 |
|  | rs1402274 | 12 | 1.24E+08 | 3.94E-14 | 0.000731 | 0.964785 | 6.98E-05 | 0.004606 | 0.710404 |
|  | rs1402275 | 12 | 1.24E+08 | 1.23E-14 | 0.002306 | 0.905342 | 0.000231 | 0.001493 | 0.919385 |
|  | rs1533703 | 12 | 1.24E+08 | 2.4E-14 | 0.001192 | 0.945073 | 0.000126 | 0.002647 | 0.862231 |
|  | rs1568427 | 12 | 1.24E+08 | 1.61E-15 | 0.017001 | 0.578096 | 0.000254 | 0.001364 | 0.9249 |
|  | rs1569068 | 12 | 1.24E+08 | 5.28E-14 | 0.000549 | 0.973124 | 1.88E-05 | 0.015992 | 0.145098 |
|  | rs1609520 | 12 | 1.24E+08 | 3.25E-15 | 0.008528 | 0.761172 | 0.000307 | 0.001141 | 0.941581 |
|  | rs1615350 | 12 | 1.24E+08 | 8.63E-15 | 0.003259 | 0.866427 | 0.000163 | 0.002068 | 0.893201 |
|  | rs1615694 | 12 | 1.24E+08 | 8.83E-14 | 0.000331 | 0.987148 | 5.44E-05 | 0.005832 | 0.63086 |
|  | rs1616131 | 12 | 1.24E+08 | 2.96E-14 | 0.000971 | 0.953491 | 2.17E-05 | 0.013958 | 0.263104 |
|  | rs1616181 | 12 | 1.24E+08 | 2.98E-14 | 0.000963 | 0.955418 | 2.17E-05 | 0.013964 | 0.249147 |
|  | rs1617434 | 12 | 1.24E+08 | 4.2E-14 | 0.000687 | 0.968317 | 0.000219 | 0.001571 | 0.916326 |
|  | rs1619561 | 12 | 1.24E+08 | 1.44E-14 | 0.00197 | 0.915757 | 5.06E-06 | 0.056108 | 0.056108 |
|  | rs1627724 | 12 | 1.24E+08 | 2.98E-14 | 0.000964 | 0.954455 | 2.65E-05 | 0.011559 | 0.442167 |
|  | rs1630905 | 12 | 1.24E+08 | 8.8E-14 | 0.000332 | 0.986817 | 6.62E-05 | 0.004841 | 0.677364 |
|  | rs1716160 | 12 | 1.24E+08 | 7.23E-14 | 0.000403 | 0.982901 | 7.23E-05 | 0.004459 | 0.728358 |
|  | rs1716165 | 12 | 1.24E+08 | 9.51E-14 | 0.000308 | 0.988755 | 2.85E-05 | 0.010767 | 0.497074 |
|  | rs1716167 | 12 | 1.24E+08 | 9.64E-14 | 0.000304 | 0.989059 | 3.73E-05 | 0.008346 | 0.52478 |
|  | rs1716168 | 12 | 1.24E+08 | 9.8E-14 | 0.000299 | 0.989958 | 5.97E-05 | 0.005339 | 0.647335 |
|  | rs1716169 | 12 | 1.24E+08 | 2.67E-14 | 0.001073 | 0.949524 | 0.000429 | 0.000836 | 0.959892 |
|  | rs1716170 | 12 | 1.24E+08 | 3.21E-14 | 0.000897 | 0.95906 | 8.05E-05 | 0.004026 | 0.778841 |
|  | rs1716171 | 12 | 1.24E+08 | 3.18E-14 | 0.000904 | 0.958164 | 8.05E-05 | 0.004026 | 0.774814 |
|  | rs1716172 | 12 | 1.24E+08 | 2.92E-14 | 0.000982 | 0.95252 | 8.58E-05 | 0.003789 | 0.814355 |
|  | rs1716173 | 12 | 1.24E+08 | 4.05E-14 | 0.000713 | 0.966219 | 4.87E-05 | 0.006479 | 0.569644 |
|  | rs1716176 | 12 | 1.24E+08 | 6.32E-14 | 0.00046 | 0.981178 | 7.88E-05 | 0.004108 | 0.762581 |
|  | rs1716177 | 12 | 1.24E+08 | 6.26E-14 | 0.000464 | 0.980255 | 7.73E-05 | 0.004183 | 0.754327 |
|  | rs1716182 | 12 | 1.24E+08 | 5.29E-14 | 0.000548 | 0.973672 | 8.35E-05 | 0.00389 | 0.810566 |
|  | rs1727290 | 12 | 1.24E+08 | 5.84E-14 | 0.000497 | 0.977858 | 6.86E-05 | 0.004683 | 0.705798 |
|  | rs1727294 | 12 | 1.24E+08 | 9.14E-14 | 0.00032 | 0.988447 | 2.3E-05 | 0.013217 | 0.331597 |
|  | rs1727296 | 12 | 1.24E+08 | 8.29E-14 | 0.000352 | 0.985118 | 2.64E-05 | 0.011596 | 0.430608 |
|  | rs1727302 | 12 | 1.24E+08 | 3.34E-15 | 0.008304 | 0.769476 | 0.000101 | 0.00324 | 0.845311 |
|  | rs1727306 | 12 | 1.24E+08 | 8.87E-14 | 0.00033 | 0.987477 | 5.19E-05 | 0.006105 | 0.606975 |
|  | rs1727309 | 12 | 1.24E+08 | 1.88E-14 | 0.001519 | 0.938457 | 0.000295 | 0.001185 | 0.94044 |
|  | rs1727310 | 12 | 1.24E+08 | 1.67E-14 | 0.001706 | 0.935324 | 0.000341 | 0.001037 | 0.947022 |
|  | rs1727313 | 12 | 1.24E+08 | 1.66E-14 | 0.001718 | 0.933618 | 0.000196 | 0.001743 | 0.906404 |
|  | rs1727314 | 12 | 1.24E+08 | 8.04E-14 | 0.000363 | 0.984766 | 2.08E-05 | 0.014552 | 0.235183 |
|  | rs1727315 | 12 | 1.24E+08 | 5.84E-14 | 0.000497 | 0.977361 | 0.000028 | 0.010957 | 0.46447 |
|  | rs1727316 | 12 | 1.24E+08 | 5.22E-14 | 0.000555 | 0.972575 | 2.55E-05 | 0.011999 | 0.407246 |
|  | rs1727317 | 12 | 1.24E+08 | 1.07E-14 | 0.002641 | 0.900653 | 0.000201 | 0.0017 | 0.909835 |
|  | rs1727318 | 12 | 1.24E+08 | 8.47E-14 | 0.000345 | 0.985463 | 2.03E-05 | 0.0149 | 0.205897 |
|  | rs1727319 | 12 | 1.24E+08 | 9.92E-15 | 0.002842 | 0.887013 | 0.000369 | 0.000961 | 0.951996 |
|  | rs1727320 | 12 | 1.24E+08 | 8.56E-14 | 0.000341 | 0.986148 | 7.22E-05 | 0.004463 | 0.723899 |
|  | rs1727322 | 12 | 1.24E+08 | 3.48E-14 | 0.000827 | 0.961637 | 0.000504 | 0.000718 | 0.966907 |
|  | rs1727323 | 12 | 1.24E+08 | 4.94E-14 | 0.000586 | 0.971459 | 7.24E-05 | 0.004448 | 0.732807 |
|  | rs1727324 | 12 | 1.24E+08 | 9.78E-15 | 0.002882 | 0.884171 | 0.000409 | 0.000873 | 0.957371 |
|  | rs1727325 | 12 | 1.24E+08 | 7.78E-14 | 0.000375 | 0.984032 | 6.86E-05 | 0.004685 | 0.701114 |
|  | rs1727331 | 12 | 1.24E+08 | 2.8E-15 | 0.009865 | 0.733643 | 0.000353 | 0.001004 | 0.950041 |
|  | rs1727332 | 12 | 1.24E+08 | 4.22E-15 | 0.006597 | 0.830875 | 0.000256 | 0.001353 | 0.926253 |
|  | rs1727334 | 12 | 1.24E+08 | 3.25E-14 | 0.000885 | 0.959945 | 8.12E-05 | 0.003994 | 0.794854 |
|  | rs1790090 | 12 | 1.24E+08 | 4.57E-14 | 0.000633 | 0.969632 | 2.18E-05 | 0.013879 | 0.304825 |
|  | rs1790095 | 12 | 1.24E+08 | 8.5E-14 | 0.000344 | 0.985807 | 7.61E-05 | 0.004247 | 0.750144 |
|  | rs1790098 | 12 | 1.24E+08 | 5.44E-14 | 0.000533 | 0.975286 | 7.52E-05 | 0.004292 | 0.745896 |
|  | rs1790100 | 12 | 1.24E+08 | 4.7E-14 | 0.000615 | 0.970873 | 7.89E-05 | 0.004104 | 0.766685 |
|  | rs1790102 | 12 | 1.24E+08 | 8.69E-14 | 0.000336 | 0.986485 | 6.58E-05 | 0.00487 | 0.672523 |
|  | rs1790108 | 12 | 1.24E+08 | 6.45E-14 | 0.000451 | 0.981629 | 2.82E-05 | 0.010891 | 0.486307 |
|  | rs1790109 | 12 | 1.24E+08 | 5.4E-14 | 0.000537 | 0.974753 | 2.8E-05 | 0.010946 | 0.475416 |
|  | rs1790115 | 12 | 1.24E+08 | 1.36E-14 | 0.002081 | 0.911781 | 5.17E-06 | 0.054964 | 0.111073 |
|  | rs1790116 | 12 | 1.24E+08 | 4E-14 | 0.000721 | 0.965506 | 2.6E-05 | 0.011766 | 0.419012 |
|  | rs1790119 | 12 | 1.24E+08 | 7.86E-14 | 0.000371 | 0.984403 | 1.66E-05 | 0.018033 | 0.129106 |
|  | rs1790120 | 12 | 1.24E+08 | 1.56E-14 | 0.001818 | 0.923125 | 0.000197 | 0.001731 | 0.908135 |
|  | rs1790123 | 12 | 1.24E+08 | 2.61E-14 | 0.001098 | 0.948451 | 0.000466 | 0.000774 | 0.963914 |
|  | rs1879380 | 12 | 1.24E+08 | 3.79E-15 | 0.007323 | 0.824277 | 0.000534 | 0.000682 | 0.969008 |
|  | rs1969354 | 12 | 1.24E+08 | 2.49E-14 | 0.001149 | 0.946222 | 0.000124 | 0.002686 | 0.859584 |
|  | rs1969355 | 12 | 1.24E+08 | 1.39E-15 | 0.019712 | 0.507247 | 0.000247 | 0.0014 | 0.920785 |
|  | rs1980251 | 12 | 1.24E+08 | 1.39E-15 | 0.019726 | 0.487535 | 0.000278 | 0.001255 | 0.938002 |
|  | rs1980252 | 12 | 1.24E+08 | 1.61E-14 | 0.001764 | 0.926678 | 0.000149 | 0.002248 | 0.88678 |
|  | rs2049114 | 12 | 1.24E+08 | 6.55E-14 | 0.000444 | 0.982073 | 5.29E-05 | 0.005992 | 0.619042 |
|  | rs2102949 | 12 | 1.24E+08 | 1.19E-14 | 0.002383 | 0.903035 | 0.000552 | 0.000661 | 0.970339 |
|  | rs2337934 | 12 | 1.24E+08 | 1.62E-14 | 0.001758 | 0.928436 | 0.000148 | 0.002264 | 0.884533 |
|  | rs2510885 | 12 | 1.24E+08 | 5.99E-14 | 0.000485 | 0.978343 | 2.02E-05 | 0.014963 | 0.190997 |
|  | rs2682429 | 12 | 1.24E+08 | 6E-14 | 0.000484 | 0.978827 | 2.41E-05 | 0.012627 | 0.395248 |
|  | rs2682431 | 12 | 1.24E+08 | 9.9E-14 | 0.000296 | 0.990253 | 7.12E-05 | 0.004522 | 0.714926 |
|  | rs2682433 | 12 | 1.24E+08 | 1.02E-14 | 0.002764 | 0.89532 | 0.000356 | 0.000994 | 0.951036 |
|  | rs2682434 | 12 | 1.24E+08 | 5.48E-14 | 0.000529 | 0.976347 | 7.39E-05 | 0.004367 | 0.741604 |
|  | rs2695476 | 12 | 1.24E+08 | 1.05E-14 | 0.002691 | 0.898011 | 0.000375 | 0.000947 | 0.952943 |
|  | rs2695478 | 12 | 1.24E+08 | 7.89E-15 | 0.003561 | 0.863168 | 0.000433 | 0.000828 | 0.961548 |
|  | rs2695479 | 12 | 1.24E+08 | 1.35E-14 | 0.002095 | 0.9097 | 0.000339 | 0.001041 | 0.945985 |
|  | rs2695481 | 12 | 1.24E+08 | 5.46E-14 | 0.000531 | 0.975817 | 2.4E-05 | 0.012667 | 0.369963 |
|  | rs2695482 | 12 | 1.24E+08 | 2.79E-14 | 0.001027 | 0.950551 | 2E-05 | 0.015128 | 0.176033 |
|  | rs2851435 | 12 | 1.24E+08 | 2.53E-14 | 0.001131 | 0.947353 | 0.000396 | 0.0009 | 0.953843 |
|  | rs2851438 | 12 | 1.24E+08 | 5.33E-14 | 0.000544 | 0.974216 | 2.41E-05 | 0.012657 | 0.38262 |
|  | rs2851443 | 12 | 1.24E+08 | 9.58E-15 | 0.002943 | 0.875432 | 0.000433 | 0.000828 | 0.960721 |
|  | rs2851447 | 12 | 1.24E+08 | 4.4E-15 | 0.006329 | 0.837203 | 0.000466 | 0.000774 | 0.964687 |
|  | rs2851451 | 12 | 1.24E+08 | 8.98E-14 | 0.000326 | 0.987803 | 6.83E-05 | 0.004704 | 0.696429 |
|  | rs28683528 | 12 | 1.24E+08 | 3.33E-14 | 0.000865 | 0.96081 | 0.001172 | 0.000328 | 0.982403 |
|  | rs2950537 | 12 | 1.24E+08 | 2.07E-14 | 0.001381 | 0.941294 | 5.29E-05 | 0.005987 | 0.625029 |
|  | rs3018098 | 12 | 1.24E+08 | 6.04E-14 | 0.000481 | 0.979791 | 7.89E-05 | 0.004103 | 0.770788 |
|  | rs3018099 | 12 | 1.24E+08 | 1.02E-14 | 0.002777 | 0.88979 | 0.000402 | 0.000888 | 0.954731 |
|  | rs34341465 | 12 | 1.24E+08 | 9.26E-15 | 0.003043 | 0.86947 | 0.000453 | 0.000794 | 0.96314 |
|  | rs34997336 | 12 | 1.24E+08 | 6.81E-15 | 0.004119 | 0.852109 | 0.000998 | 0.000381 | 0.976693 |
|  | rs35067339 | 12 | 1.24E+08 | 8.88E-16 | 0.030593 | 0.385652 | 0.001269 | 0.000305 | 0.983973 |
|  | rs4372492 | 12 | 1.24E+08 | 6.86E-14 | 0.000425 | 0.982498 | 7.28E-05 | 0.00443 | 0.737237 |
|  | rs4460848 | 12 | 1.24E+08 | 1.52E-14 | 0.001863 | 0.917621 | 0.000477 | 0.000757 | 0.965444 |
|  | rs4759371 | 12 | 1.24E+08 | 2.1E-14 | 0.00136 | 0.942654 | 0.000129 | 0.002589 | 0.87009 |
|  | rs4759409 | 12 | 1.24E+08 | 2.91E-14 | 0.000987 | 0.951538 | 8.27E-05 | 0.003923 | 0.802762 |
|  | rs4759415 | 12 | 1.24E+08 | 1.59E-14 | 0.001788 | 0.924913 | 0.000137 | 0.002434 | 0.877536 |
|  | rs4759416 | 12 | 1.24E+08 | 1.65E-14 | 0.001723 | 0.9319 | 0.000143 | 0.002338 | 0.882269 |
|  | rs4759417 | 12 | 1.24E+08 | 1.54E-14 | 0.001842 | 0.921307 | 0.000155 | 0.002175 | 0.891132 |
|  | rs4759420 | 12 | 1.24E+08 | 9.74E-16 | 0.027951 | 0.441713 | 0.001135 | 0.000338 | 0.981741 |
|  | rs58809476 | 12 | 1.24E+08 | 1.76E-14 | 0.001614 | 0.936938 | 0.000543 | 0.00067 | 0.969679 |
|  | rs6633 | 12 | 1.24E+08 | 3.45E-15 | 0.008032 | 0.777508 | 0.000647 | 0.000569 | 0.971488 |
|  | rs67624109 | 12 | 1.24E+08 | 4.64E-15 | 0.00601 | 0.843213 | 0.001485 | 0.000264 | 0.987392 |
|  | rs7137286 | 12 | 1.24E+08 | 1.58E-15 | 0.017394 | 0.543874 | 0.000271 | 0.001285 | 0.935471 |
|  | rs71444568 | 12 | 1.24E+08 | 9.04E-14 | 0.000324 | 0.988127 | 0.001246 | 0.00031 | 0.983667 |
|  | rs71444571 | 12 | 1.24E+08 | 9.68E-16 | 0.02811 | 0.413763 | 0.001437 | 0.000272 | 0.986861 |
|  | rs7304782 | 12 | 1.24E+08 | 2.99E-15 | 0.009248 | 0.752644 | 0.000278 | 0.001253 | 0.939255 |
|  | rs7306755 | 12 | 1.24E+08 | 4.23E-16 | 0.063543 | 0.131472 | 0.001403 | 0.000278 | 0.986312 |
|  | rs7313483 | 12 | 1.24E+08 | 9.8E-14 | 0.000299 | 0.989659 | 0.000126 | 0.002647 | 0.864879 |
|  | rs7953929 | 12 | 1.24E+08 | 1.8E-15 | 0.015293 | 0.625849 | 0.001283 | 0.000302 | 0.984275 |
|  | rs7955457 | 12 | 1.24E+08 | 5.81E-16 | 0.04652 | 0.284015 | 0.00135 | 0.000288 | 0.986034 |
|  | rs7980687 | 12 | 1.24E+08 | 2.84E-15 | 0.009752 | 0.743396 | 0.001573 | 0.00025 | 0.988162 |
|  | rs884548 | 12 | 1.24E+08 | 4.07E-14 | 0.00071 | 0.966929 | 2.18E-05 | 0.013921 | 0.277026 |
|  | rs941305 | 12 | 1.24E+08 | 3.12E-14 | 0.000922 | 0.95634 | 8.05E-05 | 0.004025 | 0.782866 |
|  | rs941306 | 12 | 1.24E+08 | 3.13E-14 | 0.000919 | 0.95726 | 8.11E-05 | 0.003997 | 0.790861 |
|  | rs949142 | 12 | 1.24E+08 | 7.7E-14 | 0.000379 | 0.98328 | 2.18E-05 | 0.013921 | 0.290947 |
|  | rs9739070 | 12 | 1.24E+08 | 3.95E-16 | 0.067928 | 0.067928 | 0.001409 | 0.000277 | 0.986589 |
| rs9401593 | rs12202969 | 6 | 98576223 | 3.57E-21 | 0.074162 | 0.851714 | 2.78E-06 | 0.040789 | 0.740804 |
|  | rs12206087 | 6 | 98582900 | 4.24E-21 | 0.062488 | 0.914202 | 2.64E-06 | 0.042773 | 0.658654 |
|  | rs1487441 | 6 | 98553894 | 2.48E-21 | 0.106317 | 0.512081 | 7.88E-07 | 0.136857 | 0.428278 |
|  | rs1487445 | 6 | 98565211 | 2.12E-21 | 0.123955 | 0.405763 | 8.51E-06 | 0.013951 | 0.908925 |
|  | rs1906252 | 6 | 98550289 | 5.89E-21 | 0.045126 | 0.959329 | 6.95E-07 | 0.154397 | 0.154397 |
|  | rs2388334 | 6 | 98591622 | 6.56E-21 | 0.040578 | 0.999907 | 2.93E-06 | 0.038678 | 0.819922 |
|  | rs9320913 | 6 | 98584733 | 2.05E-21 | 0.128325 | 0.281808 | 2.8E-06 | 0.040439 | 0.781243 |
|  | rs9372734 | 6 | 98577689 | 2.97E-21 | 0.088755 | 0.692205 | 2.74E-06 | 0.041361 | 0.700015 |
|  | rs9375188 | 6 | 98555272 | 3.09E-21 | 0.085348 | 0.777553 | 7.9E-07 | 0.13654 | 0.564818 |
|  | rs9401593 | 6 | 98549801 | 1.71E-21 | 0.153483 | 0.153483 | 7.87E-07 | 0.137024 | 0.291421 |
|  | rs968050 | 6 | 98574560 | 2.89E-21 | 0.091369 | 0.60345 | 2.2E-06 | 0.051063 | 0.61588 |

Abbreviations: probNorm refers to the normalized posterior p-value for each SNP. cumSum means the cumulative normalized posterior p-value for SNP sets. For each sentinel SNP, a 99% credible set of potentially causal SNPs was obtained using the FM-summary method. Each SNP has a corresponding posterior probability, i.e., probNorm, and with each superimposition of another SNP, there is a subsequent cumSum, which is obtained by the the corresponding probNorm of each SNP is summed up. Ultimately, this can be interpreted to mean that there is a 99% probability that the causal SNPs that actually have an effect on the two traits are contained in such a credible set.

## Supplementary Table 13. Results of co-localization analysis of educational attainment (EA) and each neuropsychiatric trait.

| **Model** | **Index SNP** | **CHR** | **Genome position** | **Effect allele** | **Reference allele** | **P_1_** | **P_2_** | **P_meta_** | **PP.H3** | **PP.H4** |
| --- | --- | --- | --- | --- | --- | --- | --- | --- | --- | --- |
| EA_ADHD | rs673253 | 1 | chr1:43929988-44248272 | T | C | 8.28E-10 | 1.53E-11 | 1.17E-14 | 0.22 | **0.78** |
|  | rs3791101 | 1 | chr1:44256468-44383914 | A | G | 7.14E-06 | 5.3E-11 | 7.98E-11 | 0.22 | **0.78** |
|  | rs549845 | 1 | chr1:44012923-44247233 | A | G | 5.78E-08 | 1.38E-10 | 1.832E-10 | 0.22 | **0.78** |
|  | rs62260755 | 3 | chr3:49804290-49917686 | C | G | 3.37E-12 | 6.74E-07 | 1.026E-12 | **0.84** | 0.04 |
|  | rs304137 | 5 | chr5:88161417-88170331 | A | G | 1.78E-10 | 1.79E-06 | 3.228E-16 | **0.69** | 0.31 |
|  | rs12653396 | 5 | chr5:87847273-87896602 | A | T | 2.14E-07 | 1.13E-07 | 3.725E-11 | **0.69** | 0.31 |
|  | rs4839923 | 6 | chr6:98251222-98303699 | A | G | 1.1E-07 | 1.9E-06 | 4.286E-11 | **0.83** | 0.00 |
| EA_AN | rs13093385 | 3 | chr3:49322027-49731861 | A | T | 3.43E-22 | 1E-06 | 4.377E-25 | **1.00** | 0.00 |
|  | rs13096760 | 3 | chr3:49361791-49600426 | T | C | 5.18E-09 | 1.45E-06 | 2.463E-17 | **1.00** | 0.00 |
|  | rs9821797 | 3 | chr3:48446237-49215966 | A | T | 2.4E-07 | 6.99E-15 | 7.55E-15 | **0.94** | 0.06 |
|  | rs73073015 | 3 | chr3:49220330-49877585 | A | G | 9.32E-08 | 3.24E-13 | 3.887E-13 | **1.00** | 0.00 |
|  | rs9832454 | 3 | chr3:50008118-50316007 | T | C | 2.27E-07 | 1.68E-07 | 1.428E-11 | **1.00** | 0.00 |
|  | rs6967776 | 7 | chr7:133233953-133315895 | A | G | 1.54E-07 | 9.43E-06 | 7.207E-10 | 0.21 | **0.77** |
|  | rs705696 | 12 | chr12:56480648-56480648 | A | G | 2.5E-11 | 7.67E-06 | 9.125E-12 | 0.09 | **0.87** |
| EA_ASD | rs9320913 | 6 | chr6:98549801-98591622 | A | C | 2.05E-21 | 1.51E-06 | 1.196E-36 | 0.01 | **0.98** |
|  | rs1106761 | 8 | chr8:142611971-142630782 | A | G | 4.08E-11 | 7.16E-06 | 1.514E-09 | 0.16 | **0.75** |
|  | rs62057121 | 17 | chr17:43740967-44284641 | A | G | 2.8E-08 | 5.94E-06 | 1.066E-11 | 0.21 | **0.79** |
| EA_AUD | rs2101975 | 4 | chr4:106215370-106217358 | A | G | 2.75E-07 | 5.5E-06 | 1.655E-14 | **0.56** | 0.19 |
|  | rs1338549 | 6 | chr6:98310291-98373765 | T | G | 8.26E-17 | 6.73E-06 | 1.4E-30 | 0.17 | **0.77** |
|  | rs13266268 | 8 | chr8:142611220-142615222 | T | C | 4.1E-11 | 3.95E-06 | 6.634E-12 | 0.19 | 0.43 |
|  | rs113925422 | 17 | chr17:43740967-44284641 | A | T | 1.02E-07 | 2.6E-08 | 3.904E-14 | 0.25 | **0.75** |
|  | rs538628 | 17 | chr17:44786336-44865498 | C | G | 2.16E-08 | 3.27E-08 | 4.408E-14 | 0.14 | **0.86** |
|  | rs1879581 | 17 | chr17:43522361-43545893 | T | C | 2.1E-06 | 5.16E-06 | 5.599E-11 | 0.17 | **0.83** |
| EA_BIP | rs12754946 | 1 | chr1:77989923-77989923 | T | C | 1.77E-06 | 2.39E-06 | 1.19E-10 | 0.01 | **0.99** |
|  | rs9320913 | 6 | chr6:98549801-98591622 | A | C | 2.05E-21 | 1.27E-07 | 5.566E-37 | 0.01 | **0.99** |
|  | rs10429537 | 9 | chr9:23345347-23362311 | C | G | 1.02E-21 | 1.21E-06 | 6.978E-19 | 0.01 | **0.98** |
| EA_CUD | rs12122664 | 1 | chr1:91220615-91220791 | A | C | 6.22E-07 | 1.92E-06 | 2.768E-09 | 0.45 | 0.03 |
|  | rs11711407 | 3 | chr3:50181136-50248954 | A | G | 3.73E-15 | 6.41E-06 | 2.34E-23 | **0.95** | 0.00 |
|  | rs35926495 | 3 | chr3:50255663-50337032 | T | C | 1.31E-11 | 2.73E-07 | 1.608E-12 | **0.95** | 0.00 |
|  | rs9467773 | 6 | chr6:26498426-26538268 | A | G | 3E-06 | 9.13E-06 | 1.868E-11 | 0.40 | 0.49 |
|  | rs7783012 | 7 | chr7:114109349-114287116 | A | G | 4.69E-07 | 1.84E-09 | 4.17E-12 | **0.98** | 0.02 |
| EA_MDD | rs7531118 | 1 | chr1:72748669-72838406 | T | C | 1.84E-06 | 2.15E-08 | 4.392E-10 | **0.99** | 0.00 |
|  | rs76025409 | 5 | chr5:103783801-104069917 | C | G | 5.14E-06 | 2.32E-11 | 5.972E-11 | **0.63** | 0.30 |
| EA_PTSD | rs71351952 | 20 | chr20:47523789-47764275 | T | C | 9.6E-06 | 4.94E-06 | 1.772E-09 | 0.47 | 0.44 |
| EA_TS | rs9401593 | 6 | chr6:98549801-98591622 | A | C | 1.71E-21 | 7.87E-07 | 4.122E-35 | 0.04 | **0.96** |
|  | rs12154193 | 6 | chr6:98387276-98545611 | C | G | 1.38E-11 | 9.62E-06 | 3.961E-18 | 0.04 | **0.96** |
|  | rs1619561 | 12 | chr12:123620268-123620270 | C | G | 1.44E-14 | 5.06E-06 | 6.059E-15 | **0.50** | 0.40 |

Note: ***P*_1_** is the educational attainment single-trait P value, ***P*_2_** is the neuropsychiatric traits (ADHD, AN, ASD, AUD, BIP, CUD, MDD, PTSD, or TS) single-trait P value, and ***P*meta** is the cross-trait meta-analysis P value. Chr: chromosome; EA: educational attainment.; ADHD: Attention deficit/hyperactivity disorder; AN: Anorexia nervosa; ASD: Autism spectrum disorders; AUD: Alcohol use disorders; BIP: Bipolar disorder; CUD: Cannabis use disorder; MDD: Major depressive disorder; PTSD: Posttraumatic stress disorder; TS: Tourette’s syndrome. ***PP.H3***: The posterior probability colocalized with different candidate causal variants; ***PP.H4***: The posterior probability colocalized with common candidate causal variants. Yellow background indicates that both ***PP.H4*** and ***PP.H3*** are less than 0.5.

## Supplementary Table 14. Biological process of the shared gene set between EA and neuropsychiatric disorders in Gene Ontology (GO) terms

| **GOID** | **GOTerm** | **Term PValue** | **Term PValue Corrected with Benjamini-Hochberg** | **Group PValue** | **Group PValue Corrected with Benjamini-Hochberg** | **% Associated Genes** | **Nr. Genes** | **Associated Genes Found** |
| --- | --- | --- | --- | --- | --- | --- | --- | --- |
| GO:0050808 | synapse organization | 1.12E-06 | 6.37E-05 | 1.41E-03 | 1.41E-02 | 2.54 | 12.00 | [AMIGO3, BSN, CAMKV, DAG1, LAMB2, MAPT, MEF2C, PLXNB1, PTPRF, RHOA, SEMA3F, STAU1] |
| GO:0050770 | regulation of axonogenesis | 3.24E-05 | 9.24E-04 | 1.41E-03 | 1.41E-02 | 3.47 | 7.00 | [BARHL2, MAPT, PLXNB1, RHOA, SEMA3B, SEMA3F, WNT3] |
| GO:0048675 | axon extension | 3.46E-05 | 6.58E-04 | 1.41E-03 | 1.41E-02 | 4.35 | 6.00 | [BARHL2, LAMB2, MAPT, SEMA3B, SEMA3F, WNT3] |
| GO:0061564 | axon development | 4.01E-05 | 5.72E-04 | 2.25E-03 | 1.12E-02 | 1.94 | 11.00 | [BARHL2, CELSR3, DAG1, LAMB2, MAPT, PLXNB1, PTPRF, RHOA, SEMA3B, SEMA3F, WNT3] |
| GO:0061564 | axon development | 4.01E-05 | 5.72E-04 | 1.41E-03 | 1.41E-02 | 1.94 | 11.00 | [BARHL2, CELSR3, DAG1, LAMB2, MAPT, PLXNB1, PTPRF, RHOA, SEMA3B, SEMA3F, WNT3] |
| GO:0048588 | developmental cell growth | 1.27E-04 | 1.45E-03 | 1.41E-03 | 1.41E-02 | 2.79 | 7.00 | [ARIH2, BARHL2, LAMB2, MAPT, SEMA3B, SEMA3F, WNT3] |
| GO:0060560 | developmental growth involved in morphogenesis | 1.58E-04 | 1.50E-03 | 1.41E-03 | 1.41E-02 | 2.69 | 7.00 | [BARHL2, LAMB2, MAPT, MST1, SEMA3B, SEMA3F, WNT3] |
| GO:0008361 | regulation of cell size | 2.33E-04 | 1.90E-03 | 1.41E-03 | 1.41E-02 | 3.08 | 6.00 | [BARHL2, MAPT, RHOA, SEMA3B, SEMA3F, WNT3] |
| GO:0050771 | negative regulation of axonogenesis | 3.85E-04 | 2.74E-03 | 1.41E-03 | 1.41E-02 | 5.19 | 4.00 | [RHOA, SEMA3B, SEMA3F, WNT3] |
| GO:0007411 | axon guidance | 4.09E-04 | 2.59E-03 | 1.41E-03 | 1.41E-02 | 2.30 | 7.00 | [CELSR3, DAG1, LAMB2, PLXNB1, SEMA3B, SEMA3F, WNT3] |
| GO:0120035 | regulation of plasma membrane bounded cell projection organization | 4.30E-04 | 2.45E-03 | 1.41E-03 | 1.41E-02 | 1.48 | 11.00 | [BARHL2, MAPT, MEF2C, NCKIPSD, PLEKHM1, PLXNB1, PTPRF, RHOA, SEMA3B, SEMA3F, WNT3] |
| GO:0022604 | regulation of cell morphogenesis | 6.09E-04 | 3.15E-03 | 1.41E-03 | 1.41E-02 | 1.68 | 9.00 | [BARHL2, DAG1, MAPT, PLXNB1, QRICH1, RHOA, SEMA3B, SEMA3F, WNT3] |
| GO:0048678 | response to axon injury | 6.39E-04 | 3.04E-03 | 2.25E-03 | 1.12E-02 | 4.55 | 4.00 | [DAG1, GNAI2, LAMB2, PTPRF] |
| GO:0050807 | regulation of synapse organization | 7.65E-04 | 3.36E-03 | 1.41E-03 | 1.41E-02 | 2.46 | 6.00 | [AMIGO3, CAMKV, DAG1, MEF2C, RHOA, SEMA3F] |
| GO:0048666 | neuron development | 8.97E-04 | 3.65E-03 | 1.41E-03 | 1.41E-02 | 1.15 | 14.00 | [BARHL2, CELSR3, DAG1, GNAT1, LAMB2, MAPT, MEF2C, NCKIPSD, PLXNB1, PTPRF, RHOA, SEMA3B, SEMA3F, WNT3] |
| GO:0032989 | cellular component morphogenesis | 1.35E-03 | 5.14E-03 | 2.25E-03 | 1.12E-02 | 1.29 | 11.00 | [BARHL2, CELSR3, DAG1, LAMB2, MAPT, MEF2C, PLXNB1, RHOA, SEMA3B, SEMA3F, WNT3] |
| GO:0032989 | cellular component morphogenesis | 1.35E-03 | 5.14E-03 | 1.41E-03 | 1.41E-02 | 1.29 | 11.00 | [BARHL2, CELSR3, DAG1, LAMB2, MAPT, MEF2C, PLXNB1, RHOA, SEMA3B, SEMA3F, WNT3] |
| GO:0000902 | cell morphogenesis | 1.40E-03 | 4.98E-03 | 1.41E-03 | 1.41E-02 | 1.15 | 13.00 | [BARHL2, CELSR3, COL7A1, DAG1, LAMB2, MAPT, MEF2C, PLXNB1, QRICH1, RHOA, SEMA3B, SEMA3F, WNT3] |
| GO:0051960 | regulation of nervous system development | 1.74E-03 | 5.84E-03 | 1.41E-03 | 1.41E-02 | 1.18 | 12.00 | [AMIGO3, BARHL2, DAG1, MAPT, MEF2C, NCKIPSD, PLXNB1, PTPRF, RHOA, SEMA3B, SEMA3F, WNT3] |
| GO:0022603 | regulation of anatomical structure morphogenesis | 1.95E-03 | 6.18E-03 | 1.41E-03 | 1.41E-02 | 1.11 | 13.00 | [BARHL2, CELSR3, DAG1, FOXP2, HYAL1, MAPT, MEF2C, PLXNB1, QRICH1, RHOA, SEMA3B, SEMA3F, WNT3] |
| GO:0060485 | mesenchyme development | 1.95E-03 | 5.86E-03 | 1.41E-03 | 1.41E-02 | 2.05 | 6.00 | [DAG1, ERBB3, EXOC4, MEF2C, SEMA3B, SEMA3F] |
| GO:0030308 | negative regulation of cell growth | 1.99E-03 | 5.66E-03 | 1.41E-03 | 1.41E-02 | 2.50 | 5.00 | [HYAL1, IP6K2, SEMA3B, SEMA3F, WNT3] |
| GO:0040013 | negative regulation of locomotion | 3.63E-03 | 9.85E-03 | 1.41E-03 | 1.41E-02 | 1.81 | 6.00 | [DAG1, MEF2C, RHOA, SEMA3B, SEMA3F, WNT3] |
| GO:0051271 | negative regulation of cellular component movement | 3.79E-03 | 9.82E-03 | 1.41E-03 | 1.41E-02 | 1.79 | 6.00 | [DAG1, MEF2C, RHOA, SEMA3B, SEMA3F, WNT3] |
| GO:0006935 | chemotaxis | 3.89E-03 | 9.64E-03 | 1.41E-03 | 1.41E-02 | 1.28 | 9.00 | [CELSR3, DAG1, LAMB2, MST1, PLXNB1, RHOA, SEMA3B, SEMA3F, WNT3] |
| GO:0010001 | glial cell differentiation | 4.19E-03 | 9.96E-03 | 2.25E-03 | 1.12E-02 | 2.10 | 5.00 | [DAG1, ERBB3, LAMB2, MAPT, RHOA] |
| GO:0010001 | glial cell differentiation | 4.19E-03 | 9.96E-03 | 1.41E-03 | 1.41E-02 | 2.10 | 5.00 | [DAG1, ERBB3, LAMB2, MAPT, RHOA] |
| GO:0040008 | regulation of growth | 4.80E-03 | 1.09E-02 | 1.41E-03 | 1.41E-02 | 1.24 | 9.00 | [BARHL2, HYAL1, IP6K2, MAPT, MEF2C, RHOA, SEMA3B, SEMA3F, WNT3] |
| GO:0051962 | positive regulation of nervous system development | 4.97E-03 | 1.09E-02 | 9.23E-03 | 3.08E-02 | 1.34 | 8.00 | [AMIGO3, DAG1, MAPT, MEF2C, NCKIPSD, PLXNB1, RHOA, WNT3] |
| GO:0051962 | positive regulation of nervous system development | 4.97E-03 | 1.09E-02 | 1.41E-03 | 1.41E-02 | 1.34 | 8.00 | [AMIGO3, DAG1, MAPT, MEF2C, NCKIPSD, PLXNB1, RHOA, WNT3] |
| GO:0048638 | regulation of developmental growth | 5.44E-03 | 1.15E-02 | 1.41E-03 | 1.41E-02 | 1.66 | 6.00 | [BARHL2, MAPT, MEF2C, SEMA3B, SEMA3F, WNT3] |
| GO:0040012 | regulation of locomotion | 5.94E-03 | 1.21E-02 | 1.41E-03 | 1.41E-02 | 1.06 | 11.00 | [DAG1, ERBB3, GNAI2, HYAL1, MEF2C, MST1, PLXNB1, RHOA, SEMA3B, SEMA3F, WNT3] |
| GO:0071214 | cellular response to abiotic stimulus | 6.94E-03 | 1.36E-02 | 1.34E-02 | 2.69E-02 | 1.58 | 6.00 | [DAG1, GNAT1, HYAL1, HYAL3, SLC38A3, TREX1] |
| GO:0051270 | regulation of cellular component movement | 8.36E-03 | 1.59E-02 | 1.41E-03 | 1.41E-02 | 1.01 | 11.00 | [DAG1, ERBB3, GNAI2, HYAL1, MEF2C, MST1, PLXNB1, RHOA, SEMA3B, SEMA3F, WNT3] |
| GO:0014706 | striated muscle tissue development | 8.42E-03 | 1.55E-02 | 1.41E-03 | 1.41E-02 | 1.52 | 6.00 | [ERBB3, FOXP2, GPX1, MEF2C, RHOA, USP19] |
| GO:0045927 | positive regulation of growth | 8.48E-03 | 1.51E-02 | 9.23E-03 | 3.08E-02 | 1.77 | 5.00 | [HYAL1, MAPT, MEF2C, RHOA, WNT3] |
| GO:0045927 | positive regulation of growth | 8.48E-03 | 1.51E-02 | 1.41E-03 | 1.41E-02 | 1.77 | 5.00 | [HYAL1, MAPT, MEF2C, RHOA, WNT3] |
| GO:0015718 | monocarboxylic acid transport | 9.71E-03 | 1.68E-02 | 9.71E-03 | 2.43E-02 | 2.14 | 4.00 | [EPRS1, SLC25A20, SLC26A6, SLC38A3] |
| GO:0007517 | muscle organ development | 9.89E-03 | 1.66E-02 | 1.41E-03 | 1.41E-02 | 1.46 | 6.00 | [ERBB3, FOXP2, GPX1, MEF2C, RHOA, USP19] |
| GO:0090066 | regulation of anatomical structure size | 1.19E-02 | 1.93E-02 | 1.41E-03 | 1.41E-02 | 1.26 | 7.00 | [BARHL2, GPX1, MAPT, RHOA, SEMA3B, SEMA3F, WNT3] |
| GO:0031346 | positive regulation of cell projection organization | 1.20E-02 | 1.91E-02 | 1.41E-03 | 1.41E-02 | 1.40 | 6.00 | [MAPT, NCKIPSD, PLEKHM1, PLXNB1, RHOA, WNT3] |
| GO:0001667 | ameboidal-type cell migration | 1.27E-02 | 1.95E-02 | 9.23E-03 | 3.08E-02 | 1.39 | 6.00 | [GPX1, HYAL1, MEF2C, RHOA, SEMA3B, SEMA3F] |
| GO:0001667 | ameboidal-type cell migration | 1.27E-02 | 1.95E-02 | 1.41E-03 | 1.41E-02 | 1.39 | 6.00 | [GPX1, HYAL1, MEF2C, RHOA, SEMA3B, SEMA3F] |
| GO:0019933 | cAMP-mediated signaling | 1.41E-02 | 2.12E-02 | 1.43E-02 | 2.38E-02 | 1.91 | 4.00 | [CRHR1, GNAI2, PRKAR2A, UCN2] |
| GO:0009611 | response to wounding | 1.68E-02 | 2.45E-02 | 2.25E-03 | 1.12E-02 | 1.08 | 8.00 | [DAG1, ERBB3, GNAI2, GPX1, LAMB2, PRKAR2A, PTPRF, RHOA] |
| GO:0051216 | cartilage development | 1.80E-02 | 2.57E-02 | 9.23E-03 | 3.08E-02 | 1.78 | 4.00 | [COL7A1, HYAL1, HYAL3, MEF2C] |
| GO:0048762 | mesenchymal cell differentiation | 1.91E-02 | 2.66E-02 | 1.41E-03 | 1.41E-02 | 1.75 | 4.00 | [DAG1, MEF2C, SEMA3B, SEMA3F] |
| GO:0006163 | purine nucleotide metabolic process | 2.01E-02 | 2.72E-02 | 3.18E-02 | 3.98E-02 | 1.25 | 6.00 | [AK5, GPX1, IMPDH2, PFKFB4, RHOA, TREX1] |
| GO:0060348 | bone development | 2.02E-02 | 2.68E-02 | 9.23E-03 | 3.08E-02 | 1.72 | 4.00 | [COL7A1, MEF2C, PLXNB1, RHOA] |
| GO:0060348 | bone development | 2.02E-02 | 2.68E-02 | 1.41E-03 | 1.41E-02 | 1.72 | 4.00 | [COL7A1, MEF2C, PLXNB1, RHOA] |
| GO:0009416 | response to light stimulus | 2.04E-02 | 2.64E-02 | 1.34E-02 | 2.69E-02 | 1.42 | 5.00 | [GNAT1, GPX1, HYAL1, HYAL3, TREX1] |
| GO:0050920 | regulation of chemotaxis | 2.29E-02 | 2.90E-02 | 1.41E-03 | 1.41E-02 | 1.65 | 4.00 | [MST1, SEMA3B, SEMA3F, WNT3] |
| GO:0007188 | adenylate cyclase-modulating G protein-coupled receptor signaling pathway | 2.38E-02 | 2.95E-02 | 1.43E-02 | 2.38E-02 | 1.63 | 4.00 | [CRHR1, GNAI2, GNAT1, UCN2] |
| GO:0000398 | mRNA splicing, via spliceosome | 2.39E-02 | 2.90E-02 | 2.39E-02 | 3.41E-02 | 1.36 | 5.00 | [ELAVL2, RBM5, RBM6, STH, USP4] |
| GO:0019932 | second-messenger-mediated signaling | 2.44E-02 | 2.89E-02 | 1.43E-02 | 2.38E-02 | 1.20 | 6.00 | [CRHR1, ERBB3, GNAI2, MAPT, PRKAR2A, UCN2] |
| GO:0055086 | nucleobase-containing small molecule metabolic process | 3.18E-02 | 3.70E-02 | 3.18E-02 | 3.98E-02 | 1.03 | 7.00 | [AK5, GMPPB, GPX1, IMPDH2, PFKFB4, RHOA, TREX1] |
| GO:0001501 | skeletal system development | 3.94E-02 | 4.49E-02 | 9.23E-03 | 3.08E-02 | 1.07 | 6.00 | [COL7A1, HYAL1, HYAL3, MEF2C, PLXNB1, RHOA] |
| GO:0001501 | skeletal system development | 3.94E-02 | 4.49E-02 | 1.41E-03 | 1.41E-02 | 1.07 | 6.00 | [COL7A1, HYAL1, HYAL3, MEF2C, PLXNB1, RHOA] |
| GO:0007611 | learning or memory | 4.13E-02 | 4.62E-02 | 4.13E-02 | 4.59E-02 | 1.37 | 4.00 | [FOXP2, GNAI2, MAPT, MEF2C] |
| GO:0016579 | protein deubiquitination | 4.40E-02 | 4.82E-02 | 4.40E-02 | 4.40E-02 | 1.34 | 4.00 | [RHOA, RNF123, USP19, USP4] |

## Supplementary Table 15. Horizontal pleiotropy test and heterogeneity assessment in the bidirectional MR analysis between EA and neuropsychiatric disorders.

| Exposure | Outcome | Q Statistic | Q_pvalue | *I*^2^ | Egger intercept | Egger intercept p value |
| --- | --- | --- | --- | --- | --- | --- |
| EA | AD | 67.44 | 0.296439 | 0.081 | 0.001475 | 0.408912 |
| EA | ADHD | 60.98 | 0.211005 | 0.131 | 0.011345 | 0.309701 |
| EA | ALS | 47.03 | 0.824046 | 0 | 0.001732 | 0.893315 |
| EA | AN | 40.55 | 0.578077 | 0 | 0.001027 | 0.937118 |
| EA | ASD | 45.64 | 0.720689 | 0 | -0.00908 | 0.397117 |
| EA | AUD | 160.82 | 3.76E-11 | 0.627 | 0.001043 | 0.452373 |
| EA | BIP | 52.52 | 0.236072 | 0.124 | 0.003639 | 0.780203 |
| EA | CUD | 127.18 | 2.13E-06 | 0.512 | -0.00055 | 0.974877 |
| EA | MDD | 70.43 | 0.001072 | 0.46 | 0.005318 | 0.583117 |
| EA | OCD | 19.15 | 0.999744 | 0 | -0.00919 | 0.726121 |
| EA | PTSD | 61.97 | 0.405645 | 0.032 | -0.02504 | **0.024576** |
| EA | SCZ | 48.32 | 0.232667 | 0.131 | 0.001578 | 0.868468 |
| EA | TS | 21.22 | 0.995484 | 0 | -0.03016 | 0.175895 |
| AD | EA | 28.05 | 0.213756 | 0.18 | -0.00107 | 0.387316 |
| ADHD | EA | 21.92 | 0.002617 | 0.681 | -0.0094 | 0.241746 |
| ALS | EA | 0.81 | 0.848004 | 0 | 0.002992 | 0.639441 |
| AN | EA | 36.86 | 5.99E-05 | 0.729 | -0.00588 | 0.660181 |
| ASD | EA | 47.79 | 1.08E-07 | 0.833 | 0.008934 | 0.251463 |
| AUD | EA | 8.9 | 0.113221 | 0.438 | 0.001123 | 0.816845 |
| BIP | EA | 39.1 | 2.44E-05 | 0.744 | -0.00609 | 0.599545 |
| CUD | EA | 24.23 | 0.007016 | 0.587 | -0.01357 | 0.066687 |
| MDD | EA | 21.46 | 8.45E-05 | 0.86 | -0.00258 | 0.925984 |
| OCD | EA | 15.92 | 0.101907 | 0.372 | 0.002994 | 0.466839 |
| PTSD | EA | 6.56 | 0.087392 | 0.543 | 0.007631 | 0.494845 |
| SCZ | EA | 345.28 | 3.51E-40 | 0.815 | -0.00213 | 0.577422 |
| TS | EA | 153.17 | 5.66E-25 | 0.902 | -0.00308 | 0.589325 |

Abbreviation: ADHD: Attention deficit/hyperactivity disorder; ALS: Amyotrophic lateral sclerosis; AD: Alzheimer’s dementia; AN: Anorexia nervosa; ASD: Autism spectrum disorders; AUD: Alcohol use disorders; BIP: Bipolar disorder; CUD: Cannabis use disorder; MDD: Major depressive disorder; OCD: Obsessive compulsive disorder; PTSD: Posttraumatic stress disorder; TS: Tourette’s syndrome.

## Supplementary Table 16. Causal inference between EA and neuropsychiatric traits using Two-sample MR, MR-RAPS and MR-PRESSO methods

| Exposure | Outcome | Method | N_snp | Causal_Effect_Size | 95% CI | P_value |
| --- | --- | --- | --- | --- | --- | --- |
| EA | AD | Inverse variance weighted | 63 | -0.059 | (-0.098, -0.02) | 0.002795608 |
| EA | AD | Simple median | 63 | -0.059 | (-0.115, -0.003) | 0.03786041 |
| EA | AD | Weighted median | 63 | -0.059 | (-0.113, -0.005) | 0.03226869 |
| EA | AD | Weighted mode | 63 | -0.059 | (-0.153, 0.034) | 0.211575964 |
| EA | AD | MR-Egger | 63 | -0.142 | (-0.340, 0.057) | 0.161950487 |
| EA | AD | MR-RAPS | 63 | -0.061 | (-0.100, -0.020) | 0.001783613 |
| EA | AD | MR-PRESSO:raw | 63 | -0.059 | (-0.100, -0.020) | 0.004004306 |
| EA | AD | MR-PRESSO:Outlier-corrected |  |  |  |  |
| EA | ADHD | Inverse variance weighted | 54 | -0.699 | (-0.941, -0.456) | 1.62E-08 |
| EA | ADHD | Simple median | 54 | -0.799 | (-1.136, -0.462) | 3.42E-06 |
| EA | ADHD | Weighted median | 54 | -0.816 | (-1.154, -0.478) | 2.28E-06 |
| EA | ADHD | Weighted mode | 54 | -1.278 | (-2.062, -0.494) | 0.001400593 |
| EA | ADHD | MR-Egger | 54 | -1.333 | (-2.570, -0.097) | 0.034599986 |
| EA | ADHD | MR-RAPS | 54 | -0.718 | (-0.950, -0.480) | 1.76E-09 |
| EA | ADHD | MR-PRESSO:raw | 54 | -0.699 | (-0.940, -0.460) | 6.52E-07 |
| EA | ADHD | MR-PRESSO:Outlier-corrected |  |  |  |  |
| EA | ALS | Inverse variance weighted | 58 | -0.264 | (-0.548, 0.019) | 0.0678765 |
| EA | ALS | Simple median | 58 | -0.208 | (-0.609, 0.193) | 0.308257047 |
| EA | ALS | Weighted median | 58 | -0.211 | (-0.616, 0.195) | 0.308203113 |
| EA | ALS | Weighted mode | 58 | -0.023 | (-0.883, 0.836) | 0.957541092 |
| EA | ALS | MR-Egger | 58 | -0.361 | (-1.788, 1.067) | 0.620489825 |
| EA | ALS | MR-RAPS | 58 | -0.27 | (-0.560, 0.020) | 0.070290017 |
| EA | ALS | MR-PRESSO:raw | 58 | -0.264 | (-0.520, -0.010) | 0.049170391 |
| EA | ALS | MR-PRESSO:Outlier-corrected |  |  |  |  |
| EA | AN | Inverse variance weighted | 44 | 0.333 | (0.078, 0.588) | 0.0105095 |
| EA | AN | Simple median | 44 | 0.324 | (-0.044, 0.692) | 0.08455714 |
| EA | AN | Weighted median | 44 | 0.376 | (0.003, 0.749) | 0.048324173 |
| EA | AN | Weighted mode | 44 | 0.364 | (-0.440, 1.168) | 0.375235976 |
| EA | AN | MR-Egger | 44 | 0.275 | (-1.170, 1.720) | 0.708760865 |
| EA | AN | MR-RAPS | 44 | 0.341 | (0.080, 0.600) | 0.01106429 |
| EA | AN | MR-PRESSO:raw | 44 | 0.333 | (0.090, 0.580) | 0.011659921 |
| EA | AN | MR-PRESSO:Outlier-corrected |  |  |  |  |
| EA | ASD | Inverse variance weighted | 53 | 0.369 | (0.136, 0.602) | 0.001911359 |
| EA | ASD | Simple median | 53 | 0.232 | (-0.107, 0.570) | 0.180155769 |
| EA | ASD | Weighted median | 53 | 0.241 | (-0.095, 0.578) | 0.15952802 |
| EA | ASD | Weighted mode | 53 | 0.089 | (-0.728, 0.907) | 0.830395446 |
| EA | ASD | MR-Egger | 53 | 0.878 | (-0.313, 2.069) | 0.148531827 |
| EA | ASD | MR-RAPS | 53 | 0.378 | (0.140, 0.620) | 0.002033187 |
| EA | ASD | MR-PRESSO:raw | 53 | 0.369 | (0.150, 0.590) | 0.001685498 |
| EA | ASD | MR-PRESSO:Outlier-corrected |  |  |  |  |
| EA | AUD | Inverse variance weighted | 61 | 0.04 | (0.009, 0.071) | 0.010483008 |
| EA | AUD | Simple median | 61 | 0.045 | (0.012, 0.078) | 0.007878161 |
| EA | AUD | Weighted median | 61 | 0.044 | (0.012, 0.077) | 0.007942953 |
| EA | AUD | Weighted mode | 61 | 0.045 | (-0.055, 0.144) | 0.3823316 |
| EA | AUD | MR-Egger | 61 | -0.018 | (-0.172, 0.136) | 0.818768971 |
| EA | AUD | MR-RAPS | 61 | 0.043 | (0.020, 0.060) | 8.96E-06 |
| EA | AUD | MR-PRESSO:raw | 61 | 0.04 | (0.010, 0.070) | 0.013021256 |
| EA | AUD | MR-PRESSO:Outlier-corrected | 59 | 0.037 | (0.010, 0.070) | 0.01653788 |
| EA | BIP | Inverse variance weighted | 47 | 0.245 | (-0.016, 0.506) | 0.066183342 |
| EA | BIP | Simple median | 47 | 0.173 | (-0.199, 0.545) | 0.361835937 |
| EA | BIP | Weighted median | 47 | 0.154 | (-0.213, 0.522) | 0.410518637 |
| EA | BIP | Weighted mode | 47 | 0.922 | (-0.011, 1.855) | 0.05273797 |
| EA | BIP | MR-Egger | 47 | 0.038 | (-1.425, 1.502) | 0.958993318 |
| EA | BIP | MR-RAPS | 47 | 0.252 | (0.000, 0.500) | 0.049459114 |
| EA | BIP | MR-PRESSO:raw | 47 | 0.245 | (-0.020, 0.510) | 0.072648425 |
| EA | BIP | MR-PRESSO:Outlier-corrected |  |  |  |  |
| EA | CUD | Inverse variance weighted | 63 | -0.688 | (-1.058, -0.318) | 0.000271299 |
| EA | CUD | Simple median | 63 | -0.794 | (-1.208, -0.38) | 0.000172029 |
| EA | CUD | Weighted median | 63 | -0.794 | (-1.205, -0.384) | 0.000149022 |
| EA | CUD | Weighted mode | 63 | -0.419 | (-1.360, 0.521) | 0.381934992 |
| EA | CUD | MR-Egger | 63 | -0.657 | (-2.598, 1.284) | 0.507109001 |
| EA | CUD | MR-RAPS | 63 | -0.725 | (-0.990, -0.460) | 6.79E-08 |
| EA | CUD | MR-PRESSO:raw | 63 | -0.688 | (-1.060, -0.320) | 0.00055517 |
| EA | CUD | MR-PRESSO:Outlier-corrected | 61 | -0.789 | (-1.130, -0.440) | 3.43E-05 |
| EA | MDD | Inverse variance weighted | 39 | -0.075 | (-0.290, 0.140) | 0.495625618 |
| EA | MDD | Simple median | 39 | -0.137 | (-0.402, 0.129) | 0.314133635 |
| EA | MDD | Weighted median | 39 | -0.055 | (-0.315, 0.206) | 0.681645393 |
| EA | MDD | Weighted mode | 39 | 0.266 | (-0.404, 0.937) | 0.436140302 |
| EA | MDD | MR-Egger | 39 | -0.375 | (-1.458, 0.709) | 0.497880629 |
| EA | MDD | MR-RAPS | 39 | -0.078 | (-0.240, 0.080) | 0.339563152 |
| EA | MDD | MR-PRESSO:raw | 39 | -0.075 | (-0.290, 0.140) | 0.499757426 |
| EA | MDD | MR-PRESSO:Outlier-corrected |  |  |  |  |
| EA | OCD | Inverse variance weighted | 46 | -0.072 | (-0.678, 0.534) | 0.815028195 |
| EA | OCD | Simple median | 46 | -0.268 | (-1.091, 0.554) | 0.522464641 |
| EA | OCD | Weighted median | 46 | -0.197 | (-1.024, 0.629) | 0.63963289 |
| EA | OCD | Weighted mode | 46 | -0.738 | (-2.567, 1.092) | 0.42928421 |
| EA | OCD | MR-Egger | 46 | 0.427 | (-2.416, 3.270) | 0.768331447 |
| EA | OCD | MR-RAPS | 46 | -0.073 | (-0.700, 0.550) | 0.81945995 |
| EA | OCD | MR-PRESSO:raw | 46 | -0.072 | (-0.470, 0.320) | 0.721564718 |
| EA | OCD | MR-PRESSO:Outlier-corrected |  |  |  |  |
| EA | PTSD | Inverse variance weighted | 61 | 0.016 | (-0.222, 0.254) | 0.895433316 |
| EA | PTSD | Simple median | 61 | -0.029 | (-0.371, 0.314) | 0.870208528 |
| EA | PTSD | Weighted median | 61 | -0.018 | (-0.357, 0.321) | 0.916679469 |
| EA | PTSD | Weighted mode | 61 | -0.23 | (-1.043, 0.583) | 0.578678671 |
| EA | PTSD | MR-Egger | 61 | 1.411 | (0.203, 2.619) | 0.02206891 |
| EA | PTSD | MR-RAPS | 61 | 0.016 | (-0.220, 0.260) | 0.894035024 |
| EA | PTSD | MR-PRESSO:raw | 61 | 0.016 | (-0.220, 0.250) | 0.895873043 |
| EA | PTSD | MR-PRESSO:Outlier-corrected | 61 |  |  |  |
| EA | SCZ | Inverse variance weighted | 43 | 0.152 | (-0.063, 0.368) | 0.166139291 |
| EA | SCZ | Simple median | 43 | 0.185 | (-0.115, 0.486) | 0.227084372 |
| EA | SCZ | Weighted median | 43 | 0.154 | (-0.143, 0.450) | 0.309392956 |
| EA | SCZ | Weighted mode | 43 | 0.014 | (-0.674, 0.703) | 0.967093551 |
| EA | SCZ | MR-Egger | 43 | 0.066 | (-0.971, 1.103) | 0.900437889 |
| EA | SCZ | MR-RAPS | 43 | 0.157 | (-0.050, 0.360) | 0.135701718 |
| EA | SCZ | MR-PRESSO:raw | 43 | 0.152 | (-0.060, 0.370) | 0.173451751 |
| EA | SCZ | MR-PRESSO:Outlier-corrected |  |  |  |  |
| EA | TS | Inverse variance weighted | 42 | 0.009 | (-0.488, 0.505) | 0.973154756 |
| EA | TS | Simple median | 42 | -0.196 | (-0.868, 0.476) | 0.566716593 |
| EA | TS | Weighted median | 42 | 0.009 | (-0.666, 0.684) | 0.979715247 |
| EA | TS | Weighted mode | 42 | -0.712 | (-2.136, 0.713) | 0.327378239 |
| EA | TS | MR-Egger | 42 | 1.698 | (-0.756, 4.153) | 0.17503507 |
| EA | TS | MR-RAPS | 42 | 0.009 | (-0.500, 0.520) | 0.973706873 |
| EA | TS | MR-PRESSO:raw | 42 | 0.009 | (-0.350, 0.370) | 0.962916563 |
| EA | TS | MR-PRESSO:Outlier-corrected |  |  |  |  |
| AD | EA | Inverse variance weighted | 24 | 0.03 | (-0.029, 0.088) | 0.319116529 |
| AD | EA | Simple median | 24 | 0.056 | (-0.026, 0.138) | 0.182301025 |
| AD | EA | Weighted median | 24 | 0.052 | (-0.021, 0.126) | 0.164560183 |
| AD | EA | Weighted mode | 24 | 0.054 | (-0.020, 0.128) | 0.149584886 |
| AD | EA | MR-Egger | 24 | 0.063 | (-0.032, 0.158) | 0.19166152 |
| AD | EA | MR-RAPS | 24 | 0.03 | (-0.020, 0.080) | 0.268271629 |
| AD | EA | MR-PRESSO:raw | 24 | 0.03 | (-0.030, 0.090) | 0.329483269 |
| AD | EA | MR-PRESSO:Outlier-corrected |  |  |  |  |
| ADHD | EA | Inverse variance weighted | 8 | -0.095 | (-0.130, -0.060) | 8.46E-08 |
| ADHD | EA | Simple median | 8 | -0.086 | (-0.117, -0.054) | 9.00E-08 |
| ADHD | EA | Weighted median | 8 | -0.095 | (-0.128, -0.063) | 1.21E-08 |
| ADHD | EA | Weighted mode | 8 | -0.081 | (-0.126, -0.035) | 0.000485051 |
| ADHD | EA | MR-Egger | 8 | 0.011 | (-0.153, 0.174) | 0.897860143 |
| ADHD | EA | MR-RAPS | 8 | -0.1 | (-0.120, -0.080) | 0 |
| ADHD | EA | MR-PRESSO:raw | 8 | -0.095 | (-0.130, -0.060) | 0.001056054 |
| ADHD | EA | MR-PRESSO:Outlier-corrected | 6 | -0.096 | (-0.120, -0.080) | 0.000176707 |
| ALS | EA | Inverse variance weighted | 4 | -0.015 | (-0.037, 0.007) | 0.179914507 |
| ALS | EA | Simple median | 4 | -0.015 | (-0.041, 0.012) | 0.283808311 |
| ALS | EA | Weighted median | 4 | -0.012 | (-0.037, 0.014) | 0.378059355 |
| ALS | EA | Weighted mode | 4 | -0.011 | (-0.041, 0.019) | 0.485724065 |
| ALS | EA | MR-Egger | 4 | -0.033 | (-0.103, 0.036) | 0.346956542 |
| ALS | EA | MR-RAPS | 4 | -0.015 | (-0.040, 0.010) | 0.194641557 |
| ALS | EA | MR-PRESSO:raw | 4 | -0.015 | (-0.030, 0.000) | 0.081284756 |
| ALS | EA | MR-PRESSO:Outlier-corrected |  |  |  |  |
| AN | EA | Inverse variance weighted | 11 | 0.012 | (-0.025, 0.049) | 0.518506198 |
| AN | EA | Simple median | 11 | 0.025 | (-0.009, 0.058) | 0.152174541 |
| AN | EA | Weighted median | 11 | 0.016 | (-0.015, 0.046) | 0.312073239 |
| AN | EA | Weighted mode | 11 | 0.023 | (-0.026, 0.071) | 0.367306825 |
| AN | EA | MR-Egger | 11 | 0.082 | (-0.220, 0.384) | 0.595859336 |
| AN | EA | MR-RAPS | 11 | 0.014 | (-0.010, 0.030) | 0.15947246 |
| AN | EA | MR-PRESSO:raw | 11 | 0.012 | (-0.020, 0.050) | 0.533040525 |
| AN | EA | MR-PRESSO:Outlier-corrected | 9 | 0.013 | (-0.020, 0.040) | 0.440559602 |
| ASD | EA | Inverse variance weighted | 9 | 0.004 | (-0.051, 0.059) | 0.880411561 |
| ASD | EA | Simple median | 9 | -0.013 | (-0.055, 0.029) | 0.539810995 |
| ASD | EA | Weighted median | 9 | -0.012 | (-0.052, 0.027) | 0.533470163 |
| ASD | EA | Weighted mode | 9 | -0.032 | (-0.082, 0.018) | 0.206356096 |
| ASD | EA | MR-Egger | 9 | -0.086 | (-0.238, 0.065) | 0.264546545 |
| ASD | EA | MR-RAPS | 9 | 0.005 | (-0.020, 0.030) | 0.61358763 |
| ASD | EA | MR-PRESSO:raw | 9 | 0.004 | (-0.050, 0.060) | 0.884135706 |
| ASD | EA | MR-PRESSO:Outlier-corrected | 5 | -0.011 | (-0.040, 0.020) | 0.461878351 |
| AUD | EA | Inverse variance weighted | 6 | -0.178 | (-0.539, 0.183) | 0.334375377 |
| AUD | EA | Simple median | 6 | -0.118 | (-0.488, 0.252) | 0.531529644 |
| AUD | EA | Weighted median | 6 | -0.123 | (-0.492, 0.246) | 0.51317452 |
| AUD | EA | Weighted mode | 6 | -0.114 | (-0.635, 0.407) | 0.667585517 |
| AUD | EA | MR-Egger | 6 | -0.277 | (-1.156, 0.603) | 0.537405204 |
| AUD | EA | MR-RAPS | 6 | -0.182 | (-0.460, 0.090) | 0.1955732 |
| AUD | EA | MR-PRESSO:raw | 6 | -0.178 | (-0.540, 0.180) | 0.378711301 |
| AUD | EA | MR-PRESSO:Outlier-corrected |  |  |  |  |
| BIP | EA | Inverse variance weighted | 11 | 0.036 | (0.000, 0.071) | 0.048126444 |
| BIP | EA | Simple median | 11 | 0.025 | (-0.011, 0.062) | 0.169509581 |
| BIP | EA | Weighted median | 11 | 0.051 | (0.019 ,0.084) | 0.001733122 |
| BIP | EA | Weighted mode | 11 | 0.058 | (-0.017, 0.132) | 0.127841997 |
| BIP | EA | MR-Egger | 11 | 0.1 | (-0.136, 0.337) | 0.405204871 |
| BIP | EA | MR-RAPS | 11 | 0.039 | (0.020, 0.060) | 2.44E-05 |
| BIP | EA | MR-PRESSO:raw | 11 | 0.036 | (0.000, 0.070) | 0.076347214 |
| BIP | EA | MR-PRESSO:Outlier-corrected | 8 | 0.02 | (-0.010, 0.050) | 0.203293907 |
| CUD | EA | Inverse variance weighted | 11 | -0.039 | (-0.065, -0.014) | 0.002446471 |
| CUD | EA | Simple median | 11 | -0.047 | (-0.075, -0.019) | 0.001136732 |
| CUD | EA | Weighted median | 11 | -0.034 | (-0.059, -0.009) | 0.007734931 |
| CUD | EA | Weighted mode | 11 | -0.017 | (-0.052, 0.018) | 0.337244826 |
| CUD | EA | MR-Egger | 11 | 0.08 | (-0.034, 0.194) | 0.170567353 |
| CUD | EA | MR-RAPS | 11 | -0.043 | (-0.06, -0.02) | 2.09E-06 |
| CUD | EA | MR-PRESSO:raw | 11 | -0.039 | (-0.06,-0.01) | 0.012679635 |
| CUD | EA | MR-PRESSO:Outlier-corrected | 10 | -0.032 | (-0.05,-0.01) | 0.018119557 |
| MDD | EA | Inverse variance weighted | 4 | 0.01 | (-0.133,0.153) | 0.892088627 |
| MDD | EA | Simple median | 4 | -0.013 | (-0.082,0.056) | 0.716348008 |
| MDD | EA | Weighted median | 4 | -0.027 | (-0.101,0.046) | 0.468945131 |
| MDD | EA | Weighted mode | 4 | -0.056 | (-0.133,0.02) | 0.148752322 |
| MDD | EA | MR-Egger | 4 | 0.061 | (-0.902,1.023) | 0.901776828 |
| MDD | EA | MR-RAPS | 4 | 0.012 | (-0.04,0.06) | 0.667434637 |
| MDD | EA | MR-PRESSO:raw | 4 | 0.01 | (-0.13,0.15) | 0.900679907 |
| MDD | EA | MR-PRESSO:Outlier-corrected | 2 | -0.021 | (-0.16,0.12) | 0.816576369 |
| OCD | EA | Inverse variance weighted | 11 | -0.004 | (-0.015,0.006) | 0.396290008 |
| OCD | EA | Simple median | 11 | -0.011 | (-0.024,0.002) | 0.09198361 |
| OCD | EA | Weighted median | 11 | -0.008 | (-0.02,0.004) | 0.207527633 |
| OCD | EA | Weighted mode | 11 | -0.013 | (-0.034,0.008) | 0.231661268 |
| OCD | EA | MR-Egger | 11 | -0.016 | (-0.048,0.016) | 0.321051682 |
| OCD | EA | MR-RAPS | 11 | -0.005 | (-0.01,0) | 0.270524384 |
| OCD | EA | MR-PRESSO:raw | 11 | -0.004 | (-0.01,0.01) | 0.416132454 |
| OCD | EA | MR-PRESSO:Outlier-corrected |  |  |  |  |
| PTSD | EA | Inverse variance weighted | 4 | -0.034 | (-0.083,0.014) | 0.166186436 |
| PTSD | EA | Simple median | 4 | -0.045 | (-0.085,-0.004) | 0.032754362 |
| PTSD | EA | Weighted median | 4 | -0.033 | (-0.075,0.01) | 0.137852011 |
| PTSD | EA | Weighted mode | 4 | -0.005 | (-0.061,0.051) | 0.864622906 |
| PTSD | EA | MR-Egger | 4 | -0.093 | (-0.242,0.056) | 0.219273623 |
| PTSD | EA | MR-RAPS | 4 | -0.037 | (-0.07,0) | 0.039444101 |
| PTSD | EA | MR-PRESSO:raw | 4 | -0.034 | (-0.08,0.01) | 0.260195331 |
| PTSD | EA | MR-PRESSO:Outlier-corrected |  |  |  |  |
| SCZ | EA | Inverse variance weighted | 65 | 0.003 | (-0.017,0.023) | 0.772057695 |
| SCZ | EA | Simple median | 65 | 0.01 | (-0.006,0.026) | 0.228021714 |
| SCZ | EA | Weighted median | 65 | 0 | (-0.016,0.016) | 1 |
| SCZ | EA | Weighted mode | 65 | -0.009 | (-0.049,0.031) | 0.665865231 |
| SCZ | EA | MR-Egger | 65 | 0.03 | (-0.066,0.125) | 0.543100825 |
| SCZ | EA | MR-RAPS | 65 | 0.003 | (0,0.01) | 0.42001586 |
| SCZ | EA | MR-PRESSO:raw | 65 | 0.003 | (-0.02,0.02) | 0.77299413 |
| SCZ | EA | MR-PRESSO:Outlier-corrected | 57 | -0.004 | (-0.02,0.01) | 0.640270472 |
| TS | EA | Inverse variance weighted | 16 | -0.015 | (-0.043,0.014) | 0.322352686 |
| TS | EA | Simple median | 16 | 0.001 | (-0.013,0.015) | 0.875064991 |
| TS | EA | Weighted median | 16 | -0.001 | (-0.016,0.013) | 0.854444396 |
| TS | EA | Weighted mode | 16 | -0.002 | (-0.022,0.017) | 0.828809931 |
| TS | EA | MR-Egger | 16 | 0.001 | (-0.061,0.062) | 0.984020515 |
| TS | EA | MR-RAPS | 16 | -0.026 | (-0.03,-0.02) | 1.65E-09 |
| TS | EA | MR-PRESSO:raw | 16 | -0.015 | (-0.04,0.01) | 0.338048831 |
| TS | EA | MR-PRESSO:Outlier-corrected | 14 | -0.002 | (-0.01,0.01) | 0.715377001 |

Note: *The causal effect size was the beta coefficient from linear or logistic regression models for corresponding outcome. N_snp: number of instrumental variables; The threshold of significance was at the Bonferroni-adjusted level P-value < 0.0019(0.05/13). MR-PRESSO: Outlier-corrected: Null indicates that no polytropic variant exists; ADHD: Attention deficit/hyperactivity disorder; ALS: Amyotrophic lateral sclerosis; AD: Alzheimer’s dementia; AN: Anorexia nervosa; ASD: Autism spectrum disorders; AUD: Alcohol use disorders; BIP: Bipolar disorder; CUD: Cannabis use disorder; MDD: Major depressive disorder; OCD: Obsessive compulsive disorder; PTSD: Posttraumatic stress disorder; TS: Tourette’s syndrome.

## Supplementary Table 17. Causal inference between educational attainment (EA) and neuropsychiatric traits after removing SNPs associated with potential confounding diseases or traits.

| Exposure | Outcome | Method | N_snp | Causal_Effect_Size | 95% CI | P_value |
| --- | --- | --- | --- | --- | --- | --- |
| EA | AD | IVW | 29 | -0.086 | (-0.155, -0.018) | **0.013419** |
| EA | ADHD | IVW | 26 | -0.551 | (-0.929, -0.172) | **0.004348** |
| EA | ALS | IVW | 28 | -0.268 | (-0.711, 0.174) | 0.23494 |
| EA | AN | IVW | 21 | 0.263 | (-0.131, 0.656) | 0.190866 |
| EA | ASD | IVW | 25 | 0.429 | (0.064, 0.793) | **0.021251** |
| EA | AUD | IVW | 29 | 0.022 | (-0.026, 0.071) | 0.362363 |
| EA | BIP | IVW | 23 | 0.281 | (-0.102, 0.664) | 0.150276 |
| EA | CUD | IVW | 29 | -0.672 | (-1.189, -0.155) | **0.010814** |
| EA | MDD | IVW | 19 | -0.004 | (-0.326, 0.319) | 0.983007 |
| EA | OCD | IVW | 22 | -0.362 | (-1.321, 0.597) | 0.459049 |
| EA | PTSD | IVW | 29 | 0.004 | (-0.368, 0.375) | 0.984435 |
| EA | SCZ | IVW | 20 | 0.231 | (-0.094, 0.556) | 0.16347 |
| EA | TS | IVW | 22 | 0.119 | (-0.610, 0.848) | 0.749123 |
| AD | EA | IVW | 18 | 0.046 | (-0.036, 0.129) | 0.271919 |
| ADHD | EA | IVW | 2 | -0.066 | (-0.105, -0.027) | **0.000956** |
| ASD | EA | IVW | 5 | -0.004 | (-0.075, 0.067) | 0.90785 |
| AUD | EA | IVW | 3 | -0.234 | (-0.577, 0.109) | 0.181795 |
| BIP | EA | IVW | 6 | 0.053 | (0.015, 0.091) | **0.006015** |
| CUD | EA | IVW | 7 | -0.028 | (-0.056, 0.000) | 0.052044 |
| SCZ | EA | IVW | 47 | 0.005 | (-0.013, 0.023) | 0.596056 |

Abbreviation: ADHD: Attention deficit/hyperactivity disorder; ALS: Amyotrophic lateral sclerosis; AD: Alzheimer’s dementia; AN: Anorexia nervosa; ASD: Autism spectrum disorders; AUD: Alcohol use disorders; BIP: Bipolar disorder; CUD: Cannabis use disorder; MDD: Major depressive disorder; OCD: Obsessive compulsive disorder; PTSD: Posttraumatic stress disorder; TS: Tourette’s syndrome.

## Supplementary Table 18. No. of TWAS significant genes for educational attainment (EA) and neuropsychiatric traits, and No. of shared TWAS significant genes between EA and related neuropsychiatric traits across 48 GTEx tissues.

| Tissue | EA | ADHD | | ALS | | AD | | AN | | ASD | | AUD | | BIP | | CUD | | MDD | | OCD | | PTSD | | TS | |
| --- | --- | --- | --- | --- | --- | --- | --- | --- | --- | --- | --- | --- | --- | --- | --- | --- | --- | --- | --- | --- | --- | --- | --- | --- | --- |
|  | **Freq** | **Freq** | **overlap** | **Freq** | **overlap** | **Freq** | **overlap** | **Freq** | **overlap** | **Freq** | **overlap** | **Freq** | **overlap** | **Freq** | **overlap** | **Freq** | **overlap** | **Freq** | **overlap** | **Freq** | **overlap** | **Freq** | **overlap** | **Freq** | **overlap** |
| Adipose Subcutaneous | 155 | 15 | 9 | 2 | 0 | 56 | 5 | 18 | 17 | 11 | 9 | 26 | 16 | 32 | 2 | 3 | 3 | 7 | 1 | 0 | 0 | 0 | 0 | 0 | 0 |
| Adipose Visceral Omentum | 142 | 19 | 12 | 0 | 0 | 56 | 5 | 19 | 18 | 8 | 8 | 21 | 12 | 34 | 4 | 2 | 2 | 3 | 0 | 0 | 0 | 0 | 0 | 1 | 0 |
| Adrenal Gland | 114 | 7 | 1 | 2 | 0 | 62 | 3 | 14 | 12 | 12 | 9 | 27 | 14 | 25 | 2 | 6 | 4 | 1 | 0 | 0 | 0 | 0 | 0 | 0 | 0 |
| Artery Aorta | 145 | 12 | 5 | 1 | 0 | 42 | 2 | 26 | 24 | 11 | 9 | 22 | 15 | 47 | 2 | 5 | 4 | 7 | 0 | 0 | 0 | 0 | 0 | 0 | 0 |
| Artery Coronary | 93 | 12 | 5 | 0 | 0 | 39 | 2 | 23 | 20 | 10 | 7 | 16 | 11 | 39 | 1 | 2 | 2 | 4 | 1 | 0 | 0 | 0 | 0 | 2 | 0 |
| Artery Tibial | 158 | 14 | 8 | 0 | 0 | 39 | 1 | 31 | 25 | 6 | 5 | 25 | 14 | 43 | 1 | 2 | 1 | 8 | 0 | 0 | 0 | 0 | 0 | 1 | 0 |
| Brain Amygdala | 81 | 9 | 3 | 0 | 0 | 33 | 6 | 14 | 10 | 13 | 11 | 19 | 12 | 24 | 2 | 3 | 3 | 3 | 0 | 0 | 0 | 0 | 0 | 1 | 0 |
| Brain Anterior cingulate cortex BA24 | 112 | 6 | 2 | 2 | 0 | 53 | 3 | 18 | 16 | 9 | 7 | 25 | 15 | 35 | 3 | 2 | 2 | 2 | 0 | 0 | 0 | 0 | 0 | 0 | 0 |
| Brain Caudate basal ganglia | 120 | 7 | 4 | 0 | 0 | 66 | 9 | 19 | 16 | 12 | 11 | 27 | 14 | 24 | 1 | 2 | 2 | 1 | 0 | 0 | 0 | 0 | 0 | 0 | 0 |
| Brain Cerebellar Hemisphere | 129 | 14 | 5 | 1 | 0 | 44 | 4 | 16 | 13 | 20 | 17 | 36 | 21 | 24 | 1 | 3 | 2 | 2 | 2 | 0 | 0 | 0 | 0 | 0 | 0 |
| Brain Cerebellum | 142 | 7 | 3 | 0 | 0 | 59 | 9 | 22 | 17 | 24 | 20 | 36 | 24 | 43 | 3 | 3 | 2 | 3 | 1 | 0 | 0 | 0 | 0 | 0 | 0 |
| Brain Cortex | 119 | 4 | 4 | 1 | 0 | 33 | 3 | 18 | 17 | 12 | 12 | 30 | 15 | 20 | 2 | 2 | 2 | 10 | 4 | 0 | 0 | 0 | 0 | 1 | 0 |
| Brain Frontal Cortex BA9 | 122 | 8 | 6 | 2 | 0 | 47 | 4 | 20 | 15 | 5 | 4 | 24 | 15 | 43 | 4 | 3 | 2 | 3 | 1 | 0 | 0 | 0 | 0 | 0 | 0 |
| Brain Hippocampus | 90 | 1 | 0 | 0 | 0 | 42 | 7 | 22 | 15 | 11 | 7 | 23 | 13 | 31 | 3 | 1 | 1 | 1 | 0 | 0 | 0 | 0 | 0 | 0 | 0 |
| Brain Hypothalamus | 110 | 4 | 4 | 2 | 0 | 41 | 3 | 18 | 14 | 12 | 6 | 24 | 13 | 24 | 2 | 5 | 4 | 2 | 1 | 0 | 0 | 1 | 0 | 0 | 0 |
| Brain Nucleus accumbens basal ganglia | 130 | 7 | 2 | 1 | 0 | 52 | 5 | 19 | 15 | 11 | 11 | 26 | 15 | 33 | 1 | 2 | 2 | 2 | 1 | 0 | 0 | 0 | 0 | 0 | 0 |
| Brain Putamen basal ganglia | 108 | 5 | 2 | 0 | 0 | 45 | 5 | 16 | 11 | 9 | 9 | 30 | 16 | 26 | 2 | 3 | 3 | 2 | 1 | 0 | 0 | 2 | 0 | 0 | 0 |
| Brain Spinal cord cervical c-1 | 105 | 9 | 3 | 3 | 0 | 33 | 1 | 14 | 10 | 11 | 10 | 22 | 15 | 33 | 0 | 4 | 3 | 5 | 1 | 0 | 0 | 0 | 0 | 0 | 0 |
| Brain Substantia nigra | 85 | 6 | 4 | 0 | 0 | 33 | 3 | 16 | 12 | 5 | 2 | 15 | 10 | 25 | 3 | 2 | 2 | 1 | 0 | 0 | 0 | 0 | 0 | 0 | 0 |
| Breast Mammary Tissue | 132 | 17 | 9 | 2 | 0 | 57 | 11 | 16 | 13 | 8 | 8 | 18 | 14 | 43 | 2 | 1 | 1 | 4 | 0 | 0 | 0 | 0 | 0 | 0 | 0 |
| Cells EBV-transformed lymphocytes | 96 | 8 | 2 | 0 | 0 | 32 | 3 | 22 | 16 | 9 | 7 | 19 | 15 | 41 | 4 | 1 | 1 | 3 | 1 | 1 | 0 | 0 | 0 | 0 | 0 |
| Cells Transformed fibroblasts | 124 | 11 | 7 | 0 | 0 | 54 | 6 | 22 | 18 | 9 | 8 | 25 | 13 | 30 | 3 | 3 | 2 | 5 | 0 | 0 | 0 | 0 | 0 | 0 | 0 |
| Colon Sigmoid | 117 | 5 | 3 | 1 | 0 | 26 | 4 | 21 | 17 | 8 | 8 | 25 | 17 | 45 | 2 | 5 | 3 | 7 | 0 | 0 | 0 | 0 | 0 | 1 | 0 |
| Colon Transverse | 112 | 15 | 7 | 2 | 0 | 50 | 2 | 17 | 11 | 8 | 8 | 24 | 13 | 44 | 2 | 3 | 2 | 5 | 1 | 0 | 0 | 0 | 0 | 0 | 0 |
| Esophagus Gastroesophageal Junction | 118 | 15 | 5 | 1 | 0 | 33 | 0 | 22 | 19 | 10 | 9 | 23 | 16 | 39 | 3 | 1 | 1 | 3 | 0 | 0 | 0 | 0 | 0 | 0 | 0 |
| Esophagus Mucosa | 137 | 9 | 5 | 1 | 0 | 51 | 4 | 28 | 24 | 14 | 8 | 30 | 20 | 43 | 2 | 4 | 3 | 4 | 1 | 1 | 0 | 0 | 0 | 0 | 0 |
| Esophagus Muscularis | 140 | 16 | 11 | 1 | 0 | 39 | 2 | 24 | 19 | 10 | 10 | 30 | 18 | 37 | 2 | 3 | 3 | 6 | 0 | 0 | 0 | 0 | 0 | 1 | 0 |
| Heart Atrial Appendage | 110 | 14 | 7 | 2 | 0 | 41 | 6 | 23 | 19 | 9 | 7 | 28 | 13 | 53 | 1 | 2 | 1 | 2 | 0 | 0 | 0 | 0 | 0 | 0 | 0 |
| Heart Left Ventricle | 110 | 20 | 12 | 0 | 0 | 35 | 4 | 14 | 10 | 8 | 7 | 26 | 14 | 38 | 3 | 1 | 1 | 0 | 0 | 0 | 0 | 0 | 0 | 0 | 0 |
| Liver | 101 | 7 | 4 | 0 | 0 | 50 | 6 | 23 | 17 | 9 | 9 | 20 | 10 | 39 | 6 | 1 | 0 | 3 | 1 | 0 | 0 | 0 | 0 | 0 | 0 |
| Lung | 108 | 9 | 6 | 0 | 0 | 64 | 5 | 18 | 15 | 10 | 6 | 28 | 15 | 39 | 2 | 2 | 1 | 12 | 3 | 0 | 0 | 0 | 0 | 0 | 0 |
| Minor Salivary Gland | 98 | 11 | 6 | 0 | 0 | 38 | 4 | 17 | 13 | 9 | 7 | 26 | 15 | 21 | 3 | 4 | 2 | 1 | 0 | 0 | 0 | 0 | 0 | 0 | 0 |
| Muscle Skeletal | 145 | 16 | 9 | 2 | 0 | 46 | 6 | 23 | 18 | 7 | 7 | 33 | 19 | 55 | 4 | 2 | 2 | 3 | 0 | 0 | 0 | 0 | 0 | 0 | 0 |
| Nerve Tibial | 170 | 11 | 4 | 1 | 0 | 40 | 1 | 27 | 25 | 9 | 9 | 27 | 16 | 34 | 4 | 3 | 2 | 7 | 1 | 0 | 0 | 0 | 0 | 0 | 0 |
| Ovary | 102 | 14 | 3 | 0 | 0 | 44 | 4 | 21 | 15 | 11 | 10 | 31 | 15 | 26 | 2 | 1 | 1 | 2 | 0 | 0 | 0 | 0 | 0 | 0 | 0 |
| Pancreas | 131 | 11 | 4 | 1 | 0 | 50 | 3 | 24 | 21 | 6 | 4 | 22 | 11 | 28 | 0 | 2 | 2 | 7 | 2 | 0 | 0 | 0 | 0 | 0 | 0 |
| Pituitary | 124 | 11 | 3 | 1 | 0 | 45 | 5 | 14 | 11 | 10 | 7 | 25 | 17 | 34 | 3 | 1 | 1 | 7 | 0 | 0 | 0 | 0 | 0 | 0 | 0 |
| Prostate | 122 | 10 | 4 | 0 | 0 | 28 | 4 | 15 | 11 | 7 | 7 | 22 | 14 | 23 | 0 | 0 | 0 | 6 | 0 | 0 | 0 | 0 | 0 | 0 | 0 |
| Skin Not Sun Exposed Suprapubic | 138 | 10 | 7 | 1 | 0 | 43 | 4 | 29 | 23 | 13 | 12 | 30 | 16 | 37 | 2 | 4 | 4 | 5 | 0 | 0 | 0 | 0 | 0 | 0 | 0 |
| Skin Sun Exposed Lower leg | 142 | 8 | 4 | 1 | 0 | 47 | 3 | 30 | 21 | 15 | 13 | 27 | 17 | 44 | 5 | 4 | 3 | 8 | 2 | 0 | 0 | 0 | 0 | 2 | 0 |
| Small Intestine Terminal Ileum | 97 | 14 | 8 | 1 | 0 | 35 | 1 | 14 | 12 | 7 | 6 | 31 | 18 | 39 | 3 | 3 | 2 | 7 | 1 | 0 | 0 | 1 | 0 | 0 | 0 |
| Spleen | 127 | 10 | 4 | 1 | 0 | 42 | 4 | 19 | 16 | 11 | 9 | 24 | 16 | 40 | 4 | 2 | 2 | 6 | 2 | 0 | 0 | 0 | 0 | 0 | 0 |
| Stomach | 107 | 14 | 8 | 1 | 0 | 29 | 2 | 18 | 17 | 8 | 7 | 20 | 14 | 34 | 3 | 1 | 1 | 4 | 1 | 0 | 0 | 0 | 0 | 0 | 0 |
| Testis | 212 | 8 | 7 | 2 | 0 | 48 | 2 | 18 | 14 | 21 | 15 | 38 | 23 | 45 | 3 | 2 | 2 | 3 | 0 | 0 | 0 | 0 | 0 | 0 | 0 |
| Thyroid | 156 | 16 | 4 | 1 | 0 | 46 | 1 | 21 | 17 | 11 | 10 | 33 | 18 | 74 | 4 | 5 | 3 | 10 | 0 | 0 | 0 | 0 | 0 | 0 | 0 |
| Uterus | 111 | 5 | 3 | 0 | 0 | 31 | 1 | 15 | 9 | 3 | 2 | 27 | 19 | 19 | 1 | 3 | 3 | 4 | 0 | 0 | 0 | 0 | 0 | 0 | 0 |
| Vagina | 103 | 6 | 4 | 2 | 0 | 31 | 4 | 20 | 18 | 8 | 7 | 22 | 14 | 28 | 4 | 2 | 2 | 1 | 0 | 0 | 0 | 0 | 0 | 0 | 0 |
| Whole Blood | 124 | 8 | 3 | 1 | 0 | 58 | 1 | 24 | 14 | 7 | 7 | 19 | 12 | 30 | 2 | 7 | 4 | 5 | 1 | 0 | 0 | 0 | 0 | 0 | 0 |

Note: ADHD: Attention deficit/hyperactivity disorder; ALS: Amyotrophic lateral sclerosis; AD: Alzheimer’s dementia; AN: Anorexia nervosa; ASD: Autism spectrum disorders; AUD: Alcohol use disorders; BIP: Bipolar disorder; CUD: Cannabis use disorder; MDD: Major depressive disorder; OCD: Obsessive compulsive disorder; PTSD: Posttraumatic stress disorder; SCZ: Schizophrenia; TS: Tourette’s syndrome

## Supplementary Table 19. List of instrumental variables used in our Mendelian randomization analyses.

| SNP | A1 | A2 | BETA | SE | P | N | Trait | F statistic | R^2^ |
| --- | --- | --- | --- | --- | --- | --- | --- | --- | --- |
| rs4575098 | A | G | 0.016412 | 0.002577 | 1.9E-10 | 427808.6 | AD | 40.57 | 9.48E-05 |
| rs679515 | T | C | 0.025418 | 0.002863 | 6.83E-19 | 429226.7 | AD | 78.81 | 0.000184 |
| rs4663105 | C | A | 0.031095 | 0.00222 | 1.45E-44 | 418700 | AD | 196.15 | 0.000468 |
| rs10933431 | G | C | -0.01544 | 0.002509 | 7.62E-10 | 429961.1 | AD | 37.85 | 8.8E-05 |
| rs6448453 | A | G | 0.014705 | 0.002451 | 1.98E-09 | 429961.1 | AD | 36 | 8.37E-05 |
| rs6931277 | T | A | -0.01934 | 0.002969 | 7.35E-11 | 429754 | AD | 42.42 | 9.87E-05 |
| rs9381563 | C | T | 0.014451 | 0.002272 | 1.99E-10 | 422732.9 | AD | 40.47 | 9.57E-05 |
| rs1859788 | A | G | -0.0184 | 0.002313 | 1.8E-15 | 426509.1 | AD | 63.27 | 0.000148 |
| rs7810606 | T | C | -0.01452 | 0.002183 | 2.89E-11 | 420152.6 | AD | 44.25 | 0.000105 |
| rs755951 | C | A | 0.015005 | 0.00221 | 1.13E-11 | 422103.7 | AD | 46.08 | 0.000109 |
| rs4236673 | A | G | -0.02016 | 0.002229 | 1.48E-19 | 428128.5 | AD | 81.83 | 0.000191 |
| rs11257238 | C | T | 0.012943 | 0.002261 | 1.04E-08 | 423848.7 | AD | 32.76 | 7.73E-05 |
| rs2081545 | A | C | -0.01787 | 0.00223 | 1.11E-15 | 425881.4 | AD | 64.23 | 0.000151 |
| rs867611 | G | A | -0.02043 | 0.002324 | 1.48E-18 | 427263.6 | AD | 77.28 | 0.000181 |
| rs11218343 | C | T | -0.03593 | 0.005255 | 8.12E-12 | 429714.3 | AD | 46.74 | 0.000109 |
| rs12590654 | A | G | -0.01483 | 0.002308 | 1.32E-10 | 420451.9 | AD | 41.27 | 9.82E-05 |
| rs442495 | C | T | -0.01372 | 0.002258 | 1.22E-09 | 428715.4 | AD | 36.94 | 8.61E-05 |
| rs59735493 | A | G | -0.01299 | 0.00236 | 3.73E-08 | 428873.9 | AD | 30.29 | 7.06E-05 |
| rs113260531 | A | G | 0.019986 | 0.003251 | 7.91E-10 | 429194 | AD | 37.78 | 8.8E-05 |
| rs28394864 | A | G | 0.012302 | 0.00218 | 1.68E-08 | 424326.4 | AD | 31.83 | 7.5E-05 |
| rs111278892 | G | C | 0.019912 | 0.00305 | 6.67E-11 | 428210 | AD | 42.61 | 9.95E-05 |
| rs846881 | C | A | -0.01737 | 0.002685 | 9.89E-11 | 422334.6 | AD | 41.84 | 9.91E-05 |
| rs28399657 | G | A | -0.05464 | 0.006577 | 9.82E-17 | 379267.5 | AD | 69 | 0.000182 |
| rs148601586 | G | C | 0.185399 | 0.012069 | 2.94E-53 | 379166.6 | AD | 235.99 | 0.000622 |
| rs41289512 | G | C | 0.206303 | 0.005785 | 1E-200 | 429961.1 | AD | 1271.88 | 0.002949 |
| rs41290120 | A | G | -0.09905 | 0.005778 | 7.14E-66 | 428756 | AD | 293.87 | 0.000685 |
| rs118170342 | C | T | 0.147537 | 0.005698 | 7.9E-148 | 426903.9 | AD | 670.46 | 0.001568 |
| rs204473 | A | G | -0.04167 | 0.006996 | 2.58E-09 | 429524 | AD | 35.48 | 8.26E-05 |
| rs346757 | C | G | 0.034313 | 0.003504 | 1.2E-22 | 426431.2 | AD | 95.91 | 0.000225 |
| rs6014724 | G | A | -0.02289 | 0.003688 | 5.38E-10 | 427344.8 | AD | 38.53 | 9.02E-05 |
| rs17531412 | A | G | 0.105396 | 0.0148 | 1.07E-12 | 53293 | ADHD | 50.71 | 0.000951 |
| rs1222063 | A | G | 0.096201 | 0.0174 | 3.07E-08 | 53293 | ADHD | 30.57 | 0.000573 |
| rs4858241 | T | G | 0.082197 | 0.0143 | 8.17E-09 | 53293 | ADHD | 33.04 | 0.00062 |
| rs28411770 | T | C | 0.086104 | 0.0151 | 1.15E-08 | 53293 | ADHD | 32.52 | 0.00061 |
| rs4916723 | A | C | -0.0778 | 0.0138 | 1.81E-08 | 53293 | ADHD | 31.78 | 0.000596 |
| rs10262192 | A | G | 0.074096 | 0.0135 | 3.66E-08 | 53293 | ADHD | 30.12 | 0.000565 |
| rs74760947 | A | G | -0.17961 | 0.0317 | 1.39E-08 | 53293 | ADHD | 32.1 | 0.000602 |
| rs11591402 | A | T | -0.0924 | 0.0164 | 1.76E-08 | 53293 | ADHD | 31.74 | 0.000595 |
| rs1427829 | A | G | 0.082197 | 0.0136 | 1.35E-09 | 53293 | ADHD | 36.53 | 0.000685 |
| rs8039398 | T | C | -0.08 | 0.0135 | 2.99E-09 | 53293 | ADHD | 35.11 | 0.000658 |
| rs212178 | A | G | -0.1171 | 0.0205 | 1.2E-08 | 53293 | ADHD | 32.63 | 0.000612 |
| rs3849943 | T | C | -0.1808 | 0.0202 | 3.99E-19 | 36052 | ALS | 80.11 | 0.002217 |
| rs35714695 | A | G | -0.1344 | 0.0236 | 1.29E-08 | 36052 | ALS | 32.43 | 0.000899 |
| rs78549703 | A | G | 0.1095 | 0.0193 | 1.31E-08 | 36052 | ALS | 32.19 | 0.000892 |
| rs75087725 | A | C | 0.4787 | 0.0738 | 8.65E-11 | 35585 | ALS | 42.07 | 0.001181 |
| rs2821359 | T | C | -0.082 | 0.0161 | 3.76E-07 | 23160 | AN | 25.94 | 0.001119 |
| rs13100344 | T | A | 0.074597 | 0.0136 | 4.21E-08 | 23160 | AN | 30.09 | 0.001297 |
| rs28380 | G | C | -0.07461 | 0.0143 | 1.8E-07 | 23160 | AN | 27.22 | 0.001174 |
| rs2131959 | G | C | 0.0771 | 0.015 | 3.04E-07 | 22992 | AN | 26.42 | 0.001148 |
| rs6789500 | T | C | 0.0908 | 0.0176 | 2.55E-07 | 20159 | AN | 26.62 | 0.001319 |
| rs8070063 | G | T | -0.07529 | 0.0141 | 9.73E-08 | 23160 | AN | 28.52 | 0.00123 |
| rs725861 | A | G | 0.088102 | 0.0175 | 4.86E-07 | 23160 | AN | 25.35 | 0.001093 |
| rs2008387 | G | A | -0.0815 | 0.0145 | 1.73E-08 | 23160 | AN | 31.59 | 0.001362 |
| rs9821797 | T | A | -0.1566 | 0.0201 | 6.99E-15 | 23160 | AN | 60.7 | 0.002614 |
| rs370838138 | G | C | 0.075302 | 0.0136 | 3.17E-08 | 23160 | AN | 30.66 | 0.001322 |
| rs6092932 | G | A | 0.095301 | 0.0187 | 3.5E-07 | 23160 | AN | 25.97 | 0.00112 |
| rs9874207 | T | C | -0.0813 | 0.0145 | 2.05E-08 | 23160 | AN | 31.43 | 0.001356 |
| rs6589488 | A | T | 0.127302 | 0.0195 | 6.31E-11 | 23160 | AN | 42.62 | 0.001837 |
| rs750350 | G | T | -0.1109 | 0.0208 | 9.42E-08 | 23160 | AN | 28.43 | 0.001226 |
| rs1539725 | T | C | -0.0734 | 0.0139 | 1.26E-07 | 23160 | AN | 27.88 | 0.001203 |
| rs11615526 | G | A | 0.090298 | 0.0172 | 1.52E-07 | 23160 | AN | 27.56 | 0.001189 |
| rs2287348 | C | T | -0.10441 | 0.0179 | 5.62E-09 | 23160 | AN | 34.02 | 0.001467 |
| rs10747478 | T | G | 0.075701 | 0.0137 | 3.13E-08 | 23160 | AN | 30.53 | 0.001317 |
| rs13125932 | C | T | -0.0725 | 0.0134 | 5.85E-08 | 23160 | AN | 29.27 | 0.001262 |
| rs910805 | A | G | -0.0957 | 0.016 | 2.04E-09 | 46351 | ASD | 35.77 | 0.000771 |
| rs111931861 | A | G | -0.2169 | 0.0409 | 1.12E-07 | 46351 | ASD | 28.12 | 0.000606 |
| rs2224274 | T | C | 0.070999 | 0.0138 | 2.86E-07 | 46351 | ASD | 26.47 | 0.000571 |
| rs325485 | A | G | 0.072804 | 0.0143 | 3.25E-07 | 46351 | ASD | 25.92 | 0.000559 |
| rs112635299 | T | G | 0.220997 | 0.0432 | 3.04E-07 | 46351 | ASD | 26.17 | 0.000564 |
| rs10099100 | C | G | 0.084304 | 0.0147 | 1.07E-08 | 46351 | ASD | 32.89 | 0.000709 |
| rs45595836 | T | C | 0.138996 | 0.0272 | 3.13E-07 | 46351 | ASD | 26.11 | 0.000563 |
| rs2391769 | A | G | -0.0769 | 0.0145 | 1.14E-07 | 46351 | ASD | 28.13 | 0.000607 |
| rs6701243 | A | C | 0.073501 | 0.0144 | 3.07E-07 | 46351 | ASD | 26.05 | 0.000562 |
| rs1452075 | T | C | 0.080704 | 0.0155 | 2.07E-07 | 46351 | ASD | 27.11 | 0.000585 |
| rs11940694 | G | A | 0.011217 | 0.001234 | 9.84E-20 | 117878 | AUD | 82.67 | 0.000701 |
| rs1260326 | C | T | 0.007777 | 0.001224 | 2.11E-10 | 121604 | AUD | 40.36 | 0.000332 |
| rs13135688 | C | G | -0.01278 | 0.002215 | 7.98E-09 | 121355 | AUD | 33.28 | 0.000274 |
| rs144198753 | T | C | -0.06433 | 0.005715 | 2.22E-29 | 120486 | AUD | 126.71 | 0.001051 |
| rs147431626 | A | G | -0.00876 | 0.001432 | 9.55E-10 | 120054 | AUD | 37.42 | 0.000312 |
| rs4953148 | T | A | 0.00729 | 0.001313 | 2.81E-08 | 119522 | AUD | 30.84 | 0.000258 |
| rs62325470 | T | C | -0.05584 | 0.006536 | 1.32E-17 | 120735 | AUD | 72.99 | 0.000604 |
| rs7934481 | T | C | 0.007858 | 0.00131 | 2.01E-09 | 121470 | AUD | 35.97 | 0.000296 |
| rs9607805 | T | C | 0.007436 | 0.00135 | 3.58E-08 | 120262 | AUD | 30.37 | 0.000252 |
| rs10455979 | C | G | -0.075 | 0.0137 | 4.6E-08 | 23291 | BIP | 29.97 | 0.001285 |
| rs10744560 | T | C | 0.083201 | 0.014 | 2.92E-09 | 23291 | BIP | 35.32 | 0.001514 |
| rs111444407 | T | C | 0.1166 | 0.0184 | 2.4E-10 | 23291 | BIP | 40.16 | 0.001721 |
| rs11724116 | T | C | -0.10409 | 0.0188 | 3.27E-08 | 23291 | BIP | 30.66 | 0.001315 |
| rs13231398 | C | G | -0.1207 | 0.0219 | 3.36E-08 | 23291 | BIP | 30.38 | 0.001303 |
| rs138321 | A | G | 0.079301 | 0.0135 | 4.69E-09 | 23291 | BIP | 34.51 | 0.001479 |
| rs17150022 | T | C | -0.1132 | 0.0204 | 2.7E-08 | 23291 | BIP | 30.79 | 0.00132 |
| rs174592 | A | G | -0.0774 | 0.0141 | 3.66E-08 | 23291 | BIP | 30.13 | 0.001292 |
| rs2302417 | A | T | -0.0793 | 0.0136 | 4.93E-09 | 23291 | BIP | 34 | 0.001458 |
| rs2314398 | C | G | 0.084102 | 0.0144 | 5.92E-09 | 23291 | BIP | 34.11 | 0.001463 |
| rs329319 | A | G | 0.078802 | 0.0139 | 1.54E-08 | 23291 | BIP | 32.14 | 0.001378 |
| rs55648125 | A | G | -0.1171 | 0.0215 | 4.92E-08 | 23291 | BIP | 29.66 | 0.001272 |
| rs71395455 | A | G | 0.082096 | 0.0146 | 1.93E-08 | 23291 | BIP | 31.62 | 0.001356 |
| rs73496688 | A | T | 0.108702 | 0.019 | 1.05E-08 | 23291 | BIP | 32.73 | 0.001403 |
| rs884301 | T | C | 0.080298 | 0.0138 | 5.8E-09 | 23291 | BIP | 33.86 | 0.001452 |
| rs9834970 | T | C | -0.101 | 0.0134 | 5.53E-14 | 23291 | BIP | 56.81 | 0.002434 |
| rs1392816 | T | C | -0.0998 | 0.0184 | 6.14E-08 | 351174 | CUD | 29.42 | 8.38E-05 |
| rs719504 | A | G | 0.1025 | 0.0192 | 9E-08 | 350403 | CUD | 28.5 | 8.13E-05 |
| rs11715758 | A | G | -0.0935 | 0.0175 | 8.91E-08 | 355885 | CUD | 28.55 | 8.02E-05 |
| rs72818514 | T | C | -0.1828 | 0.0342 | 9.33E-08 | 355548 | CUD | 28.57 | 8.03E-05 |
| rs553920 | T | C | 0.104 | 0.0198 | 1.6E-07 | 353969 | CUD | 27.59 | 7.79E-05 |
| rs7783012 | A | G | 0.101 | 0.0168 | 1.84E-09 | 357019 | CUD | 36.14 | 0.000101 |
| rs11783093 | T | C | -0.145 | 0.0244 | 2.68E-09 | 355028 | CUD | 35.31 | 9.95E-05 |
| rs719012 | T | C | 0.0961 | 0.0187 | 2.87E-07 | 356928 | CUD | 26.41 | 7.4E-05 |
| rs1509513 | A | G | 0.0857 | 0.0168 | 3.18E-07 | 356900 | CUD | 26.02 | 7.29E-05 |
| rs9787909 | A | C | 0.1137 | 0.0225 | 4.52E-07 | 354449 | CUD | 25.54 | 7.2E-05 |
| rs17271123 | T | G | 0.1284 | 0.0252 | 3.54E-07 | 291017 | CUD | 25.96 | 8.92E-05 |
| rs17514242 | C | G | -0.088 | 0.0171 | 2.56E-07 | 357037 | CUD | 26.48 | 7.42E-05 |
| rs11130222 | A | T | 0.026 | 0.003 | 4.58E-25 | 293723 | EA | 75.11 | 0.000256 |
| rs7029201 | A | G | 0.025 | 0.003 | 6.14E-23 | 293723 | EA | 69.44 | 0.000236 |
| rs28792186 | T | C | -0.025 | 0.003 | 6.41E-22 | 293723 | EA | 69.44 | 0.000236 |
| rs12987662 | A | C | 0.022 | 0.003 | 3.25E-18 | 293723 | EA | 53.78 | 0.000183 |
| rs9527702 | A | G | 0.023 | 0.003 | 4.99E-17 | 293723 | EA | 58.78 | 0.0002 |
| rs34305371 | A | G | 0.036 | 0.004 | 2.34E-16 | 293723 | EA | 81 | 0.000276 |
| rs9739070 | A | G | 0.024 | 0.003 | 3.95E-16 | 293723 | EA | 64 | 0.000218 |
| rs6882046 | A | G | -0.021 | 0.003 | 7.92E-14 | 293723 | EA | 49 | 0.000167 |
| rs17824247 | T | C | -0.018 | 0.003 | 5.29E-13 | 293723 | EA | 36 | 0.000123 |
| rs61160187 | A | G | -0.018 | 0.003 | 5.93E-13 | 293723 | EA | 36 | 0.000123 |
| rs11191193 | A | G | 0.019 | 0.003 | 6.97E-13 | 293723 | EA | 40.11 | 0.000137 |
| rs11588857 | A | G | 0.022 | 0.003 | 1.31E-12 | 293723 | EA | 53.78 | 0.000183 |
| rs2456973 | A | C | -0.018 | 0.003 | 1.58E-12 | 293723 | EA | 36 | 0.000123 |
| rs9792504 | A | G | -0.018 | 0.003 | 3.25E-12 | 293723 | EA | 36 | 0.000123 |
| rs4863692 | T | G | 0.018 | 0.003 | 3.8E-12 | 293723 | EA | 36 | 0.000123 |
| rs12969294 | A | G | -0.018 | 0.003 | 1.11E-11 | 293723 | EA | 36 | 0.000123 |
| rs6839705 | A | C | 0.017 | 0.003 | 1.72E-11 | 293723 | EA | 32.11 | 0.000109 |
| rs7964899 | A | G | 0.017 | 0.002 | 1.99E-11 | 293723 | EA | 72.25 | 0.000246 |
| rs7599488 | T | C | -0.017 | 0.002 | 2.05E-11 | 293723 | EA | 72.25 | 0.000246 |
| rs12410444 | A | G | -0.018 | 0.003 | 2.14E-11 | 293723 | EA | 36 | 0.000123 |
| rs1106761 | A | G | -0.017 | 0.003 | 4.08E-11 | 293723 | EA | 32.11 | 0.000109 |
| rs58694847 | C | G | -0.018 | 0.003 | 7.41E-11 | 293723 | EA | 36 | 0.000123 |
| rs1008078 | T | C | -0.016 | 0.003 | 7.88E-11 | 293723 | EA | 28.44 | 9.68E-05 |
| rs34344888 | A | G | -0.016 | 0.003 | 1.11E-10 | 293723 | EA | 28.44 | 9.68E-05 |
| rs1378214 | T | C | -0.016 | 0.003 | 1.2E-10 | 293723 | EA | 28.44 | 9.68E-05 |
| rs16845580 | T | C | 0.016 | 0.003 | 2.07E-10 | 293723 | EA | 28.44 | 9.68E-05 |
| rs71537331 | T | C | -0.017 | 0.003 | 2.38E-10 | 293723 | EA | 32.11 | 0.000109 |
| rs12900061 | A | G | 0.021 | 0.003 | 2.46E-10 | 293723 | EA | 49 | 0.000167 |
| rs35771425 | T | C | 0.019 | 0.003 | 2.62E-10 | 293723 | EA | 40.11 | 0.000137 |
| rs7776010 | T | C | -0.021 | 0.003 | 3.03E-10 | 293723 | EA | 49 | 0.000167 |
| rs12514965 | T | C | 0.018 | 0.003 | 5.12E-10 | 293723 | EA | 36 | 0.000123 |
| rs4741351 | A | G | -0.017 | 0.003 | 1.13E-09 | 293723 | EA | 32.11 | 0.000109 |
| rs320700 | A | G | 0.016 | 0.003 | 1.5E-09 | 293723 | EA | 28.44 | 9.68E-05 |
| rs28420834 | A | G | -0.015 | 0.003 | 1.55E-09 | 293723 | EA | 25 | 8.51E-05 |
| rs62263923 | A | G | -0.016 | 0.003 | 1.63E-09 | 293723 | EA | 28.44 | 9.68E-05 |
| rs9616906 | A | G | 0.015 | 0.003 | 1.73E-09 | 293723 | EA | 25 | 8.51E-05 |
| rs9556958 | T | C | -0.015 | 0.002 | 1.89E-09 | 293723 | EA | 56.25 | 0.000191 |
| rs4800490 | A | C | -0.015 | 0.002 | 2.13E-09 | 293723 | EA | 56.25 | 0.000191 |
| rs4240470 | C | G | 0.016 | 0.003 | 2.63E-09 | 293723 | EA | 28.44 | 9.68E-05 |
| rs8049439 | T | C | 0.015 | 0.003 | 2.69E-09 | 293723 | EA | 25 | 8.51E-05 |
| rs1396967 | T | C | -0.015 | 0.003 | 2.9E-09 | 293723 | EA | 25 | 8.51E-05 |
| rs10483349 | A | G | -0.019 | 0.003 | 3.02E-09 | 293723 | EA | 40.11 | 0.000137 |
| rs141979783 | T | C | 0.037 | 0.006 | 6.67E-09 | 293723 | EA | 38.03 | 0.000129 |
| rs12534506 | A | T | -0.015 | 0.003 | 7.95E-09 | 293723 | EA | 25 | 8.51E-05 |
| rs13421974 | T | C | 0.014 | 0.002 | 8.96E-09 | 293723 | EA | 49 | 0.000167 |
| rs1424580 | T | C | 0.018 | 0.003 | 9.58E-09 | 293723 | EA | 36 | 0.000123 |
| rs4244613 | A | G | -0.014 | 0.003 | 9.94E-09 | 293723 | EA | 21.78 | 7.41E-05 |
| rs3890065 | C | G | 0.015 | 0.003 | 1.03E-08 | 293723 | EA | 25 | 8.51E-05 |
| rs523934 | A | G | 0.015 | 0.003 | 1.09E-08 | 293723 | EA | 25 | 8.51E-05 |
| rs62100767 | A | G | 0.014 | 0.003 | 1.11E-08 | 293723 | EA | 21.78 | 7.41E-05 |
| rs11222416 | T | C | -0.015 | 0.003 | 1.17E-08 | 293723 | EA | 25 | 8.51E-05 |
| rs12962421 | A | G | -0.014 | 0.002 | 1.5E-08 | 293723 | EA | 49 | 0.000167 |
| rs766406 | T | G | 0.014 | 0.003 | 1.89E-08 | 293723 | EA | 21.78 | 7.41E-05 |
| rs4478846 | T | C | 0.018 | 0.003 | 1.93E-08 | 293723 | EA | 36 | 0.000123 |
| rs7033137 | C | G | 0.016 | 0.003 | 2.14E-08 | 293723 | EA | 28.44 | 9.68E-05 |
| rs538628 | C | G | -0.018 | 0.003 | 2.16E-08 | 293723 | EA | 36 | 0.000123 |
| rs13010288 | T | G | 0.02 | 0.004 | 2.21E-08 | 293723 | EA | 25 | 8.51E-05 |
| rs4493682 | C | G | 0.019 | 0.003 | 2.27E-08 | 293723 | EA | 40.11 | 0.000137 |
| rs11726992 | T | C | 0.014 | 0.003 | 2.58E-08 | 293723 | EA | 21.78 | 7.41E-05 |
| rs4468571 | A | G | -0.014 | 0.003 | 2.59E-08 | 293723 | EA | 21.78 | 7.41E-05 |
| rs10006235 | T | C | -0.015 | 0.003 | 2.63E-08 | 293723 | EA | 25 | 8.51E-05 |
| rs4974424 | A | G | -0.019 | 0.003 | 2.64E-08 | 293723 | EA | 40.11 | 0.000137 |
| rs56236451 | A | G | 0.018 | 0.003 | 2.99E-08 | 293723 | EA | 36 | 0.000123 |
| rs1382358 | T | C | 0.021 | 0.004 | 3.17E-08 | 293723 | EA | 27.56 | 9.38E-05 |
| rs12761761 | T | C | 0.017 | 0.003 | 3.19E-08 | 293723 | EA | 32.11 | 0.000109 |
| rs3095075 | A | G | -0.014 | 0.002 | 3.53E-08 | 293723 | EA | 49 | 0.000167 |
| rs7146434 | A | G | -0.014 | 0.002 | 3.74E-08 | 293723 | EA | 49 | 0.000167 |
| rs7948975 | T | C | 0.014 | 0.003 | 3.83E-08 | 293723 | EA | 21.78 | 7.41E-05 |
| rs10772644 | C | G | 0.021 | 0.004 | 4.11E-08 | 293723 | EA | 27.56 | 9.38E-05 |
| rs111321694 | T | C | -0.018 | 0.003 | 4.23E-08 | 293723 | EA | 36 | 0.000123 |
| rs17425572 | A | G | 0.014 | 0.002 | 4.58E-08 | 293723 | EA | 49 | 0.000167 |
| rs1035578 | A | G | -0.013 | 0.002 | 4.71E-08 | 293723 | EA | 42.25 | 0.000144 |
| rs6905391 | A | G | -0.074 | 0.0112 | 3.47E-11 | 69115.85 | MDD | 43.66 | 0.000631 |
| rs12129573 | A | C | 0.047799 | 0.0082 | 5.45E-09 | 69115.85 | MDD | 33.98 | 0.000491 |
| rs7531118 | T | C | -0.045 | 0.008 | 2.15E-08 | 69115.85 | MDD | 31.64 | 0.000458 |
| rs76025409 | C | G | 0.056702 | 0.0085 | 2.32E-11 | 67392.14 | MDD | 44.5 | 0.00066 |
| rs1950829 | A | G | 0.045403 | 0.0079 | 8.15E-09 | 69115.85 | MDD | 33.03 | 0.000478 |
| rs12568997 | A | G | -0.2933 | 0.058 | 4.23E-07 | 9725 | OCD | 25.57 | 0.002623 |
| rs116347760 | A | T | 0.629701 | 0.1335 | 2.39E-06 | 9725 | OCD | 22.25 | 0.002283 |
| rs56343802 | A | T | -0.1698 | 0.0368 | 3.97E-06 | 9725 | OCD | 21.29 | 0.002185 |
| rs13141765 | T | C | -0.2663 | 0.0558 | 1.86E-06 | 9725 | OCD | 22.78 | 0.002337 |
| rs12504244 | C | G | -0.1689 | 0.0352 | 1.62E-06 | 9725 | OCD | 23.02 | 0.002362 |
| rs1030757 | A | C | -0.1653 | 0.0339 | 1.09E-06 | 9725 | OCD | 23.78 | 0.002439 |
| rs55687617 | A | G | -0.2702 | 0.0575 | 2.67E-06 | 9725 | OCD | 22.08 | 0.002266 |
| rs4733767 | A | G | 0.193501 | 0.039 | 7.1E-07 | 9725 | OCD | 24.62 | 0.002525 |
| rs72781967 | T | C | -0.1666 | 0.0353 | 2.43E-06 | 9725 | OCD | 22.27 | 0.002286 |
| rs639560 | T | C | -0.4158 | 0.0879 | 2.23E-06 | 9725 | OCD | 22.38 | 0.002296 |
| rs72783425 | A | C | 0.339703 | 0.0733 | 3.53E-06 | 9725 | OCD | 21.48 | 0.002204 |
| rs9952159 | T | C | 0.182297 | 0.0396 | 4.21E-06 | 9725 | OCD | 21.19 | 0.002175 |
| rs117310268 | T | C | 0.448697 | 0.0965 | 3.31E-06 | 9725 | OCD | 21.62 | 0.002219 |
| rs77885126 | T | C | -0.6025 | 0.1312 | 4.38E-06 | 9725 | OCD | 21.09 | 0.002164 |
| rs909701 | C | G | -0.1554 | 0.0337 | 4.05E-06 | 9725 | OCD | 21.26 | 0.002182 |
| rs34517852 | A | T | 0.1095 | 0.0185 | 3.16E-09 | 30273.8 | PTSD | 35.03 | 0.001156 |
| rs57753395 | A | G | 0.3764 | 0.0705 | 9.13E-08 | 66052.2 | PTSD | 28.51 | 0.000431 |
| rs763753 | A | G | -0.114 | 0.0221 | 2.43E-07 | 70237.5 | PTSD | 26.61 | 0.000379 |
| rs17108326 | A | G | 0.105998 | 0.0209 | 3.91E-07 | 70237.5 | PTSD | 25.72 | 0.000366 |
| rs36127550 | T | G | -0.1027 | 0.0204 | 4.63E-07 | 69136.6 | PTSD | 25.34 | 0.000366 |
| rs2319280 | A | C | -0.0905 | 0.0148 | 9.61E-10 | 36033.86 | SCZ | 37.4 | 0.001037 |
| rs12093576 | T | C | 0.063397 | 0.0112 | 1.31E-08 | 36033.86 | SCZ | 32.04 | 0.000888 |
| rs533123 | A | G | 0.077998 | 0.0142 | 3.64E-08 | 36033.86 | SCZ | 30.17 | 0.000837 |
| rs6694545 | A | G | 0.0787 | 0.0127 | 6.06E-10 | 36033.86 | SCZ | 38.4 | 0.001065 |
| rs11210892 | A | G | -0.0674 | 0.0115 | 4.13E-09 | 36033.86 | SCZ | 34.35 | 0.000952 |
| rs10890030 | T | C | -0.066 | 0.0109 | 1.22E-09 | 36033.86 | SCZ | 36.66 | 0.001017 |
| rs301798 | A | G | -0.0647 | 0.0115 | 2.04E-08 | 36033.86 | SCZ | 31.65 | 0.000878 |
| rs2802535 | T | C | 0.117401 | 0.0138 | 1.61E-17 | 36033.86 | SCZ | 72.37 | 0.002005 |
| rs16825349 | A | G | -0.0797 | 0.0142 | 1.84E-08 | 36033.86 | SCZ | 31.5 | 0.000874 |
| rs4340536 | C | G | -0.0599 | 0.0109 | 3.57E-08 | 36033.86 | SCZ | 30.2 | 0.000837 |
| rs10196799 | A | T | 0.074096 | 0.0109 | 1.12E-11 | 36033.86 | SCZ | 46.21 | 0.001281 |
| rs55775495 | T | C | -0.0682 | 0.0114 | 1.91E-09 | 36033.86 | SCZ | 35.79 | 0.000992 |
| rs2949006 | T | G | 0.104 | 0.0137 | 3.45E-14 | 36033.86 | SCZ | 57.63 | 0.001597 |
| rs12712510 | T | C | 0.063097 | 0.0113 | 2.38E-08 | 34085.99 | SCZ | 31.18 | 0.000914 |
| rs4144795 | C | G | 0.0787 | 0.0113 | 3.12E-12 | 36033.86 | SCZ | 48.51 | 0.001344 |
| rs12474906 | A | C | 0.082004 | 0.0142 | 7.98E-09 | 34085.99 | SCZ | 33.35 | 0.000977 |
| rs11682175 | T | C | -0.0739 | 0.0112 | 4.61E-11 | 34085.99 | SCZ | 43.54 | 0.001276 |
| rs12163529 | A | G | -0.0705 | 0.011 | 1.59E-10 | 36033.86 | SCZ | 41.07 | 0.001139 |
| rs28886334 | A | G | 0.060803 | 0.0111 | 4.8E-08 | 36033.86 | SCZ | 30.01 | 0.000832 |
| rs13071962 | A | G | -0.08141 | 0.0135 | 1.82E-09 | 36033.86 | SCZ | 36.36 | 0.001008 |
| rs17194490 | T | G | 0.099203 | 0.0147 | 1.69E-11 | 36033.86 | SCZ | 45.54 | 0.001262 |
| rs6550435 | T | G | -0.077 | 0.0113 | 8.06E-12 | 36033.86 | SCZ | 46.43 | 0.001287 |
| rs4481150 | T | C | 0.069097 | 0.0109 | 2.29E-10 | 36033.86 | SCZ | 40.18 | 0.001114 |
| rs832187 | T | C | -0.069 | 0.0112 | 7.33E-10 | 36033.86 | SCZ | 37.95 | 0.001052 |
| rs62244881 | T | C | 0.092397 | 0.0162 | 1.29E-08 | 36033.86 | SCZ | 32.53 | 0.000902 |
| rs13107325 | T | C | 0.153896 | 0.0212 | 3.85E-13 | 36033.86 | SCZ | 52.7 | 0.00146 |
| rs7683893 | T | C | -0.0604 | 0.0109 | 3.4E-08 | 36033.86 | SCZ | 30.7 | 0.000851 |
| rs215412 | A | G | 0.067696 | 0.0115 | 3.56E-09 | 36033.86 | SCZ | 34.65 | 0.000961 |
| rs3849046 | T | C | 0.062101 | 0.0109 | 1.3E-08 | 36033.86 | SCZ | 32.46 | 0.0009 |
| rs3112532 | A | G | -0.0689 | 0.0114 | 1.57E-09 | 36033.86 | SCZ | 36.53 | 0.001013 |
| rs11740474 | A | T | -0.0625 | 0.011 | 1.5E-08 | 36033.86 | SCZ | 32.29 | 0.000895 |
| rs4391122 | A | G | -0.0785 | 0.0109 | 5.7E-13 | 36033.86 | SCZ | 51.87 | 0.001437 |
| rs301714 | C | G | 0.149798 | 0.0258 | 6.59E-09 | 36033.86 | SCZ | 33.71 | 0.000935 |
| rs1233578 | A | G | 0.188701 | 0.0161 | 1.48E-31 | 36033.86 | SCZ | 137.37 | 0.003798 |
| rs9270565 | T | C | -0.0775 | 0.0115 | 1.51E-11 | 36033.86 | SCZ | 45.41 | 0.001259 |
| rs1339227 | T | C | -0.0636 | 0.0114 | 2.64E-08 | 36033.86 | SCZ | 31.12 | 0.000863 |
| rs217289 | A | G | -0.0664 | 0.011 | 1.58E-09 | 36033.86 | SCZ | 36.43 | 0.00101 |
| rs117074560 | T | C | -0.16579 | 0.0267 | 5.46E-10 | 36033.86 | SCZ | 38.56 | 0.001069 |
| rs12532143 | T | C | 0.080796 | 0.0115 | 2.33E-12 | 36033.86 | SCZ | 49.36 | 0.001368 |
| rs10954580 | A | G | 0.064204 | 0.0114 | 1.64E-08 | 36033.86 | SCZ | 31.72 | 0.00088 |
| rs58120505 | T | C | 0.082704 | 0.0111 | 7.33E-14 | 36033.86 | SCZ | 55.51 | 0.001538 |
| rs12704290 | A | G | -0.1053 | 0.0168 | 3.48E-10 | 36033.86 | SCZ | 39.29 | 0.001089 |
| rs73191547 | A | T | -0.06731 | 0.0115 | 4.58E-09 | 36033.86 | SCZ | 34.25 | 0.00095 |
| rs7815859 | A | C | 0.083302 | 0.0134 | 4.33E-10 | 36033.86 | SCZ | 38.65 | 0.001071 |
| rs4129585 | A | C | 0.078099 | 0.0109 | 7.7E-13 | 36033.86 | SCZ | 51.34 | 0.001423 |
| rs78895722 | C | G | -0.1841 | 0.0318 | 7.34E-09 | 34888.33 | SCZ | 33.52 | 0.00096 |
| rs57023171 | T | C | -0.06481 | 0.0113 | 9.13E-09 | 36033.86 | SCZ | 32.89 | 0.000912 |
| rs35612961 | A | G | -0.0773 | 0.014 | 3.37E-08 | 36033.86 | SCZ | 30.49 | 0.000845 |
| rs7033189 | A | G | -0.0656 | 0.0116 | 1.55E-08 | 36033.86 | SCZ | 31.98 | 0.000887 |
| rs7085104 | A | G | 0.097399 | 0.0114 | 1.37E-17 | 36033.86 | SCZ | 73 | 0.002022 |
| rs7893279 | T | G | 0.113802 | 0.0175 | 7.33E-11 | 36033.86 | SCZ | 42.29 | 0.001172 |
| rs72829007 | T | G | 0.169701 | 0.0292 | 6E-09 | 36033.86 | SCZ | 33.78 | 0.000936 |
| rs12420205 | T | C | 0.0766 | 0.0119 | 1.39E-10 | 36033.86 | SCZ | 41.43 | 0.001149 |
| rs10791097 | T | G | 0.076896 | 0.0109 | 1.61E-12 | 36033.86 | SCZ | 49.77 | 0.001379 |
| rs3758927 | C | G | 0.089603 | 0.0136 | 4.27E-11 | 36033.86 | SCZ | 43.41 | 0.001203 |
| rs11027857 | A | G | 0.064401 | 0.0109 | 2.95E-09 | 36033.86 | SCZ | 34.91 | 0.000968 |
| rs7951870 | T | C | -0.09199 | 0.0144 | 1.58E-10 | 36033.86 | SCZ | 40.81 | 0.001131 |
| rs4766428 | T | C | 0.0656 | 0.011 | 2.76E-09 | 36033.86 | SCZ | 35.57 | 0.000986 |
| rs1615350 | T | C | -0.0861 | 0.0123 | 2.28E-12 | 36033.86 | SCZ | 49 | 0.001358 |
| rs1024582 | A | G | 0.099302 | 0.0114 | 2.71E-18 | 36033.86 | SCZ | 75.88 | 0.002101 |
| rs672107 | A | T | -0.0632 | 0.0114 | 3.02E-08 | 36033.86 | SCZ | 30.73 | 0.000852 |
| rs61937595 | T | C | -0.1444 | 0.0206 | 2.37E-12 | 36033.86 | SCZ | 49.14 | 0.001362 |
| rs35229468 | T | C | 0.085701 | 0.0119 | 6.62E-13 | 36033.86 | SCZ | 51.86 | 0.001437 |
| rs10149407 | T | C | 0.0656 | 0.0117 | 2.35E-08 | 36033.86 | SCZ | 31.44 | 0.000872 |
| rs221903 | T | C | 0.0656 | 0.0112 | 5.63E-09 | 36033.86 | SCZ | 34.31 | 0.000951 |
| rs2332700 | C | G | 0.077998 | 0.0125 | 4.27E-10 | 36033.86 | SCZ | 38.94 | 0.001079 |
| rs2414718 | A | G | 0.069498 | 0.011 | 2.35E-10 | 36033.86 | SCZ | 39.92 | 0.001107 |
| rs28681284 | T | C | -0.0991 | 0.0135 | 2.26E-13 | 36033.86 | SCZ | 53.89 | 0.001493 |
| rs783540 | A | G | -0.06119 | 0.011 | 2.49E-08 | 36033.86 | SCZ | 30.95 | 0.000858 |
| rs67119537 | T | C | 0.084396 | 0.0117 | 5.62E-13 | 36033.86 | SCZ | 52.03 | 0.001442 |
| rs4702 | A | G | -0.0785 | 0.0114 | 4.9E-12 | 36033.86 | SCZ | 47.42 | 0.001314 |
| rs1076884 | C | G | 0.076804 | 0.0127 | 1.55E-09 | 36033.86 | SCZ | 36.57 | 0.001014 |
| rs3814883 | T | C | -0.0719 | 0.0111 | 7.82E-11 | 36033.86 | SCZ | 41.96 | 0.001163 |
| rs12933068 | A | G | -0.0607 | 0.0109 | 2.88E-08 | 36033.86 | SCZ | 31.01 | 0.00086 |
| rs2955368 | A | G | -0.0652 | 0.0115 | 1.43E-08 | 36033.86 | SCZ | 32.14 | 0.000891 |
| rs4523957 | T | G | 0.065001 | 0.0114 | 1.25E-08 | 36033.86 | SCZ | 32.51 | 0.000901 |
| rs9636107 | A | G | -0.0798 | 0.0108 | 1.86E-13 | 36033.86 | SCZ | 54.6 | 0.001513 |
| rs72986630 | T | C | 0.144602 | 0.0239 | 1.47E-09 | 36033.86 | SCZ | 36.61 | 0.001015 |
| rs2916068 | A | G | -0.0646 | 0.0113 | 1.05E-08 | 36033.86 | SCZ | 32.68 | 0.000906 |
| rs2053079 | A | G | -0.0725 | 0.0128 | 1.32E-08 | 36033.86 | SCZ | 32.08 | 0.000889 |
| rs6065094 | A | G | -0.0751 | 0.0116 | 1.11E-10 | 36033.86 | SCZ | 41.92 | 0.001162 |
| rs35846931 | C | G | 0.817098 | 0.1773 | 4.05E-06 | 14307 | TS | 21.24 | 0.001483 |
| rs72734943 | A | G | 0.716102 | 0.1557 | 4.26E-06 | 14307 | TS | 21.15 | 0.001477 |
| rs117648881 | A | G | -0.5232 | 0.1064 | 8.79E-07 | 14307 | TS | 24.18 | 0.001687 |
| rs2788499 | A | T | 0.406398 | 0.0874 | 3.35E-06 | 14307 | TS | 21.62 | 0.001509 |
| rs4047771 | A | C | 0.140501 | 0.0299 | 2.66E-06 | 14307 | TS | 22.08 | 0.001541 |
| rs1865896 | A | G | -0.1215 | 0.0263 | 3.77E-06 | 14307 | TS | 21.34 | 0.00149 |
| rs10846381 | A | T | -0.1249 | 0.0267 | 2.98E-06 | 14307 | TS | 21.88 | 0.001527 |
| rs11075471 | T | C | -0.491 | 0.1059 | 3.53E-06 | 14307 | TS | 21.5 | 0.0015 |
| rs6670211 | A | C | -0.1263 | 0.0262 | 1.43E-06 | 14307 | TS | 23.24 | 0.001622 |
| rs4430756 | T | C | 0.1792 | 0.0385 | 3.19E-06 | 14307 | TS | 21.66 | 0.001512 |
| rs148879093 | A | G | 0.852499 | 0.1835 | 3.37E-06 | 14307 | TS | 21.58 | 0.001507 |
| rs117780640 | C | G | 0.208704 | 0.0441 | 2.18E-06 | 14307 | TS | 22.4 | 0.001563 |
| rs12459560 | T | G | 0.177502 | 0.036 | 8.24E-07 | 14307 | TS | 24.31 | 0.001697 |
| rs72853320 | A | G | 0.183396 | 0.0383 | 1.66E-06 | 14307 | TS | 22.93 | 0.0016 |
| rs150975336 | C | G | -0.4846 | 0.1028 | 2.42E-06 | 14307 | TS | 22.22 | 0.001551 |
| rs12703000 | A | T | 0.126298 | 0.0271 | 3.09E-06 | 14307 | TS | 21.72 | 0.001516 |
| rs66904072 | A | G | -0.3306 | 0.0704 | 2.64E-06 | 14307 | TS | 22.05 | 0.001539 |
| rs73205493 | T | C | 0.1501 | 0.0314 | 1.79E-06 | 14307 | TS | 22.85 | 0.001595 |
| rs2504235 | A | G | 0.149204 | 0.0266 | 2.11E-08 | 14307 | TS | 31.46 | 0.002195 |
| rs191044310 | A | T | -0.6227 | 0.1185 | 1.48E-07 | 14307 | TS | 27.61 | 0.001927 |
| rs77313409 | A | C | 0.436699 | 0.0927 | 2.5E-06 | 14307 | TS | 22.19 | 0.001549 |
| rs2708146 | A | G | 0.132702 | 0.026 | 3.24E-07 | 14307 | TS | 26.05 | 0.001818 |
| rs1906252 | A | C | -0.1285 | 0.0259 | 6.95E-07 | 14307 | TS | 24.62 | 0.001718 |

Abbreviation: A1: effect allele; A2: non-effect allele; R^2^: percent phenotypic variation; ADHD: Attention deficit/hyperactivity disorder; ALS: Amyotrophic lateral sclerosis; AD: Alzheimer’s dementia; AN: Anorexia nervosa; ASD: Autism spectrum disorders; AUD: Alcohol use disorders; BIP: Bipolar disorder; CUD: Cannabis use disorder; MDD: Major depressive disorder; OCD: Obsessive compulsive disorder; PTSD: Posttraumatic stress disorder; TS: Tourette’s syndrome.

## Supplementary Table 20. List of SNPs with genome-wide significance (*P* < 5×10^-8^) for potential confounding traits or disease searched from PhenoScanner and GWAS catalog.

| Trait-IVs | Num | snp | related phenotypes from PhenoScanner | related phenotypes from GWAS Catalog |
| --- | --- | --- | --- | --- |
| EA | 37 | rs1008078 | Townsend deprivation index at recruitment /// Body mass index | Smoking initiation (ever regular vs never regular) |
|  |  | rs1106761 | Alcohol intake frequency /// Waist circumference ///  Weight /// Whole body fat mass | Body mass index /// Intelligence |
|  |  | rs11130222 | Inflammatory bowel disease /// Body mass index ///  Hip circumference /// Time spent watching television ///  Waist circumference /// Ulcerative colitis | |
|  |  | rs11191193 | Fluid intelligence score | Raw vegetable consumption |
|  |  | rs11222416 | Body mass index /// Schizophrenia |  |
|  |  | rs11726992 | Pulse rate /// Time spent using computer |  |
|  |  | rs12410444 | Fluid intelligence score | Major depressive disorder vs ADHD (ordinary least squares (OLS)) |
|  |  | rs12514965 | Self-reported hypertension |  |
|  |  | rs12761761 | Time spent watching television |  |
|  |  | rs12962421 | Age at menarche /// Nap during day |  |
|  |  | rs12969294 | Neuroticism score /// Sensitivity or hurt feelings /// Time spent watching television /// Worrier or anxious feelings ///  Worry too long after embarrassment /// Neuroticism | Principal component-derived dietary pattern 1 |
|  |  | rs12987662 | Body mass index /// Hip circumference /// Waist circumference /// Rheumatoid arthritis | |
|  |  | rs13010288 | Sodium in urine | Insomnia |
|  |  | rs17824247 | Morning or evening person | Intelligence |
|  |  | rs2456973 | Allergic disease /// Alopecia areata /// Generalized vitiligo /// Vitiligo ///  Body mass index /// Pain type experienced in last month: headache ///  Self-reported asthma /// Type 1 diabetes ///  Type 1 diabetes autoantibodies /// Diabetes mellitus type 1 | Vitiligo |
|  |  | rs28792186 | Alcohol intake frequency ///Body mass index /// Fluid intelligence score ///  Sodium in urine /// Time spent using computer ///  Time spent watching television | |
|  |  | rs34305371 | | Insomnia /// Intelligence /// General cognitive ability |
|  |  | rs4468571 | Fed-up feelings /// Miserableness /// Neuroticism score | |
|  |  | rs4478846 | Time spent watching television /// Waist circumference ///  Whole body fat mass /// Schizophrenia | |
|  |  | rs4493682 | Age first birth |  |
|  |  | rs4800490 | Alcohol intake frequency /// Body mass index /// Hip circumference /// Usual walking pace /// Waist circumference | |
|  |  | rs4863692 |  | Body mass index |
|  |  | rs523934 | Time spent watching television | Smoking initiation |
|  |  | rs538628 | Parkinsons disease /// Alcohol intake frequency /// Irritability ///  Miserableness /// Mood swings ///  Mouth or teeth dental problems: mouth ulcers /// Nap during day /// Neuroticism score | Cortical surface area /// Feeling nervous /// Male-pattern baldness |
|  |  | rs61160187 | Time spent watching television |  |
|  |  | rs62100767 | Fed-up feelings /// Frequency of depressed mood in last 2 weeks ///  Frequency of tiredness or lethargy in last 2 weeks ///  Miserableness /// Mood swings | |
|  |  | rs62263923 | Basal metabolic rate /// Body fat percentage /// Body mass index ///  Ever smoked /// Hip circumference /// Past tobacco smoking /// Risk taking ///  Waist circumference /// Whole body fat-free mass /// Whole body water mas | |
|  |  | rs6839705 | Birth weight /// Birth weight of first child /// Fluid intelligence score /// | Haemorrhoidal disease |
|  |  | rs6882046 | Body mass index /// Neuroticism /// Alcohol intake frequency ///  Average weekly red wine intake ///  Body fat percentage /// Irritability ///  Job involves mainly walking or standing | Depression /// Intelligence /// Neuroticism ///  General cognitive ability /// Sunburns |
|  |  | rs7029201 | Bipolar disorder /// Basal metabolic rate /// Fluid intelligence score ///  Time spent using computer /// Time spent watching television ///  Whole body fat-free mass /// Whole body water mass | |
|  |  | rs71537331 | Diastolic blood pressure | |
|  |  | rs7599488 | Time spent watching television | Cigarettes smoked per day /// Schizophrenia ///  General cognitive ability |
|  |  | rs766406 | Asthma /// Disorders of mineral metabolism /// Hip circumference ///  Pack years of smoking preview only /// | Chronotype /// Asthma /// Height |
|  |  | rs8049439 | Body mass index /// Hip circumference /// Waist circumference ///  Weight /// Crohns disease /// Gene expression ///  Inflammatory bowel disease /// Inflammatory bowel disease early onset ///Alcohol intake frequency ///  Basal metabolic rate /// Body fat percentage /// Comparative body size at age 10 ///  Comparative height size at age 10 /// Fluid intelligence score ///  Types of physical activity in last 4 weeks: light diy /// Whole body fat mass ///  Whole body fat-free mass /// Whole body water mass /// Intelligence | Inflammatory bowel disease (early onset) ///  Verbal-numerical reasoning |
|  |  | rs9527702 | Body mass index |  |
|  |  | rs9556958 |  | Household income |
|  |  | rs9739070 | Allergy /// Basal metabolic rate /// Comparative height size at age 10 ///  Diastolic blood pressure /// Height /// Sitting height /// Whole body fat-free mass ///  Whole body water mass /// Time spent watching television | Allergy |
| AD | 9 | rs118170342 | Medication for cholesterol, blood pressure or diabetes: cholesterol lowering medication ///  Self-reported high cholesterol /// Treatment with cholesterol lowering medication | Total lipids in IDL (UKB data field 23524) ///  Cholesterol in small LDL (UKB data field 23547) ///  Total lipids in large LDL (UKB data field 23531) |
|  |  | rs148601586 | | Free cholesterol in small LDL (UKB data field 23549) ///  Apolipoprotein B levels |
|  |  | rs1859788 | Pulse rate |  |
|  |  | rs28394864 | Allergic disease /// Asthma /// Diastolic blood pressure /// Coronary artery disease | Triglyceride levels /// Body fat distribution (leg fat ratio) ///  Body fat distribution (trunk fat ratio) /// Total testosterone levels |
|  |  | rs28399657 | Self-reported high cholesterol /// Low density lipoprotein /// APOB apolipoprotein B /// LDL cholesterol | |
|  |  | rs41289512 | Self-reported high cholesterol /// Coronary artery disease | Total cholesterol levels (UKB data field 23400) |
|  |  | rs41290120 | Coronary artery disease /// Myocardial infarction /// Low density lipoprotein /// Total cholesterol | Concentration of medium LDL particles (UKB data field 23537) ///  Free cholesterol to total lipids in large LDL percentage (UKB data field 23617) ///  Total fatty acids levels (UKB data field 23442) |
|  |  | rs59735493 | Body fat percentage /// Hip circumference /// Waist circumference | |
|  |  | rs6931277 | Body mass index /Inflammatory bowel disease /// Ulcerative colitis ///  Primary sclerosing cholangitis /// Asthma /// Parkinsons disease | |
| ADHD | 6 | rs10262192 | Risk taking /// Types of physical activity in last 4 weeks: light diy | |
|  |  | rs1427829 | Body fat percentage /// Body mass index /// Hip circumference /// Waist circumference | Educational attainment (years of education) |
|  |  | rs17531412 | Age at menarche /// Age completed full time education ///  Fluid intelligence score /// Years of educational attainment | |
|  |  | rs212178 | Total cholesterol |  |
|  |  | rs4916723 | Alcohol intake frequency | Alcohol consumption (drinks per week) /// Alcohol consumption /// Drinks per week |
|  |  | rs4858241 |  | Insomnia /// Educational attainment /// Smoking initiation |
| AN | 4 | rs10747478 | Body fat percentage /// Body mass index /// Hip circumference /// Waist circumference | |
|  |  | rs725861 | Allergic disease /// Asthma /// Self-reported asthma /// Snoring | Snoring |
|  |  | rs750350 | Ever smoked /// Past tobacco smoking /// Smoking status: previous | |
|  |  | rs9821797 | Inflammatory bowel disease /// Qualifications: college or university degree | |
| ASD | 5 | rs10099100 | Neuroticism score /// Suffer from nerves |  |
|  |  | rs112635299 | Coronary artery disease | Alcohol consumption /// Coronary artery disease /// Total testosterone levels |
|  |  | rs1452075 | Waist circumference /// Arm fat mass right /// Hip circumference | Waist-hip ratio /// Educational attainment /// Body mass index |
|  |  | rs325485 | Nap during day /// Waist circumference | Triglyceride levels /// Multisite chronic pain |
|  |  | rs2391769 |  |  |
| AUD | 5 | rs1260326 | Type II diabetes /// Total cholesterol /// Triglycerides /// Fasting insulin | |
|  |  | rs13135688 | High density lipoprotein /// HDL cholesterol /// Body fat percentage ///  Body mass index /// Waist circumference | |
|  |  | rs4953148 | Irritability /// Pack years of smoking preview only /// Ever smoked | Chronic obstructive pulmonary disease liability (machine learning-based score) |
|  |  | rs7934481 | Body mass index ///Hip circumference /// Waist circumference | |
|  |  | rs9607805 | Allergic disease /// Neuroticism /// Current tobacco smoking ///  Neuroticism score /// Smoking status: current | Smoking cessation /// Neurociticism /// Insomnia |
| BIP | 6 | rs10744560 | Cross disorder /// Schizophrenia |  |
|  |  | rs111444407 | Body mass index |  |
|  |  | rs174592 | Low density lipoprotein /// Total cholesterol /// Triglycerides ///  Nap during day /// Pulse rate /// Asthma /// High density lipoprotein | Phosphatidylcholines (38:5)A levels /// Triacylglycerol (56:4) levels |
|  |  | rs2302417 | Body mass index /// Pulse rate /// Schizophrenia ///  Adiponectin levels /// Cross disorder /// Age at menarche | Pulse pressure /// Body mass index |
|  |  | rs71395455 | Schizophrenia |  |
|  |  | rs9834970 | Schizophrenia |  |
| CUD | 4 | rs11783093 | Alcohol intake versus 10 years previously /// Schizophrenia ///  Bipolar disorder lithium response categorical or schizophrenia | Schizophrenia |
|  |  | rs1392816 |  | Educational attainment (years of education) |
|  |  | rs719012 | Body mass index /// Hip circumference /// Waist circumference | |
|  |  | rs7783012 | Morning or evening person /// Risk taking /// Sleep duration ///  Types of physical activity in last 4 weeks: light diy /// Age first birth | |
| MDD | 4 | rs12129573 | Schizophrenia | Schizophrenia |
|  |  | rs6905391 | Schizophrenia |  |
|  |  | rs7531118 | Hip circumference /// Waist circumference /// Obesity | Obesity /// Drinks per week /// Anorexia nervosa, attention-deficit///  Intelligence /// Waist circumference |
|  |  | rs76025409 | Nap during day /// Sleeplessness or insomnia |  |
| OCD | 1 | rs4733767 |  | Colorectal cancer |
| PTSD | 1 | rs36127550 | | Coronary artery disease /// Osteoarthritis /// Waist-hip ratio |
| SCZ | 22 | rs10791097 | Body fat percentage /// Body mass index /// Hip circumference | |
|  |  | rs10954580 | Years of educational attainment |  |
|  |  | rs11682175 | Genetic generalised epilepsy /// sleep duration |  |
|  |  | rs12163529 | Nervous feelings /// Neuroticism score | |
|  |  | rs1233578 | /// Asthma  Autism spectrum disorder or schizophrenia /// Schizophrenia ///  Doctor diagnosed sarcoidosis ///  Fluid intelligence score /// Self-reported sarcoidosis | Chronic obstructive pulmonary disease ///  Age of onset of childhood onset asthma /// Asthma /// Intelligence |
|  |  | rs12420205 | Fed-up feelings /// Guilty feelings /// Miserableness ///  Mood swings /// Neuroticism score | Experiencing mood swings /// Depressed affect /// Feeling guilty |
|  |  | rs12474906 | Triglycerides |  |
|  |  | rs13071962 | Schizophrenia |  |
|  |  | rs13107325 | Body mass index /// High density lipoprotein /// HDL cholesterol ///  Systolic blood pressure /// Crohns disease | |
|  |  | rs1615350 | Years of educational attainment |  |
|  |  | rs217289 | Nap during day |  |
|  |  | rs28681284 | Cigarettes per day |  |
|  |  | rs3758927 |  | Intelligence |
|  |  | rs4129585 | Nervous feelings /// Time spent using computer | Neuroticism /// Systolic blood pressure |
|  |  | rs4391122 | Years of educational attainment |  |
|  |  | rs4481150 | Adiponectin levels /// Adiponectin levels female /// Body mass index | |
|  |  | rs6550435 |  | Bipolar disorder |
|  |  | rs7085104 | Body mass index males /// Basal metabolic rate ///  Ever smoked /// Hip circumference ///  Nervous feelings /// Past tobacco smoking | |
|  |  | rs533123 |  | Educational attainment |
|  |  | rs4702 |  | Opioid use disorder /// Insomnia |
|  |  | rs1076884 |  | Alcohol consumption (drinks per week) |
|  |  | rs3814883 |  | Triglyceride levels /// HDL cholesterol levels ///  High density lipoprotein cholesterol levels ///  Apolipoprotein A1 levels /// Insomnia |
| TS | 2 | rs1906252 | Years of education | Household income |
|  |  | rs2504235 | Body mass index | Body mass index |

Abbreviation: ADHD: Attention deficit/hyperactivity disorder; ALS: Amyotrophic lateral sclerosis; AD: Alzheimer’s dementia; AN: Anorexia nervosa; ASD: Autism spectrum disorders; AUD: Alcohol use disorders; BIP: Bipolar disorder; CUD: Cannabis use disorder; MDD: Major depressive disorder; OCD: Obsessive compulsive disorder; PTSD: Posttraumatic stress disorder; TS: Tourette’s syndrome

# Supplementary Figure

## Supplementary Figure 1. Annotation-specific Genetic Correlations (±SE) of EA with Neuropsychiatric Traits.

Genetic correlations were estimated by partitioned cross-trait LD score regression. * *P* values for genetic correlation < 0.05/ (13×13). EA: Educational attainment; ADHD: Attention deficit/hyperactivity disorder; ALS: Amyotrophic lateral sclerosis; AD: Alzheimer’s dementia; AN: Anorexia nervosa; ASD: Autism spectrum disorders; AUD: Alcohol use disorders; BIP: Bipolar disorder; CUD: Cannabis use disorder; MDD: Major depressive disorder; OCD: Obsessive compulsive disorder; PTSD: Posttraumatic stress disorder; SCZ: Schizophrenia; TS: Tourette’s syndrome; DGF=DNase digital genomic footprints; DHS=DNaseI hypersensitivity sites; FetalDHS=DNaseI hypersensitivity sites for fetal tissues; H3K4me1, H3K4me3, H3K9ac, and H3K27ac=Histone marks; TFBS= transcription factor binding sites.

A. Negative Genetic correlation of EA with Neuropsychiatric traits by Functional Category

B. Positive Genetic correlation of EA with Neuropsychiatric traits by Functional Category

A.


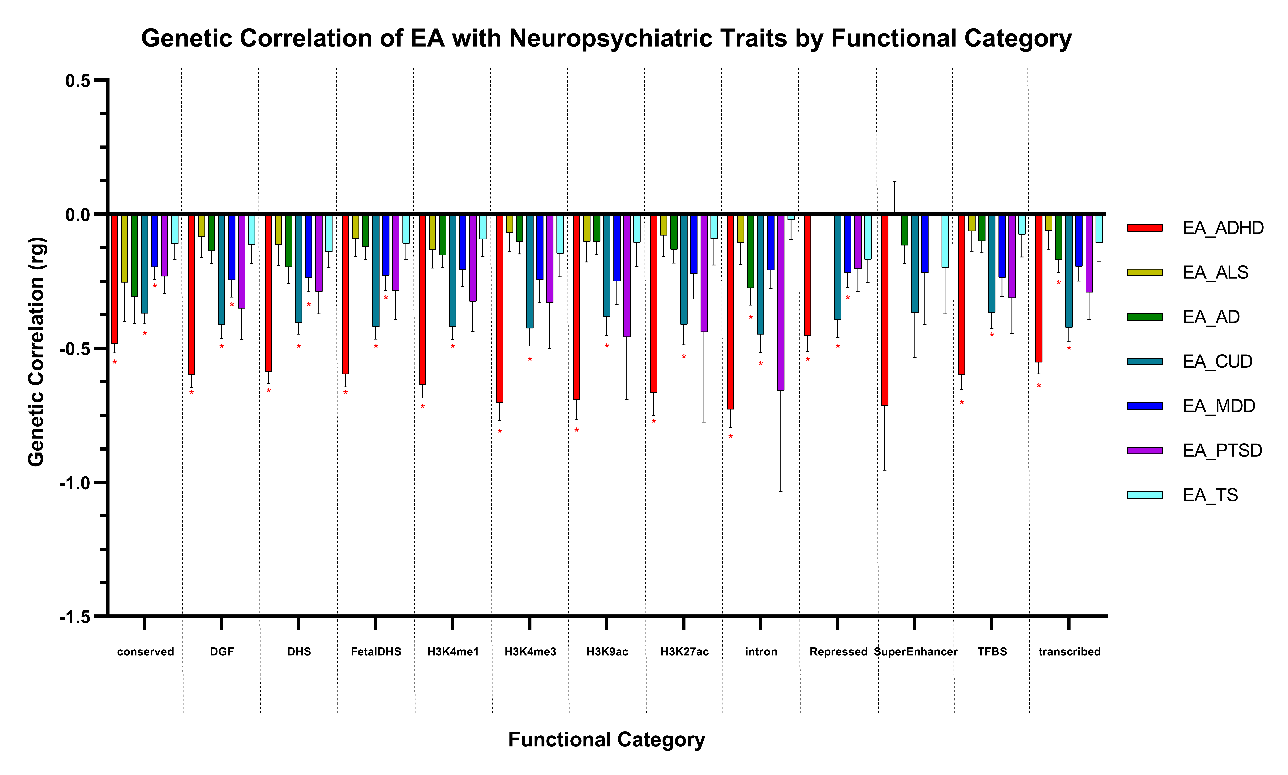


B.


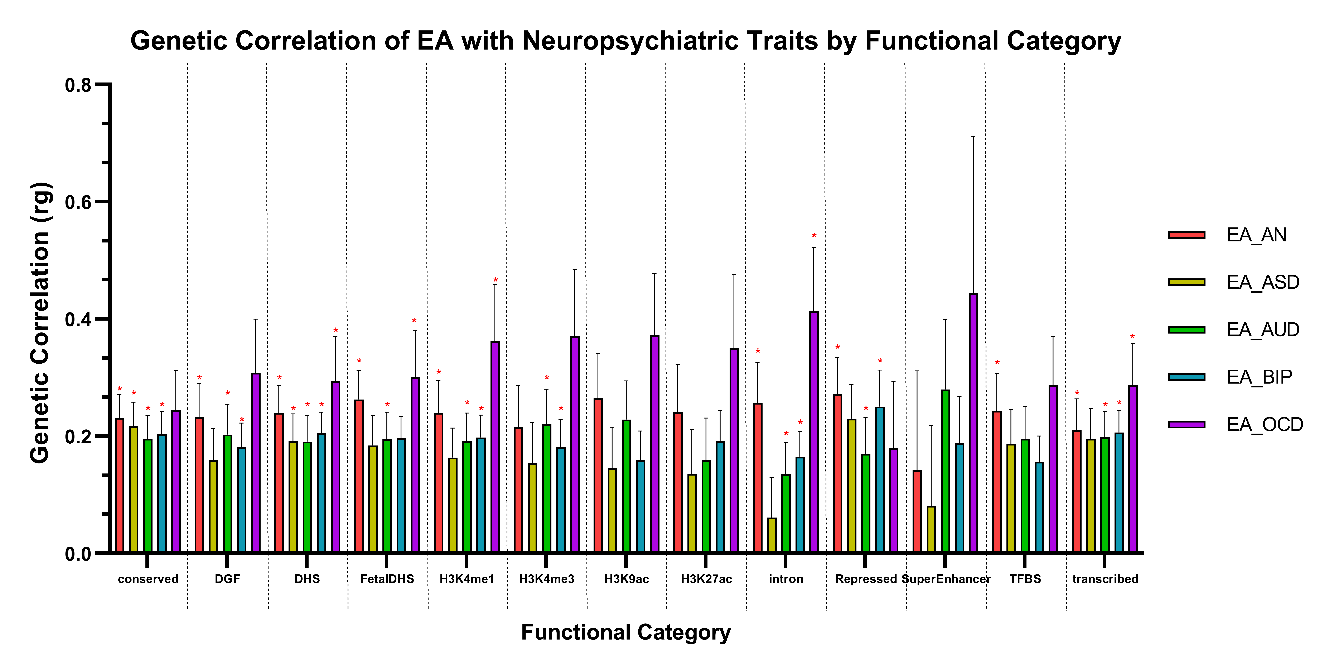


## Supplementary Figure 2. The QQ plot and inflation lambda for the cross-trait GWAS.


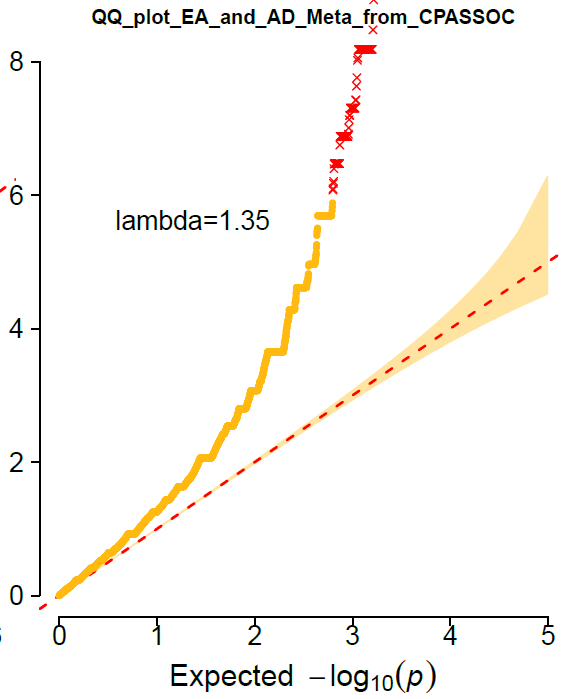

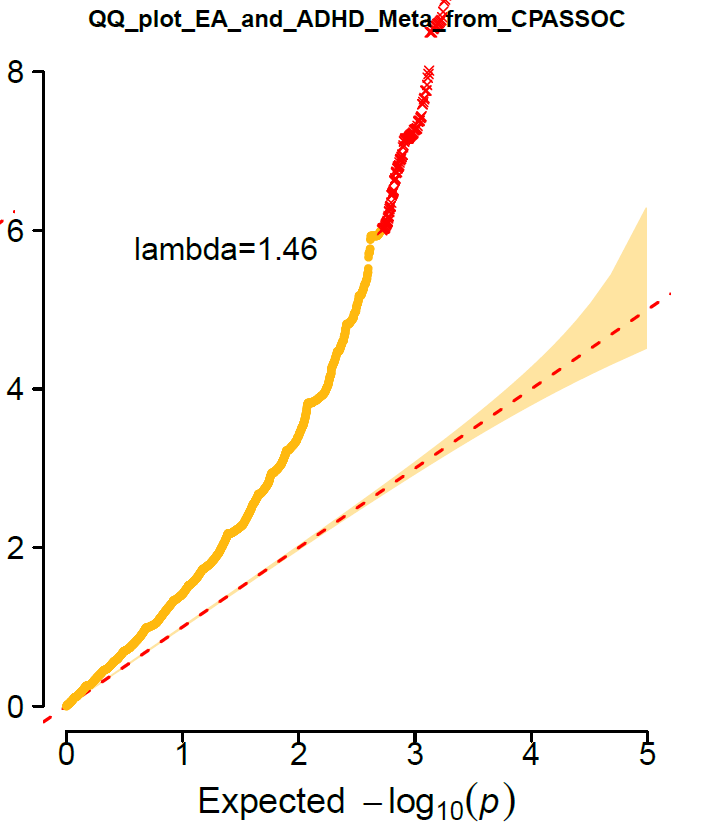

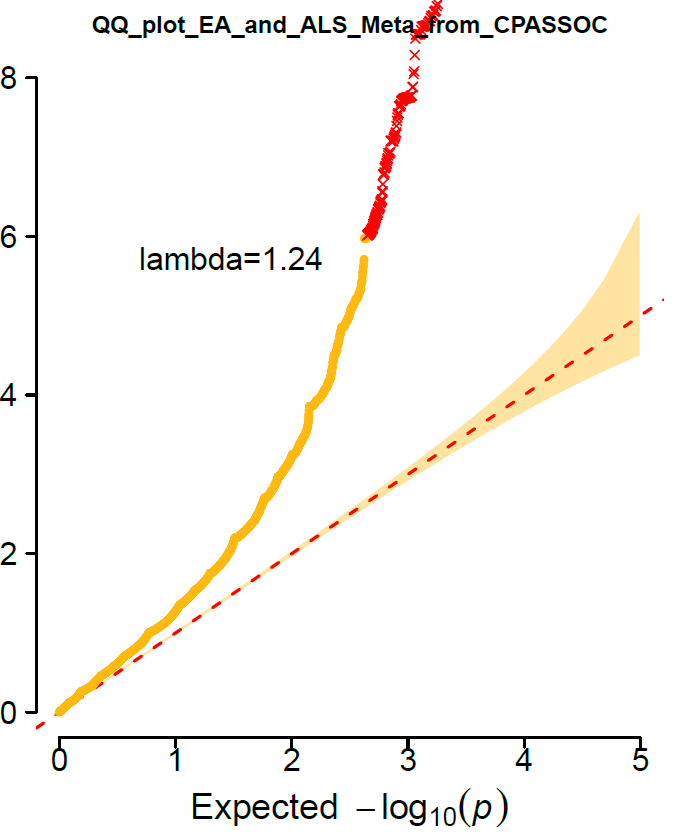


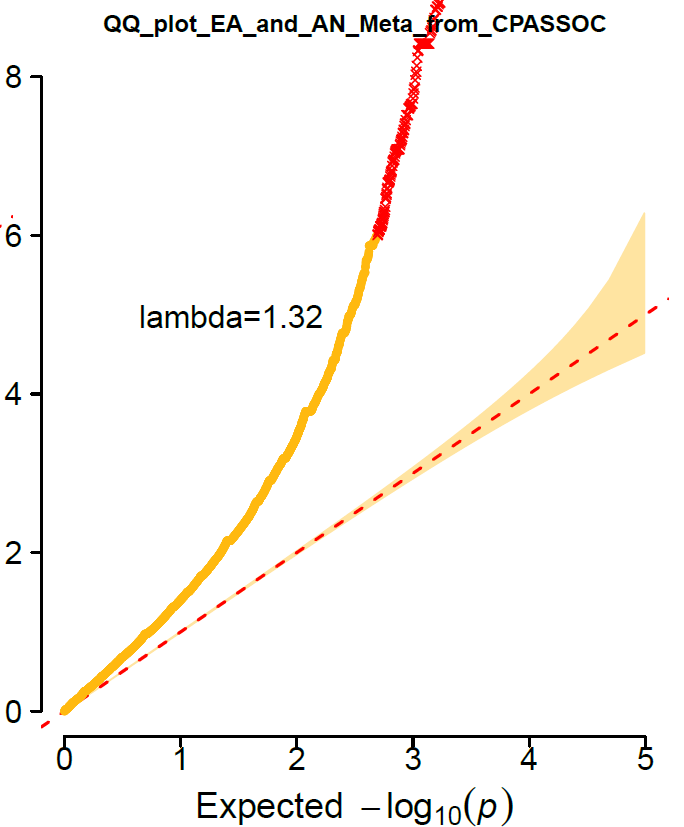

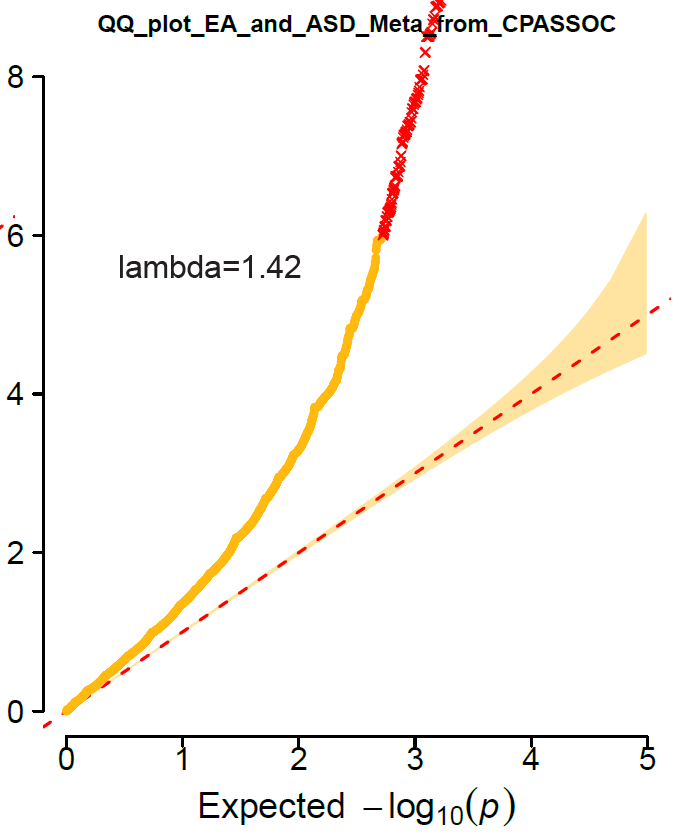

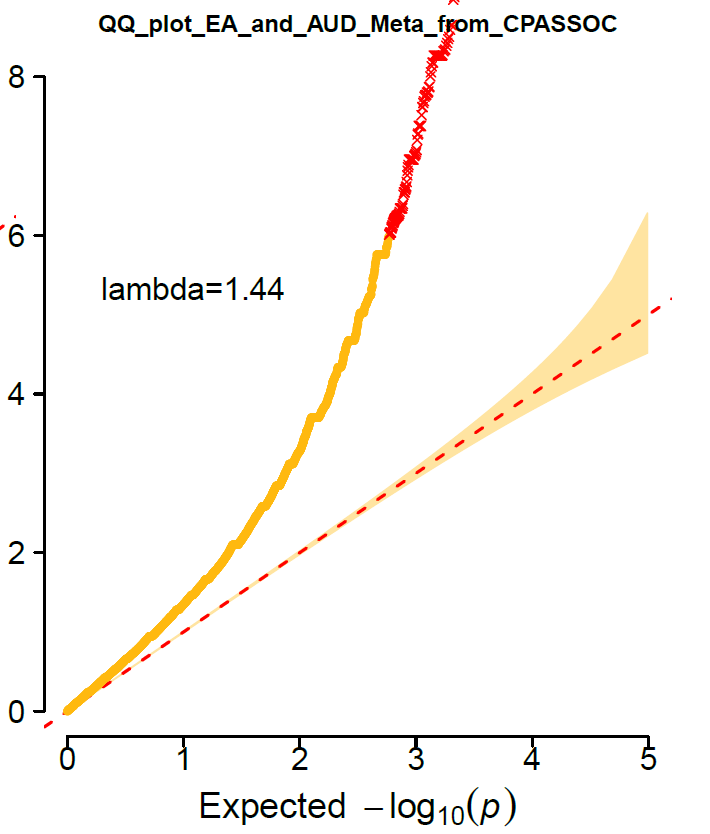


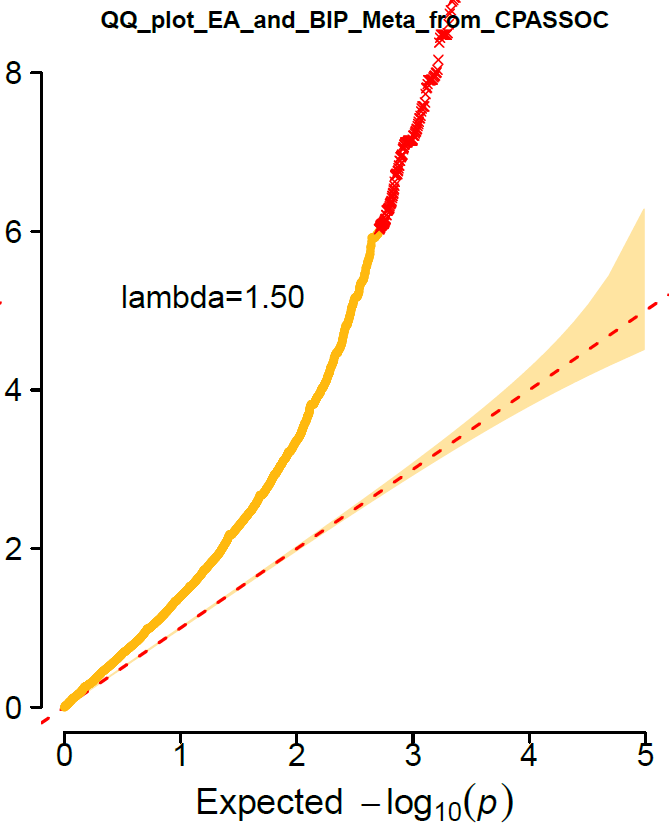

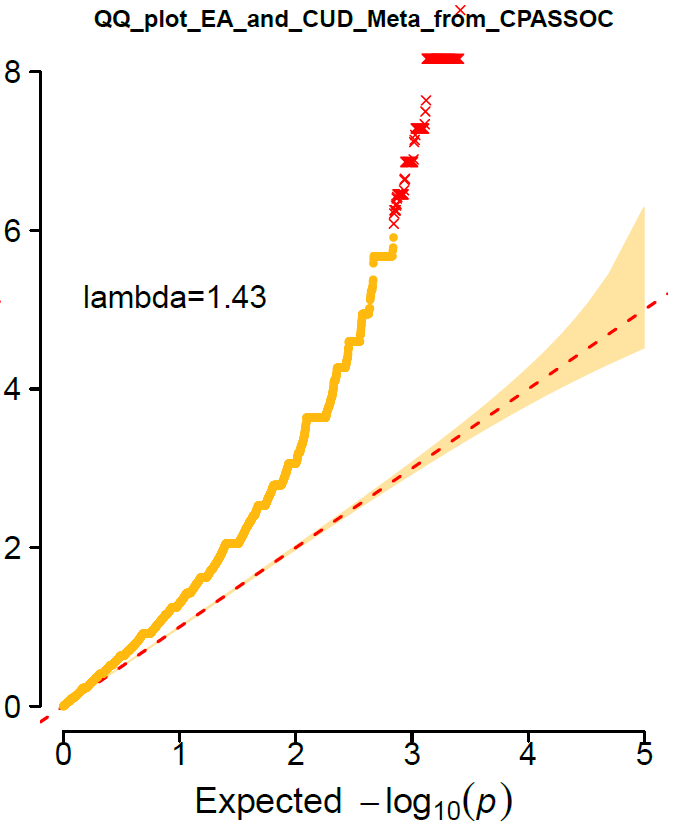

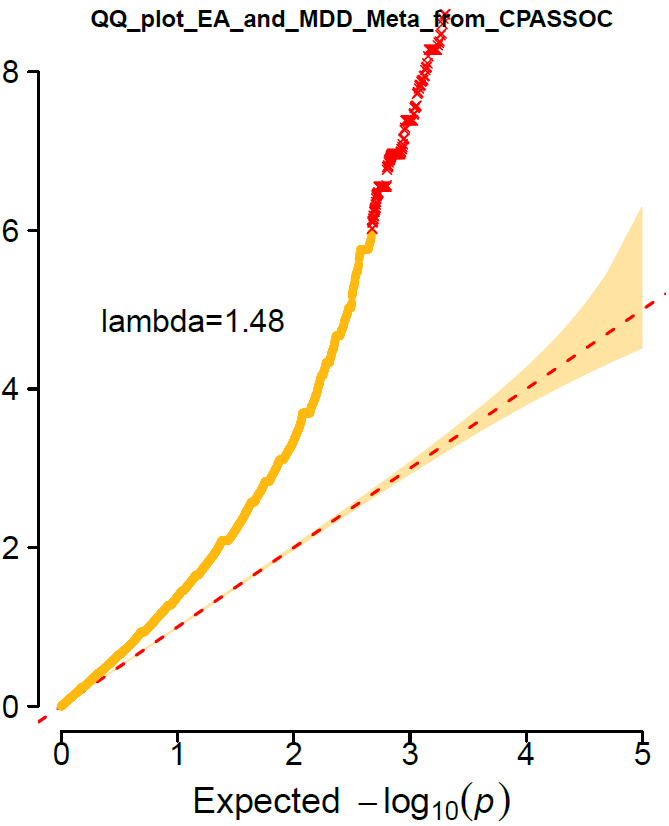


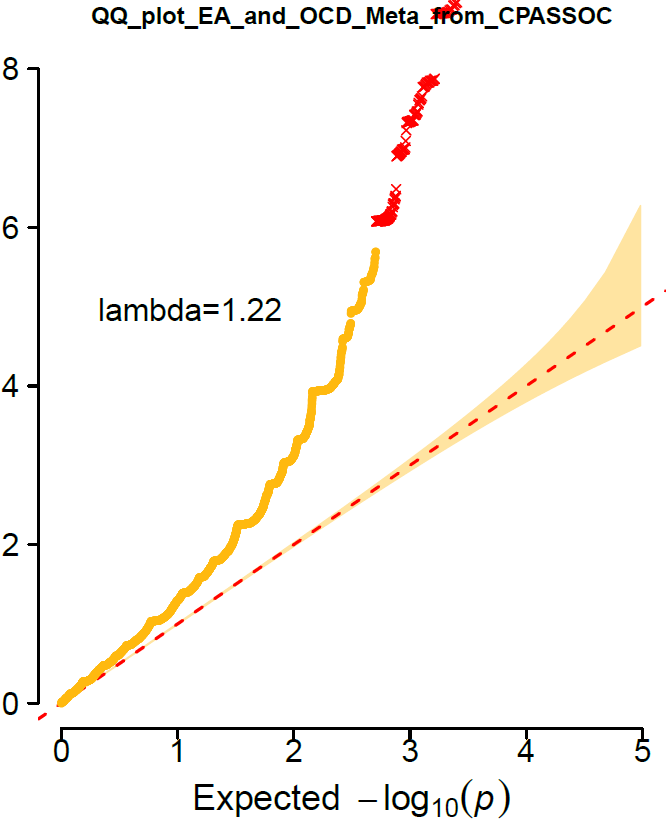

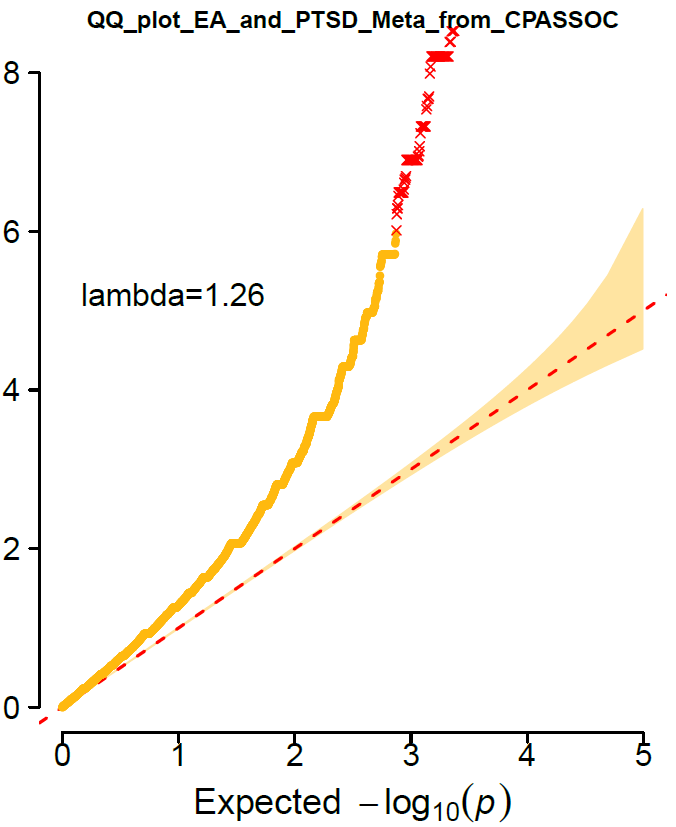

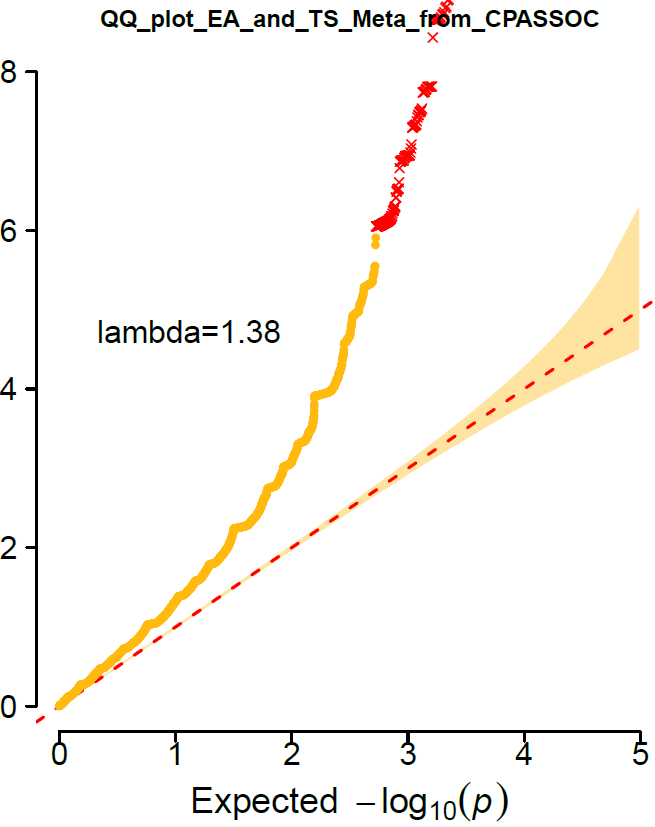


## Supplementary Figure 3. Number of significant expression-trait associations from transcriptome-wide association study (TWAS) for EA, AD, AUD, and BIP

The total number is the significant expression-trait associations after Bonferroni correction (false discovery rate < 0.05); GTEx: genotype-tissue expression project; EA: Educational attainment; AD: Alzheimer’s dementia; AUD: Alcohol use disorders; BIP: Bipolar disorder


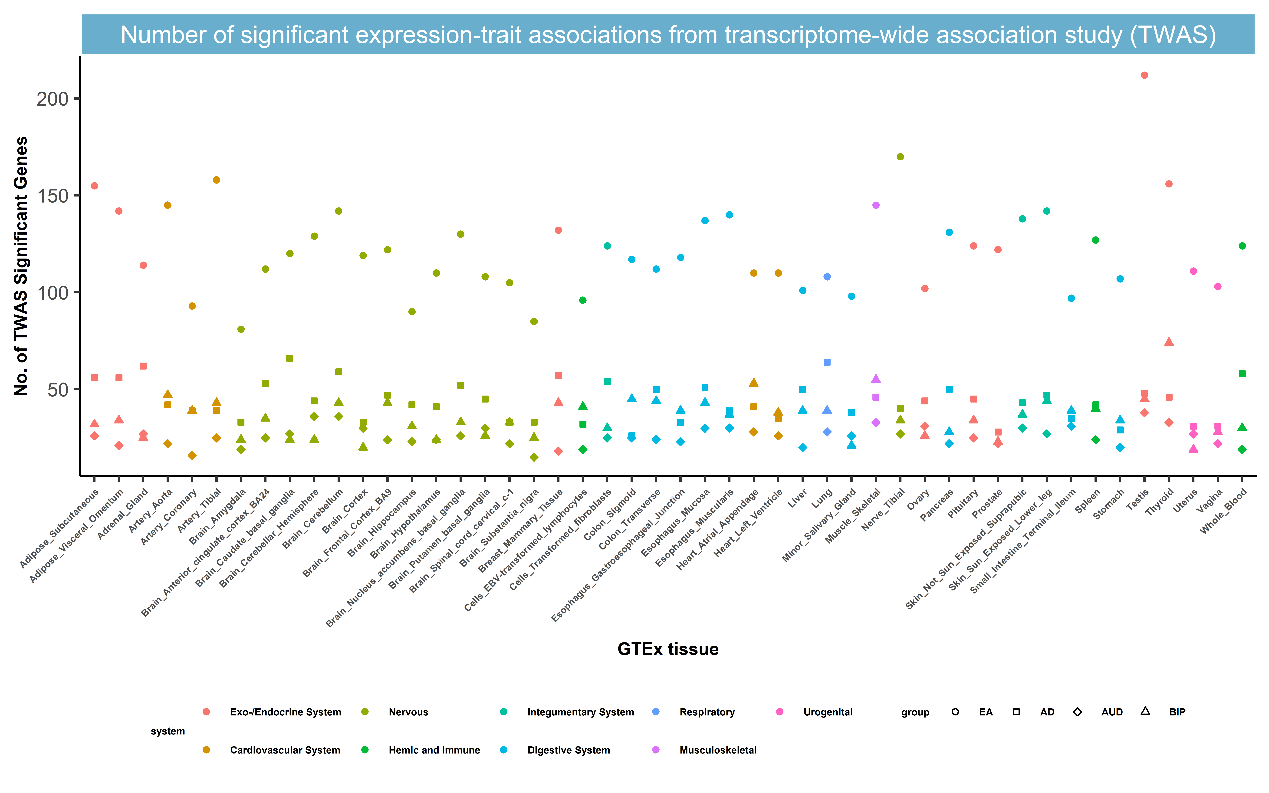


## Supplementary Figure 4. Number of significant expression-trait associations from transcriptome-wide association study (TWAS) for ADHD, AN, and ASD

The total number is the significant expression-trait associations after Bonferroni correction (false discovery rate < 0.05); GTEx: genotype-tissue expression project; ADHD: Attention deficit/hyperactivity disorder; AN: Anorexia nervosa; ASD: Autism spectrum disorders


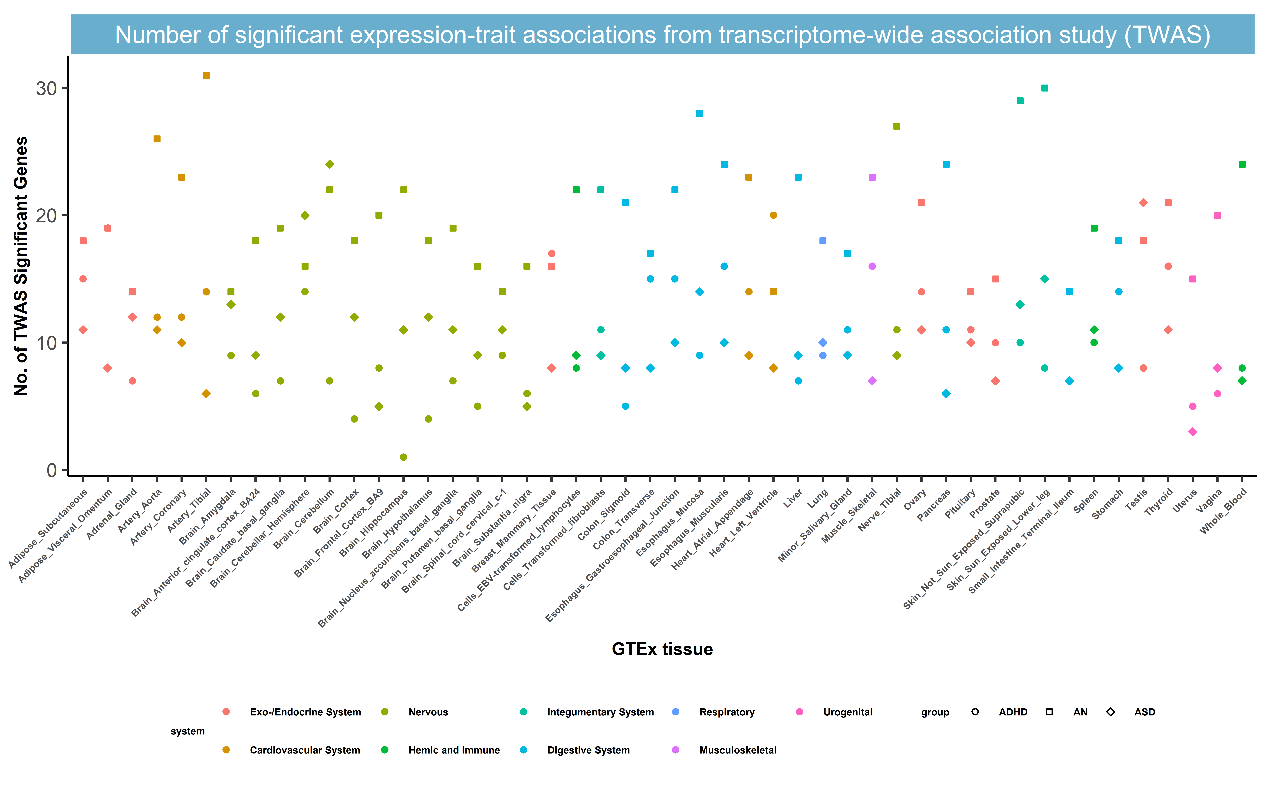


## Supplementary Figure 5. Number of significant expression-trait associations from transcriptome-wide association study (TWAS) for CUD and MDD

The total number is the significant expression-trait associations after Bonferroni correction (false discovery rate < 0.05); GTEx: genotype-tissue expression project; CUD: Cannabis use disorder; MDD: Major depressive disorder


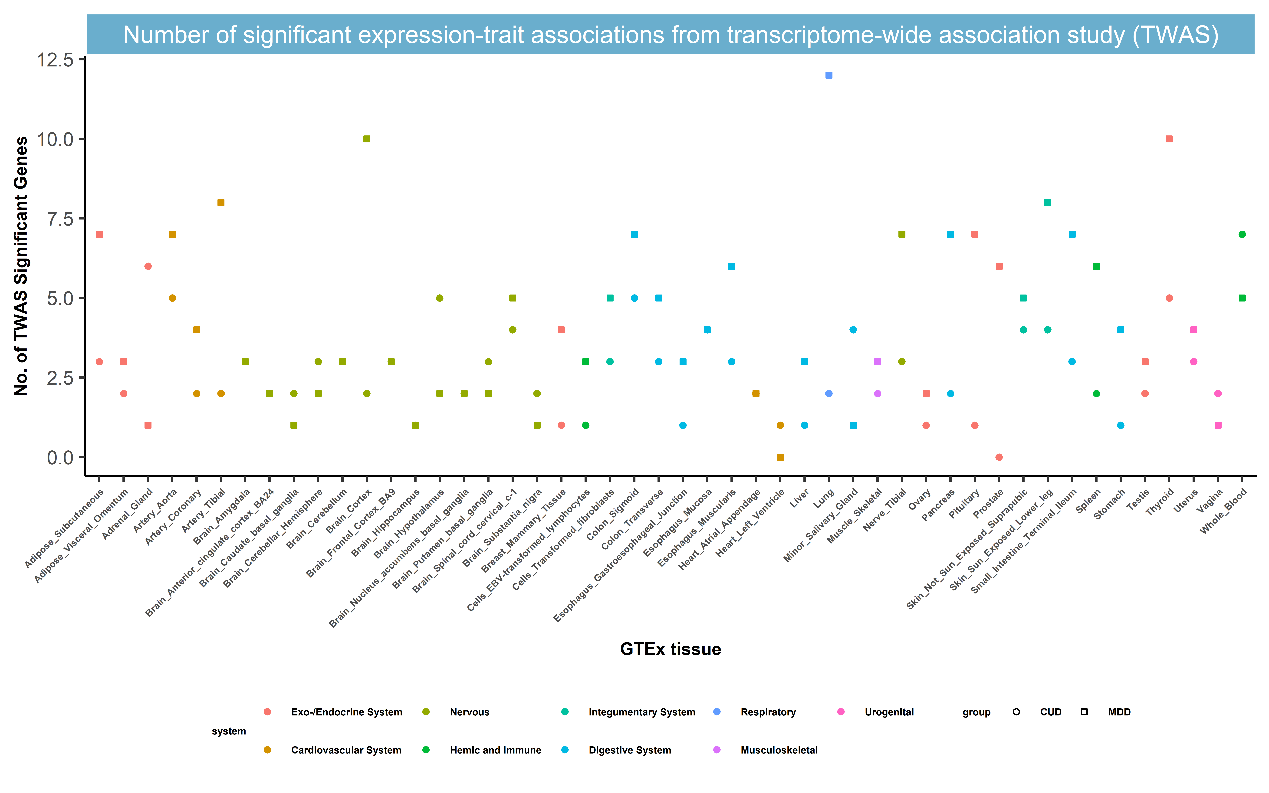

Supplement: Supplementary file 1 [file Data_Sheet_1.docx]
